# A new small-sized stem salamander from the Middle Jurassic of Western Siberia, Russia

**DOI:** 10.1371/journal.pone.0228610

**Published:** 2020-02-19

**Authors:** Pavel Skutschas, Veniamin Kolchanov, Sergey Krasnolutskii, Alexander Averianov, Rico Schellhorn, Julia Schultz, Thomas Martin

**Affiliations:** 1 Vertebrate Zoology Department, Saint Petersburg State University, Saint Petersburg, Russia; 2 Sharypovo Regional Museum, Sharypovo, Krasnoyarsk Territory, Russia; 3 Zoological Institute of the Russian Academy of Sciences, Saint Petersburg, Russia; 4 Russian Academy of Sciences, Borissiak Paleontological Institute, Moscow, Russia; 5 Institute of Geology and Petroleum Technology, Kazan Federal University, Kazan, Russia; 6 Section Paleontology, Institute of Geosciences, Rheinische Friedrich-Wilhelms-Universität Bonn, Bonn, Germany; University College London, UNITED KINGDOM

## Abstract

Salamanders (Caudata) are one of the three modern groups of amphibians known from the Middle Jurassic. The early stages of evolution of these amphibians are still poorly known, especially for stem taxa of Jurassic age. A new small-sized stem salamander, *Egoria malashichevi* gen. et sp. nov., from the Middle Jurassic (Bathonian) Itat Formation of the Berezovsk Quarry locality in Western Siberia, Russia, is described on the basis of isolated vertebrae, including an atlas centrum and a fragmentary trunk vertebra centrum previously referred to an undescribed salamander taxon (“Berezovsk salamander A”). The new taxon is diagnosed by the following unique combination of vertebral characters: atlantal anterior cotyles with elliptical anterior outline, located at an angle of approximately 135–137 degrees to each other; wide posterior portion of the atlantal centrum; ossified portion of the intercotylar tubercle represented by dorsal and ventral lips; absence of a deep depression on the ventral surface of the atlantal centrum; absence of pronounced ventrolateral ridges on the atlas; absence of spinal nerve foramina; presence of a pitted texture on the ventral and lateral surfaces of the centra and lateral surfaces neural arch pedicels; presence of a short atlantal neural arch with its anterior border situated behind the level of the anterior cotyles; short trunk vertebrae; and upper transverse process (= diapophysis) larger than lower transverse process (= parapophysis) on the trunk vertebrae; notochordal canal opens in the upper half of the cotyle (= the lower portion of the centrum is more massive and less compact than the upper portion). The microanatomical organization of the atlas and trunk vertebrae is characterized by the presence of inner cancellous endochondral bone. The small body size (about 180–215 mm) of *Egoria malashichevi* gen. et sp. nov. indicates that that not all stem salamanders were large neotenic forms (up to 550–600 mm in *Urupia* and *Marmorerpeton*) and hints at a broader ecological role for stem salamanders.

## Introduction

Salamanders (Caudata) are one of the three modern groups of amphibians with a superficially primitive external morphology (i.e. fore and hindlimbs limbs of nearly equal size and well-developed tail) and predominantly Laurasian distribution (e.g. [1, 2]). Salamanders first appear in the fossil record in the Middle Jurassic (Bathonian) and they are represented by both stem and crown taxa which may co-occur at the same localities [2–7].

Our knowledge of the early stages of salamander evolution and the origin of modern salamander clades is hampered by the scarcity of fossil material, especially for early diverging stem salamanders of Jurassic age [2]. Recent discoveries of stem salamanders in Jurassic and Cretaceous deposits of Siberia are beginning to fill geographic, temporal, taxonomic and anatomic gaps in the fossil record for the basal-most salamanders (e.g. [1]).

The Middle Jurassic (Bathonian) Itat Formation of the Berezovsk Quarry locality (= the Berezovsk coal mine) in Krasnoyarsk Territory, Western Siberia has produced remains of some of the geologically oldest known salamanders, including taxa of varying grades of evolution (namely both stem and crown salamanders) [1, 6,8,9]. One stem salamander genus, *Urupia* (with the type species *U*. *monstrosa*), and one crown salamander species, *Kiyatriton krasnolutskii*, were recently described based on isolated incomplete cranial and postcranial bones from the Berezovsk Quarry locality [6, 8].

In addition to the material assigned to *U*. *monstrosa*, fragmentary atlases and trunk vertebrae belonging to a stem salamander that differs from *Urupia* were informally attributed to an undescribed taxon “Berezovsk salamander A” [1].

In this paper, we describe all material of stem “Berezovsk salamander A” discovered to date from the Berezovsk Quarry locality, formally name this taxon and discuss some paleobiological aspects of stem salamanders.

Institutional abbreviations: DVZ, Department of Vertebrate Zoology, Saint Petersburg State University, Saint Petersburg, Russia. ZIN PH, Paleoherpetological collection, Zoological Institute, Russian Academy of Sciences, Saint Petersburg, Russia.

## Material and methods

To prepare figures and to gain microanatomical information, all described vertebral specimens of *Egoria malashichevi* gen. et sp. nov. and atlases of *Kokartus* and *Kulgeriherpeton* (used for comparisons) were CT-scanned (at 70 kV and 139 μA, generating a nominal resolution of 2.97 μm, with cubic voxels and an output of 2472 x 2472 pixels per slice for *Egoria malashichevi* gen. et sp. nov. and *Kokartus* and at 100 kV and 100μA, generating a nominal resolution of 4.48 μm, with cubic voxels and an output of 4000 × 4000 pixels per slice for *Kulgeriherpeton*) at the Saint Petersburg State University Research Centre for X-ray Diffraction Studies (Saint Petersburg, Russia) using a Skyscan 1172. Segmentation of the CT scan data and 3D model reconstructions were made with Amira 6.3.0 (FEIVSG Company).

For the body length estimates of the largest individuals of *Egoria malashichevi* gen. et sp. nov. and *Urupia monstrosa* we used skeletal proportions of the stem salamander *Karaurus* (atlantal centrum/total skeleton length ratio is about 1:56) and the modern cryptobranchid *Cryptobranchus alleganiensis* (atlantal centrum/total skeleton length ratio is about 1:48), and for the body length estimates of *Kiyatriton krasnolutskii* we used the skeletal proportions of the modern hynobiid *Ranodon sibiricus* (atlantal centrum/total skeleton length ratio is about 1:27) and the modern cryptobranchid *Cryptobranchus*. The skeletal proportions of *Karaurus* were measured on the basis of the holotype of *K*. *sharovi* housed in the Borissiak Paleontological Institute of the Russian Academy of Sciences, Moscow, Russia ([10], pers. obs.). The skeletons of a subadult *C*. *alleganiensis* (specimen DVZ M 2/12; total length of skeleton about 320–330 mm, atlantal length about 6.8 mm) and an adult *R*. *sibiricus* (specimen DVZ M 3/12; total length of skeleton about 96 mm, atlantal length about 3.5) used in this study (for the body length estimations of *Egoria malashichevi* gen. et sp. nov. and *Kiyatriton krasnolutskii*) are housed in the morphological collection of the Department of Vertebrate Zoology, Saint Petersburg State University, Saint Petersburg, Russia.

All published specimens of *Kiyatriton krasnolutskii* [6] used for the body length estimates of this species and all studied specimens of *Egoria malashichevi* gen. et sp. nov. are stored in the Paleoherpetological collection (ZIN PH) of the Zoological Institute of the Russian Academy of Sciences, Saint Petersburg, Russia.

The CT data used for digital restoration of specimens of *Egoria malashichevi* gen. et sp. nov.are available (see Supporting Information files) for the purpose of scientific study.

## Nomenclatural acts

The electronic edition of this article conforms to the requirements of the amended International Code of Zoological Nomenclature, and hence the new names contained herein are available under that Code from the electronic edition of this article. This published work and the nomenclatural acts it contains have been registered in ZooBank, the online registration system for the ICZN. The ZooBank LSIDs (Life Science Identifiers) can be resolved and the associated information viewed through any standard web browser by appending the LSID to the prefix "http://zoobank.org/". The LSID for this publication is: urn:lsid:zoobank.org:pub:BE35D3E0-32B8-4C9D-9BD7-2D6EB76884EC. The electronic edition of this work was published in a journal with an ISSN, and has been archived and is available from the following digital repositories: PubMed Central, LOCKSS.

## Systematic paleontology

Amphibia [11]

Caudata [12]

*Egoria* gen. nov.

urn:lsid:zoobank.org:act:1A6C7AA4-DB27-421C-8F1D-B4FD9CD0E193

Type species. *Egoria malashichevi* sp. nov.

Diagnosis. Referred to stem group salamanders based on the absence of spinal nerve foramina in the atlas, the presence of an anteroposteriorly short atlantal neural arch with an anterior border that is situated posteriorly from the level of the anterior cotyles, the presence of a pitted texture on the ventral and lateral surfaces of the atlas and trunk vertebrae. Differs from all other stem salamanders for which the morphology of the atlantal vertebral centrum is known (namely *Urupia* from the Berezovsk Quarry locality [8], *Kokartus* from the Middle Jurassic (Bathonian) of Kyrgyzstan[4, 13], *Marmorerpeton* from the Middle Jurassic (Bathonian) of United Kingdom [3], and *Kulgeriherpeton* the Early Cretaceous (Berriasian–Barremian) of Russia [14]) by a relatively wider posterior portion of the atlantal centrum (ratio of maximal anterior width/maximal posterior width about 1.9 vs. 2.3 in *Urupia*, 2.00–2.25 in *Kokartus*, 2.2–2.6 in *Marmorerpeton* and about 2.2 in *Kulgeriherpeton*) and by anterior cotyles with elliptical anterior outline, located at an angle of approximately 135–137 degrees to each other (vs. on the same horizontal plane in *Urupia*, *Marmorerpeton*, *Kulgeriherpeton* and 150–160 degrees in *Kokartus*) and forming a “heart-shaped” anterior outline of the atlantal centrum. Differs further from *Urupia*, *Marmorerpeton*, *Kulgeriherpeton* and *Kokartus* by absence of a depression on the ventral surface of the anterior portion of the atlantal centrum; from *Marmorerpeton* and *Urupia* by presence of an intercotylar tubercle on the atlas with ossified dorsal and ventral lips (vs. absence of the intercotylar tubercle and presence of only the notochordal central pit in *Urupia* and *M*. *freemani;* and undivided intercotylar tubercle in *M*. *kermacki*). Additionally, *Egoria* gen. nov. differs from *Urupia* by the lack of pronounced ventro-lateral ridges on the atlas, and by less dorso-ventrally compressed atlantal anterior cotyles (ratio of maximum height/width about 0.8 vs. about 0.5 in *Urupia*); from *Marmorerpeton* in the presence of atlantal transverse processes; and from *Kulgeriherpeton* by the lack of a transversal ridge and a depression on the ventral surface of the posterior portion of the centrum. Differs from all other stem salamanders for which the morphology of the trunk vertebral centra is known (namely *Urupia*, *Kokartus*, *Marmorerpeton*, and *Kulgeriherpeton*) in having the upper transverse process (= diapophysis) remarkably larger than the lower transverse process (= parapophysis); from *Urupia*, *Kokartus*, and *Kulgeriherpeton* in anteroposteriorly short trunk vertebral centra (ratio of ventral midline length: maximum centrum width about 1.2–1.3 vs. 1.4 in *Urupia*, 1.5–1.6 in *Kokartus* and 1.6 in *Kulgeriherpeton*).

Etymology. In honor of our friend and colleague, vertebrate zoologist Egor Malashichev, who over many years helped and supported us in developing paleontological research (including studies of stem salamanders) at the Vertebrate Zoology Department, Saint Petersburg, during many years and who recently passed away.

Remarks. Compared to other (but formally unnamed) stem salamanders known from the Middle Jurassic (Bathonian) of United Kingdom (“Kirtlington salamander A” sensu [15] see also [5]) and from the Early Cretaceous of Western Siberia, Russia (“Shestakovo stem salamander”, see [7]), *Egoria* gen. nov. is differentiated by the following features: (1) differs from both “Kirtlington salamander A” and “Shestakovo stem salamander in having an upper transverse process (= diapophysis) that is remarkably larger (namely more dorsoventrally expanded) than the lower transverse process (= parapophysis) (vs. diapophysis and parapophysis of the almost the same size in “Kirtlington salamander A” and “Shestakovo stem salamander”); (2) differs from “Shestakovo stem salamander” in having anteroposteriorly short trunk vertebral centra (vs. anteroposteriorly elongated in “Shestakovo stem salamander”); (3) differs from “Shestakovo stem salamander”in having an open notochordal canal (vs. closed notochordal canal in “Shestakovo stem salamander”, the presence/absence of the open notochordal canal is not described for “Kirtlington salamander A”); (4) differs from “Shestakovo stem salamander” in lacking of a subcentral depression on the ventral surface and a distinct tubercle (= ?anterior basapophysis) on the lateral surface of the centrum (vs. the presence of subcentral depression on the ventral surface and distinct tubercle on the lateral surface of the centrum in “Shestakovo stem salamander”).

*Egoria malashichevi* sp. nov.

urn:lsid:zoobank.org:act:0591C1C2-83E9-449F-B41B-F24DA0943C40

Figs 1–6A–6D.

**Fig 1:**
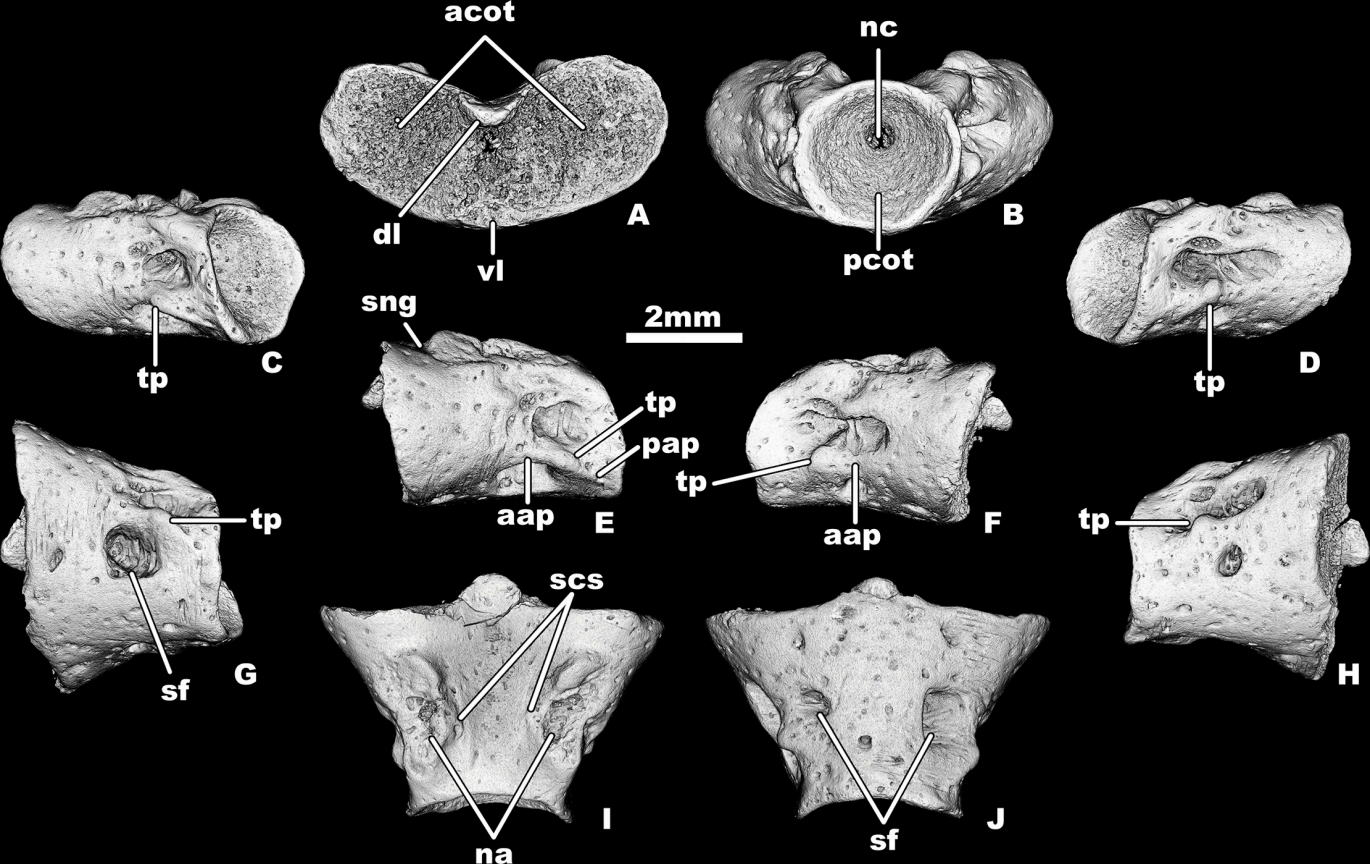
*Egoria malashichevi* gen. et sp. nov., digital restoration of atlantal centrum ZIN PH 40/144 (holotype), in anterior (A), posterior (B), posterior-left lateral (C), posterior-right lateral (D), left lateral (E), right lateral (F), ventral-left lateral (G),ventral-right lateral (H), dorsal (I) and ventral (J) views. Abbreviations: aap–anterior alar process, acot–anterior cotyle, dl–dorsal lip of intercotylar process, na–neural arch pedicel, nc–notochord canal, pap–posterior alar process, pcot–posterior cotyle, scs–spinal cord supports, sf–subcentral foramen, sng–spinal nerve groove, tp–transverse process, vl–ventral lip of intercotylar process. All images at same magnification.

**Fig 2:**
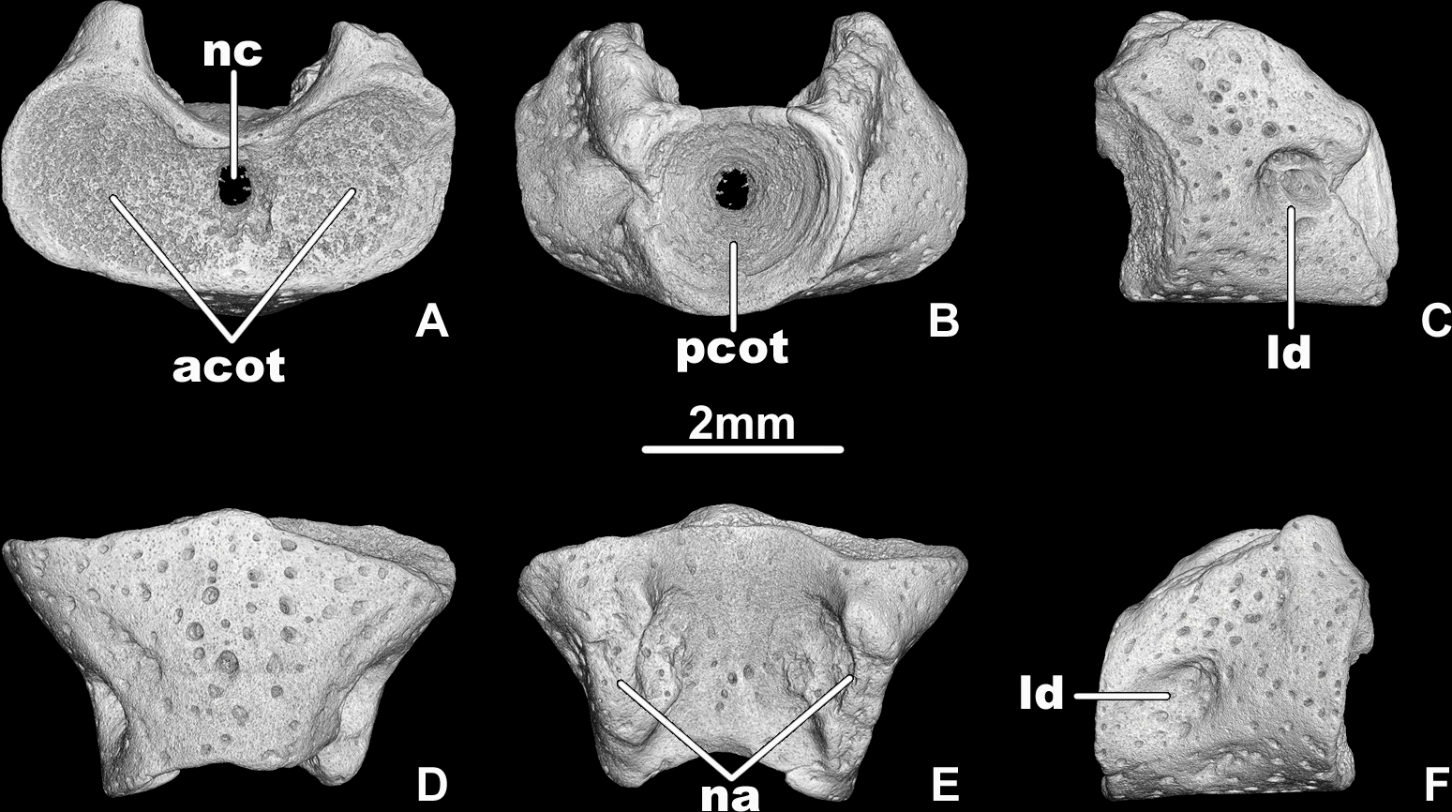
*Egoria malashichevi* gen. et sp. nov., digital restoration of atlantal centrum ZIN PH 5/144 (referred specimen), in anterior (A), posterior (B), left lateral (C), ventral (D), dorsal (E) and right lateral (F) views. Abbreviations: acot–anterior cotyle, ld–lateral depression, na–neural arch pedicel, nc–notochord canal, pcot–posterior cotyle. All images at same magnification.

**Fig 3:**
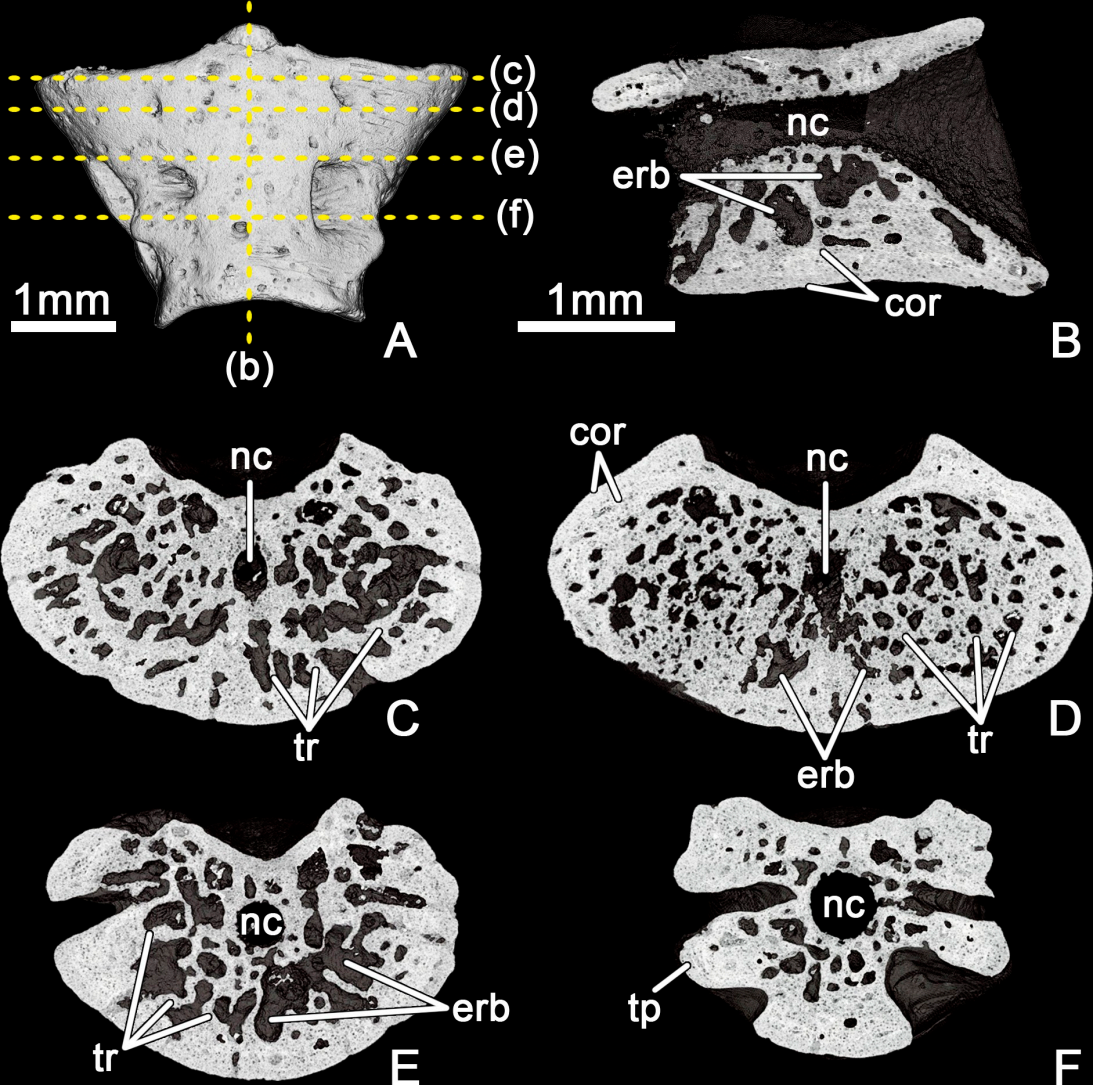
*Egoria malashichevi* gen. et sp. nov., digital restoration of atlantal centrum ZIN PH 40/144 (holotype), entire specimen in ventral view (A), showing locations of microCT digital sections: longitudinal section in vertical plane (B) and transverse sections from anterior to posterior (C–F). Abbreviations: cor–cortex, erb–erosion bay, nc–notochord canal, tp–transverse process, tr–trabecular bone. All images showing digital sections at same magnification.

**Fig 4:**
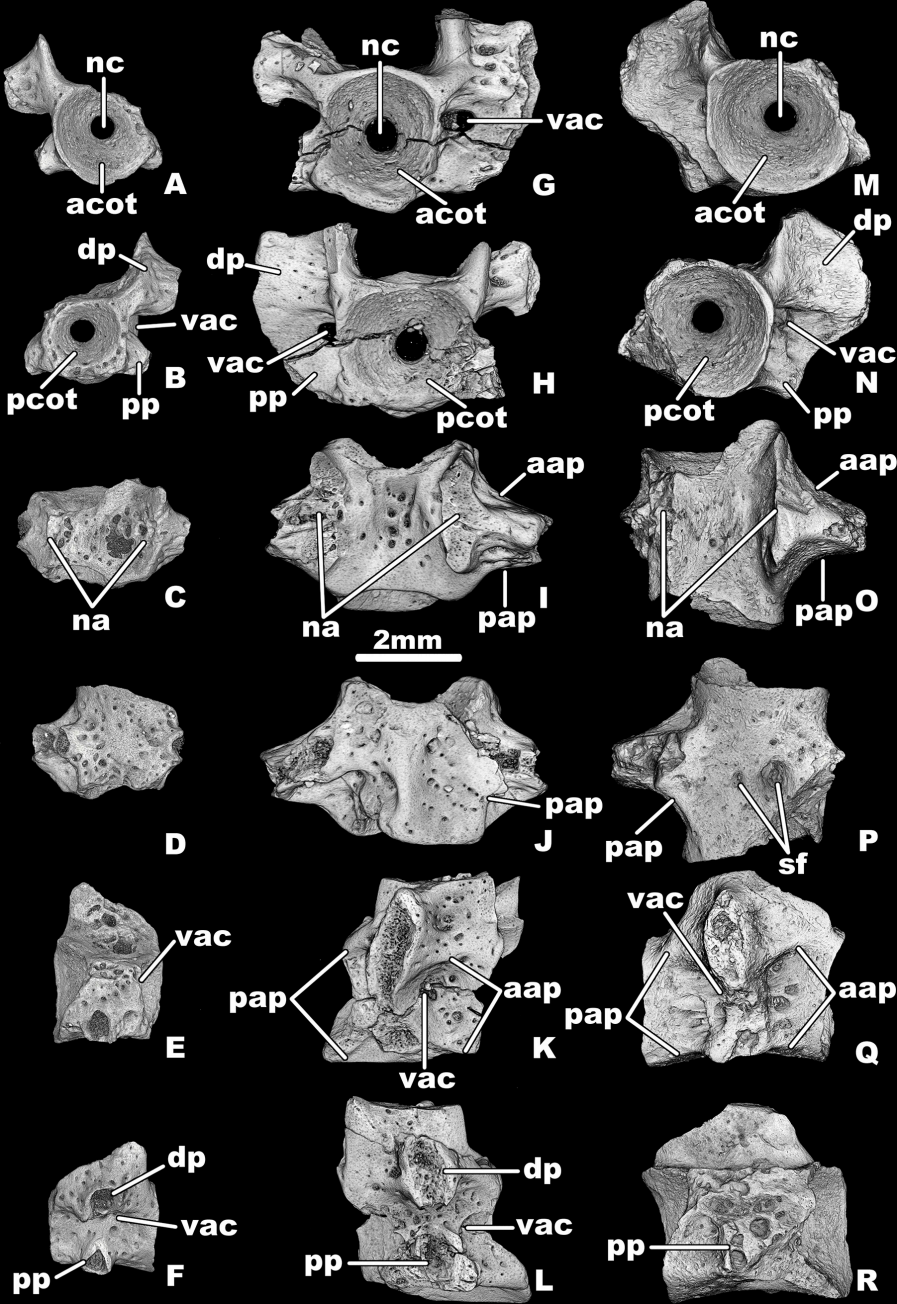
*Egoria malashichevi* gen. et sp. nov., digital restorations of three trunk vertebrae:A–F, ZIN PH 6/144 in anterior (A), posterior (B), dorsal (C), ventral (D), right lateral (E) and left lateral (F) views; G–L, ZIN PH 29/144 in anterior (G), posterior (H), dorsal (I), ventral (J), right lateral (K) and left lateral (L) views; and M-R, ZIN PH 32/144 in anterior (M), posterior (N), dorsal (O), ventral (P), right lateral (Q) and left lateral (R) views. Abbreviations: aap–anterior alar process, acot–anterior cotyle, dp–diapophysis, na–neural arch, nc–notochord canal, pap–posterior alar process, pcot–posterior cotyle, pp–parapophysis, sf–subcentral foramen, vac–vertebrarterial canal. All images at same magnification.

**Fig 5:**
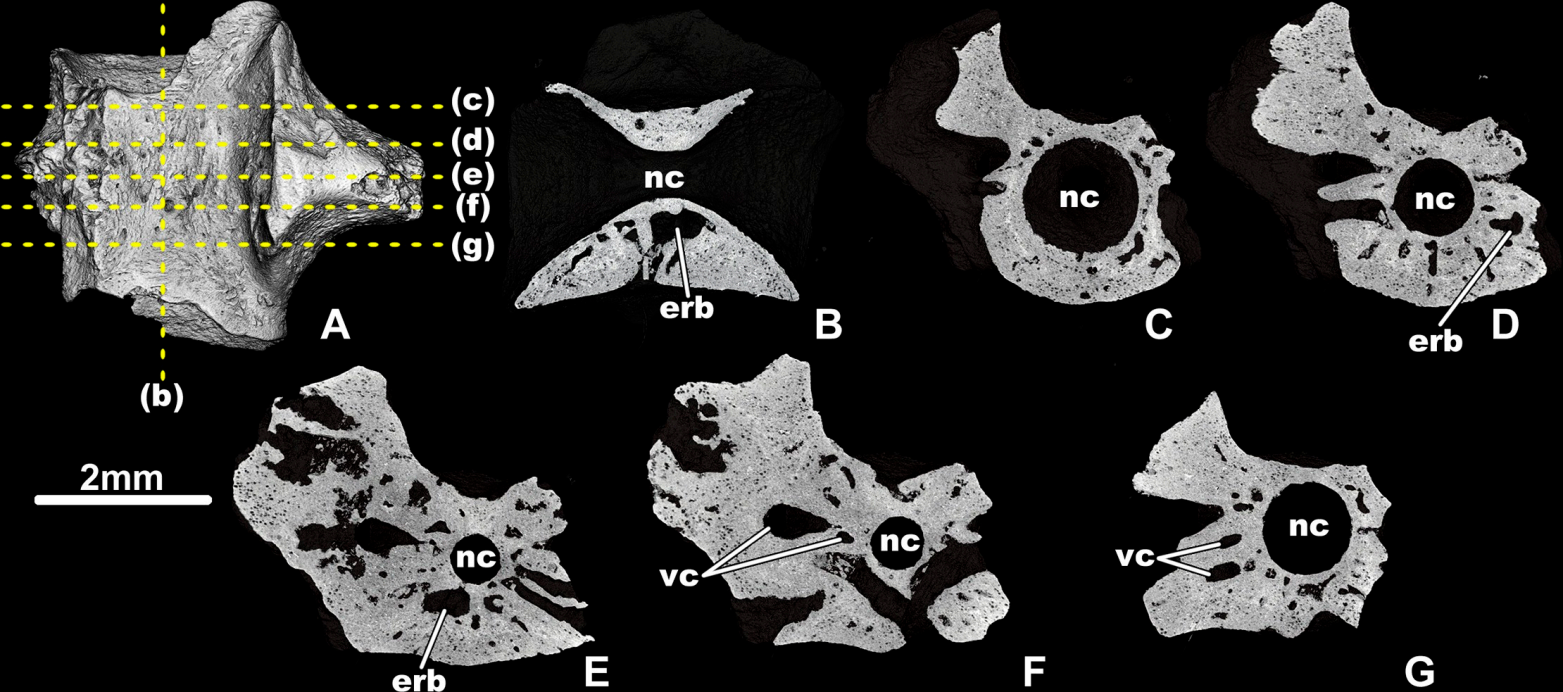
*Egoria malashichevi* gen. et sp. nov., digital restoration of trunk vertebra ZIN PH 32/144, entire specimen in dorsal view (A), showing locations of microCT digital sectionsin: longitudinal section in vertical plane (B) and transverse sections from anterior to posterior (C–G). Abbreviations: erb–erosion bay, nc–notochord canal, vc–vascular canal. All images showing digital sections at same magnification.

**Fig 6:**
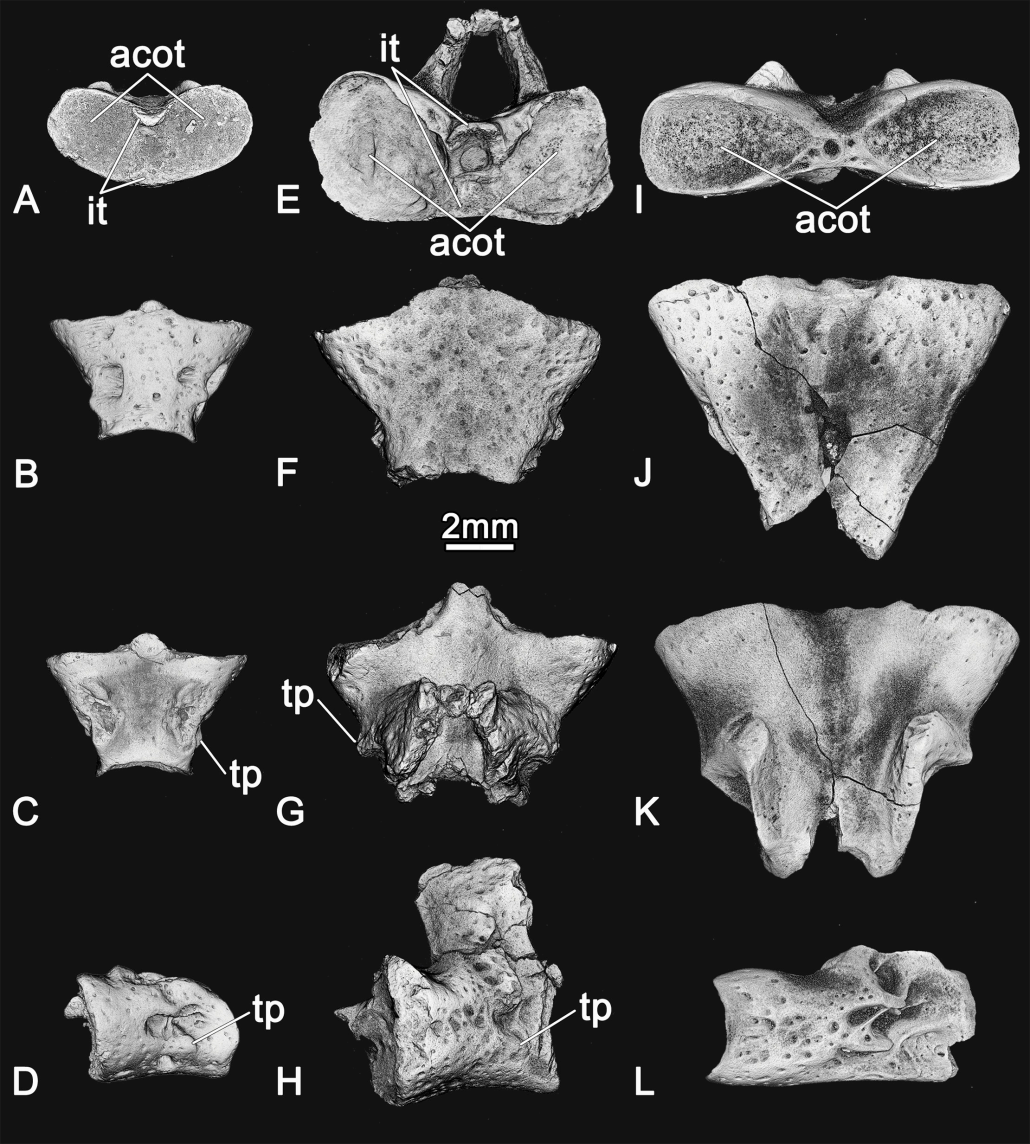
Digital reconstructions of atlases of representative stem group salamanders: A–D, *Egoria malashichevi* gen. et sp. nov., ZIN PH 40/144 (holotype) in anterior (A), ventral (B), dorsal (C) and left lateral (D) views; E–H, *Kokartus honorarius*, ZIN PH 3/47 (referred specimen) in anterior (E), ventral (F), dorsal (G) and left lateral (H) views; I–L, *Urupia monstrosa* ZIN PH 1/144 (holotype) in anterior (I), ventral (J), dorsal (K) and left lateral (L) views. Abbreviations: acot–anterior cotyle, it–intercotylar tubercle, tp–transverse process. All images at same magnification.

Synonym: “Berezovsk salamander B” ([1] p. 443, caption fig 3D–3F, 3J and 3K, table 1).

Holotype. ZIN PH 40/144, atlantal centrum.

Referred specimens. ZIN PH 5/144 –atlantal centrum, ZIN PH 6/144, 29/144, 32/144 –fragmentary trunk vertebrae.

Type locality and horizon. Berezovsk Quarry, near Nikol’skoe Village, Sharypovo District, Krasnoyarsk Territory, Russia; upper part of Itat Formation, Middle Jurassic (Bathonian).

Etymology. As for the genus.

Remarks. The above-listed fragmentary atlantal vertebral centrum (Fig 2) is comparable to the holotype atlantal centrum of *Egoria malashichevi* sp. nov. (Fig 1) in morphology (i.e. centra are short and relatively wide posteriorly, anterior cotyles with elliptical anterior outline forming a “heart-shaped” anterior outline of the atlantal centrum) and trunk vertebral centra (Fig 5) are comparable to the holotype atlantal centrum of *Egoria malashichevi* sp. nov. (Fig 1) in size and morphology (i.e. centra are short and relatively wide, deeply amphicoelous and with open notochordal canals, notochordal canal opens in the upper half of the posterior atlantal cotyle and both anterior and posterior cotyles of the trunk vertebrae) and, on that basis, are assigned to the same species.

We suggest that all largest specimens of *Egoria malashichevi* sp. nov. (the holotype atlantal centrum, and trunk vertebral centra) are from mature individuals for the following reasons: (1) these specimens have well developed morphological structures (e.g. ridges, processes, and tubercles) while in young individuals of salamanders, including stem taxa, they are less pronounced (see ontogenetic changes in morphology of the atlas of *Kokartus* in [13]), and (2) there are no specimens of larger size of *Egoria malashichevi* sp. nov. in our sample that can be referred to this new taxon, whereas *Urupia* are represented by vertebral specimens of all size classes (from very small, comparable in size with that of *Egoria malashichevi* sp. nov. to large adult individuals with estimated body length up to 550–600 mm).

Description. The atlas is known from two centra of different sizes (the maximum anterior width (i.e., between the lateral rims of the anterior cotyles) varies from 4.9–6.2 mm and the ventral midline length, excluding the intercotylar tubercle (= odontoid process), varies from 3–4 mm). The atlantal centrum (Figs 1 and 2) is wider than long, the ratio of the ventral midline length without intercotylar tubercle: the maximum anterior width about 1.6. The anterior cotyles (Figs 1 and 2) are elliptical in anterior outline (ratio of maximum height:width about 0.8) and they are located at an angle of approximately 135–137 degrees to each other, forming a “heart-shaped” anterior outline. The intercotylar tubercle is not fully ossified and is represented by dorsal and ventral lips that are separated by a depression with an open notochordal canal extending between the anterior cotyles. The articular surfaces of the anterior cotyles are moderately dorsoventrally concave. The posterior cotyle is nearly circular in posterior outline (Figs 1 and 2). The inner surface of the posterior cotyle is deeply concave and the notochordal canal opens in the upper half of the posterior cotyle (Figs 1 and 2).

The ventral surface of the centrum is flat or slightly convex with no depressions and has large subcentral foramina (Fig 1) Ventro-lateral and transverse ridges are absent.

The lateral surfaces of the smaller centrum (specimen ZIN PH 5/144, Fig 2) have a relatively shallow lateral depression and no transverse processes. In the larger and more complete holotype ZIN PH 40/144, distinct unipartite transverse processes are present (Fig 1). Similar variations (i.e. no transverse processes in smaller specimens vs. the presence of transverse processes in larger specimens) were described for the atlantal centra of *Kokartus* [13].

The transverse processes in ZIN PH 40/144 are short and do not project far from the lateral wall of the centrum. There are relatively deep lateral depressions on the lateral surfaces of the centrum ZIN PH 40/144 that are divided into dorsal and ventral portions by lateral ridges. These ridges (anterior and posterior alar processes) are associated with the transverse process. The anterior alar process is short, low and antero-posteriorly oriented, whereas the posterior alar process is relatively sharp, narrow and obliquely oriented. Hypapophyses and basapophyses are absent in both atlantal specimens.

The pedicels of the neural arch are massive and its anterior borders are situated behind the level of the anterior cotyle. The spinal nerve foramen is absent. There is a distinct groove for passage of the first spinal nerve on the anterior edge and the antero-lateral part of the pedicel of the neural arch. The small spinal cord supports are present in the neural canal at the base of the pedicel of the neural arch (Fig 1).

The ventral and lateral surfaces of the centrum and the lateral surfaces of the neural arch pedicels are rugose and indented by scattered, small, rounded and oval pits.

The internal microanatomical organization of the centrum is characterized by the presence of a relatively thin, compact, periosteal cortex and an inner cancellous endochondral bone with numerous erosion bays that are separated by irregularly arranged trabeculae of varying thickness (Fig 3). In the longitudinal section (vertical plane), the upper (dorsally to the notochondral canal) and lower (ventrally to the notochondral canal) parts of the centrum are microanatomically distinct (i.e. the lower portion of the centrum is more massive and less compact).

Three referred specimens (ZIN PH 6/144, 29/144, 32/144) collectively document the structure of the trunk vertebrae. The centrum is short and relatively wide (ratio of ventral midline length: maximum width is about 0.85). The centrum is deeply amphicoelous and has an open notochordal canal. The notochordal canal opens in the upper half of the cotyles. Both cotyles are deeply concave and circular in outline. Hypapophyses and basapophyses are absent. The subcentral keel on the ventral surface is low or absent. The ventral surface of the largest centra is pierced by one or two large subcentral foramina (Fig 4).

The transverse processes (= rib-bearers) are bicipitate, widely divergent, and connected by a webbing of bone. The upper transverse process (= diapophysis) is larger (more dorsoventrally expanded) than the lower transverse process (= parapophysis) and its base is situated at the level of the border between the centrum and the neural arch pedicel. The lower transverse process is situated relatively low and its base is situated at the level of the ventral surface of the centrum. The base of the transverse processes is perforated anteriorly and posteriorly by a vertebrarterial canal. No defined ridges are associated with the transverse processes on the smallest centrum ZIN PH 6/144, but on the largest specimens ZIN PH 29/144 and 32/144, short and low anterior and posterior ridges (= anterior and posterior alar processes) extend anteriorly and posteriorly from the upper and lower transverse processes, respectively, giving a total of four short alar ridges on each lateral surface.

The ventral and lateral surfaces of the centrum and the lateral surfaces of the neural arch pedicels are indented by scattered small rounded and oval pits.

The internal microanatomy of the trunk vertebrae is characterized by a more compact organization than in the atlas with a fewer number of erosion bays. In contrast to the atlas, the border between the periosteal cortex and the inner cancellous endochondral bone is not clearly visible on the microCT digital sections and the central part of the trunk centrum has few vascular canals that are radially arranged (Fig 5). Like in the atlantal centrum, the lower portion is more massive and less compact than the upper one (Fig 5).

## Discussion

*Egoria malashichevi* gen. et sp. nov. can be assigned to stem group salamanders on the basis of the following features: the absence of spinal nerve foramina in the atlas (vs. spinal nerve foramen fully enclosed by bone in crown salamanders); the presence of pitted ventral and lateral surfaces of the atlas (vs. smooth external vertebral surface in most crown salamanders); and an antero-posteriorly short neural arch with its anterior border situated behind the level of the anterior cotyles (vs. long neural arch with its anterior border situated at the level of the anterior cotyles in crown salamanders). All these characters are primitive for crown salamanders and characteristic for stem group salamanders (e.g. [7, 14]).

Among stem salamanders, the atlas of *Egoria malashichevi* gen. et sp. nov. most closely resembles that of the karaurid salamander *Kokartus* from the Middle Jurassic (Bathonian) of Kyrgyzstan in the presence of transverse processes, an intercotylar tubercle with ossified dorsal and ventral lips, the lack of pronounced ventro-lateral ridges, and the presence of slight dorsoventral compressions at the anterior cotyles ([13]; Fig 6). The trunk vertebrae referred to *Egoria malashichevi* gen. et sp. nov. are most similar to those of *Marmorerpeton* [3] in having anteroposteriorly short centra and open notochordal canals, but differ in being smaller and having larger upper transverse processes than lowers. The relationship of *Egoria malashichevi* gen. et sp. nov. with other stem salamanders is uncertain because there is limited information on the anatomy of the new taxon, and for most of the other stem salamanders (e.g. *Marmorerpeton*, *Urupia*, *Kulgeriherpeton*, and “Kirtlington salamander A”).

As currently understood, the vertebrate fauna of the Berezovsk Quarry locality contains three salamander taxa: the stem salamanders *Urupia monstrosa*, and *Egoria malashichevi* gen. et sp. nov., and the crown salamander *Kiyatriton krasnolutskii* with possible cryptobranchoid affinities ([1, 6, 8];this paper). The presence of three different salamander taxa in one vertebrate fauna suggests that those salamanders occupied different ecological niches. *Urupia monstrosa*, like all previously described stem salamanders, was a large (length of atlantal centrum excluding the intercotylar tubercle up to 10 mm, estimated body size between 480–560 mm using atlas/total skeleton length ratio of *Karaurus* and *Cryptobranchus*) neotenic form, whereas *Kiyatriton krasnolutskii* was considerebly smaller (length of atlas excluding the intercotylar tubercle up to 1.5 mm, estimated body size about 47–72 mm using atlas/total skeleton length ratio of *Ranodon* and *Cryptobranchus*; see discussion of body size and neoteny of Jurassic–Early Cretaceous salamanders in [7, 16,17]) and also probably a metamorphosed salamander sharing several skeletal features with modern hynobiids [6, 18]. *Egoria malashichevi* gen. et sp. nov. (length of atlas excluding the intercotylar tubercle up to 3 mm, estimated body size about 180–215 mm using atlas/total skeleton length ratio of *Karaurus* and *Cryptobranchus*) was larger than *Kiyatriton krasnolutskii* and significantly smaller than *Urupia monstrosa*. Several morphological characters (transverse processes on the atlas, not fully ossified intercotylar tubercle on the atlas, and open notochordal canal in the trunk vertebrae) support the neotenic nature of the new salamander. The finding of a smaller-sized stem salamander suggests that members of the stem-group were not only represented by large neotenics, as previously thought, but also occupied the ecological niche of small to medium-sized neotenic forms (at least in the Middle Jurassic). Possibly the absence of small-sized stem salamanders in other Middle Jurassic–Early Cretaceous vertebrate faunas is due to the fact that they were outcompeted by evolutionary more advanced crown salamanders.

## Supporting information

S1 File Scaled down image sequence of CT-reconstruction, *Egoria malashichevi* gen. et sp. nov., atlantal centrum ZIN PH 40/144 (holotype). (ZIP)

S2 File Scaled down image sequence of CT-reconstruction, *Egoria malashichevi* gen. et sp. nov., atlantal centrum ZIN PH 5/144. (ZIP)

S3 File Scaled down image sequence of CT-reconstruction, *Egoria malashichevi* gen. et sp. nov., trunk vertebra ZIN PH 6/144. (ZIP)

S4 File Scaled down image sequence of CT-reconstruction, *Egoria malashichevi* gen. et sp. nov., trunk vertebra ZIN PH 29/144. (ZIP)

S5 File Scaled down image sequence of CT-reconstruction, *Egoria malashichevi* gen. et sp. nov., trunk vertebra ZIN PH 32/144. (ZIP)
