# Supplementary figures and images for: A new small-sized stem salamander from the Middle Jurassic of Western Siberia, Russia (part 2 of 10)
Source: PLoS One. 2020 Feb 19;15(2):e0228610. doi: 10.1371/journal.pone.0228610 (PMC7029856; doi:10.1371/journal.pone.0228610)

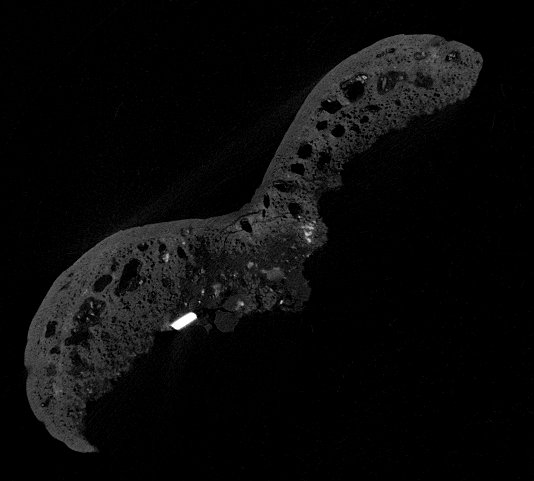

Supplement: S1 File — (ZIP) [file pone.0228610.s001.zip › 40_144/Br-11__IR_rec0746.jpg]

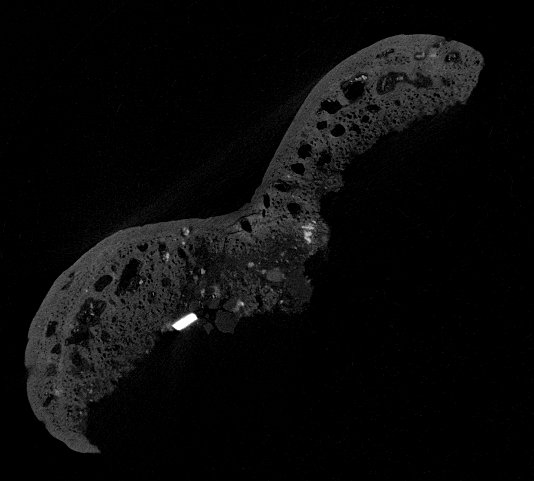

Supplement: S1 File — (ZIP) [file pone.0228610.s001.zip › 40_144/Br-11__IR_rec0750.jpg]

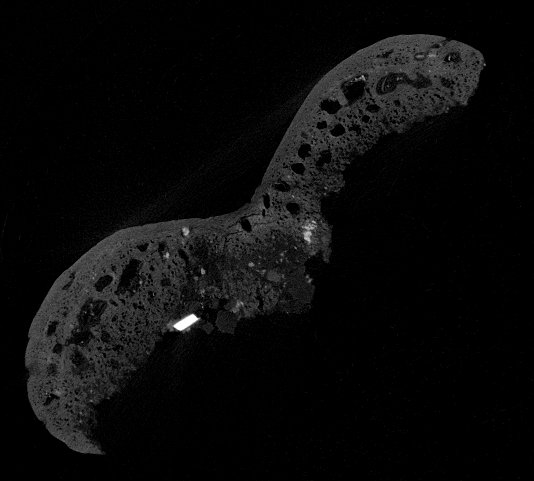

Supplement: S1 File — (ZIP) [file pone.0228610.s001.zip › 40_144/Br-11__IR_rec0754.jpg]

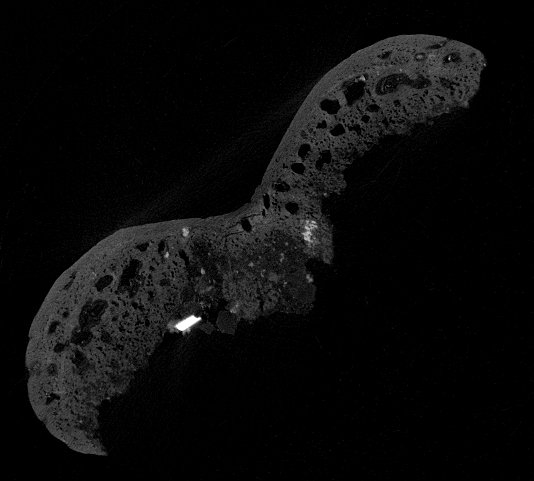

Supplement: S1 File — (ZIP) [file pone.0228610.s001.zip › 40_144/Br-11__IR_rec0758.jpg]

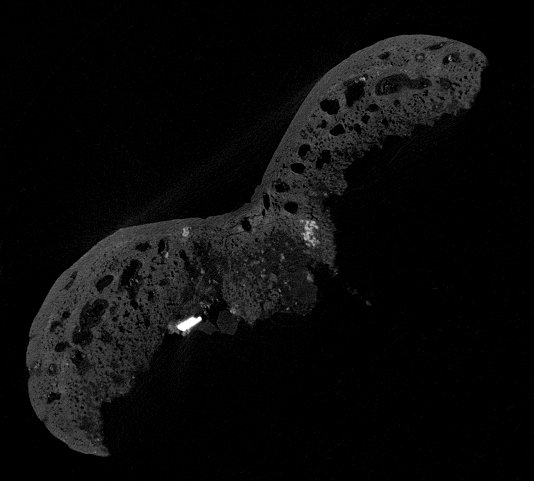

Supplement: S1 File — (ZIP) [file pone.0228610.s001.zip › 40_144/Br-11__IR_rec0762.jpg]

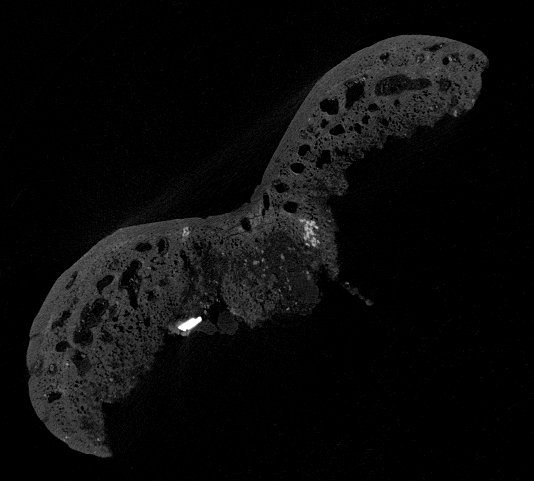

Supplement: S1 File — (ZIP) [file pone.0228610.s001.zip › 40_144/Br-11__IR_rec0766.jpg]

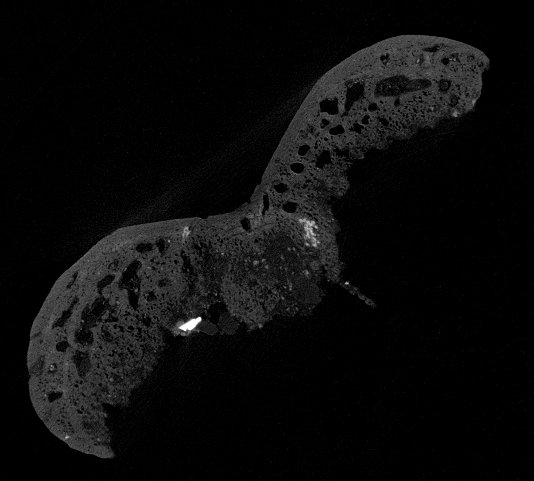

Supplement: S1 File — (ZIP) [file pone.0228610.s001.zip › 40_144/Br-11__IR_rec0770.jpg]

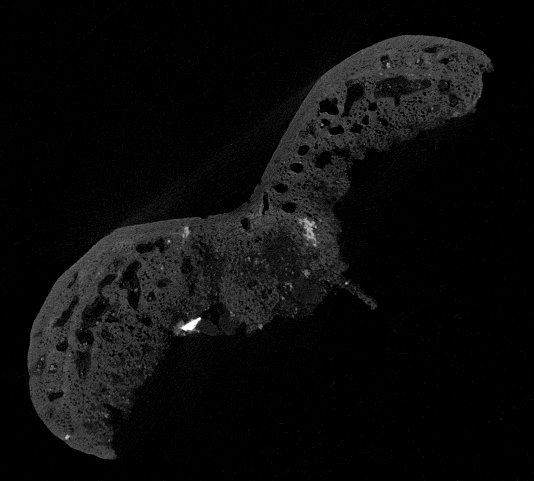

Supplement: S1 File — (ZIP) [file pone.0228610.s001.zip › 40_144/Br-11__IR_rec0774.jpg]

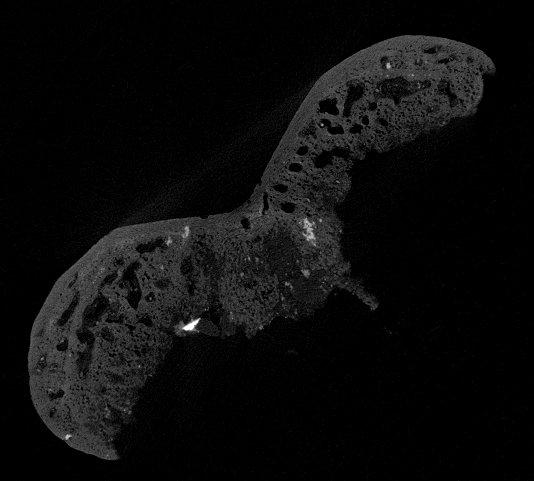

Supplement: S1 File — (ZIP) [file pone.0228610.s001.zip › 40_144/Br-11__IR_rec0778.jpg]

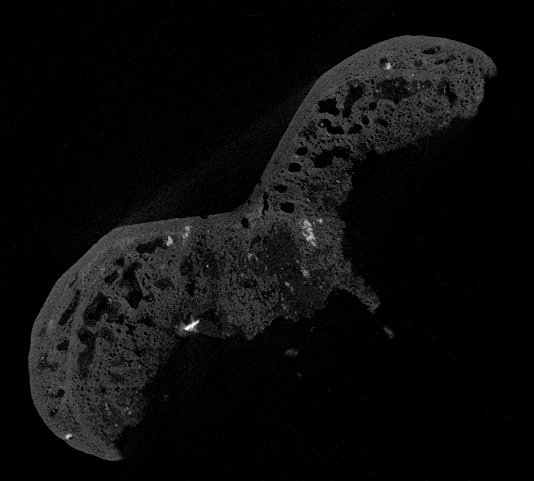

Supplement: S1 File — (ZIP) [file pone.0228610.s001.zip › 40_144/Br-11__IR_rec0782.jpg]

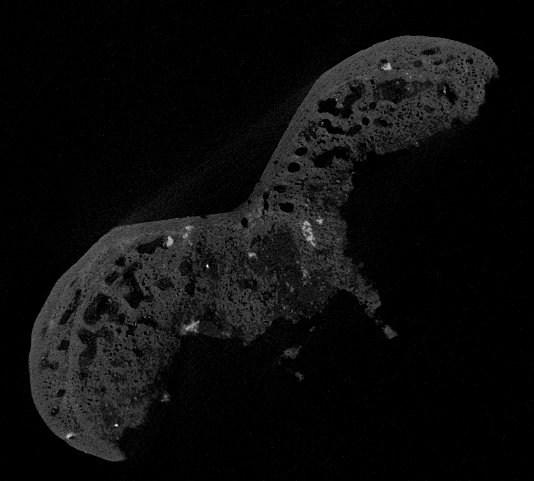

Supplement: S1 File — (ZIP) [file pone.0228610.s001.zip › 40_144/Br-11__IR_rec0786.jpg]

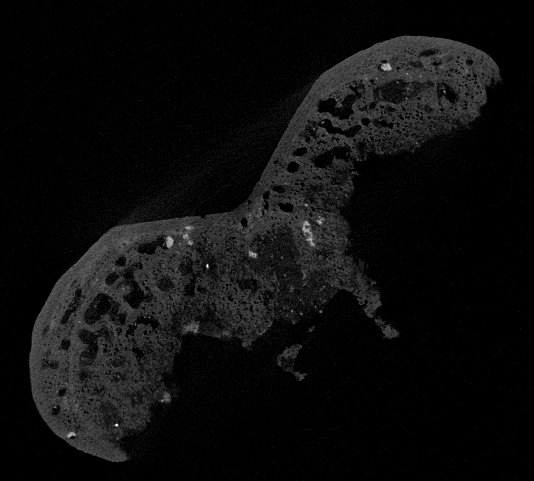

Supplement: S1 File — (ZIP) [file pone.0228610.s001.zip › 40_144/Br-11__IR_rec0790.jpg]

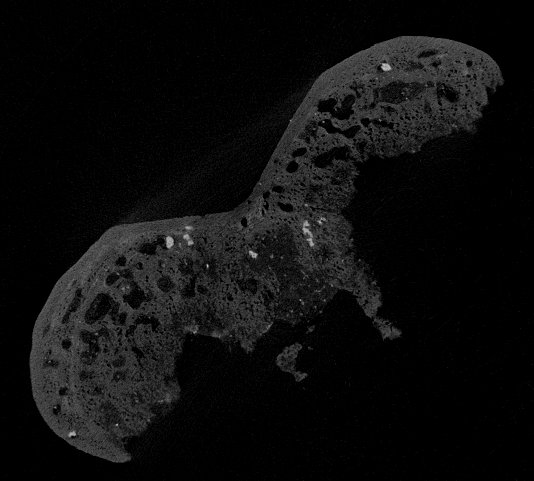

Supplement: S1 File — (ZIP) [file pone.0228610.s001.zip › 40_144/Br-11__IR_rec0794.jpg]

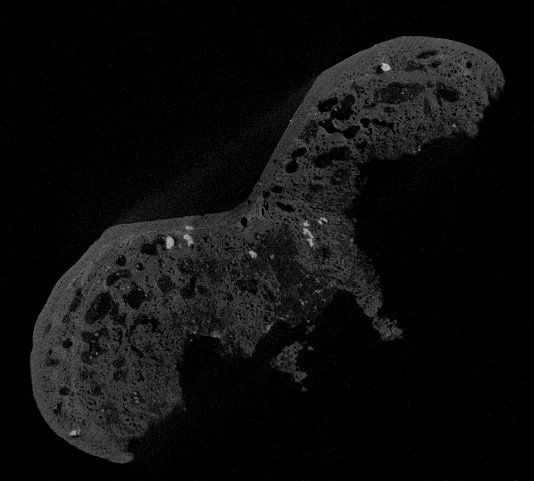

Supplement: S1 File — (ZIP) [file pone.0228610.s001.zip › 40_144/Br-11__IR_rec0798.jpg]

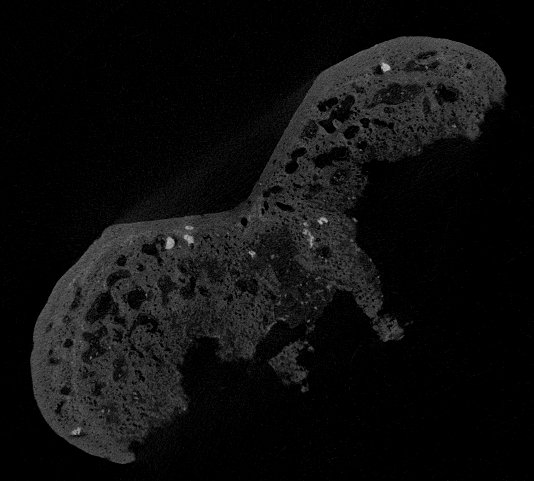

Supplement: S1 File — (ZIP) [file pone.0228610.s001.zip › 40_144/Br-11__IR_rec0802.jpg]

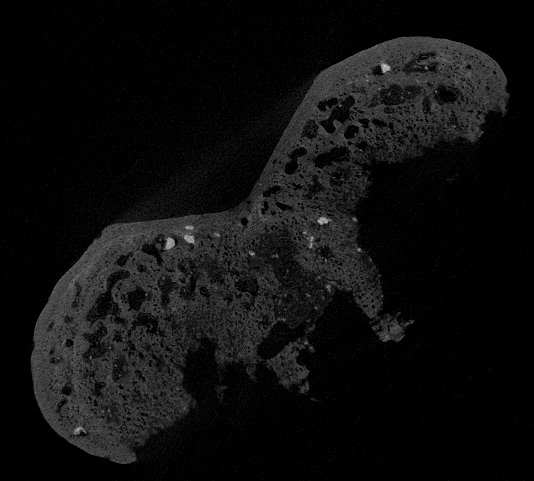

Supplement: S1 File — (ZIP) [file pone.0228610.s001.zip › 40_144/Br-11__IR_rec0806.jpg]

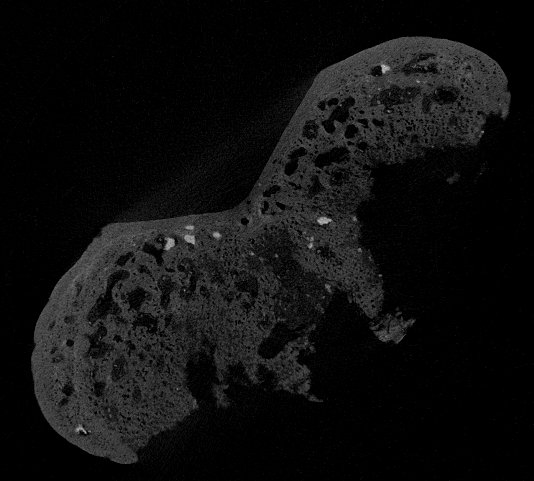

Supplement: S1 File — (ZIP) [file pone.0228610.s001.zip › 40_144/Br-11__IR_rec0810.jpg]

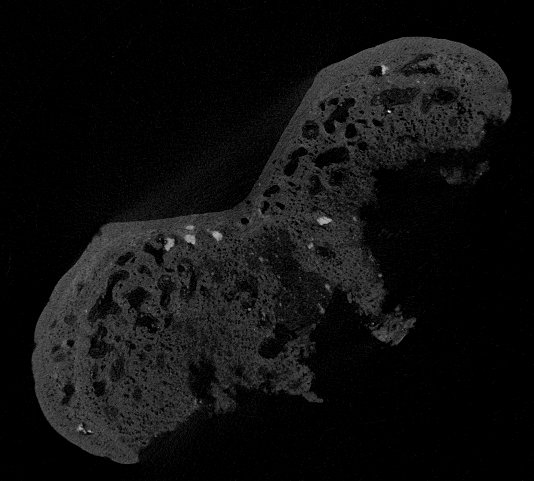

Supplement: S1 File — (ZIP) [file pone.0228610.s001.zip › 40_144/Br-11__IR_rec0814.jpg]

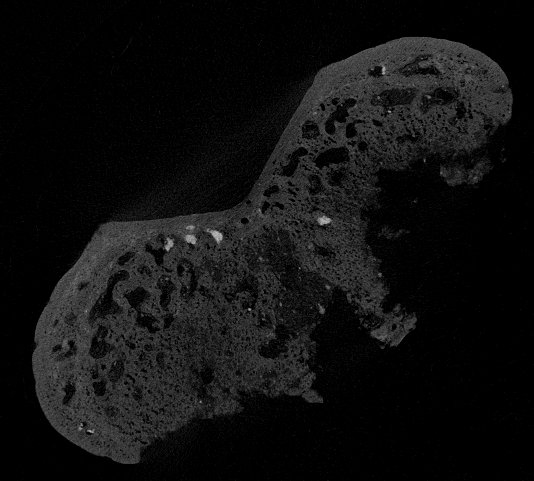

Supplement: S1 File — (ZIP) [file pone.0228610.s001.zip › 40_144/Br-11__IR_rec0818.jpg]

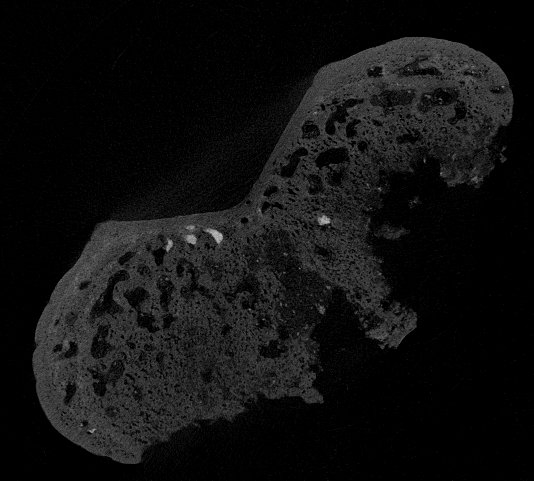

Supplement: S1 File — (ZIP) [file pone.0228610.s001.zip › 40_144/Br-11__IR_rec0822.jpg]

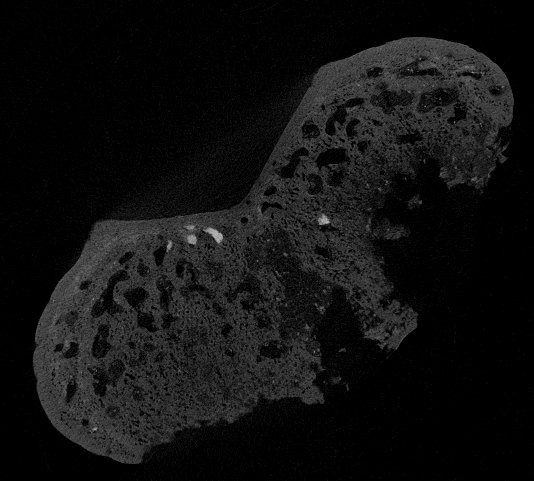

Supplement: S1 File — (ZIP) [file pone.0228610.s001.zip › 40_144/Br-11__IR_rec0826.jpg]

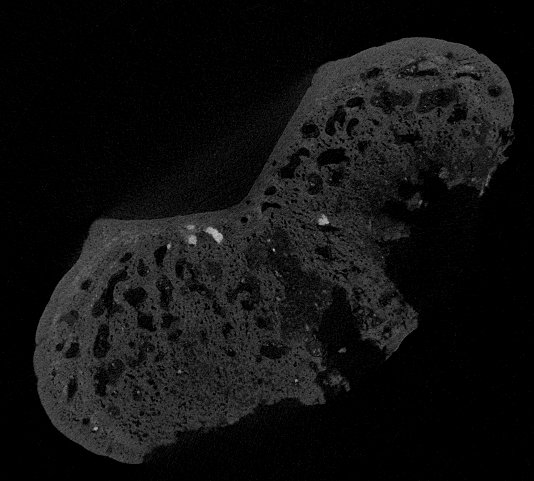

Supplement: S1 File — (ZIP) [file pone.0228610.s001.zip › 40_144/Br-11__IR_rec0830.jpg]

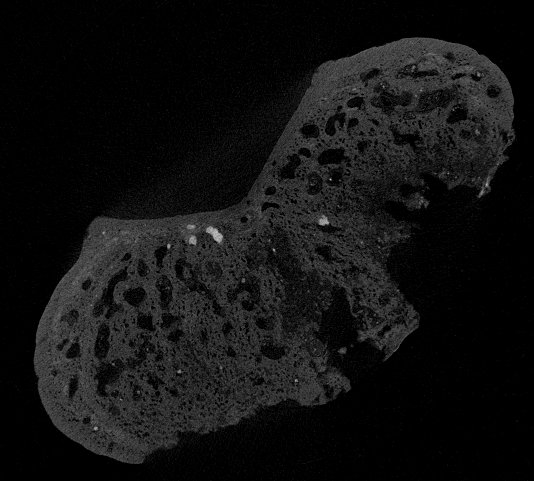

Supplement: S1 File — (ZIP) [file pone.0228610.s001.zip › 40_144/Br-11__IR_rec0834.jpg]

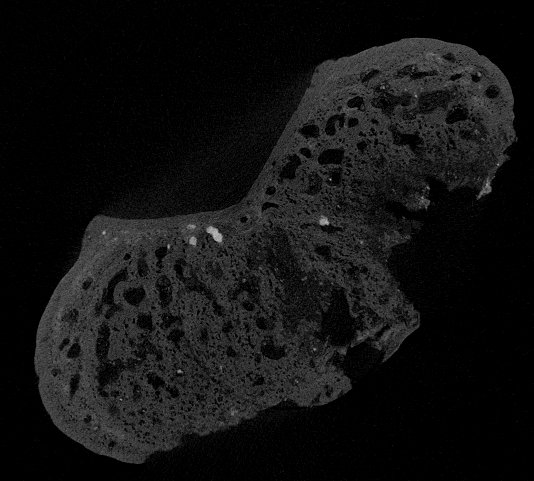

Supplement: S1 File — (ZIP) [file pone.0228610.s001.zip › 40_144/Br-11__IR_rec0838.jpg]

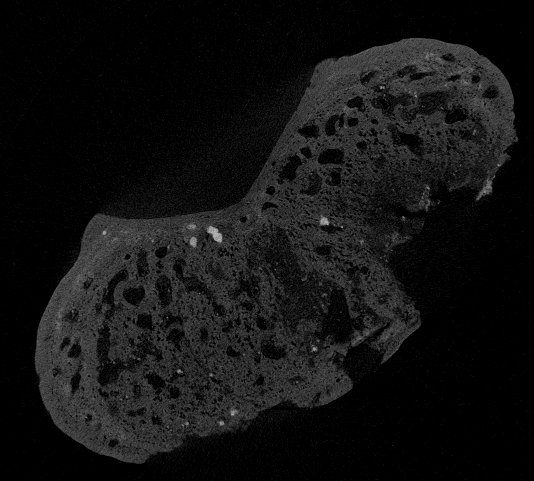

Supplement: S1 File — (ZIP) [file pone.0228610.s001.zip › 40_144/Br-11__IR_rec0842.jpg]

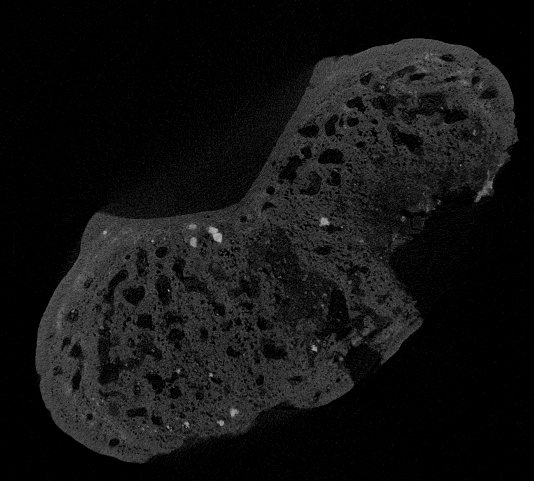

Supplement: S1 File — (ZIP) [file pone.0228610.s001.zip › 40_144/Br-11__IR_rec0846.jpg]

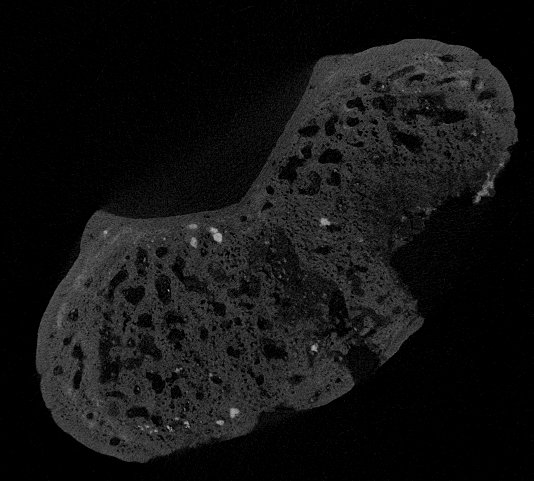

Supplement: S1 File — (ZIP) [file pone.0228610.s001.zip › 40_144/Br-11__IR_rec0850.jpg]

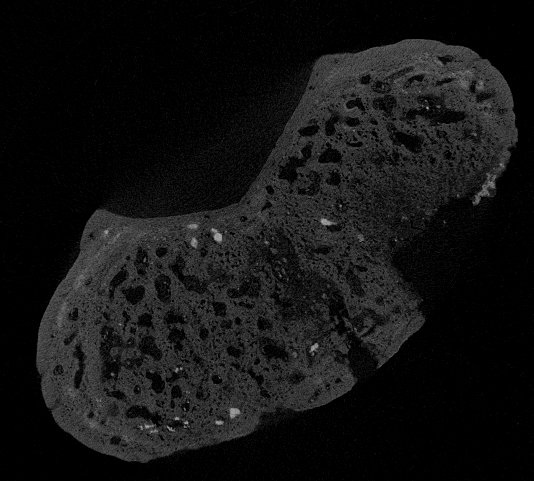

Supplement: S1 File — (ZIP) [file pone.0228610.s001.zip › 40_144/Br-11__IR_rec0854.jpg]

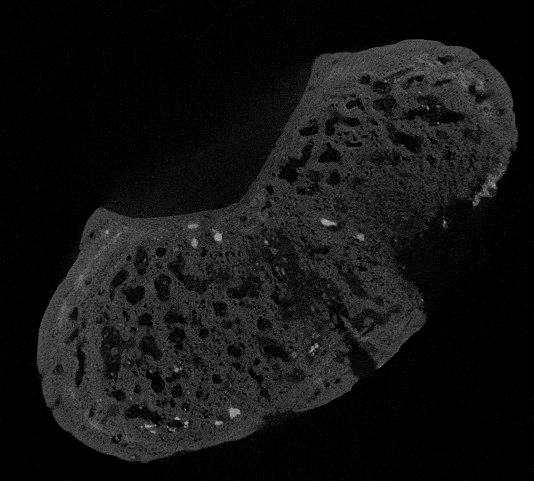

Supplement: S1 File — (ZIP) [file pone.0228610.s001.zip › 40_144/Br-11__IR_rec0858.jpg]

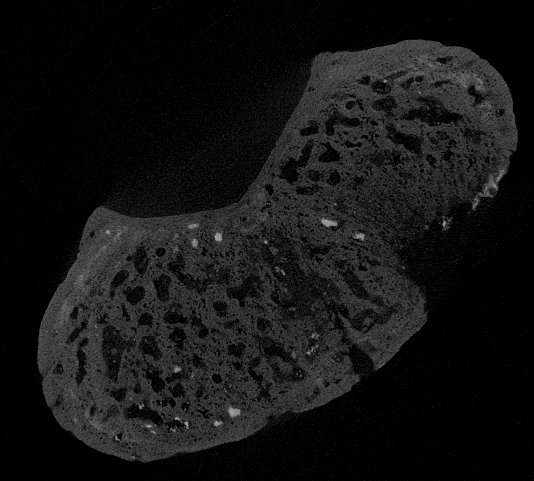

Supplement: S1 File — (ZIP) [file pone.0228610.s001.zip › 40_144/Br-11__IR_rec0862.jpg]

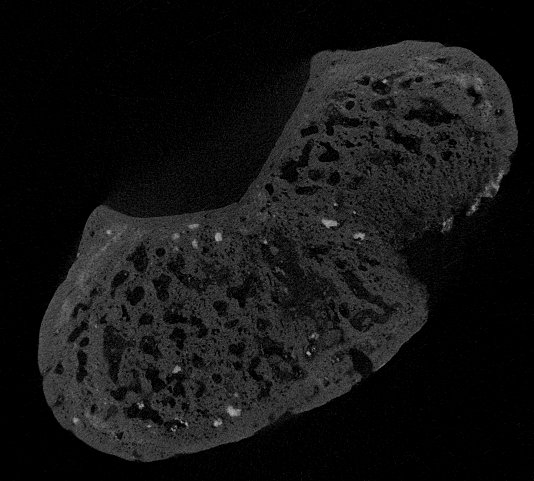

Supplement: S1 File — (ZIP) [file pone.0228610.s001.zip › 40_144/Br-11__IR_rec0866.jpg]

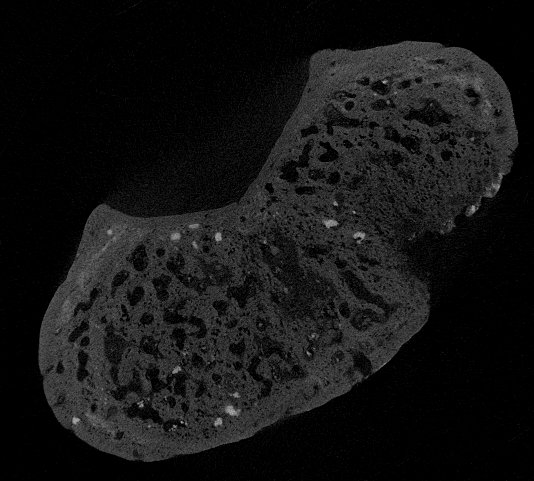

Supplement: S1 File — (ZIP) [file pone.0228610.s001.zip › 40_144/Br-11__IR_rec0870.jpg]

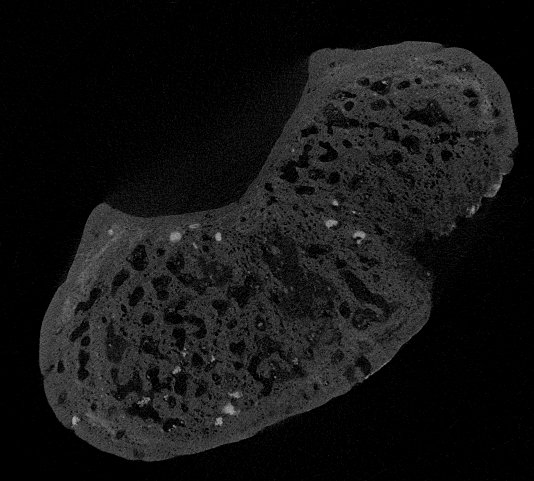

Supplement: S1 File — (ZIP) [file pone.0228610.s001.zip › 40_144/Br-11__IR_rec0874.jpg]

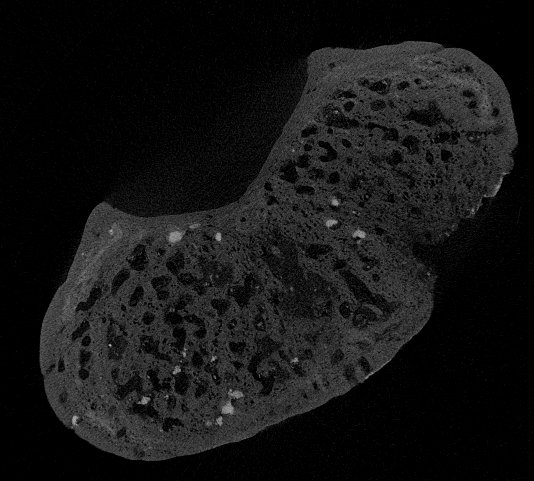

Supplement: S1 File — (ZIP) [file pone.0228610.s001.zip › 40_144/Br-11__IR_rec0878.jpg]

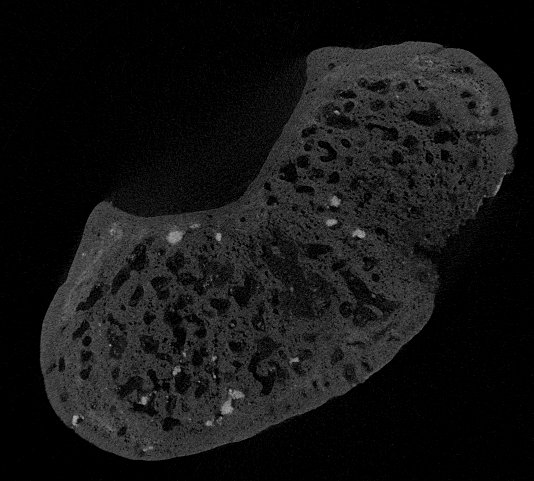

Supplement: S1 File — (ZIP) [file pone.0228610.s001.zip › 40_144/Br-11__IR_rec0882.jpg]

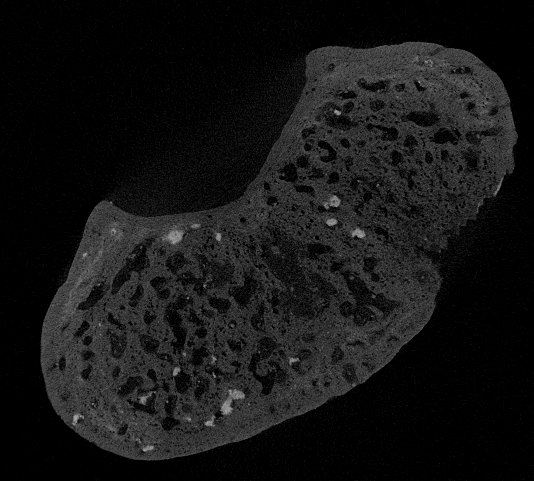

Supplement: S1 File — (ZIP) [file pone.0228610.s001.zip › 40_144/Br-11__IR_rec0886.jpg]

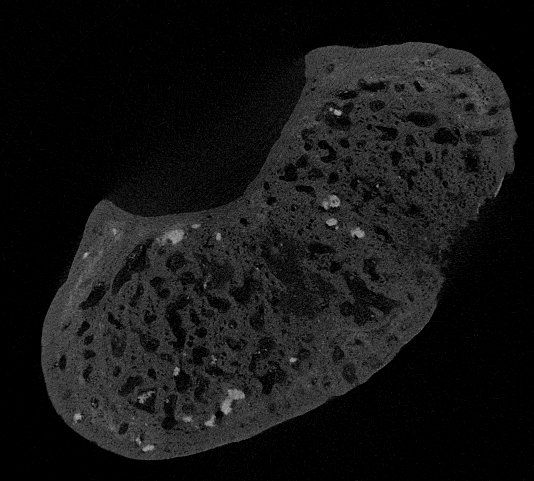

Supplement: S1 File — (ZIP) [file pone.0228610.s001.zip › 40_144/Br-11__IR_rec0890.jpg]

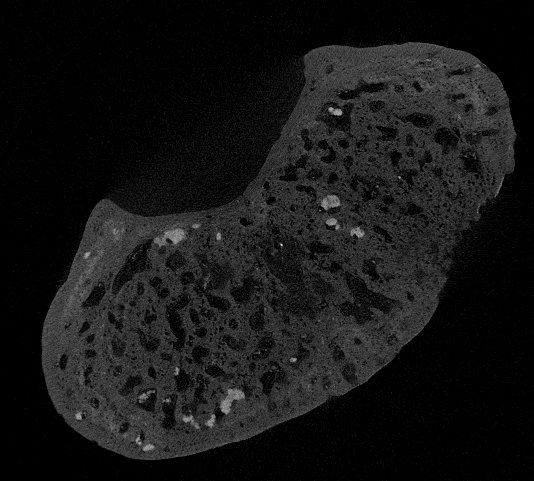

Supplement: S1 File — (ZIP) [file pone.0228610.s001.zip › 40_144/Br-11__IR_rec0894.jpg]

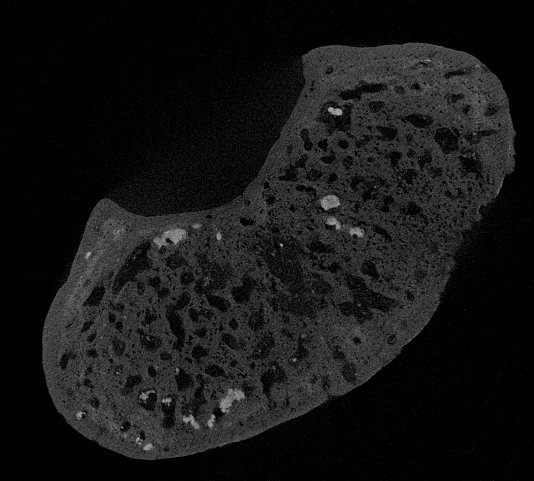

Supplement: S1 File — (ZIP) [file pone.0228610.s001.zip › 40_144/Br-11__IR_rec0898.jpg]

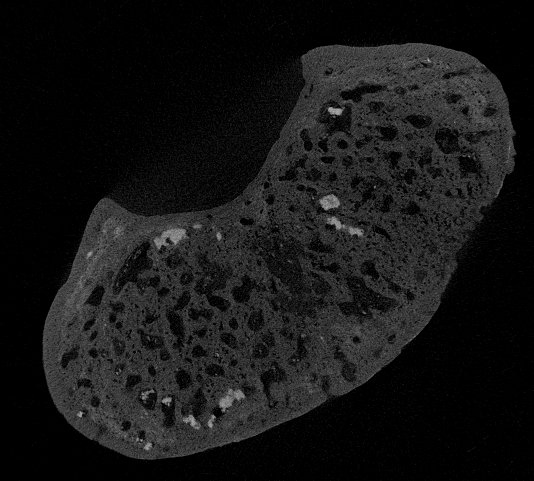

Supplement: S1 File — (ZIP) [file pone.0228610.s001.zip › 40_144/Br-11__IR_rec0902.jpg]

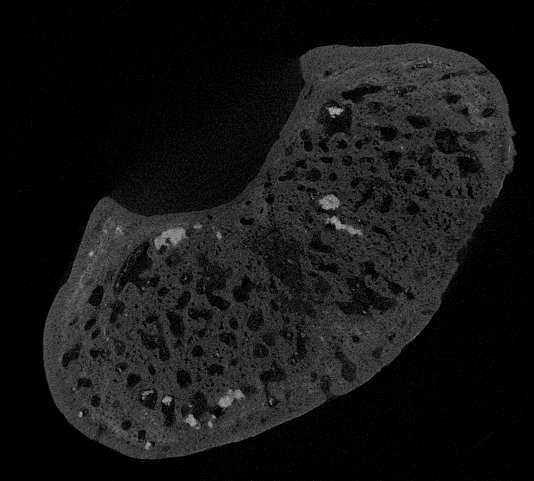

Supplement: S1 File — (ZIP) [file pone.0228610.s001.zip › 40_144/Br-11__IR_rec0906.jpg]

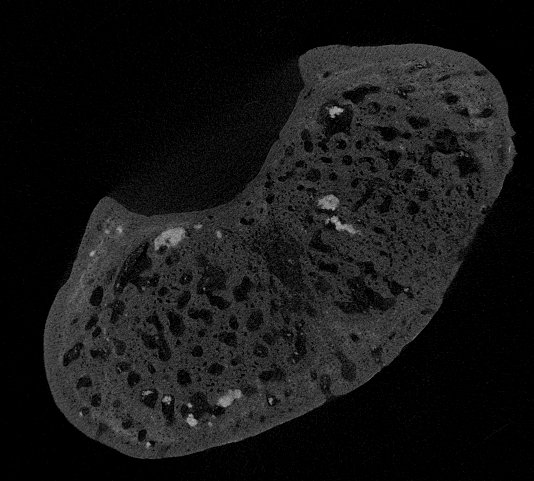

Supplement: S1 File — (ZIP) [file pone.0228610.s001.zip › 40_144/Br-11__IR_rec0910.jpg]

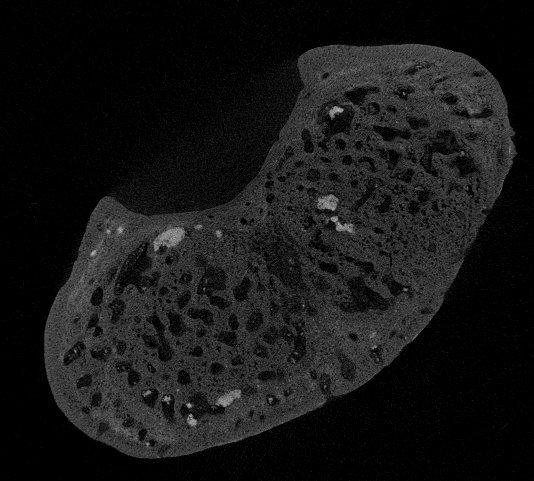

Supplement: S1 File — (ZIP) [file pone.0228610.s001.zip › 40_144/Br-11__IR_rec0914.jpg]

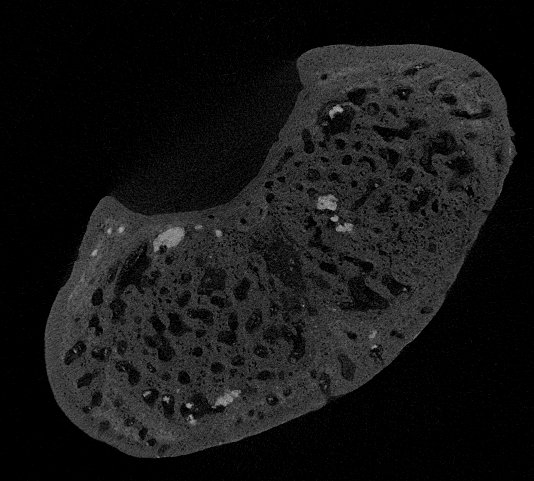

Supplement: S1 File — (ZIP) [file pone.0228610.s001.zip › 40_144/Br-11__IR_rec0918.jpg]

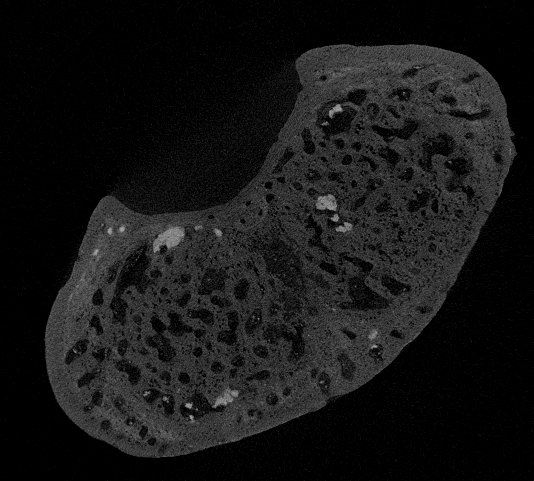

Supplement: S1 File — (ZIP) [file pone.0228610.s001.zip › 40_144/Br-11__IR_rec0922.jpg]

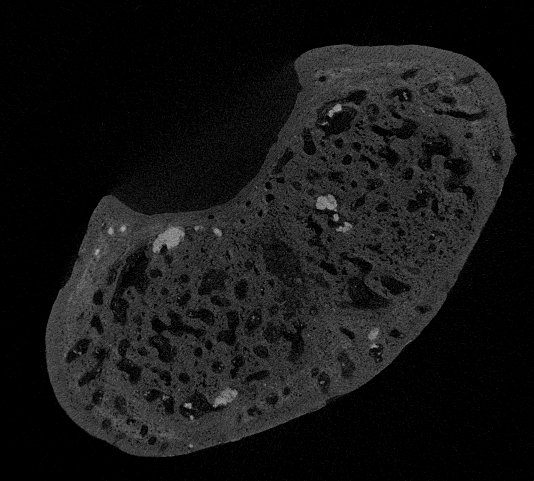

Supplement: S1 File — (ZIP) [file pone.0228610.s001.zip › 40_144/Br-11__IR_rec0926.jpg]

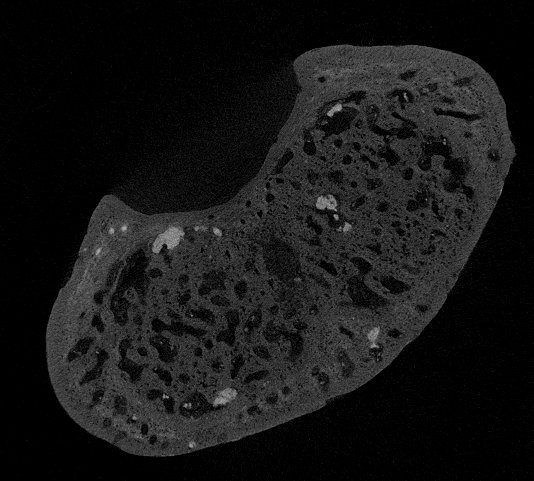

Supplement: S1 File — (ZIP) [file pone.0228610.s001.zip › 40_144/Br-11__IR_rec0930.jpg]

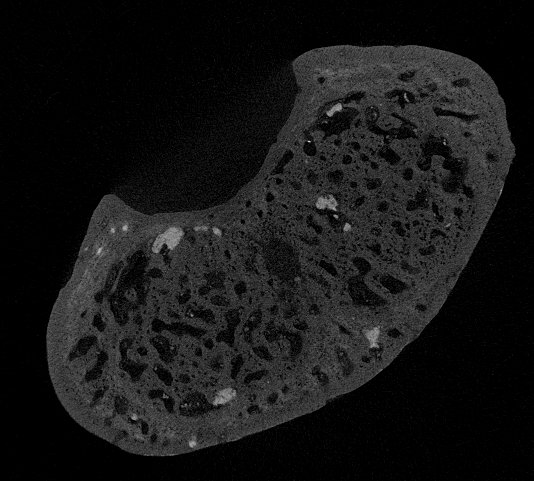

Supplement: S1 File — (ZIP) [file pone.0228610.s001.zip › 40_144/Br-11__IR_rec0934.jpg]

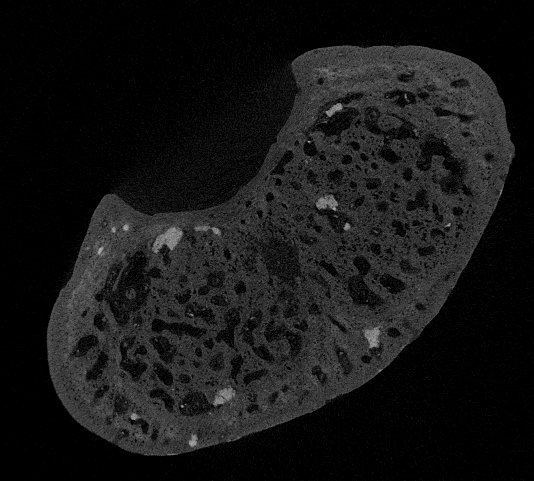

Supplement: S1 File — (ZIP) [file pone.0228610.s001.zip › 40_144/Br-11__IR_rec0938.jpg]

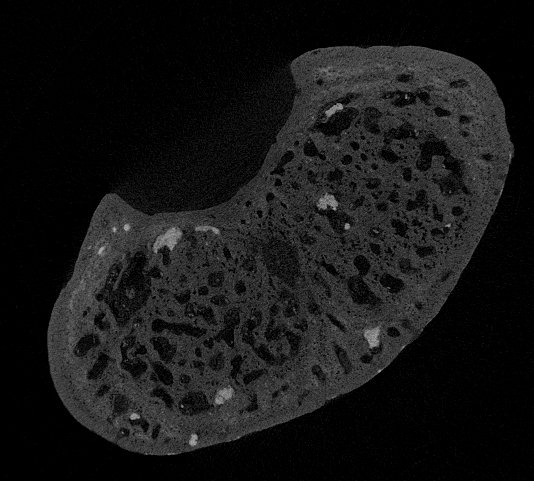

Supplement: S1 File — (ZIP) [file pone.0228610.s001.zip › 40_144/Br-11__IR_rec0942.jpg]

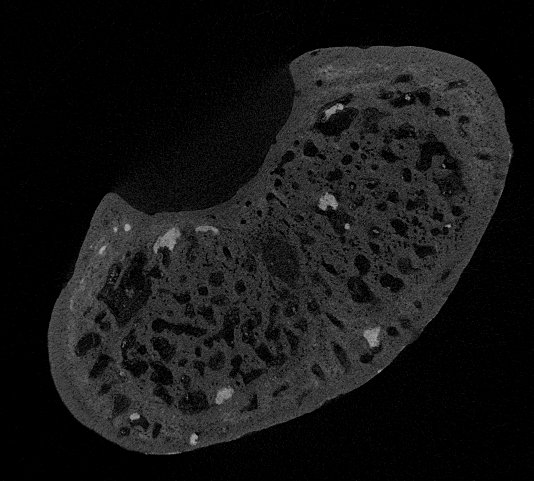

Supplement: S1 File — (ZIP) [file pone.0228610.s001.zip › 40_144/Br-11__IR_rec0946.jpg]

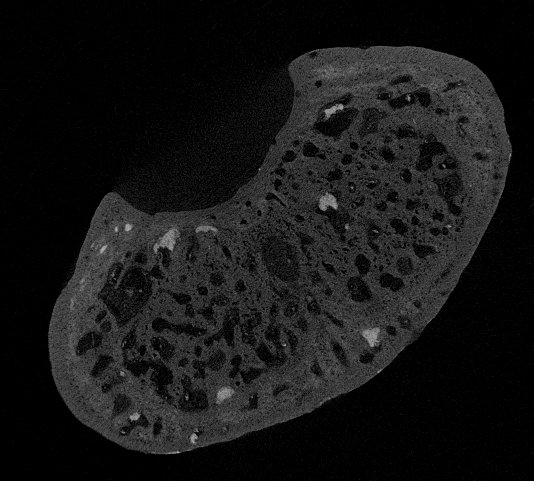

Supplement: S1 File — (ZIP) [file pone.0228610.s001.zip › 40_144/Br-11__IR_rec0950.jpg]

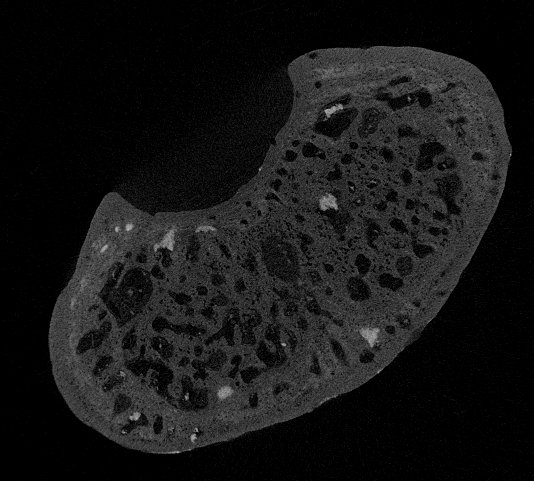

Supplement: S1 File — (ZIP) [file pone.0228610.s001.zip › 40_144/Br-11__IR_rec0954.jpg]

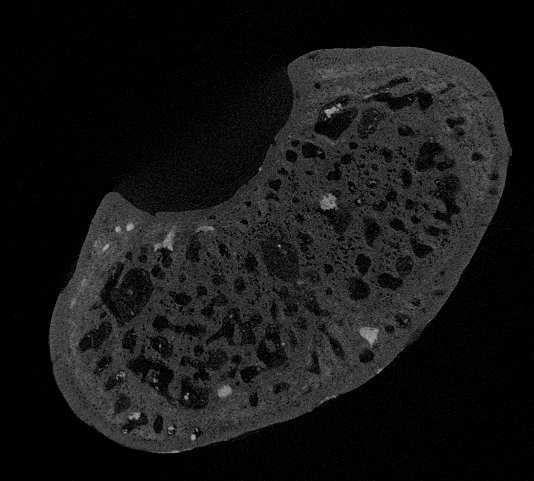

Supplement: S1 File — (ZIP) [file pone.0228610.s001.zip › 40_144/Br-11__IR_rec0958.jpg]

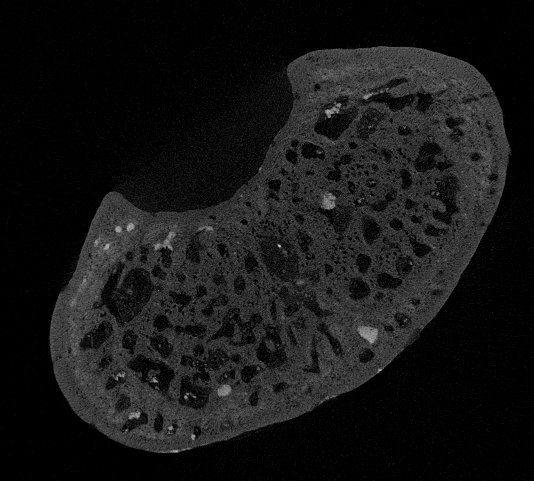

Supplement: S1 File — (ZIP) [file pone.0228610.s001.zip › 40_144/Br-11__IR_rec0962.jpg]

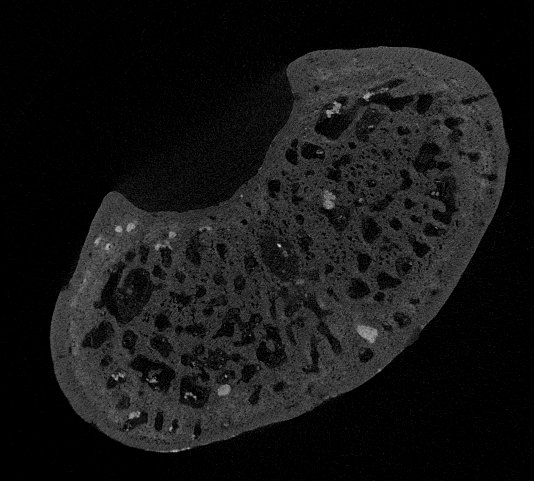

Supplement: S1 File — (ZIP) [file pone.0228610.s001.zip › 40_144/Br-11__IR_rec0966.jpg]

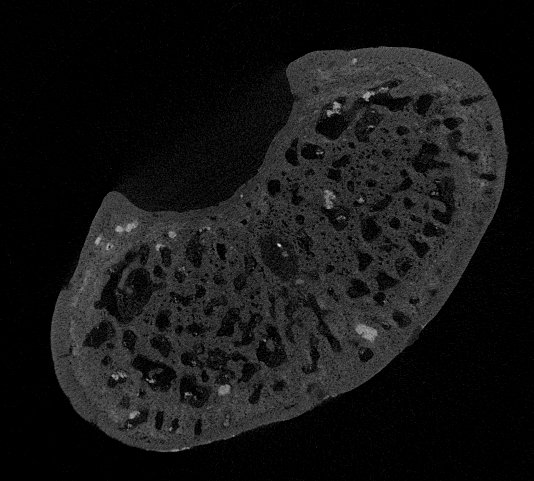

Supplement: S1 File — (ZIP) [file pone.0228610.s001.zip › 40_144/Br-11__IR_rec0970.jpg]

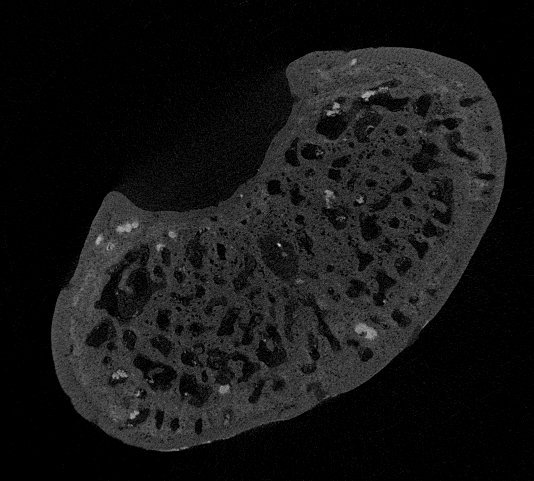

Supplement: S1 File — (ZIP) [file pone.0228610.s001.zip › 40_144/Br-11__IR_rec0974.jpg]

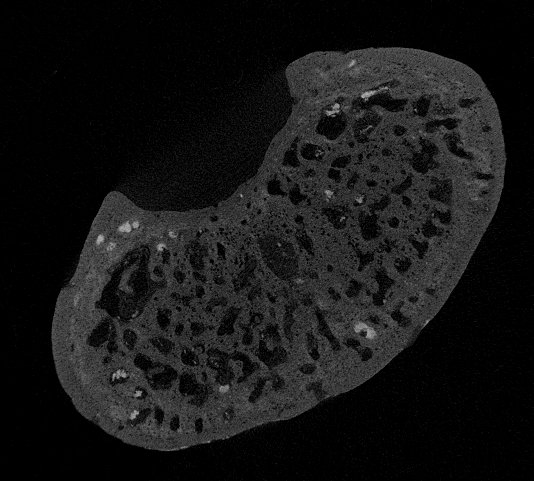

Supplement: S1 File — (ZIP) [file pone.0228610.s001.zip › 40_144/Br-11__IR_rec0978.jpg]

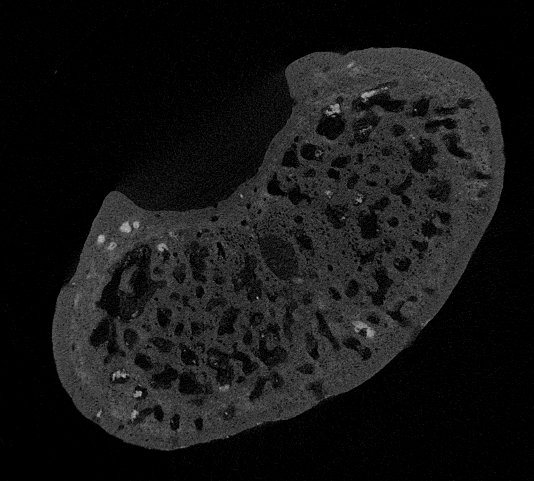

Supplement: S1 File — (ZIP) [file pone.0228610.s001.zip › 40_144/Br-11__IR_rec0982.jpg]

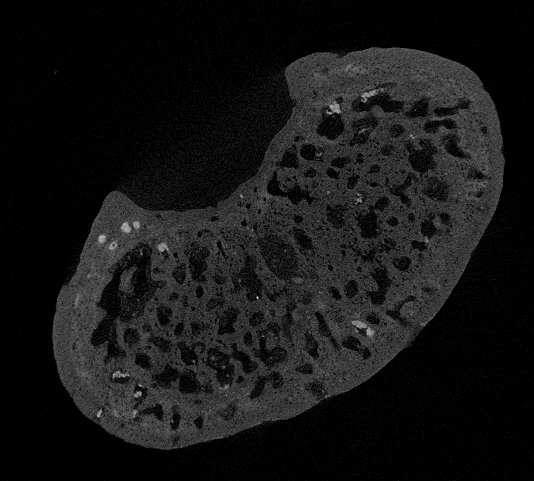

Supplement: S1 File — (ZIP) [file pone.0228610.s001.zip › 40_144/Br-11__IR_rec0986.jpg]

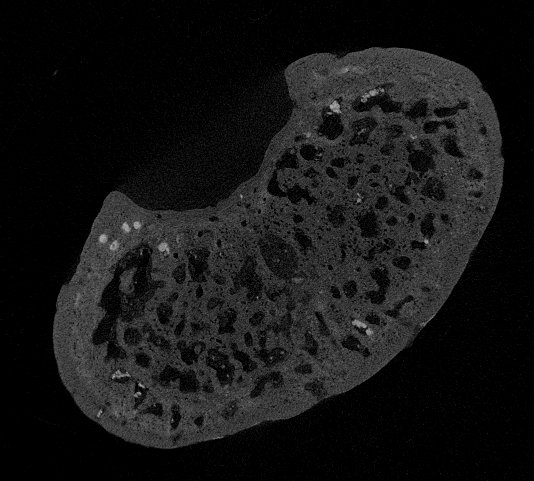

Supplement: S1 File — (ZIP) [file pone.0228610.s001.zip › 40_144/Br-11__IR_rec0990.jpg]

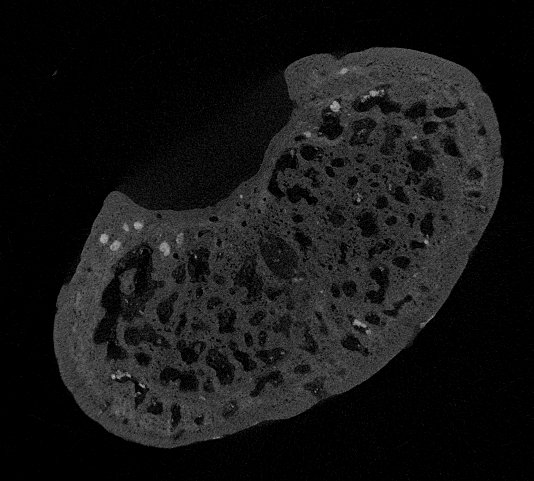

Supplement: S1 File — (ZIP) [file pone.0228610.s001.zip › 40_144/Br-11__IR_rec0994.jpg]

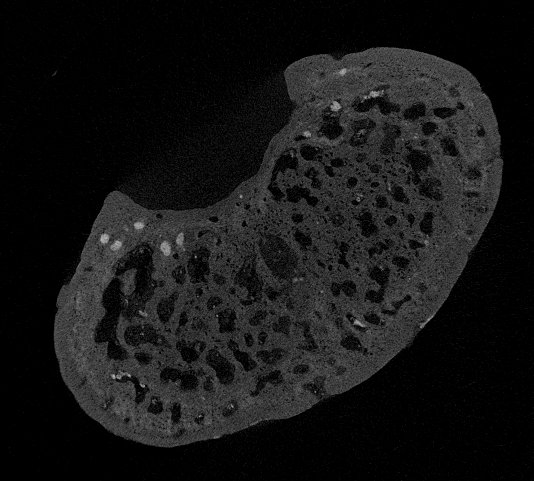

Supplement: S1 File — (ZIP) [file pone.0228610.s001.zip › 40_144/Br-11__IR_rec0998.jpg]

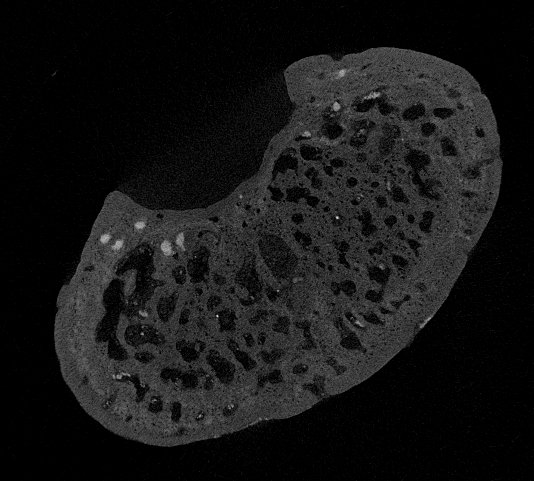

Supplement: S1 File — (ZIP) [file pone.0228610.s001.zip › 40_144/Br-11__IR_rec1002.jpg]

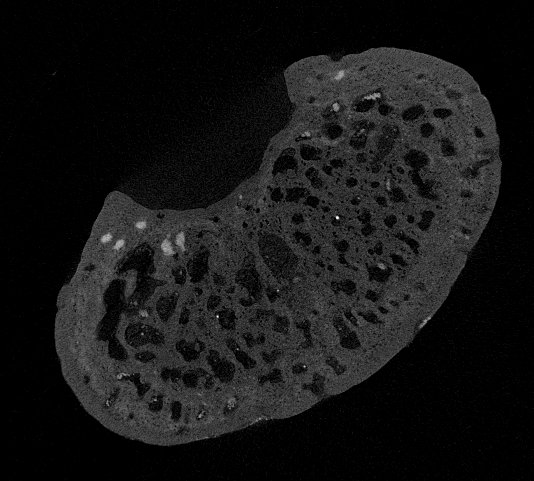

Supplement: S1 File — (ZIP) [file pone.0228610.s001.zip › 40_144/Br-11__IR_rec1006.jpg]

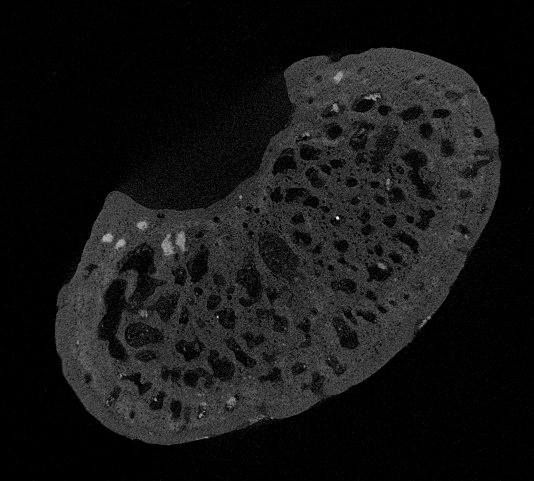

Supplement: S1 File — (ZIP) [file pone.0228610.s001.zip › 40_144/Br-11__IR_rec1010.jpg]

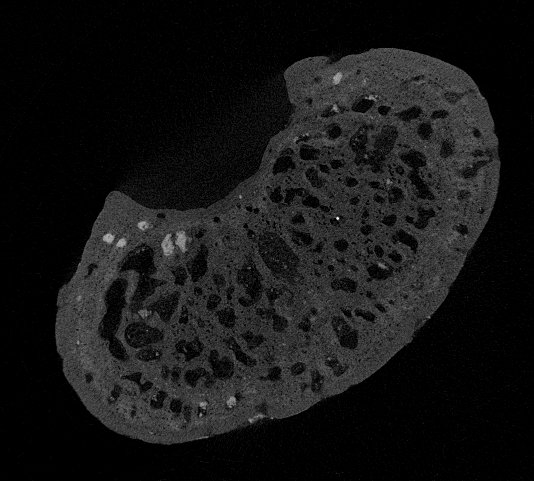

Supplement: S1 File — (ZIP) [file pone.0228610.s001.zip › 40_144/Br-11__IR_rec1014.jpg]

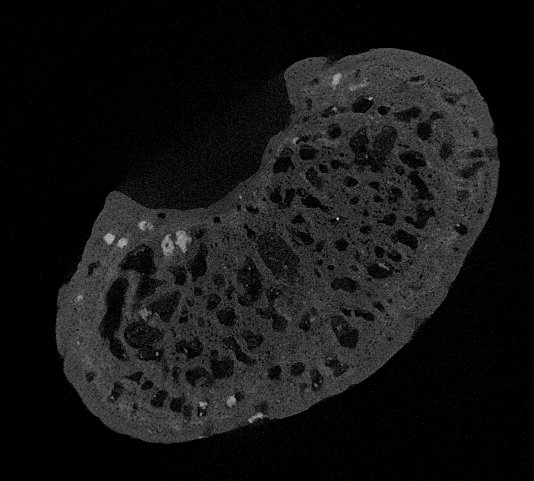

Supplement: S1 File — (ZIP) [file pone.0228610.s001.zip › 40_144/Br-11__IR_rec1018.jpg]

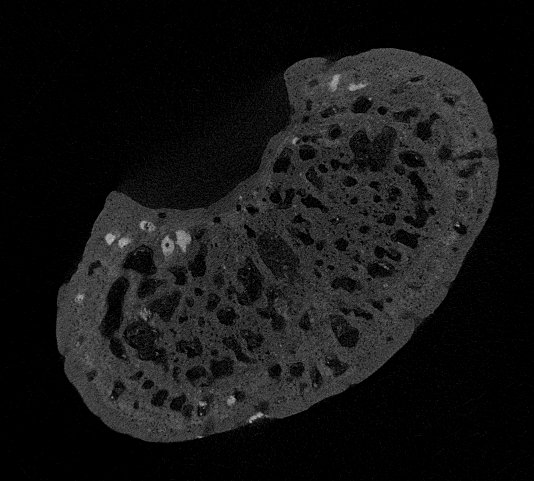

Supplement: S1 File — (ZIP) [file pone.0228610.s001.zip › 40_144/Br-11__IR_rec1022.jpg]

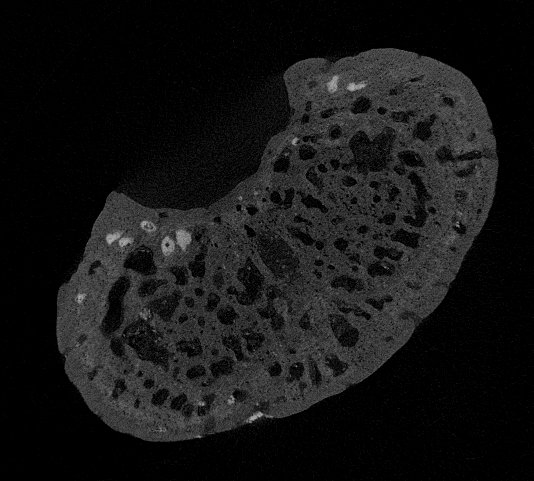

Supplement: S1 File — (ZIP) [file pone.0228610.s001.zip › 40_144/Br-11__IR_rec1026.jpg]

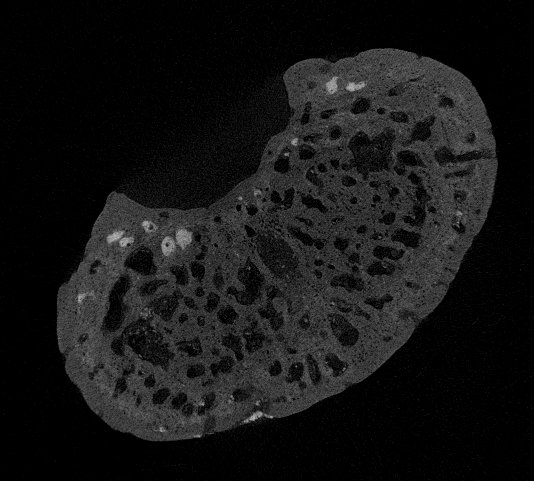

Supplement: S1 File — (ZIP) [file pone.0228610.s001.zip › 40_144/Br-11__IR_rec1030.jpg]

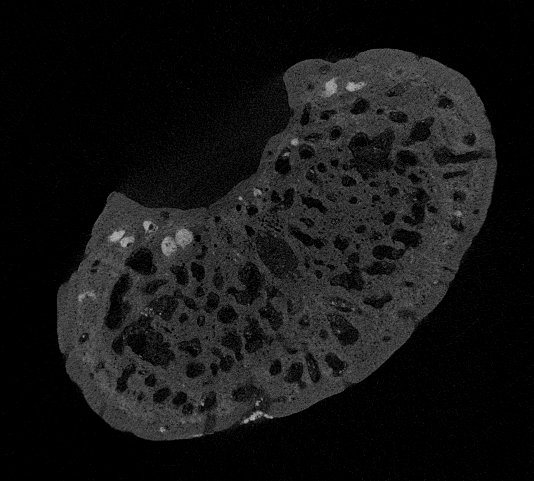

Supplement: S1 File — (ZIP) [file pone.0228610.s001.zip › 40_144/Br-11__IR_rec1034.jpg]

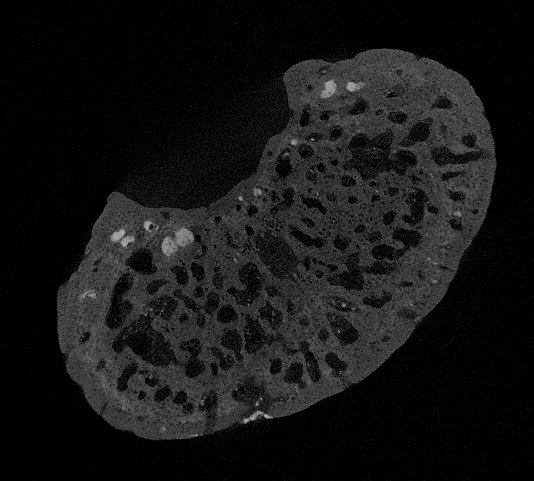

Supplement: S1 File — (ZIP) [file pone.0228610.s001.zip › 40_144/Br-11__IR_rec1038.jpg]

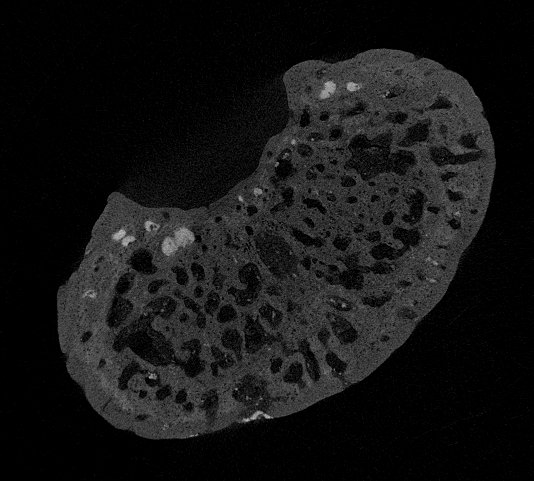

Supplement: S1 File — (ZIP) [file pone.0228610.s001.zip › 40_144/Br-11__IR_rec1042.jpg]

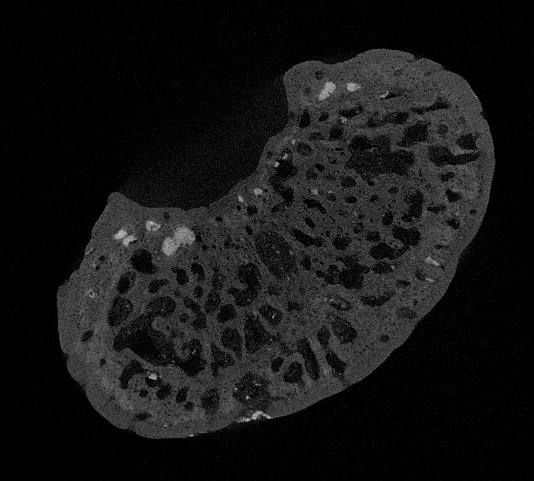

Supplement: S1 File — (ZIP) [file pone.0228610.s001.zip › 40_144/Br-11__IR_rec1046.jpg]

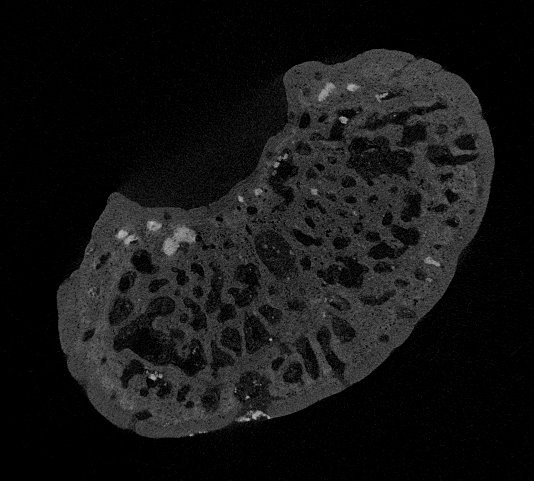

Supplement: S1 File — (ZIP) [file pone.0228610.s001.zip › 40_144/Br-11__IR_rec1050.jpg]

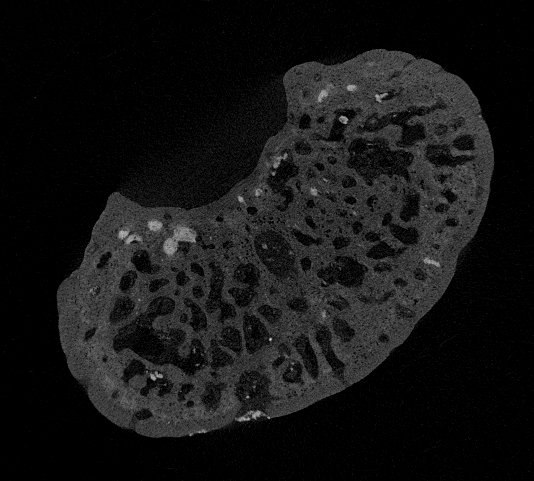

Supplement: S1 File — (ZIP) [file pone.0228610.s001.zip › 40_144/Br-11__IR_rec1054.jpg]

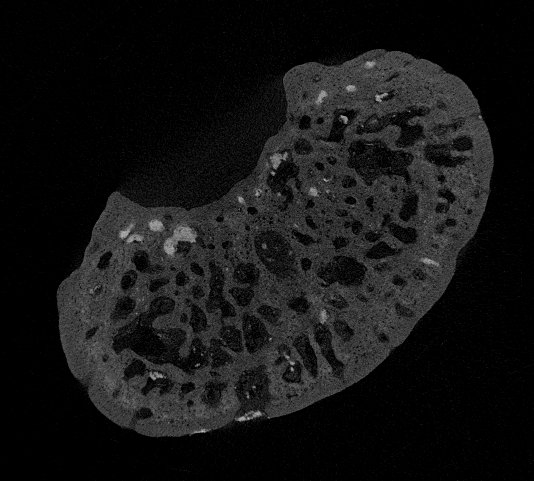

Supplement: S1 File — (ZIP) [file pone.0228610.s001.zip › 40_144/Br-11__IR_rec1058.jpg]

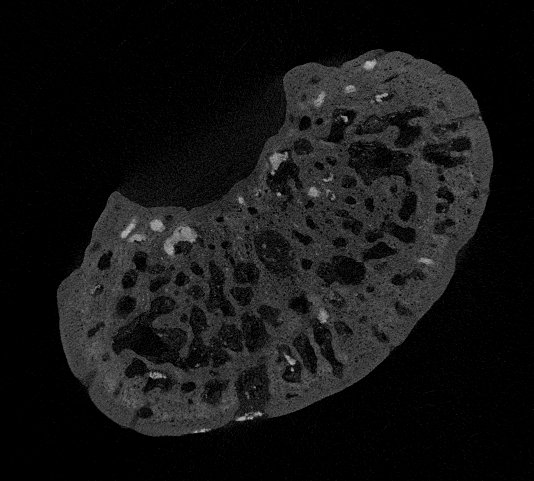

Supplement: S1 File — (ZIP) [file pone.0228610.s001.zip › 40_144/Br-11__IR_rec1062.jpg]

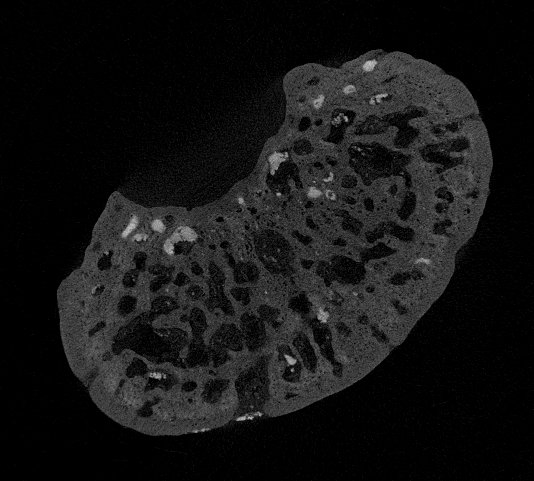

Supplement: S1 File — (ZIP) [file pone.0228610.s001.zip › 40_144/Br-11__IR_rec1066.jpg]

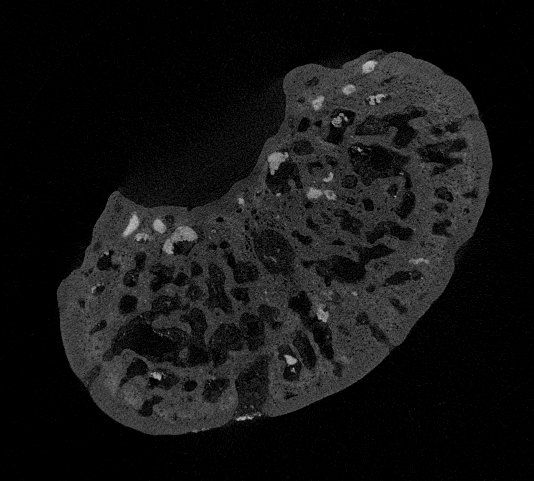

Supplement: S1 File — (ZIP) [file pone.0228610.s001.zip › 40_144/Br-11__IR_rec1070.jpg]

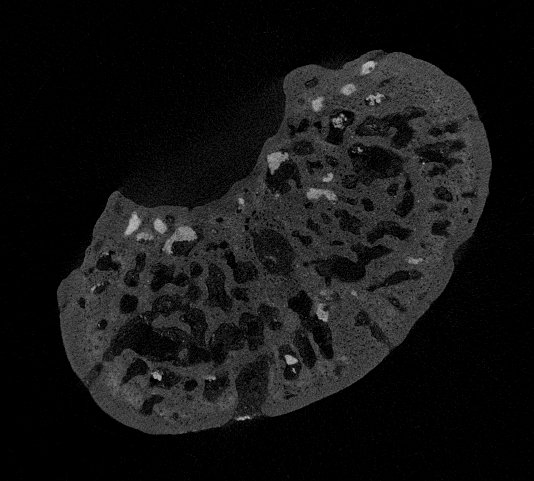

Supplement: S1 File — (ZIP) [file pone.0228610.s001.zip › 40_144/Br-11__IR_rec1074.jpg]

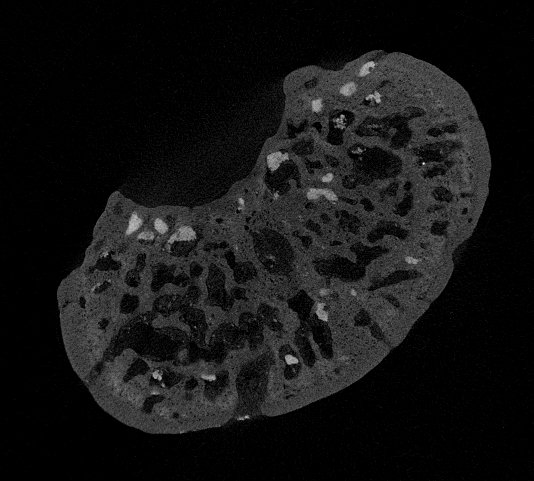

Supplement: S1 File — (ZIP) [file pone.0228610.s001.zip › 40_144/Br-11__IR_rec1078.jpg]

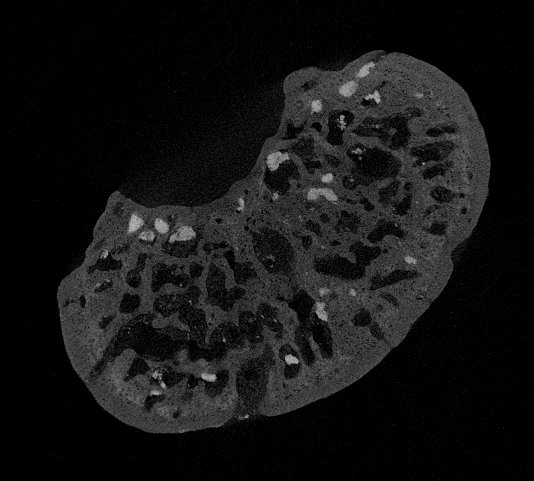

Supplement: S1 File — (ZIP) [file pone.0228610.s001.zip › 40_144/Br-11__IR_rec1082.jpg]

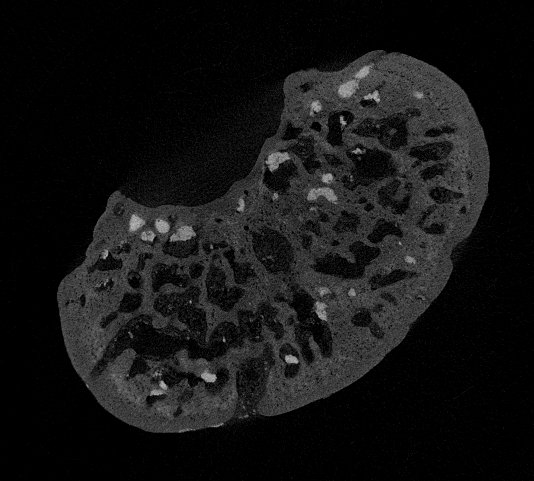

Supplement: S1 File — (ZIP) [file pone.0228610.s001.zip › 40_144/Br-11__IR_rec1086.jpg]

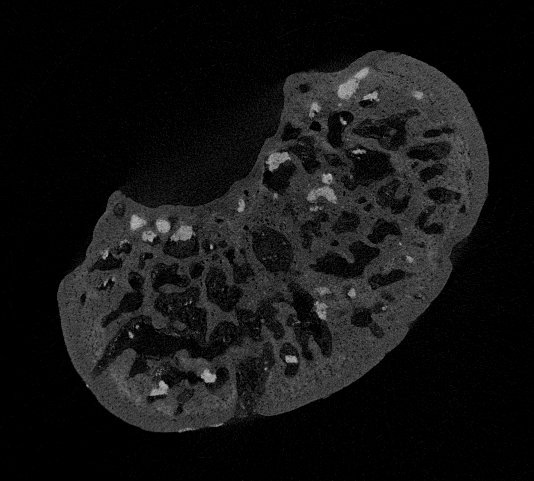

Supplement: S1 File — (ZIP) [file pone.0228610.s001.zip › 40_144/Br-11__IR_rec1090.jpg]

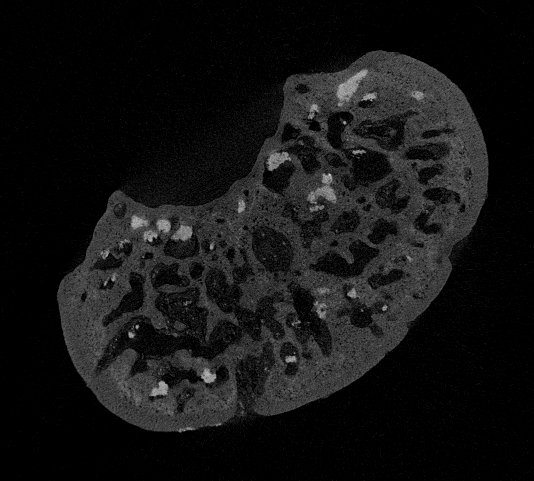

Supplement: S1 File — (ZIP) [file pone.0228610.s001.zip › 40_144/Br-11__IR_rec1094.jpg]

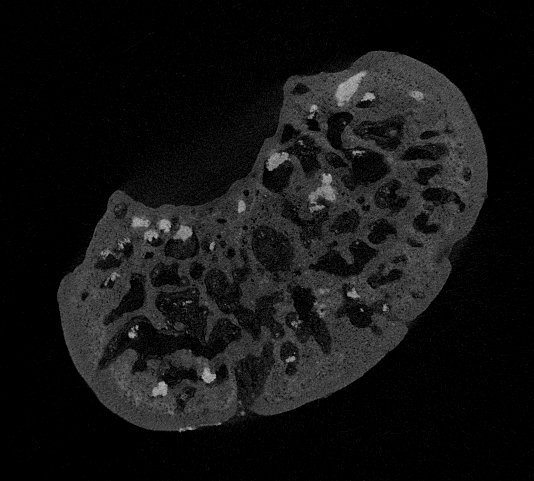

Supplement: S1 File — (ZIP) [file pone.0228610.s001.zip › 40_144/Br-11__IR_rec1098.jpg]

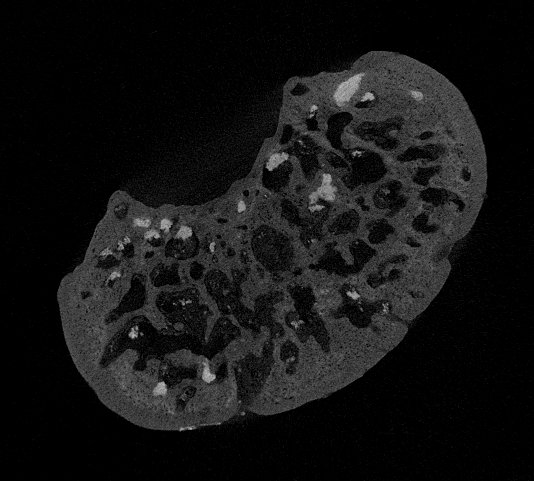

Supplement: S1 File — (ZIP) [file pone.0228610.s001.zip › 40_144/Br-11__IR_rec1102.jpg]

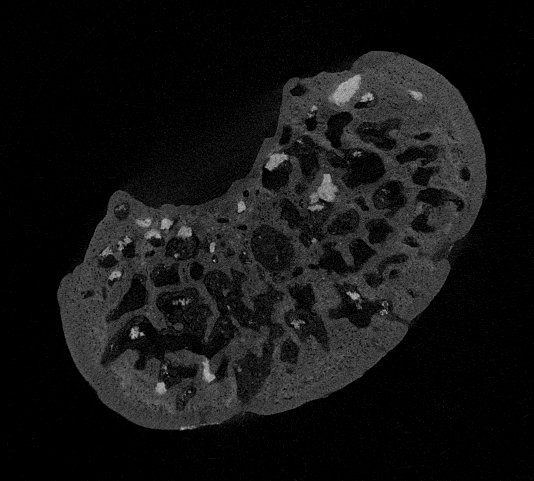

Supplement: S1 File — (ZIP) [file pone.0228610.s001.zip › 40_144/Br-11__IR_rec1106.jpg]

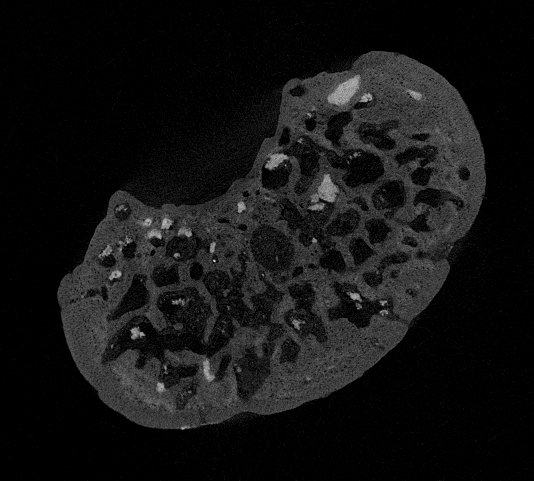

Supplement: S1 File — (ZIP) [file pone.0228610.s001.zip › 40_144/Br-11__IR_rec1110.jpg]

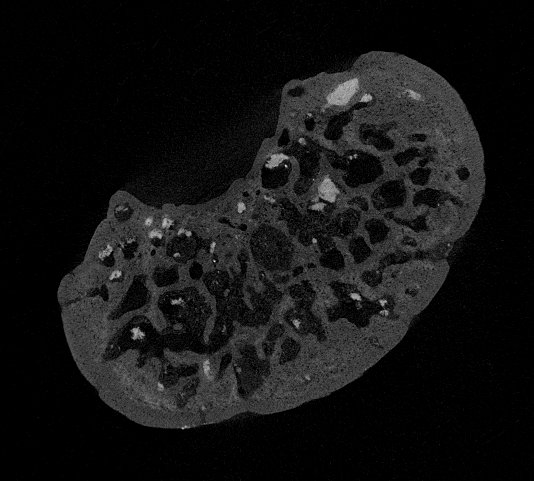

Supplement: S1 File — (ZIP) [file pone.0228610.s001.zip › 40_144/Br-11__IR_rec1114.jpg]

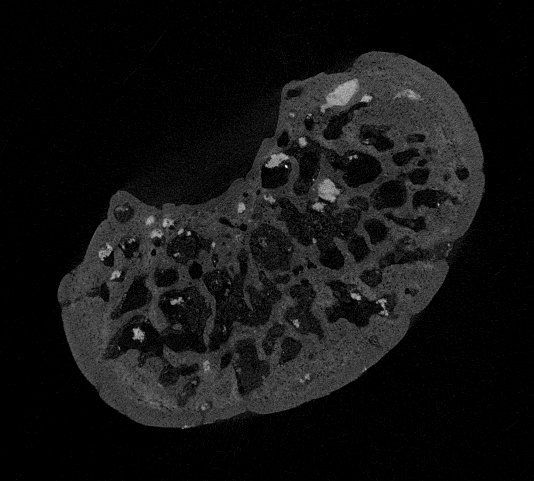

Supplement: S1 File — (ZIP) [file pone.0228610.s001.zip › 40_144/Br-11__IR_rec1118.jpg]

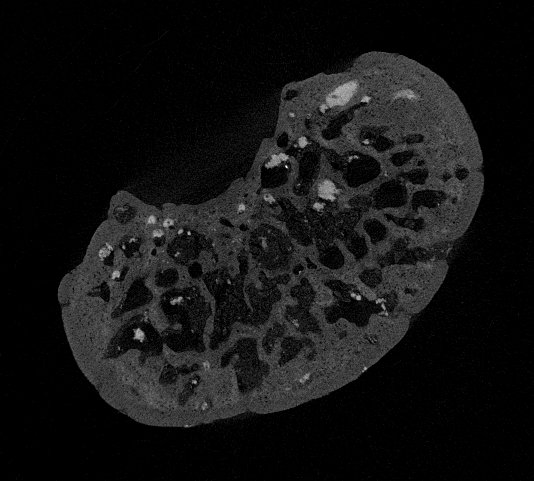

Supplement: S1 File — (ZIP) [file pone.0228610.s001.zip › 40_144/Br-11__IR_rec1122.jpg]

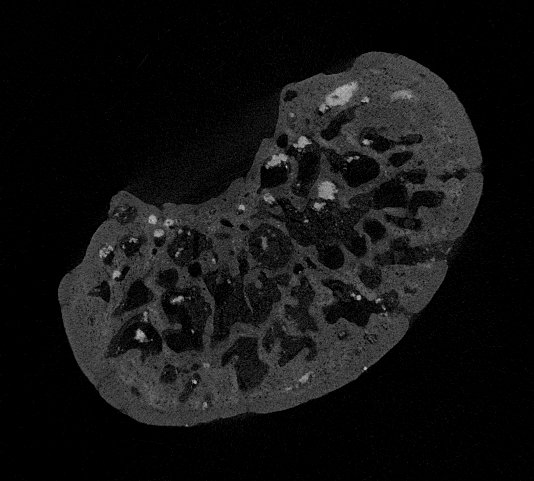

Supplement: S1 File — (ZIP) [file pone.0228610.s001.zip › 40_144/Br-11__IR_rec1126.jpg]

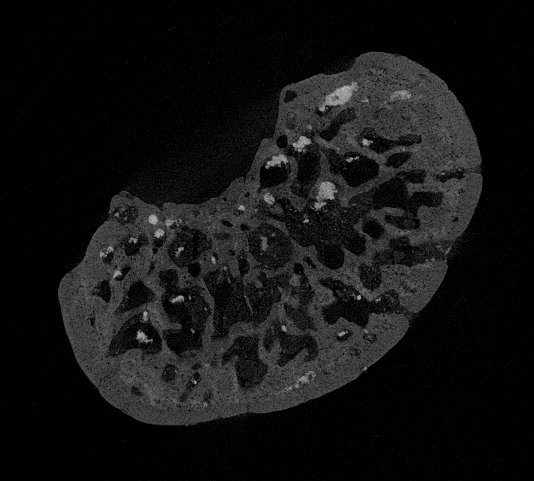

Supplement: S1 File — (ZIP) [file pone.0228610.s001.zip › 40_144/Br-11__IR_rec1130.jpg]

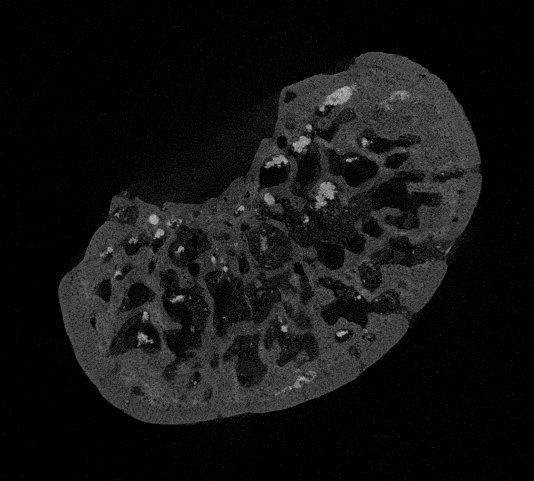

Supplement: S1 File — (ZIP) [file pone.0228610.s001.zip › 40_144/Br-11__IR_rec1134.jpg]

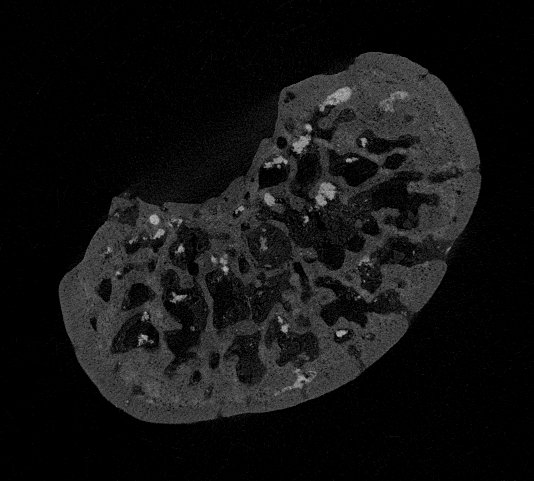

Supplement: S1 File — (ZIP) [file pone.0228610.s001.zip › 40_144/Br-11__IR_rec1138.jpg]

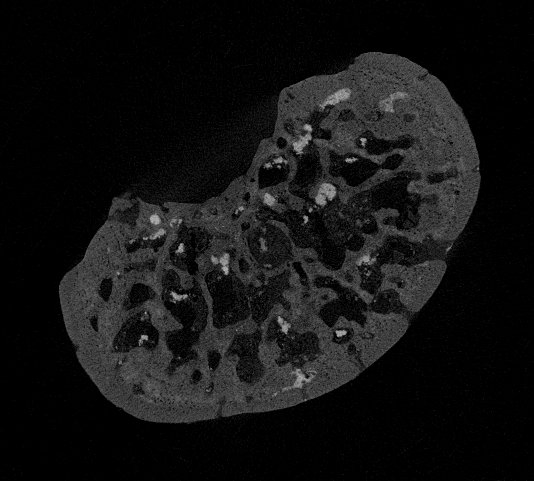

Supplement: S1 File — (ZIP) [file pone.0228610.s001.zip › 40_144/Br-11__IR_rec1142.jpg]
