# Supplementary figures and images for: A new small-sized stem salamander from the Middle Jurassic of Western Siberia, Russia (part 1 of 10)
Source: PLoS One. 2020 Feb 19;15(2):e0228610. doi: 10.1371/journal.pone.0228610 (PMC7029856; doi:10.1371/journal.pone.0228610)

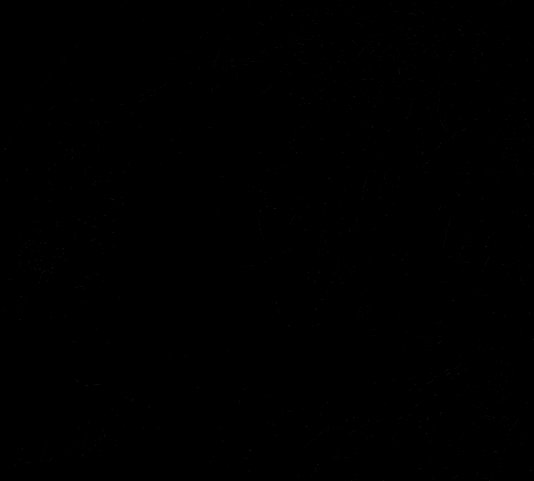

Supplement: S1 File — (ZIP) [file pone.0228610.s001.zip › 40_144/Br-11__IR_rec0346.jpg]

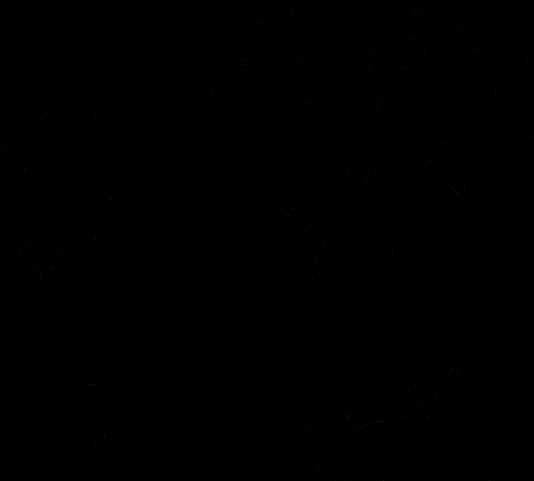

Supplement: S1 File — (ZIP) [file pone.0228610.s001.zip › 40_144/Br-11__IR_rec0350.jpg]

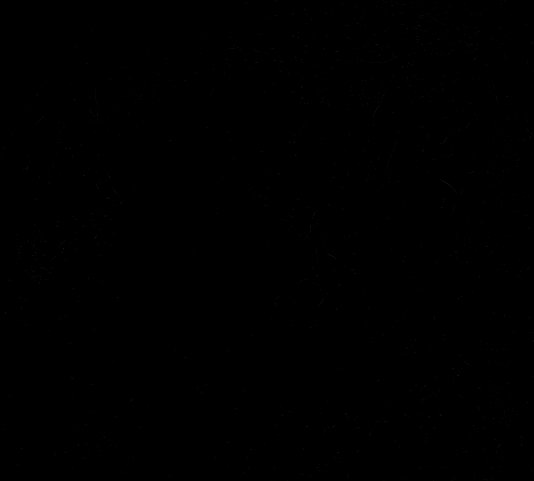

Supplement: S1 File — (ZIP) [file pone.0228610.s001.zip › 40_144/Br-11__IR_rec0354.jpg]

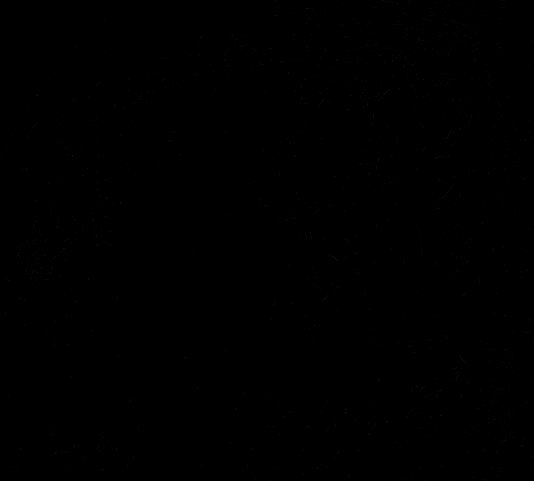

Supplement: S1 File — (ZIP) [file pone.0228610.s001.zip › 40_144/Br-11__IR_rec0358.jpg]

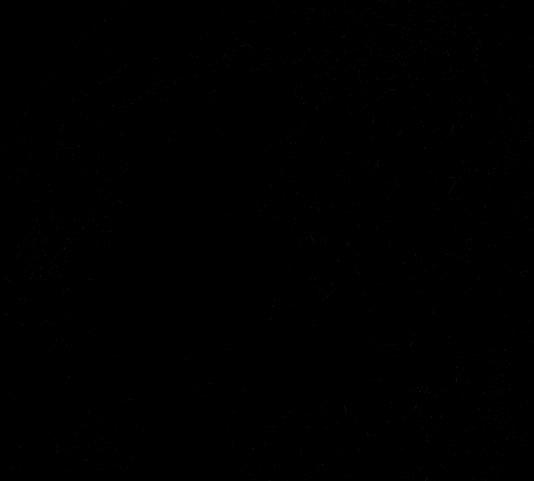

Supplement: S1 File — (ZIP) [file pone.0228610.s001.zip › 40_144/Br-11__IR_rec0362.jpg]

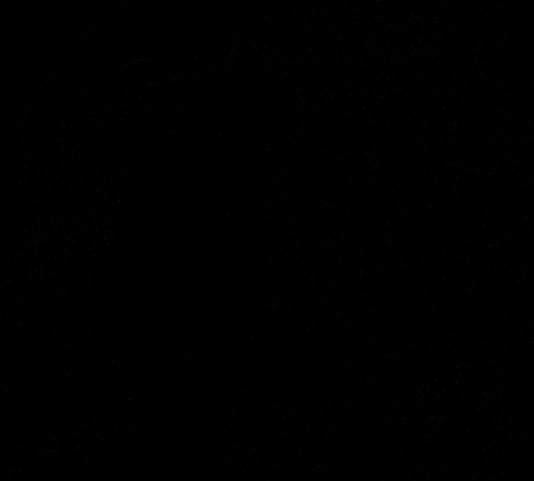

Supplement: S1 File — (ZIP) [file pone.0228610.s001.zip › 40_144/Br-11__IR_rec0366.jpg]

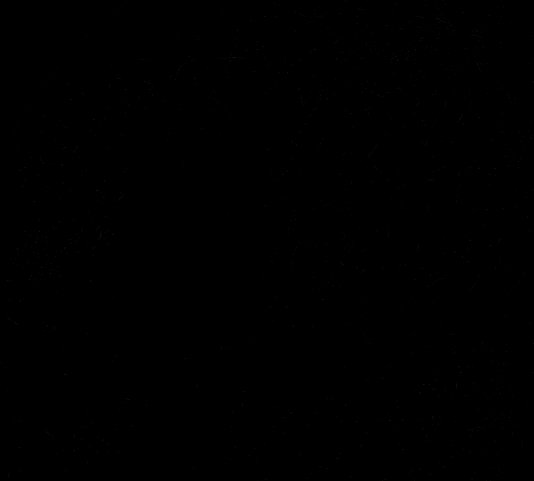

Supplement: S1 File — (ZIP) [file pone.0228610.s001.zip › 40_144/Br-11__IR_rec0370.jpg]

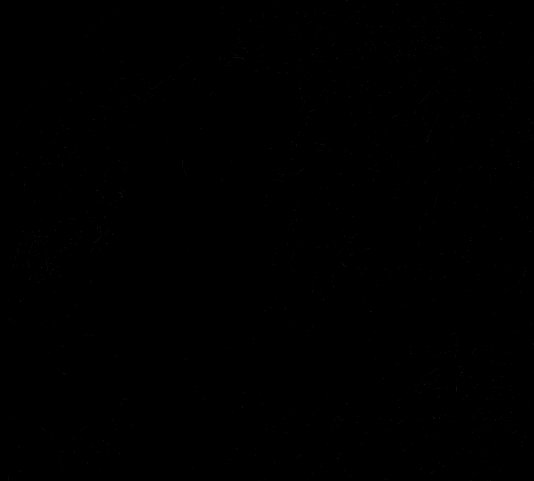

Supplement: S1 File — (ZIP) [file pone.0228610.s001.zip › 40_144/Br-11__IR_rec0374.jpg]

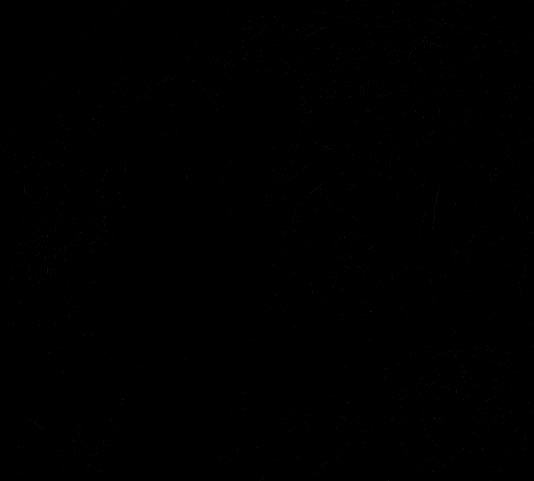

Supplement: S1 File — (ZIP) [file pone.0228610.s001.zip › 40_144/Br-11__IR_rec0378.jpg]

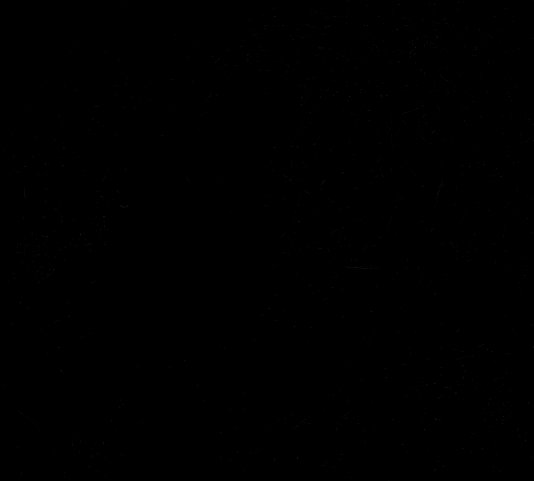

Supplement: S1 File — (ZIP) [file pone.0228610.s001.zip › 40_144/Br-11__IR_rec0382.jpg]

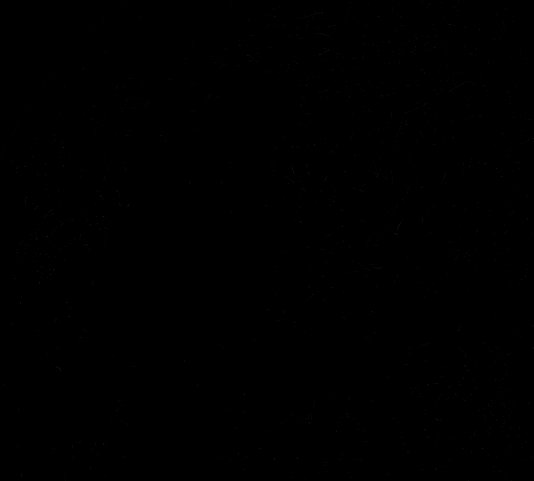

Supplement: S1 File — (ZIP) [file pone.0228610.s001.zip › 40_144/Br-11__IR_rec0386.jpg]

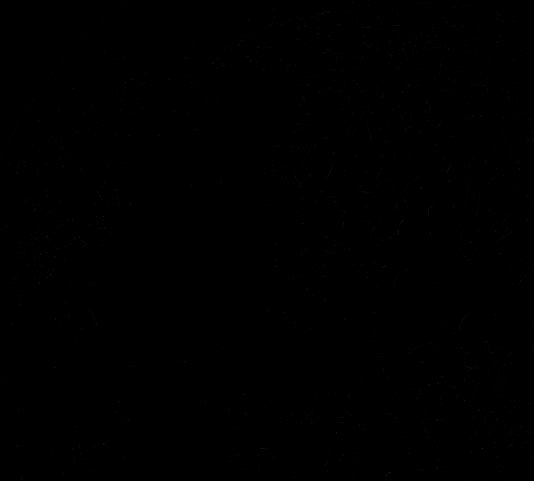

Supplement: S1 File — (ZIP) [file pone.0228610.s001.zip › 40_144/Br-11__IR_rec0390.jpg]

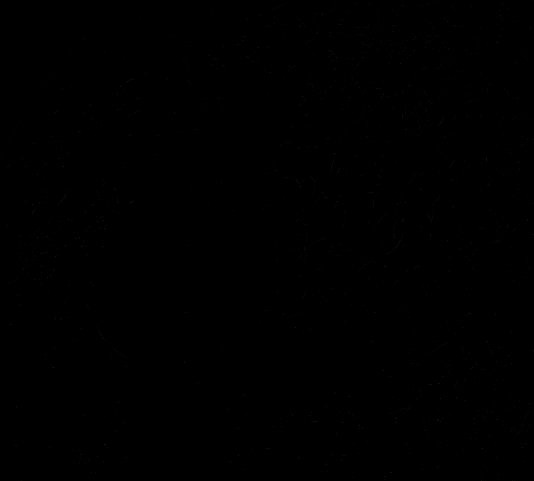

Supplement: S1 File — (ZIP) [file pone.0228610.s001.zip › 40_144/Br-11__IR_rec0394.jpg]

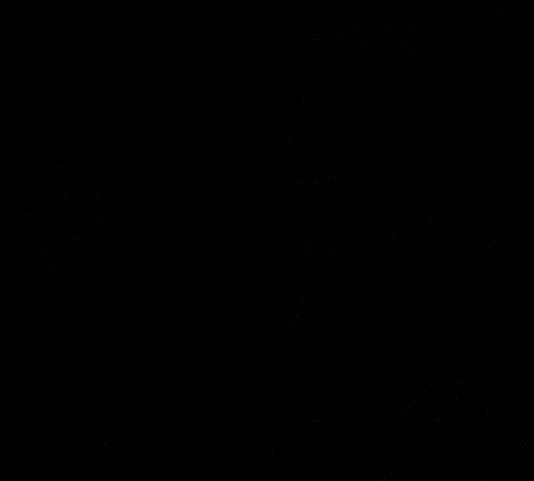

Supplement: S1 File — (ZIP) [file pone.0228610.s001.zip › 40_144/Br-11__IR_rec0398.jpg]

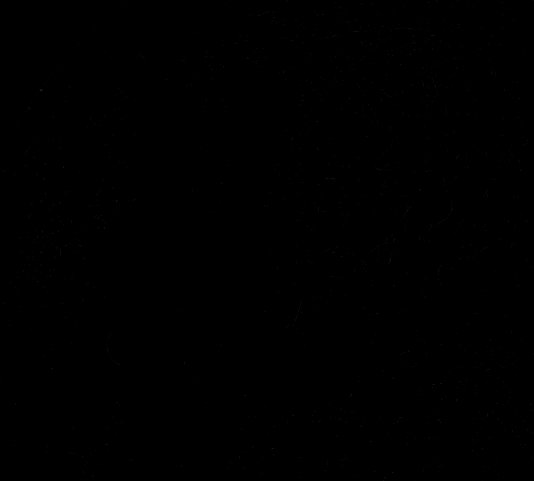

Supplement: S1 File — (ZIP) [file pone.0228610.s001.zip › 40_144/Br-11__IR_rec0402.jpg]

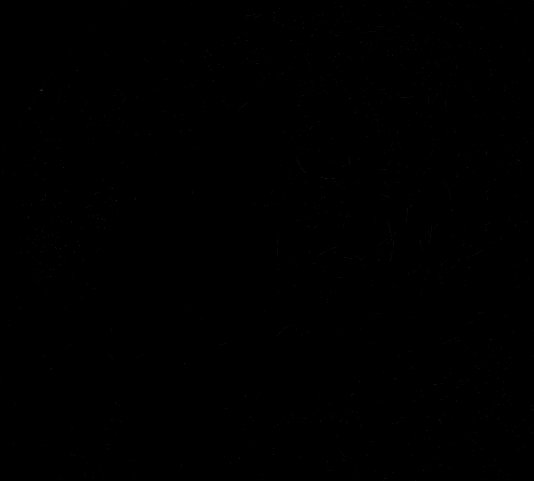

Supplement: S1 File — (ZIP) [file pone.0228610.s001.zip › 40_144/Br-11__IR_rec0406.jpg]

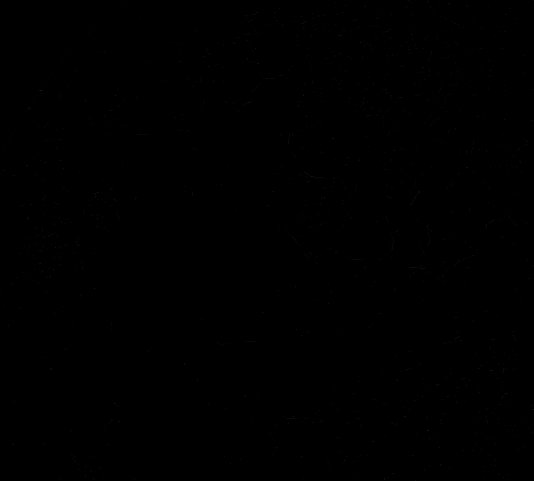

Supplement: S1 File — (ZIP) [file pone.0228610.s001.zip › 40_144/Br-11__IR_rec0410.jpg]

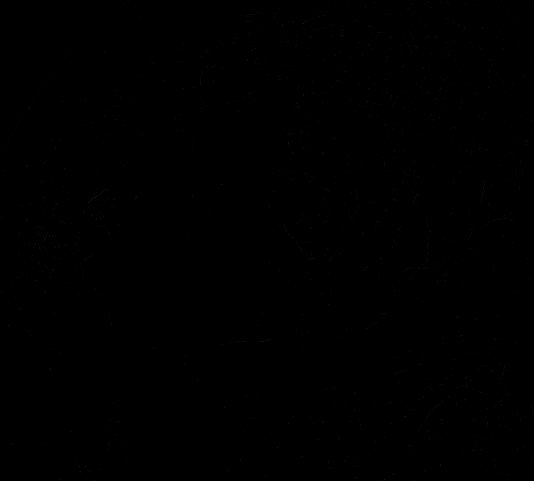

Supplement: S1 File — (ZIP) [file pone.0228610.s001.zip › 40_144/Br-11__IR_rec0414.jpg]

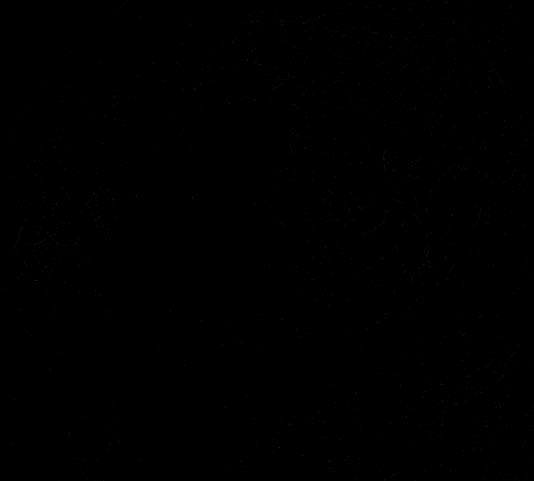

Supplement: S1 File — (ZIP) [file pone.0228610.s001.zip › 40_144/Br-11__IR_rec0418.jpg]

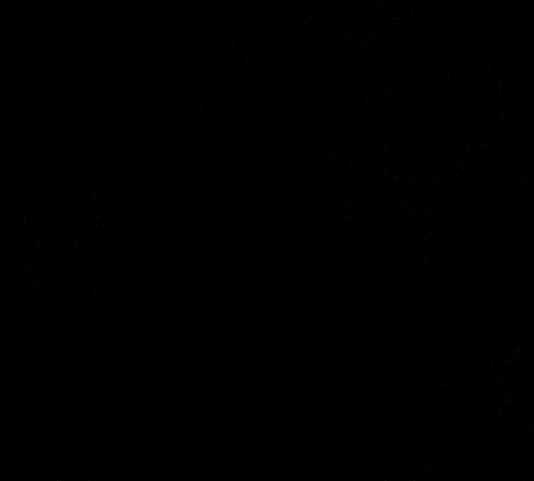

Supplement: S1 File — (ZIP) [file pone.0228610.s001.zip › 40_144/Br-11__IR_rec0422.jpg]

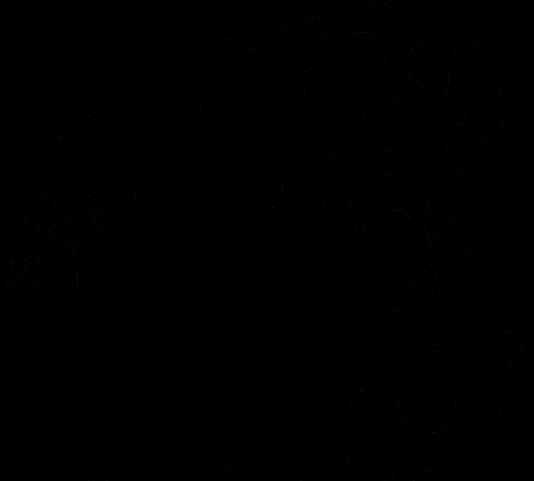

Supplement: S1 File — (ZIP) [file pone.0228610.s001.zip › 40_144/Br-11__IR_rec0426.jpg]

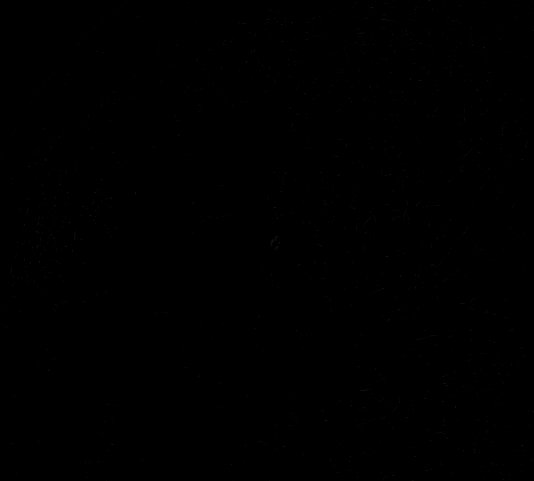

Supplement: S1 File — (ZIP) [file pone.0228610.s001.zip › 40_144/Br-11__IR_rec0430.jpg]

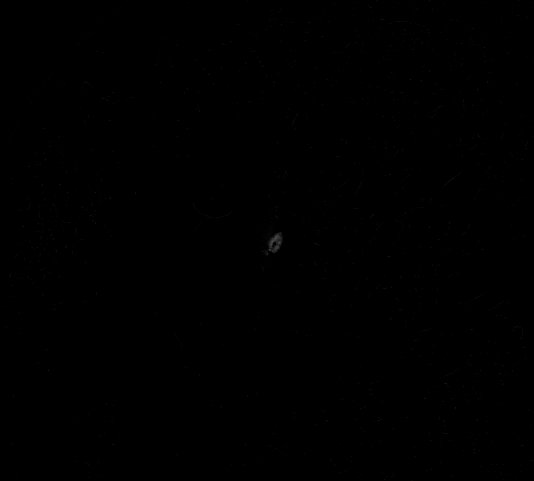

Supplement: S1 File — (ZIP) [file pone.0228610.s001.zip › 40_144/Br-11__IR_rec0434.jpg]

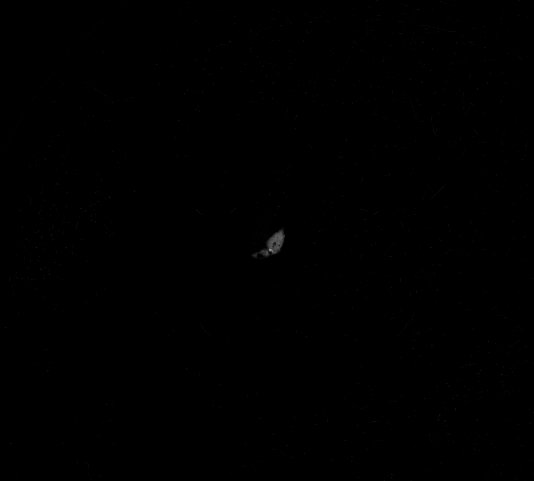

Supplement: S1 File — (ZIP) [file pone.0228610.s001.zip › 40_144/Br-11__IR_rec0438.jpg]

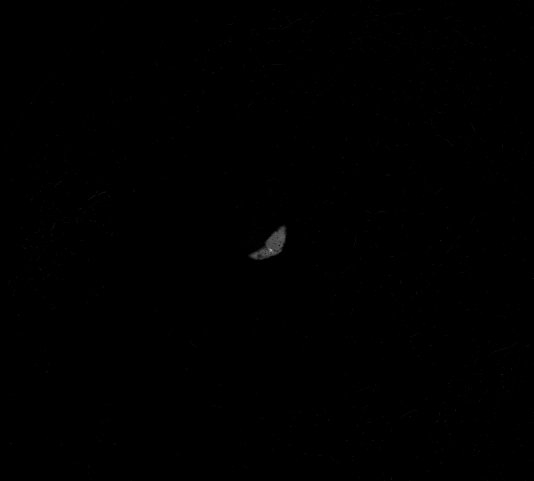

Supplement: S1 File — (ZIP) [file pone.0228610.s001.zip › 40_144/Br-11__IR_rec0442.jpg]

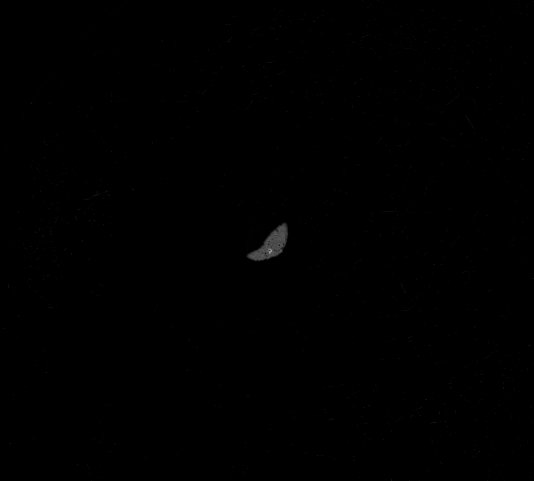

Supplement: S1 File — (ZIP) [file pone.0228610.s001.zip › 40_144/Br-11__IR_rec0446.jpg]

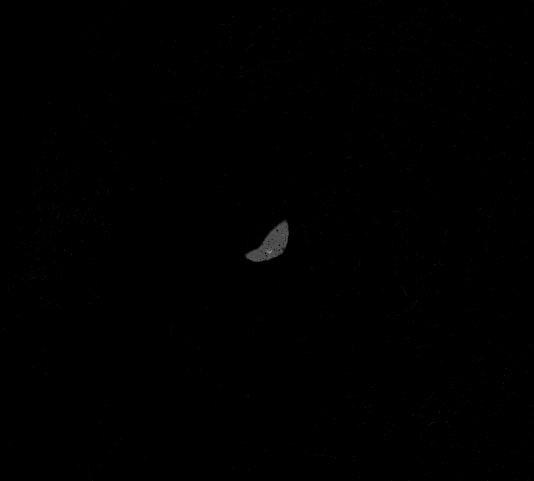

Supplement: S1 File — (ZIP) [file pone.0228610.s001.zip › 40_144/Br-11__IR_rec0450.jpg]

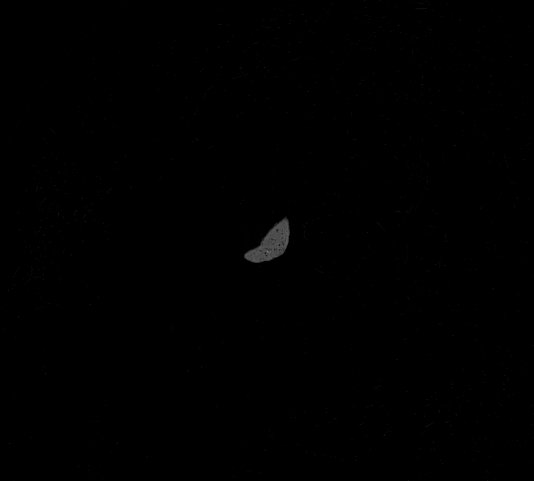

Supplement: S1 File — (ZIP) [file pone.0228610.s001.zip › 40_144/Br-11__IR_rec0454.jpg]

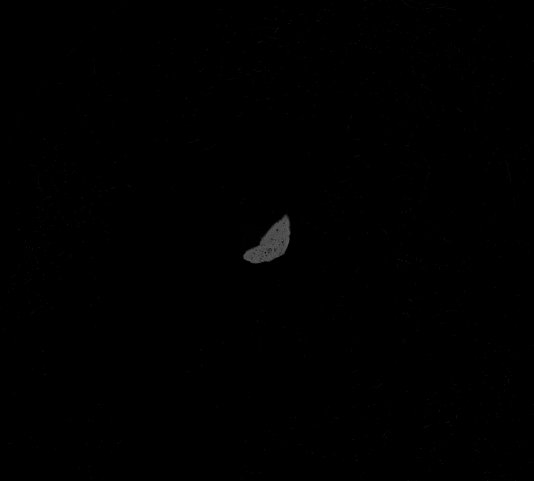

Supplement: S1 File — (ZIP) [file pone.0228610.s001.zip › 40_144/Br-11__IR_rec0458.jpg]

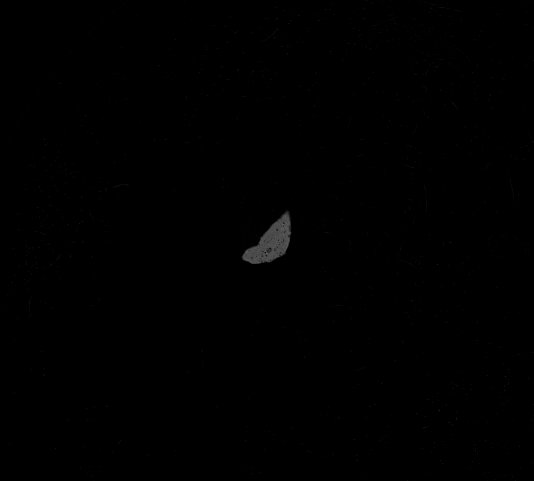

Supplement: S1 File — (ZIP) [file pone.0228610.s001.zip › 40_144/Br-11__IR_rec0462.jpg]

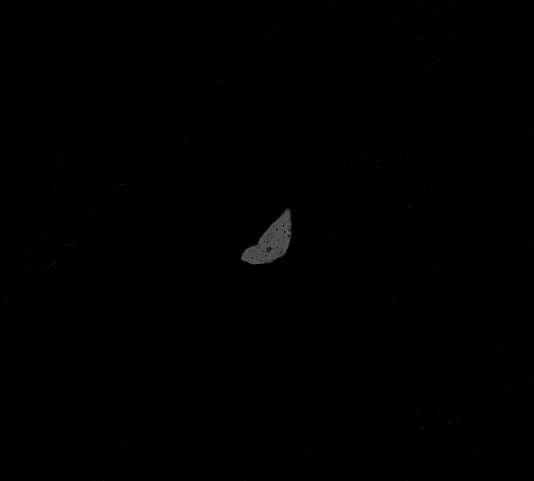

Supplement: S1 File — (ZIP) [file pone.0228610.s001.zip › 40_144/Br-11__IR_rec0466.jpg]

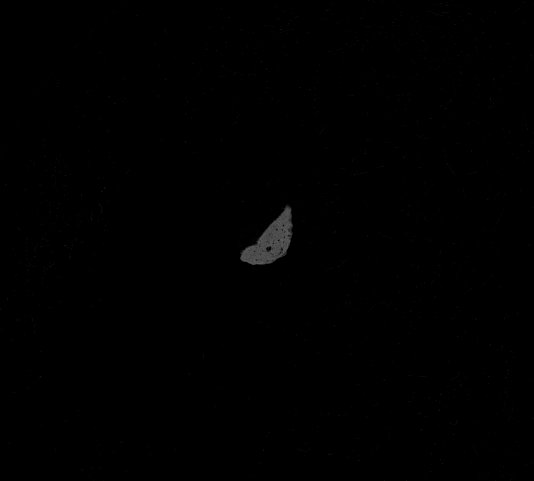

Supplement: S1 File — (ZIP) [file pone.0228610.s001.zip › 40_144/Br-11__IR_rec0470.jpg]

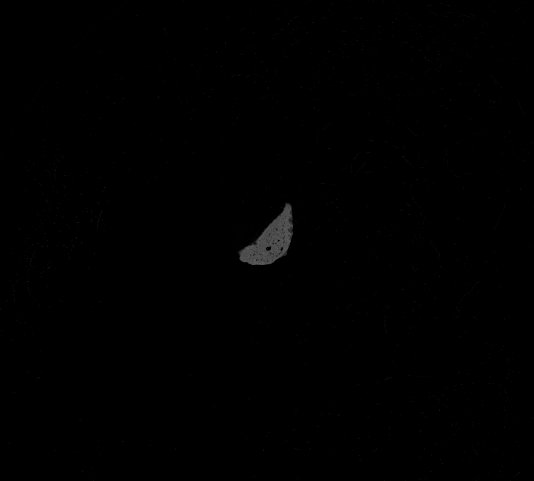

Supplement: S1 File — (ZIP) [file pone.0228610.s001.zip › 40_144/Br-11__IR_rec0474.jpg]

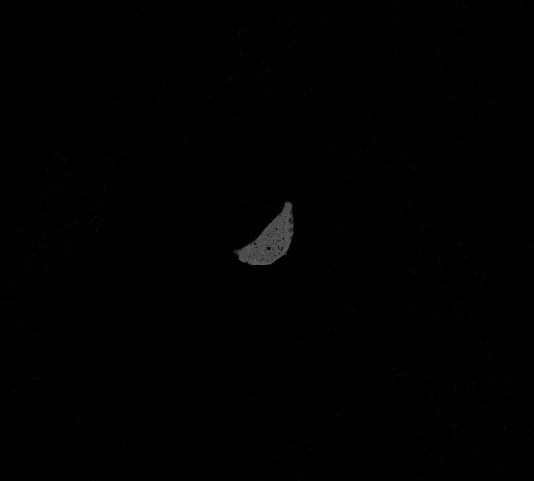

Supplement: S1 File — (ZIP) [file pone.0228610.s001.zip › 40_144/Br-11__IR_rec0478.jpg]

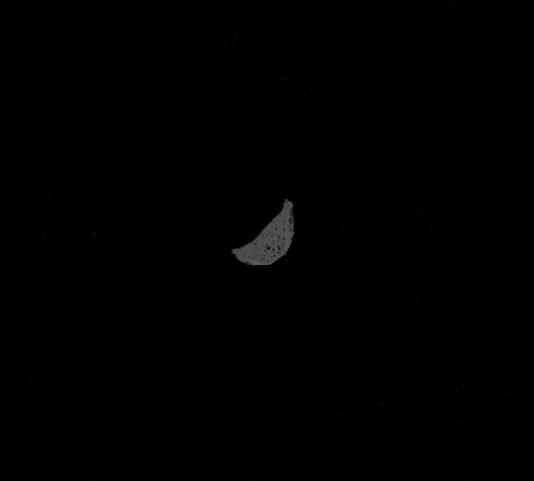

Supplement: S1 File — (ZIP) [file pone.0228610.s001.zip › 40_144/Br-11__IR_rec0482.jpg]

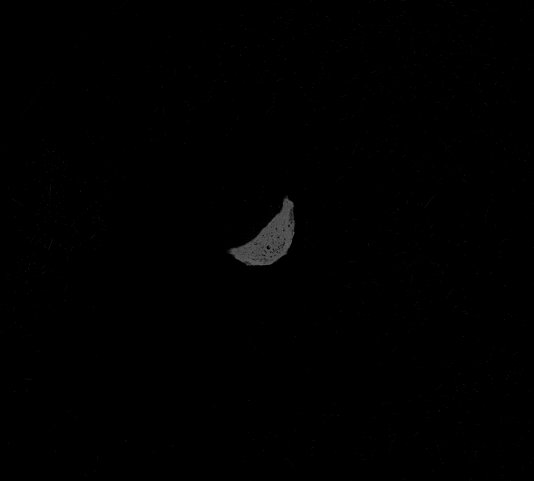

Supplement: S1 File — (ZIP) [file pone.0228610.s001.zip › 40_144/Br-11__IR_rec0486.jpg]

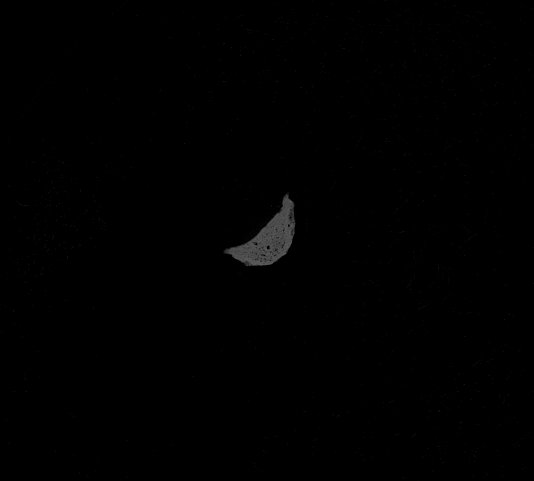

Supplement: S1 File — (ZIP) [file pone.0228610.s001.zip › 40_144/Br-11__IR_rec0490.jpg]

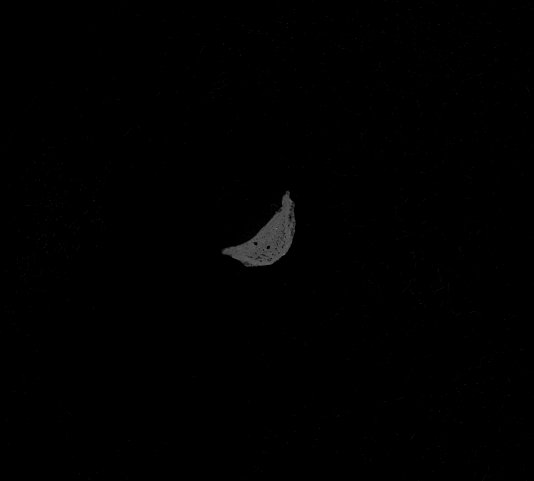

Supplement: S1 File — (ZIP) [file pone.0228610.s001.zip › 40_144/Br-11__IR_rec0494.jpg]

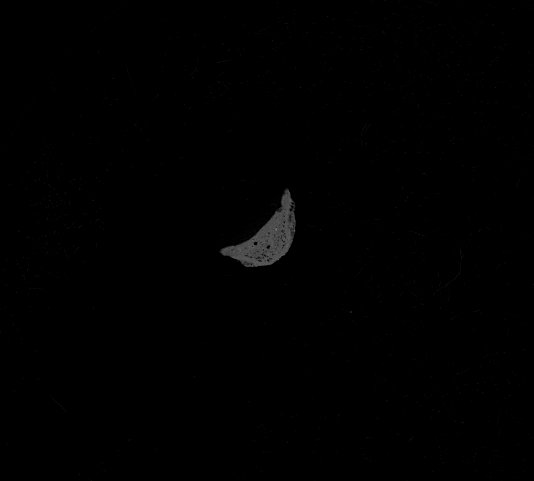

Supplement: S1 File — (ZIP) [file pone.0228610.s001.zip › 40_144/Br-11__IR_rec0498.jpg]

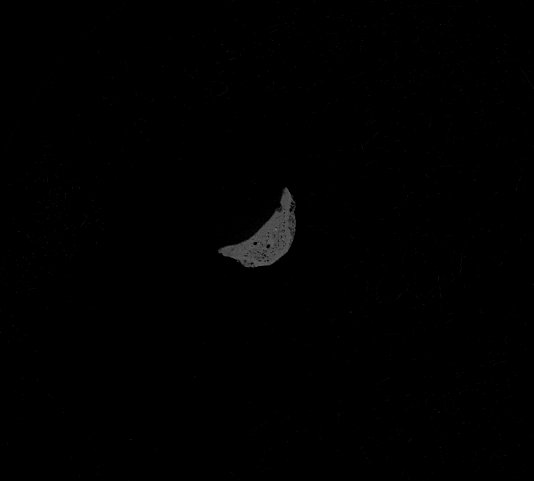

Supplement: S1 File — (ZIP) [file pone.0228610.s001.zip › 40_144/Br-11__IR_rec0502.jpg]

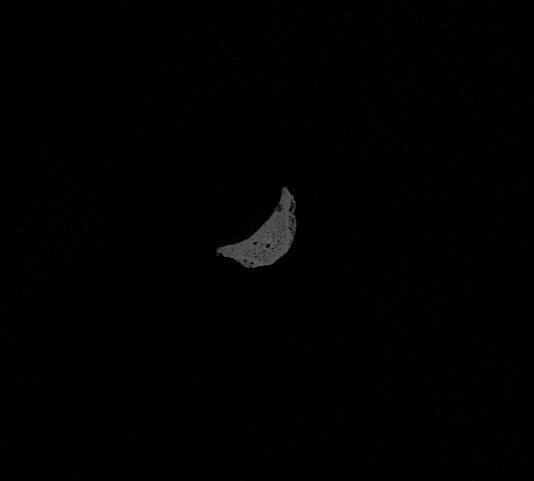

Supplement: S1 File — (ZIP) [file pone.0228610.s001.zip › 40_144/Br-11__IR_rec0506.jpg]

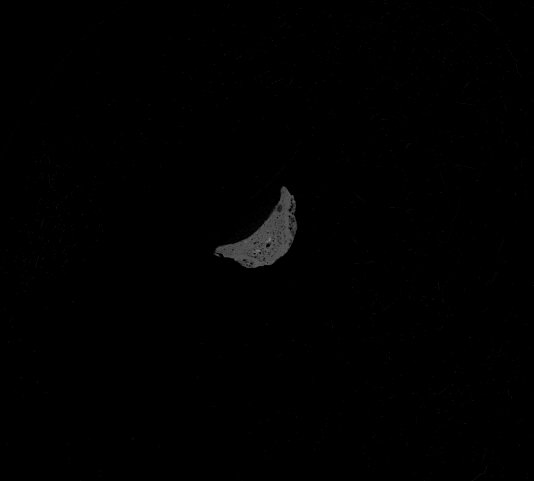

Supplement: S1 File — (ZIP) [file pone.0228610.s001.zip › 40_144/Br-11__IR_rec0510.jpg]

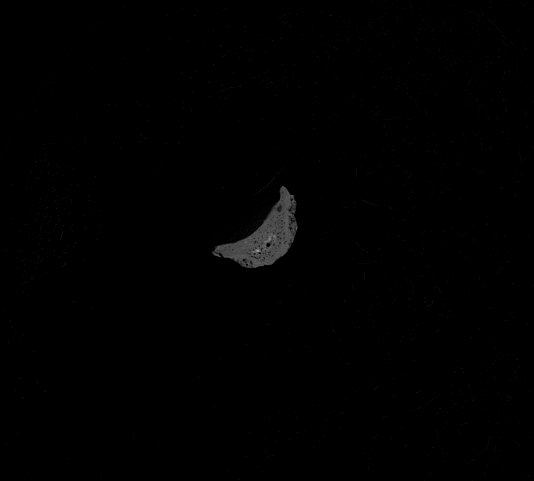

Supplement: S1 File — (ZIP) [file pone.0228610.s001.zip › 40_144/Br-11__IR_rec0514.jpg]

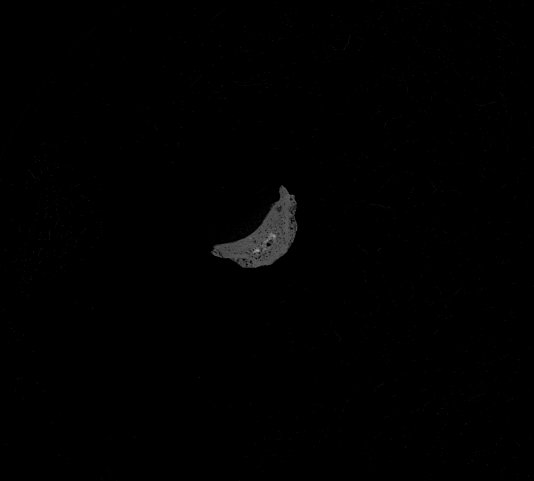

Supplement: S1 File — (ZIP) [file pone.0228610.s001.zip › 40_144/Br-11__IR_rec0518.jpg]

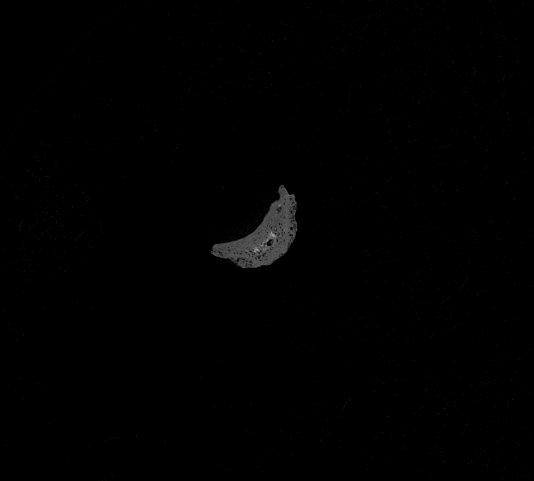

Supplement: S1 File — (ZIP) [file pone.0228610.s001.zip › 40_144/Br-11__IR_rec0522.jpg]

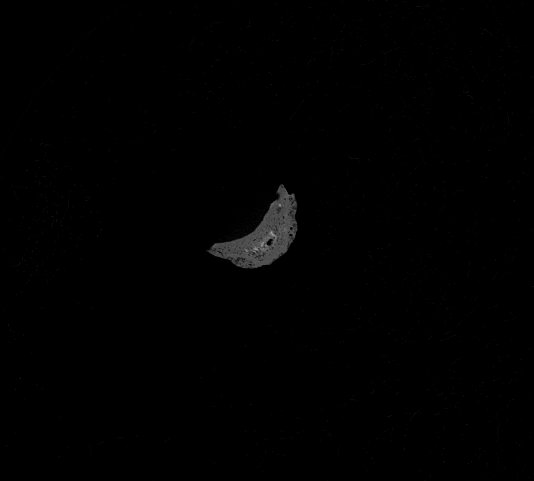

Supplement: S1 File — (ZIP) [file pone.0228610.s001.zip › 40_144/Br-11__IR_rec0526.jpg]

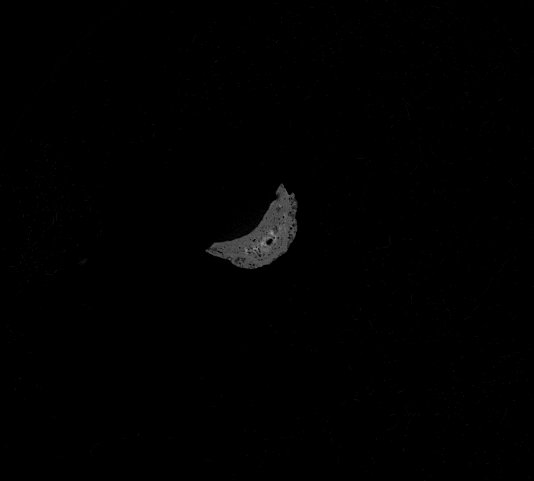

Supplement: S1 File — (ZIP) [file pone.0228610.s001.zip › 40_144/Br-11__IR_rec0530.jpg]

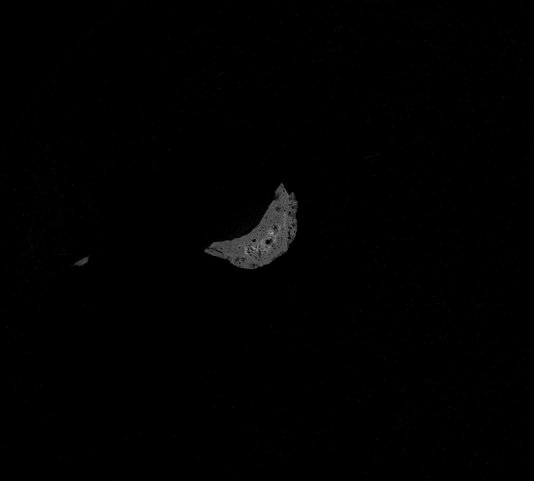

Supplement: S1 File — (ZIP) [file pone.0228610.s001.zip › 40_144/Br-11__IR_rec0534.jpg]

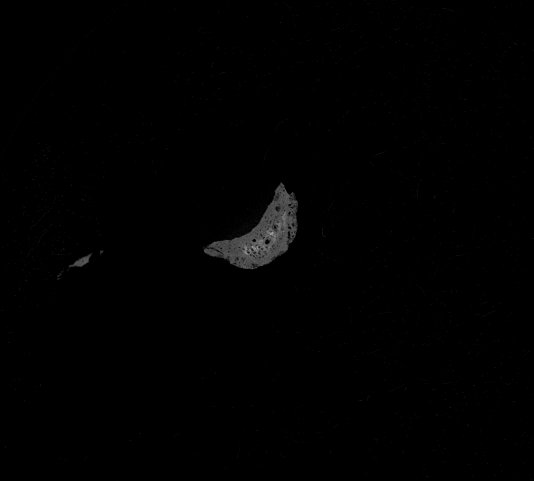

Supplement: S1 File — (ZIP) [file pone.0228610.s001.zip › 40_144/Br-11__IR_rec0538.jpg]

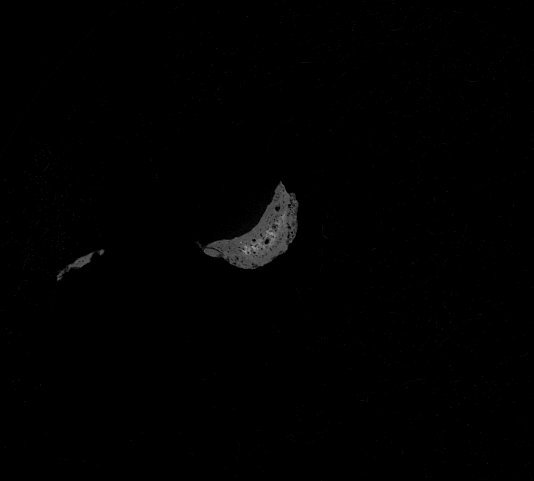

Supplement: S1 File — (ZIP) [file pone.0228610.s001.zip › 40_144/Br-11__IR_rec0542.jpg]

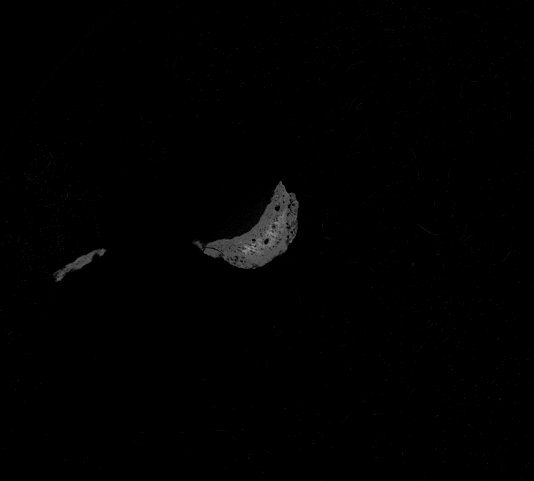

Supplement: S1 File — (ZIP) [file pone.0228610.s001.zip › 40_144/Br-11__IR_rec0546.jpg]

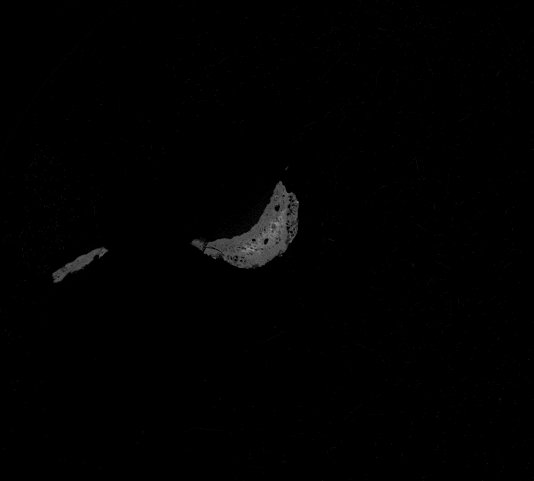

Supplement: S1 File — (ZIP) [file pone.0228610.s001.zip › 40_144/Br-11__IR_rec0550.jpg]

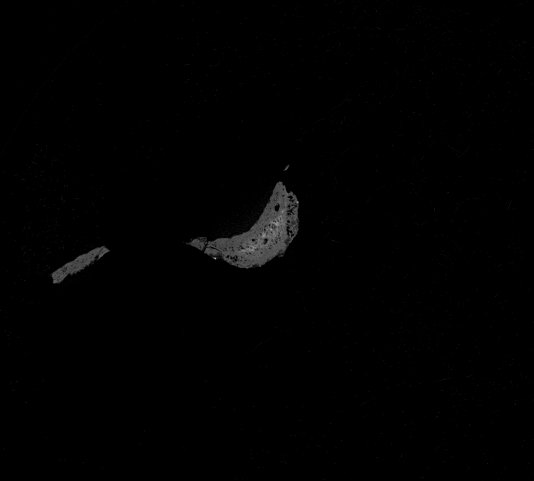

Supplement: S1 File — (ZIP) [file pone.0228610.s001.zip › 40_144/Br-11__IR_rec0554.jpg]

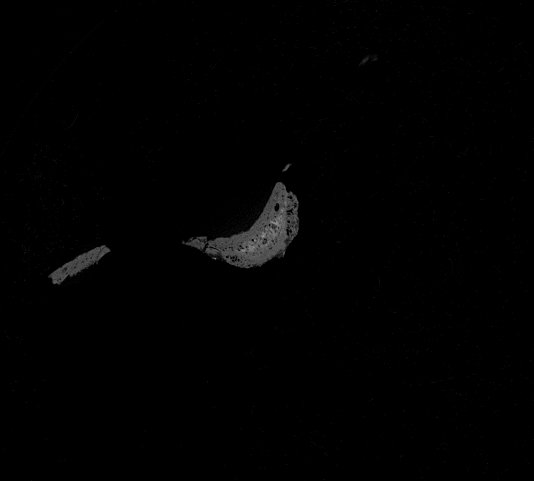

Supplement: S1 File — (ZIP) [file pone.0228610.s001.zip › 40_144/Br-11__IR_rec0558.jpg]

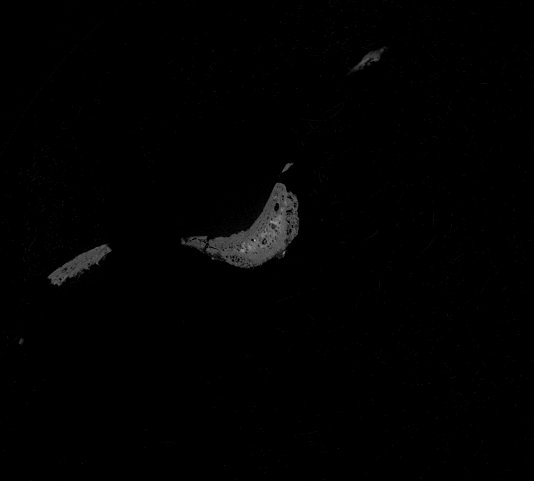

Supplement: S1 File — (ZIP) [file pone.0228610.s001.zip › 40_144/Br-11__IR_rec0562.jpg]

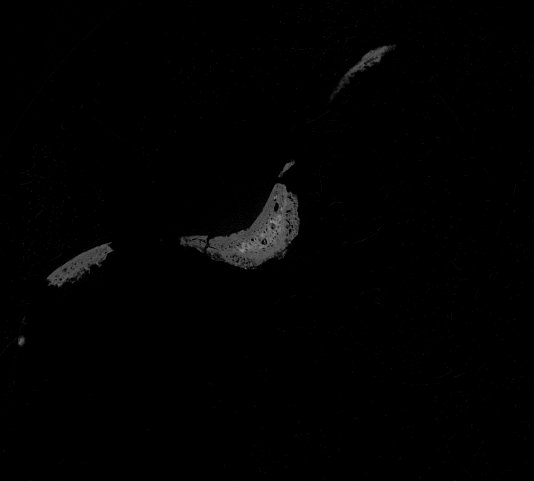

Supplement: S1 File — (ZIP) [file pone.0228610.s001.zip › 40_144/Br-11__IR_rec0566.jpg]

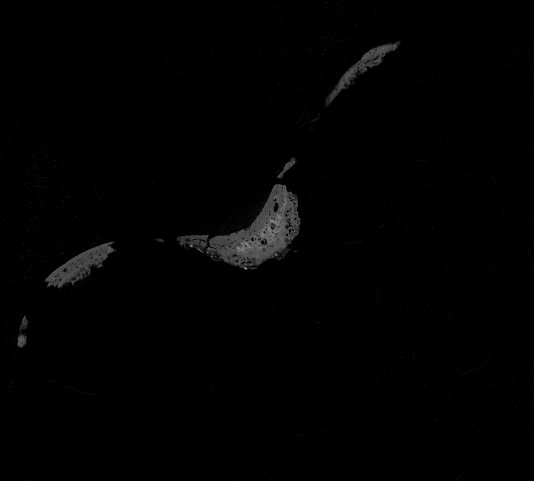

Supplement: S1 File — (ZIP) [file pone.0228610.s001.zip › 40_144/Br-11__IR_rec0570.jpg]

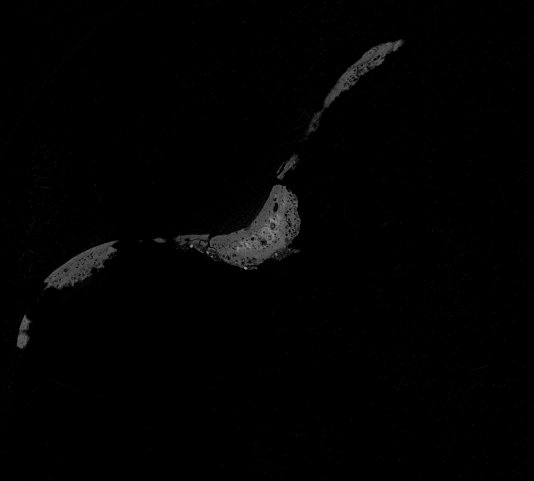

Supplement: S1 File — (ZIP) [file pone.0228610.s001.zip › 40_144/Br-11__IR_rec0574.jpg]

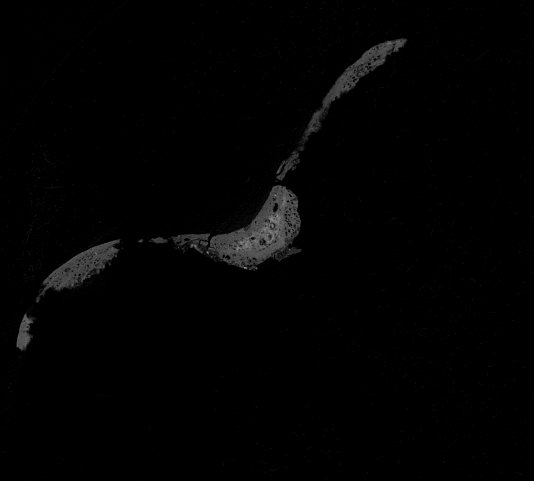

Supplement: S1 File — (ZIP) [file pone.0228610.s001.zip › 40_144/Br-11__IR_rec0578.jpg]

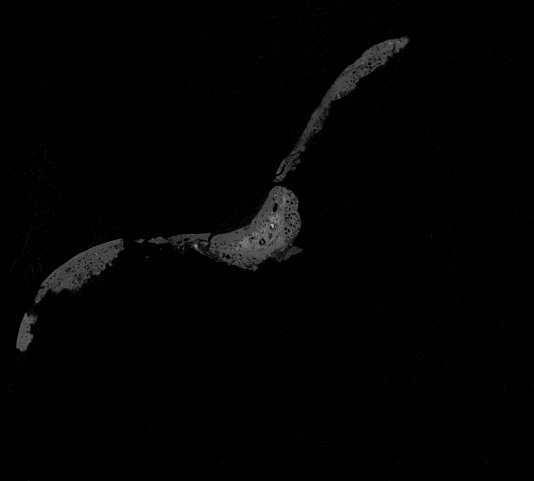

Supplement: S1 File — (ZIP) [file pone.0228610.s001.zip › 40_144/Br-11__IR_rec0582.jpg]

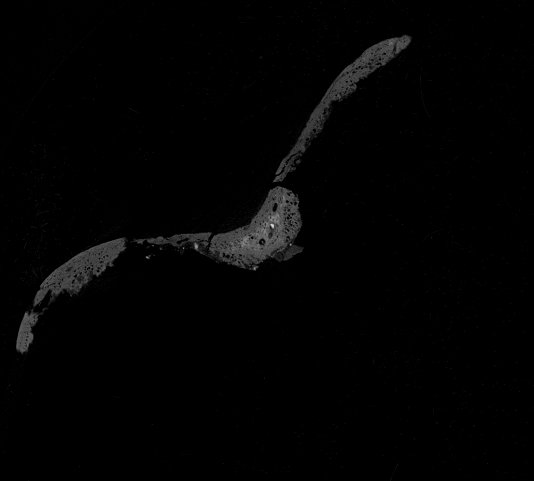

Supplement: S1 File — (ZIP) [file pone.0228610.s001.zip › 40_144/Br-11__IR_rec0586.jpg]

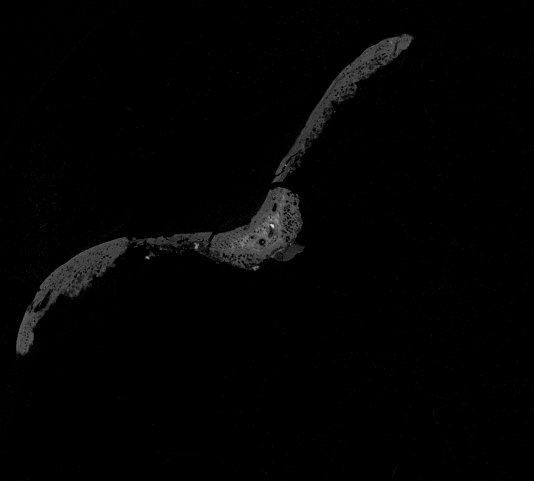

Supplement: S1 File — (ZIP) [file pone.0228610.s001.zip › 40_144/Br-11__IR_rec0590.jpg]

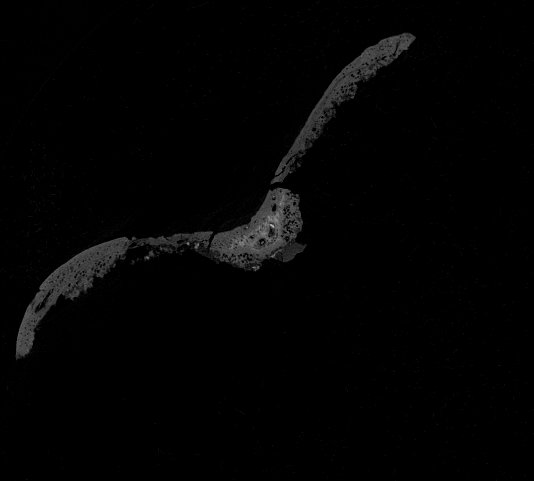

Supplement: S1 File — (ZIP) [file pone.0228610.s001.zip › 40_144/Br-11__IR_rec0594.jpg]

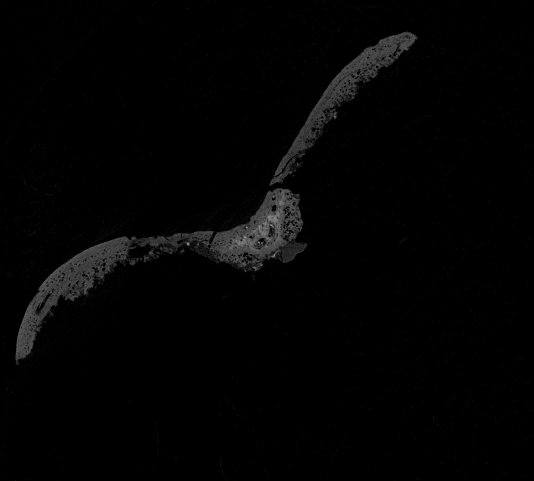

Supplement: S1 File — (ZIP) [file pone.0228610.s001.zip › 40_144/Br-11__IR_rec0598.jpg]

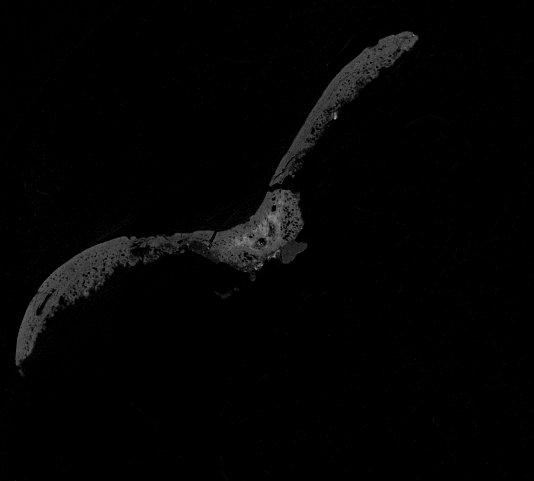

Supplement: S1 File — (ZIP) [file pone.0228610.s001.zip › 40_144/Br-11__IR_rec0602.jpg]

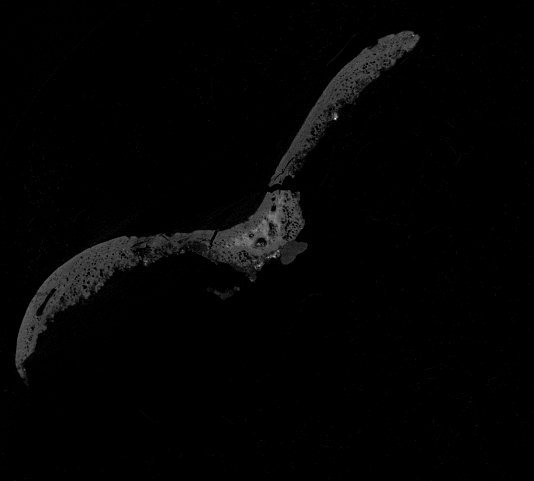

Supplement: S1 File — (ZIP) [file pone.0228610.s001.zip › 40_144/Br-11__IR_rec0606.jpg]

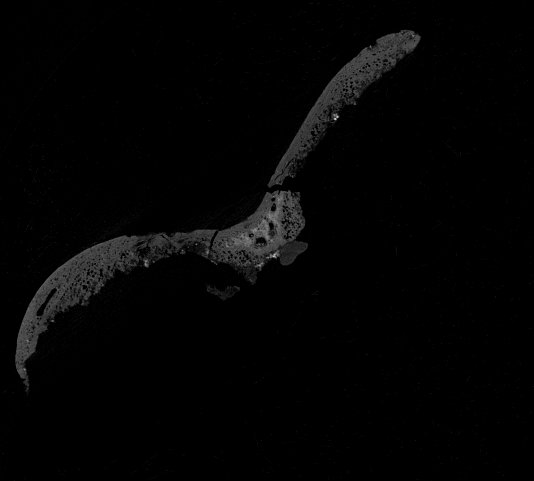

Supplement: S1 File — (ZIP) [file pone.0228610.s001.zip › 40_144/Br-11__IR_rec0610.jpg]

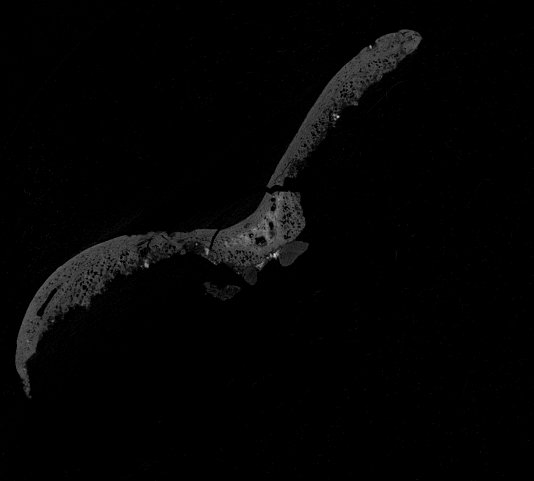

Supplement: S1 File — (ZIP) [file pone.0228610.s001.zip › 40_144/Br-11__IR_rec0614.jpg]

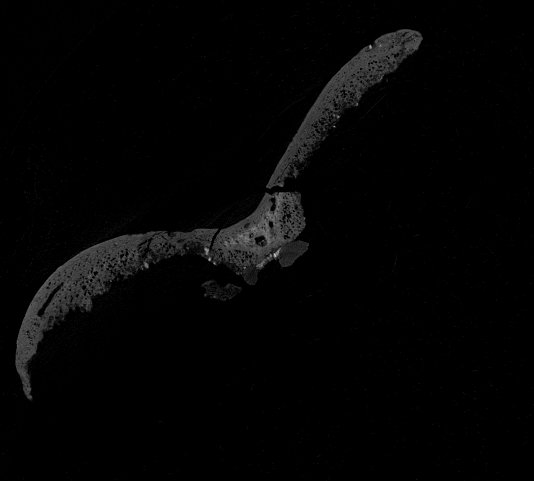

Supplement: S1 File — (ZIP) [file pone.0228610.s001.zip › 40_144/Br-11__IR_rec0618.jpg]

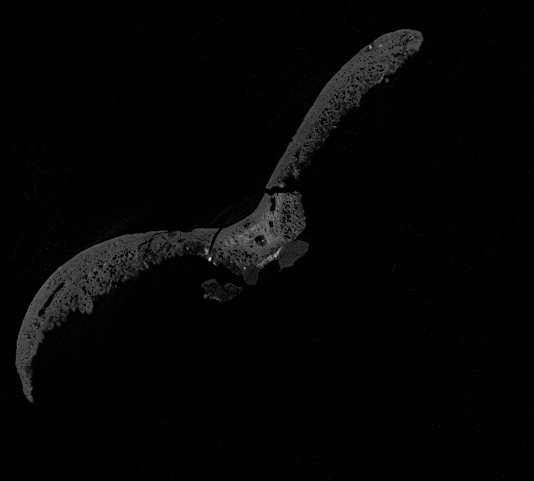

Supplement: S1 File — (ZIP) [file pone.0228610.s001.zip › 40_144/Br-11__IR_rec0622.jpg]

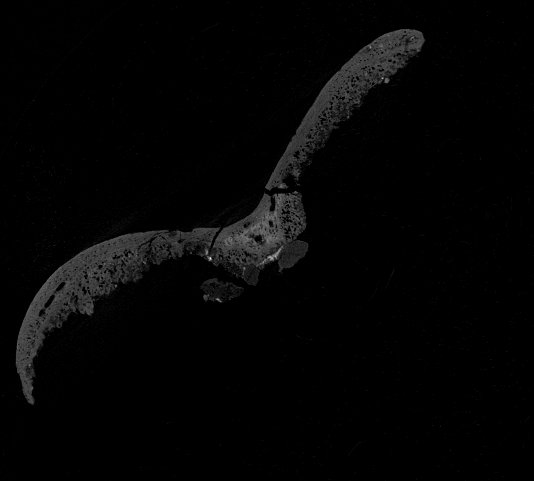

Supplement: S1 File — (ZIP) [file pone.0228610.s001.zip › 40_144/Br-11__IR_rec0626.jpg]

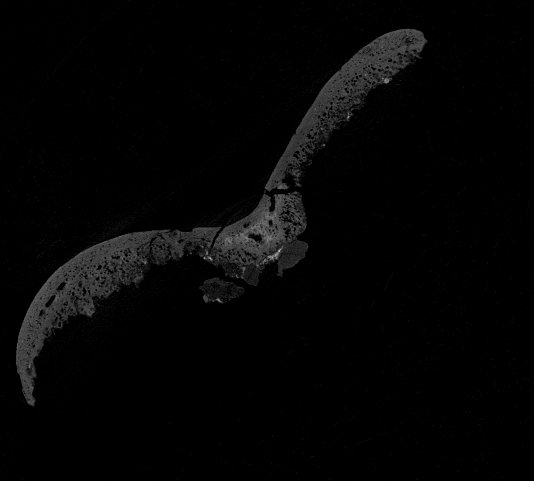

Supplement: S1 File — (ZIP) [file pone.0228610.s001.zip › 40_144/Br-11__IR_rec0630.jpg]

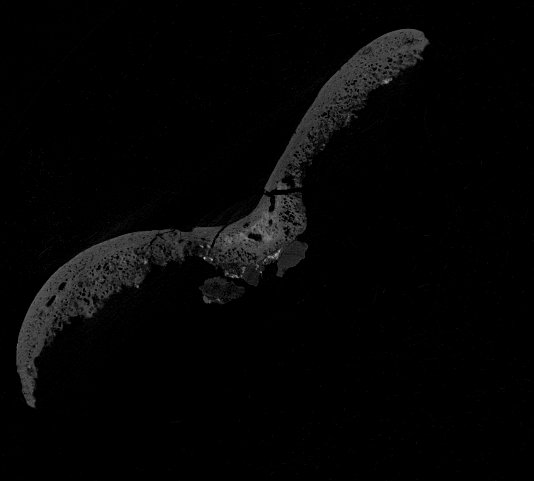

Supplement: S1 File — (ZIP) [file pone.0228610.s001.zip › 40_144/Br-11__IR_rec0634.jpg]

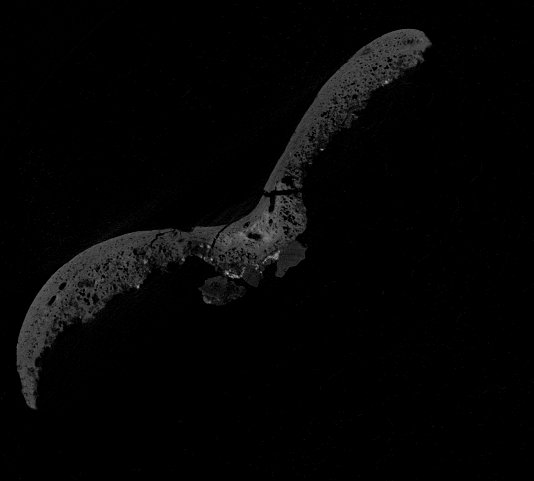

Supplement: S1 File — (ZIP) [file pone.0228610.s001.zip › 40_144/Br-11__IR_rec0638.jpg]

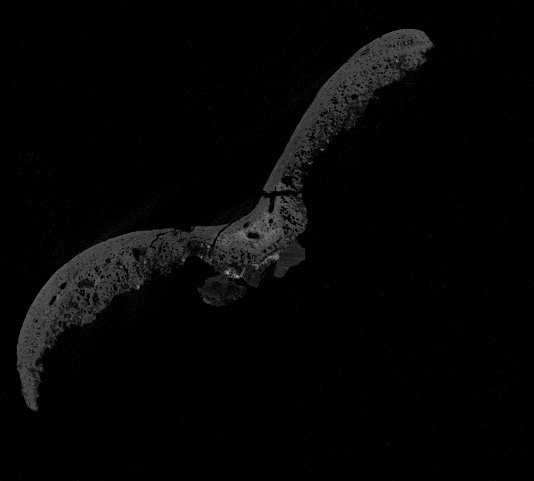

Supplement: S1 File — (ZIP) [file pone.0228610.s001.zip › 40_144/Br-11__IR_rec0642.jpg]

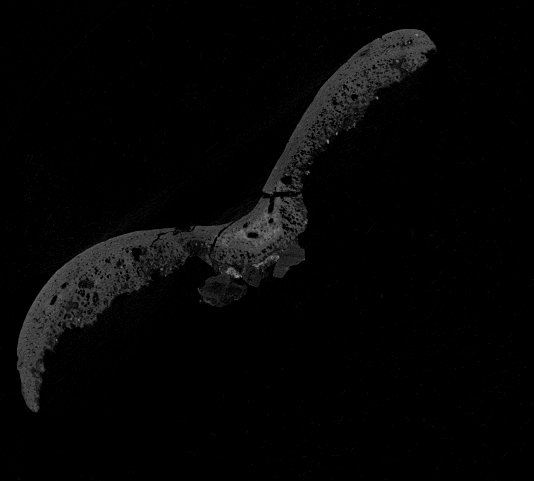

Supplement: S1 File — (ZIP) [file pone.0228610.s001.zip › 40_144/Br-11__IR_rec0646.jpg]

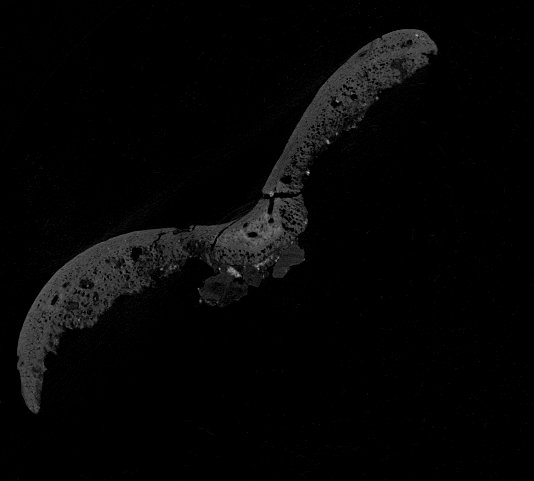

Supplement: S1 File — (ZIP) [file pone.0228610.s001.zip › 40_144/Br-11__IR_rec0650.jpg]

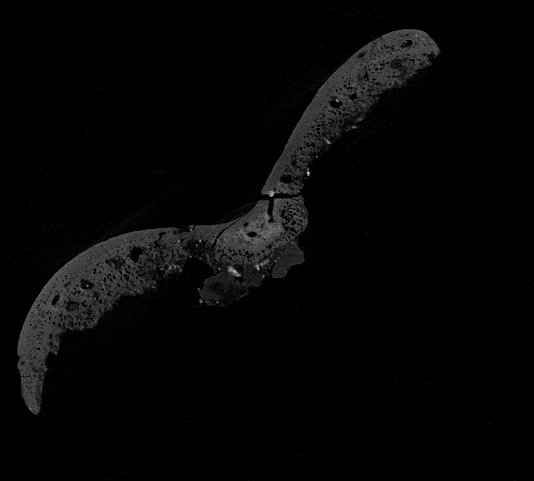

Supplement: S1 File — (ZIP) [file pone.0228610.s001.zip › 40_144/Br-11__IR_rec0654.jpg]

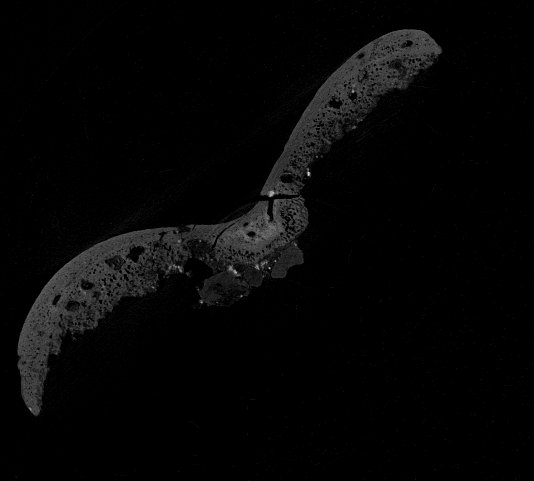

Supplement: S1 File — (ZIP) [file pone.0228610.s001.zip › 40_144/Br-11__IR_rec0658.jpg]

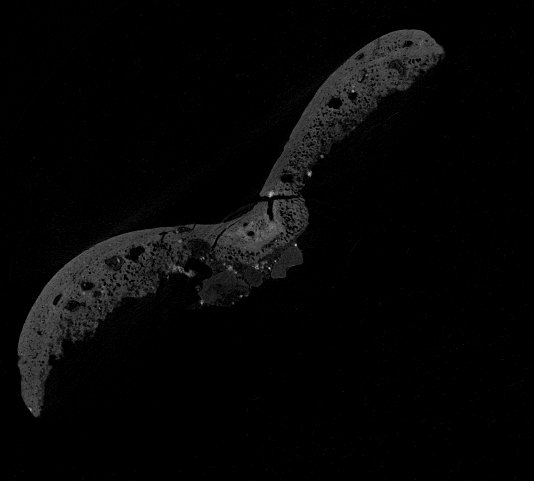

Supplement: S1 File — (ZIP) [file pone.0228610.s001.zip › 40_144/Br-11__IR_rec0662.jpg]

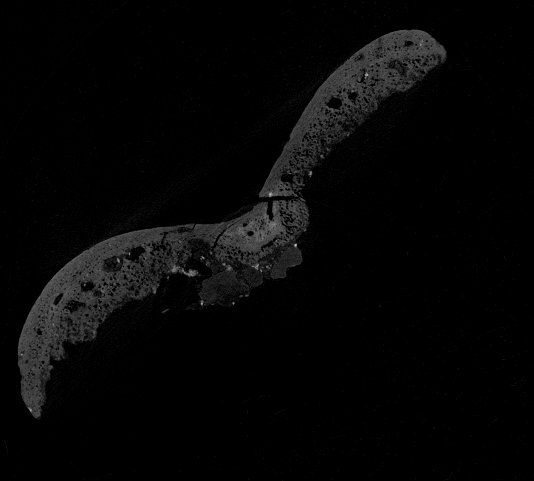

Supplement: S1 File — (ZIP) [file pone.0228610.s001.zip › 40_144/Br-11__IR_rec0666.jpg]

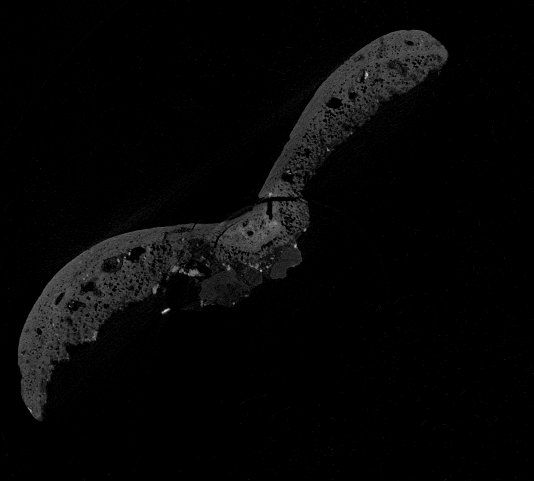

Supplement: S1 File — (ZIP) [file pone.0228610.s001.zip › 40_144/Br-11__IR_rec0670.jpg]

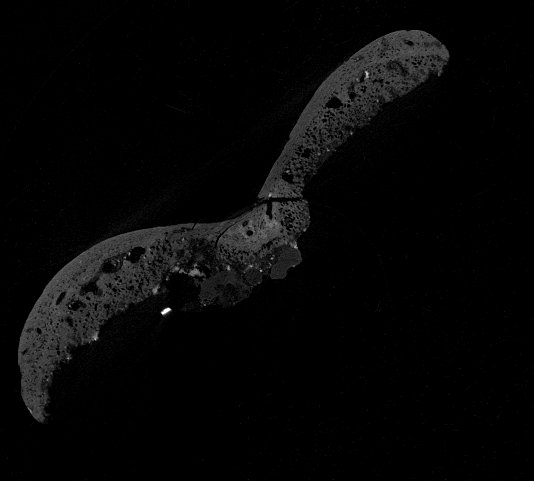

Supplement: S1 File — (ZIP) [file pone.0228610.s001.zip › 40_144/Br-11__IR_rec0674.jpg]

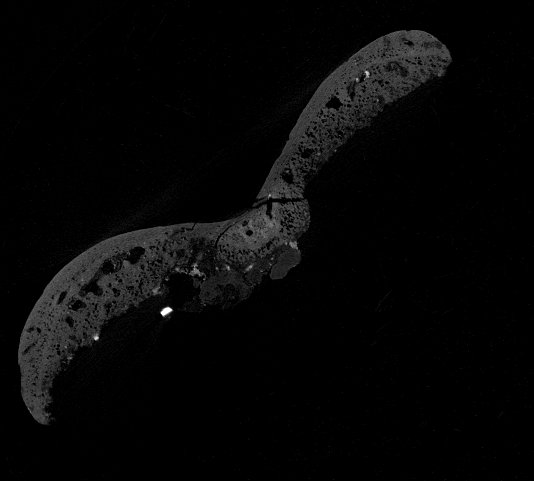

Supplement: S1 File — (ZIP) [file pone.0228610.s001.zip › 40_144/Br-11__IR_rec0678.jpg]

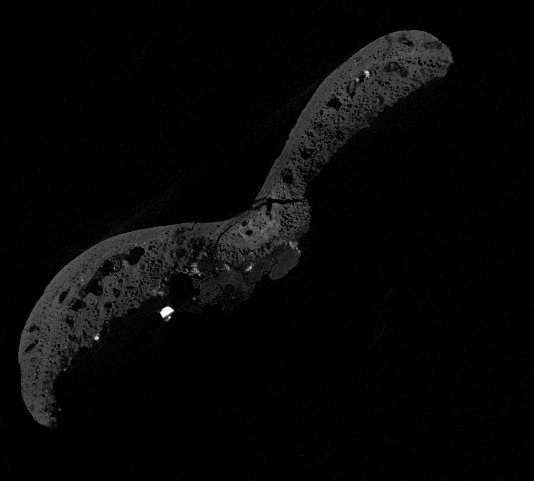

Supplement: S1 File — (ZIP) [file pone.0228610.s001.zip › 40_144/Br-11__IR_rec0682.jpg]

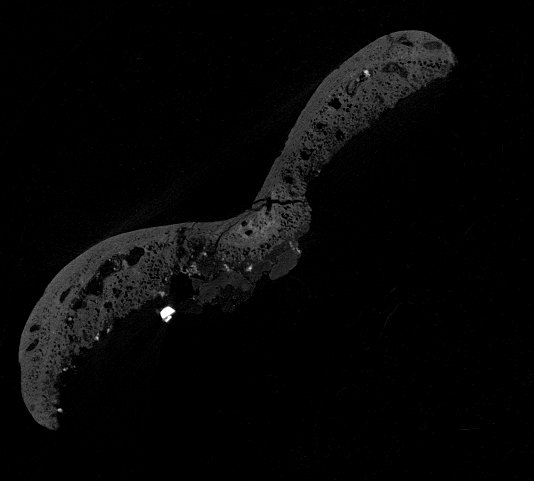

Supplement: S1 File — (ZIP) [file pone.0228610.s001.zip › 40_144/Br-11__IR_rec0686.jpg]

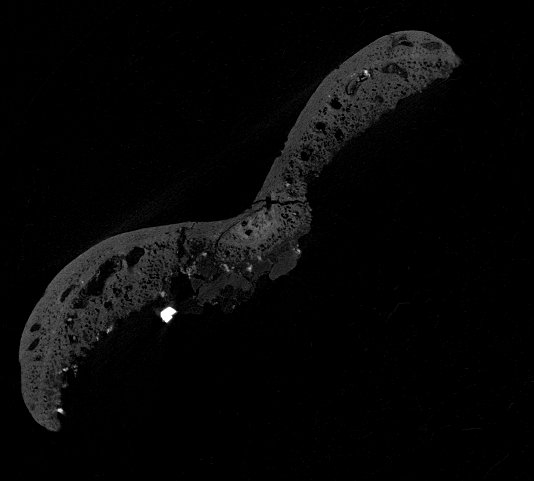

Supplement: S1 File — (ZIP) [file pone.0228610.s001.zip › 40_144/Br-11__IR_rec0690.jpg]

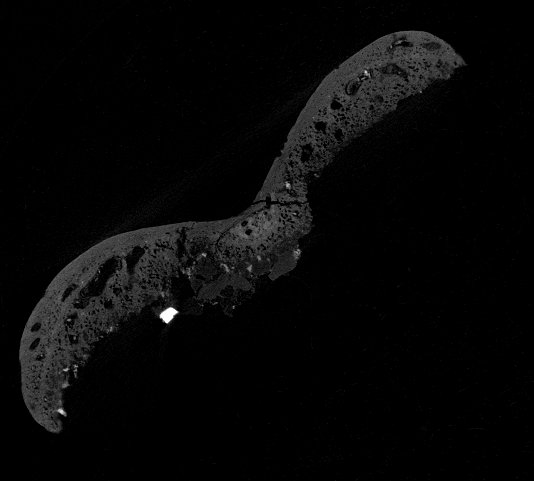

Supplement: S1 File — (ZIP) [file pone.0228610.s001.zip › 40_144/Br-11__IR_rec0694.jpg]

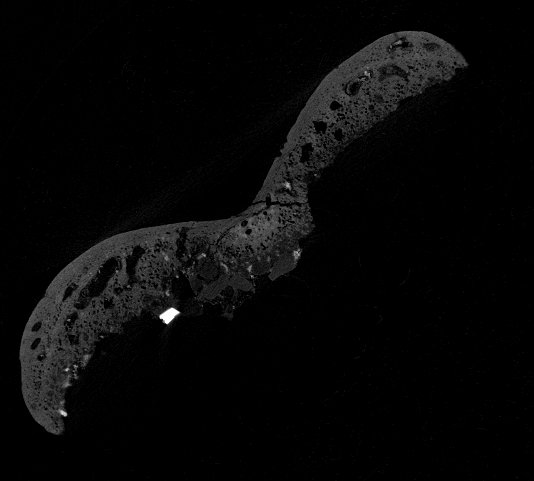

Supplement: S1 File — (ZIP) [file pone.0228610.s001.zip › 40_144/Br-11__IR_rec0698.jpg]

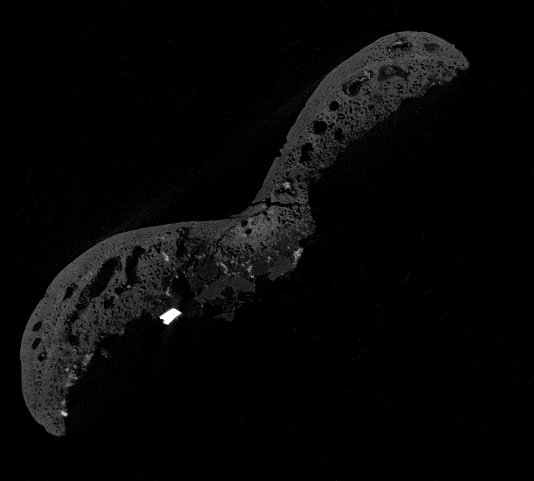

Supplement: S1 File — (ZIP) [file pone.0228610.s001.zip › 40_144/Br-11__IR_rec0702.jpg]

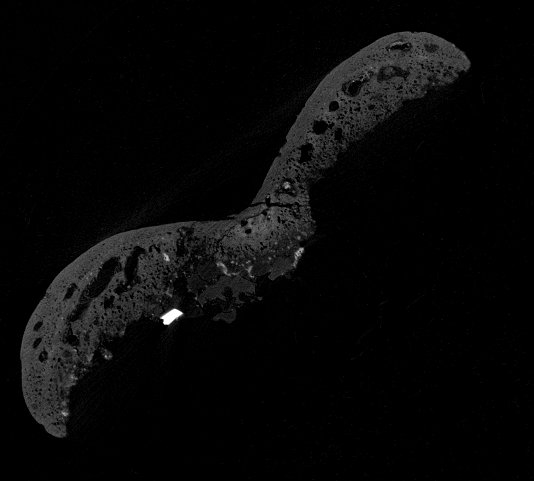

Supplement: S1 File — (ZIP) [file pone.0228610.s001.zip › 40_144/Br-11__IR_rec0706.jpg]

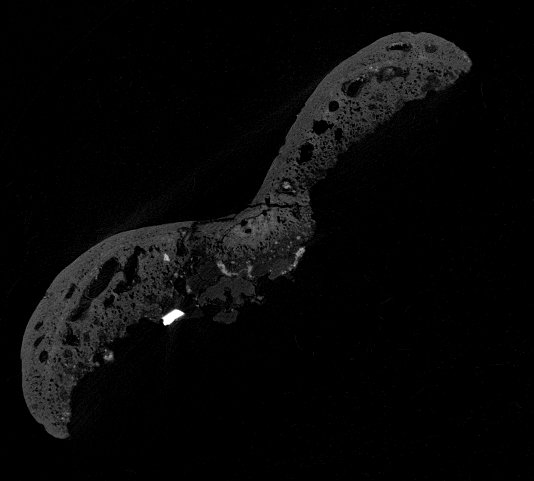

Supplement: S1 File — (ZIP) [file pone.0228610.s001.zip › 40_144/Br-11__IR_rec0710.jpg]

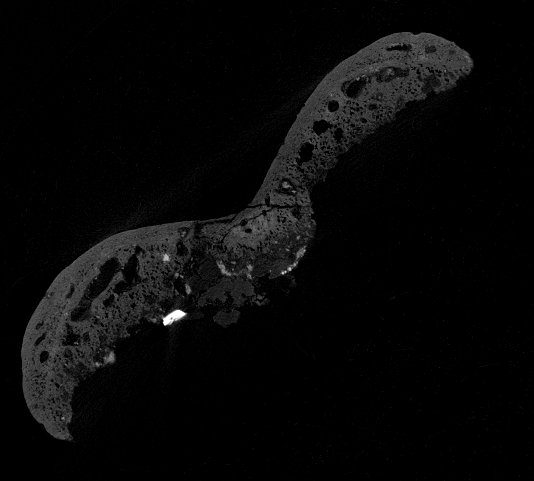

Supplement: S1 File — (ZIP) [file pone.0228610.s001.zip › 40_144/Br-11__IR_rec0714.jpg]

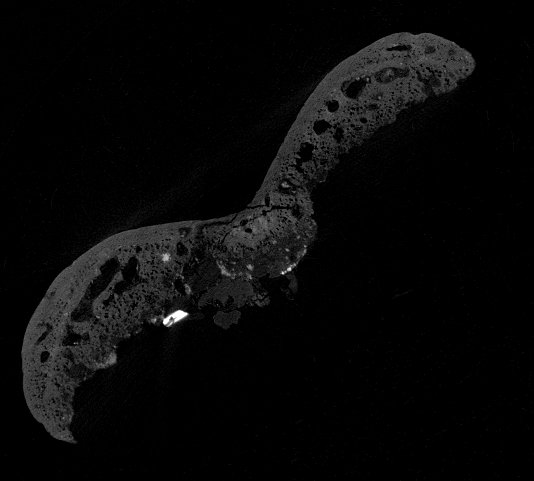

Supplement: S1 File — (ZIP) [file pone.0228610.s001.zip › 40_144/Br-11__IR_rec0718.jpg]

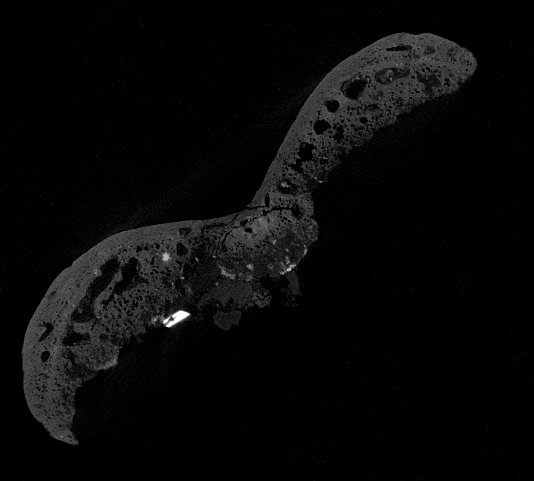

Supplement: S1 File — (ZIP) [file pone.0228610.s001.zip › 40_144/Br-11__IR_rec0722.jpg]

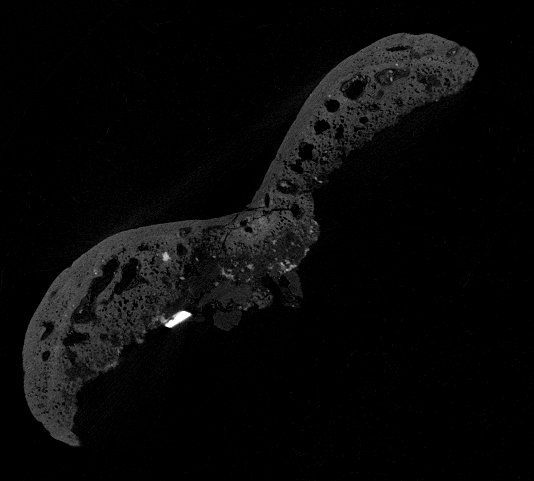

Supplement: S1 File — (ZIP) [file pone.0228610.s001.zip › 40_144/Br-11__IR_rec0726.jpg]

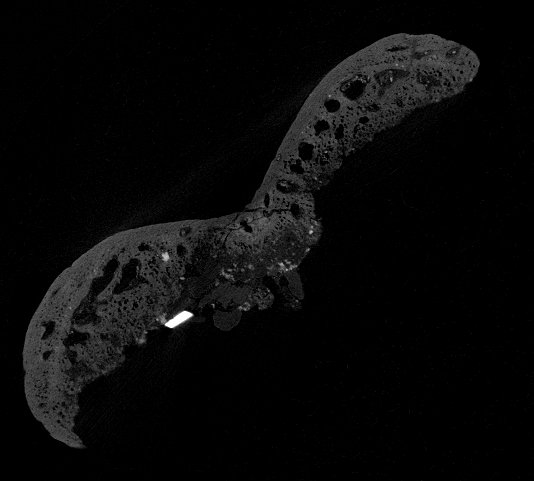

Supplement: S1 File — (ZIP) [file pone.0228610.s001.zip › 40_144/Br-11__IR_rec0730.jpg]

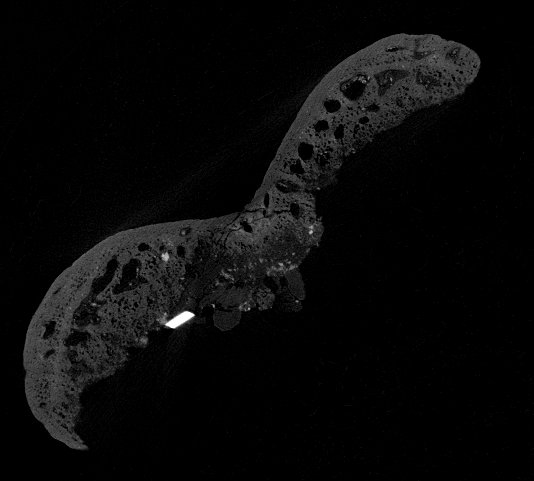

Supplement: S1 File — (ZIP) [file pone.0228610.s001.zip › 40_144/Br-11__IR_rec0734.jpg]

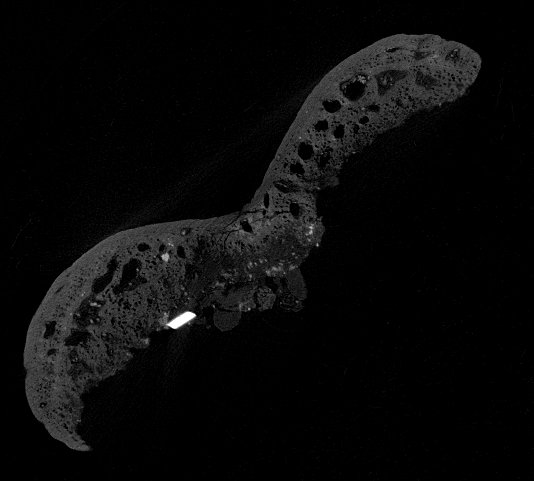

Supplement: S1 File — (ZIP) [file pone.0228610.s001.zip › 40_144/Br-11__IR_rec0738.jpg]

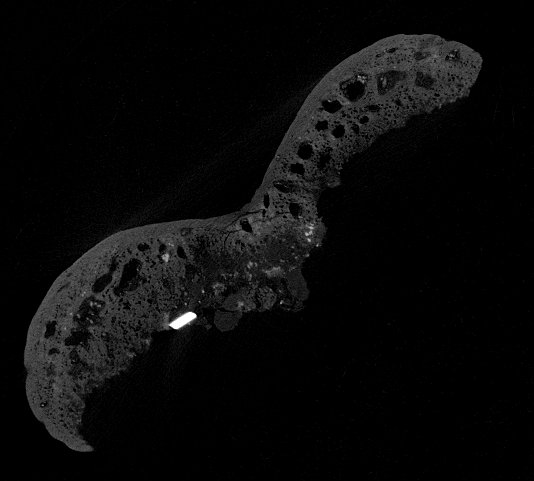

Supplement: S1 File — (ZIP) [file pone.0228610.s001.zip › 40_144/Br-11__IR_rec0742.jpg]
