# Supplementary figures and images for: A new small-sized stem salamander from the Middle Jurassic of Western Siberia, Russia (part 4 of 10)
Source: PLoS One. 2020 Feb 19;15(2):e0228610. doi: 10.1371/journal.pone.0228610 (PMC7029856; doi:10.1371/journal.pone.0228610)

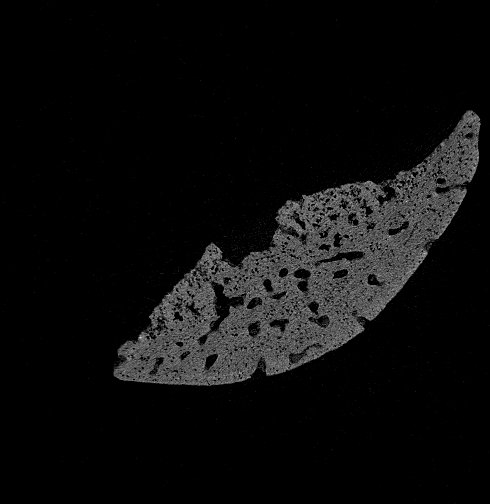

Supplement: S2 File — (ZIP) [file pone.0228610.s002.zip › 5_144/Br-16_IR_rec1063.jpg]

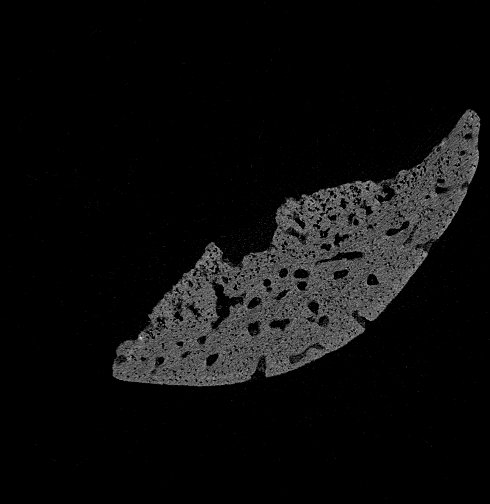

Supplement: S2 File — (ZIP) [file pone.0228610.s002.zip › 5_144/Br-16_IR_rec1067.jpg]

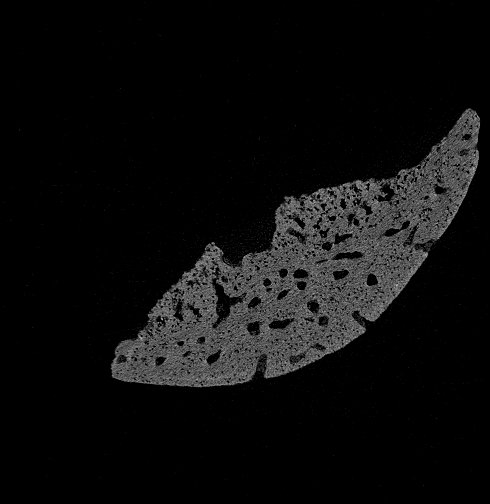

Supplement: S2 File — (ZIP) [file pone.0228610.s002.zip › 5_144/Br-16_IR_rec1071.jpg]

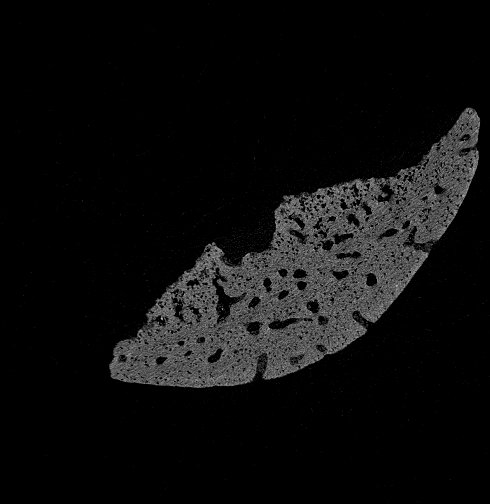

Supplement: S2 File — (ZIP) [file pone.0228610.s002.zip › 5_144/Br-16_IR_rec1075.jpg]

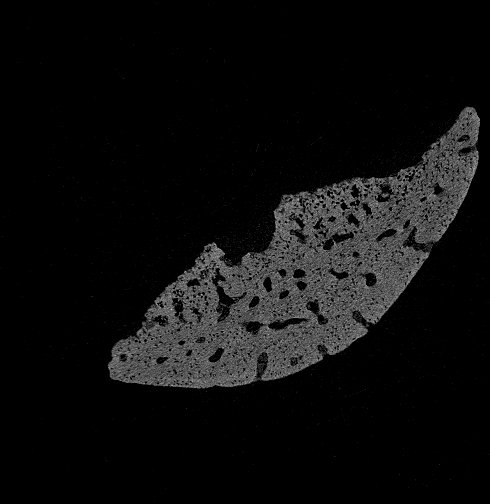

Supplement: S2 File — (ZIP) [file pone.0228610.s002.zip › 5_144/Br-16_IR_rec1079.jpg]

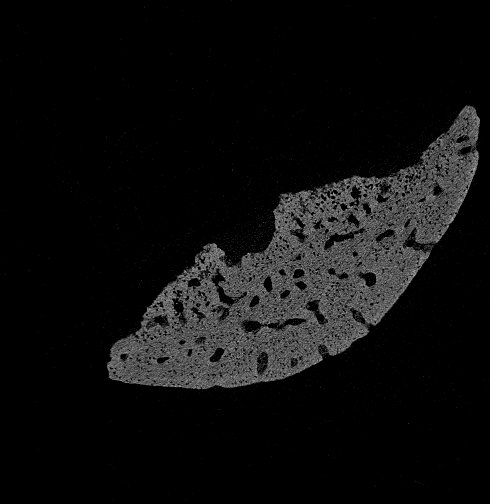

Supplement: S2 File — (ZIP) [file pone.0228610.s002.zip › 5_144/Br-16_IR_rec1083.jpg]

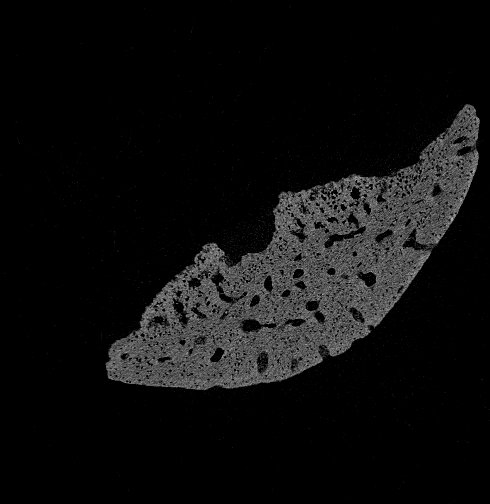

Supplement: S2 File — (ZIP) [file pone.0228610.s002.zip › 5_144/Br-16_IR_rec1087.jpg]

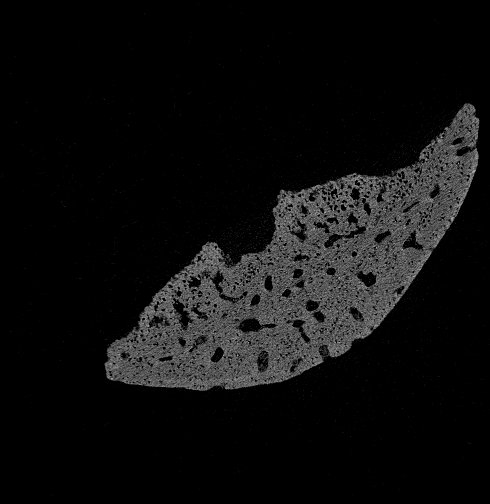

Supplement: S2 File — (ZIP) [file pone.0228610.s002.zip › 5_144/Br-16_IR_rec1091.jpg]

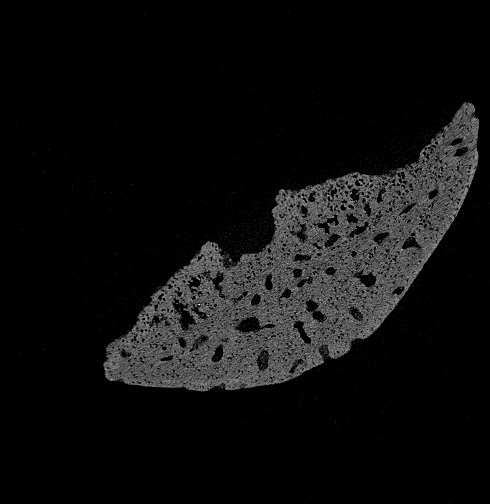

Supplement: S2 File — (ZIP) [file pone.0228610.s002.zip › 5_144/Br-16_IR_rec1095.jpg]

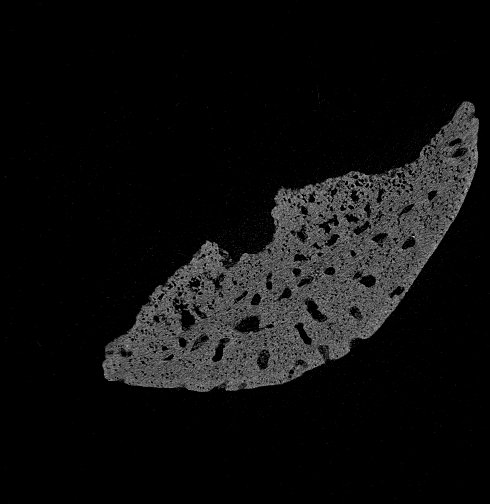

Supplement: S2 File — (ZIP) [file pone.0228610.s002.zip › 5_144/Br-16_IR_rec1099.jpg]

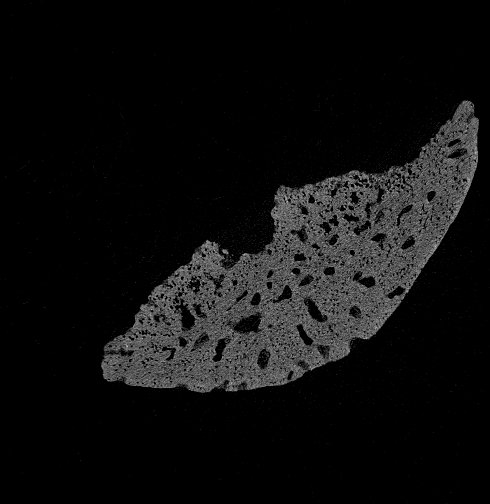

Supplement: S2 File — (ZIP) [file pone.0228610.s002.zip › 5_144/Br-16_IR_rec1103.jpg]

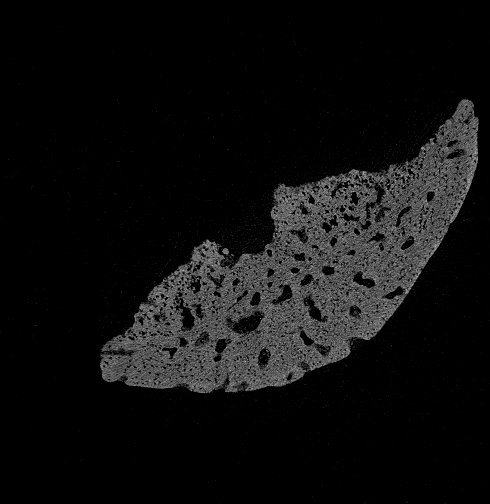

Supplement: S2 File — (ZIP) [file pone.0228610.s002.zip › 5_144/Br-16_IR_rec1107.jpg]

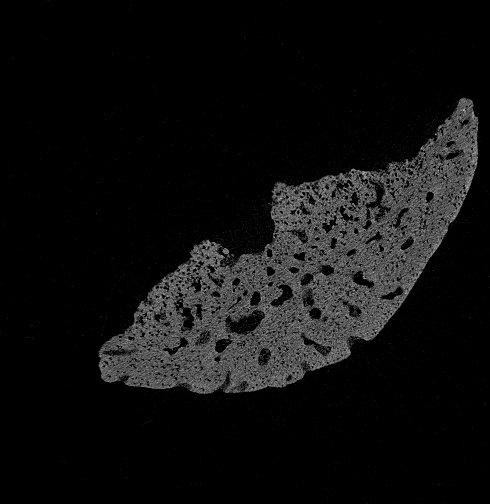

Supplement: S2 File — (ZIP) [file pone.0228610.s002.zip › 5_144/Br-16_IR_rec1111.jpg]

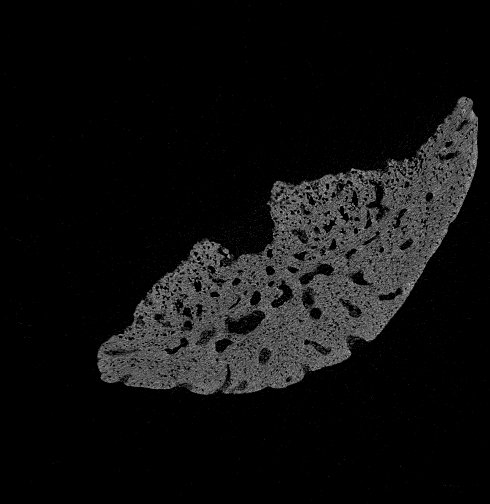

Supplement: S2 File — (ZIP) [file pone.0228610.s002.zip › 5_144/Br-16_IR_rec1115.jpg]

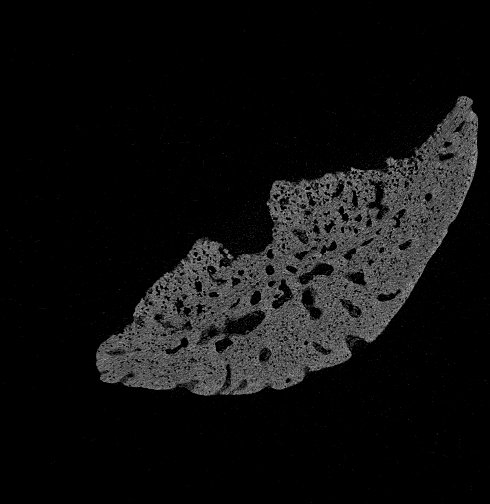

Supplement: S2 File — (ZIP) [file pone.0228610.s002.zip › 5_144/Br-16_IR_rec1119.jpg]

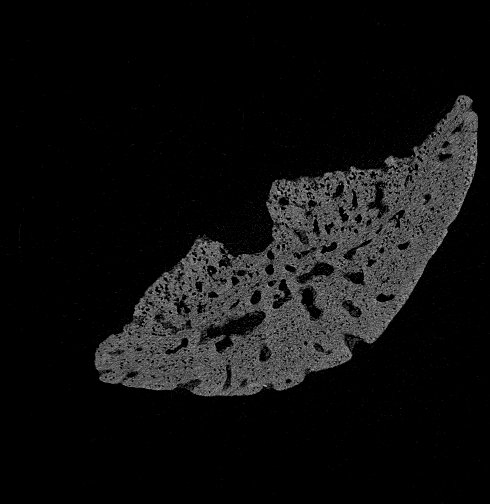

Supplement: S2 File — (ZIP) [file pone.0228610.s002.zip › 5_144/Br-16_IR_rec1123.jpg]

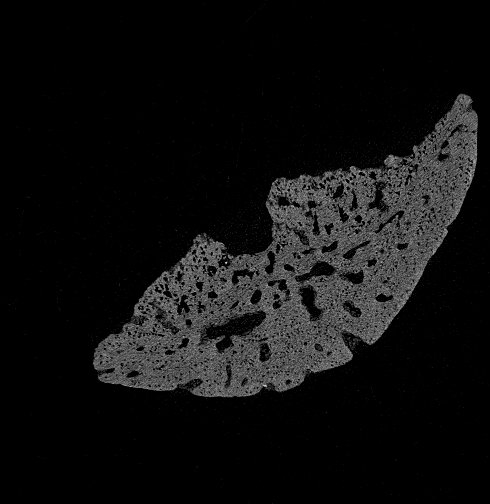

Supplement: S2 File — (ZIP) [file pone.0228610.s002.zip › 5_144/Br-16_IR_rec1127.jpg]

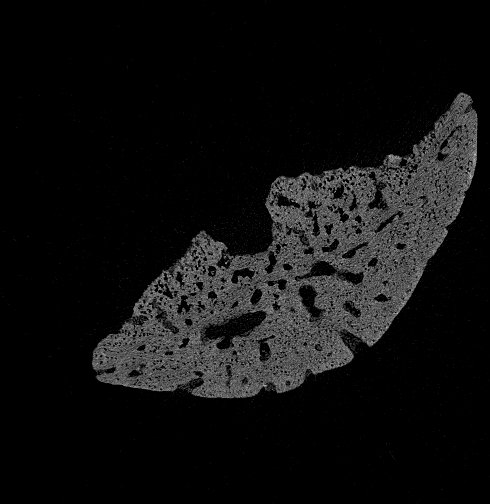

Supplement: S2 File — (ZIP) [file pone.0228610.s002.zip › 5_144/Br-16_IR_rec1131.jpg]

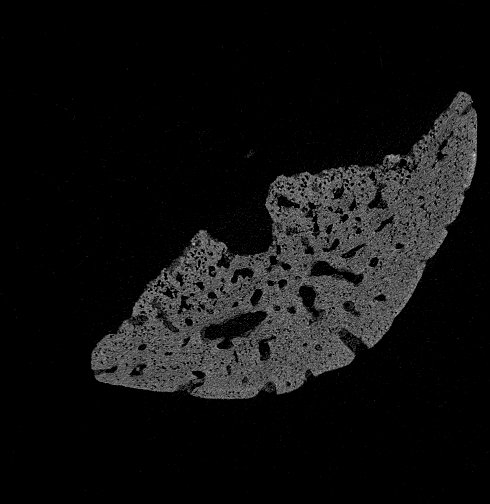

Supplement: S2 File — (ZIP) [file pone.0228610.s002.zip › 5_144/Br-16_IR_rec1135.jpg]

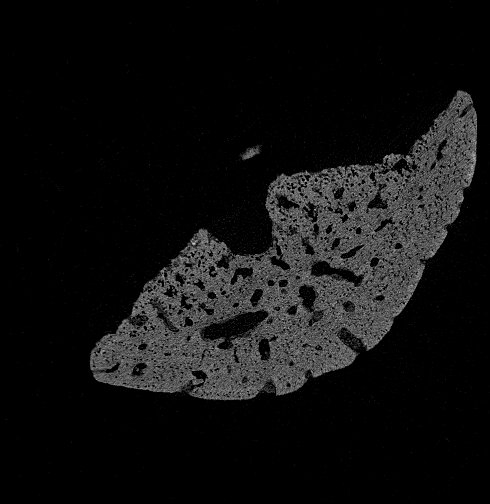

Supplement: S2 File — (ZIP) [file pone.0228610.s002.zip › 5_144/Br-16_IR_rec1139.jpg]

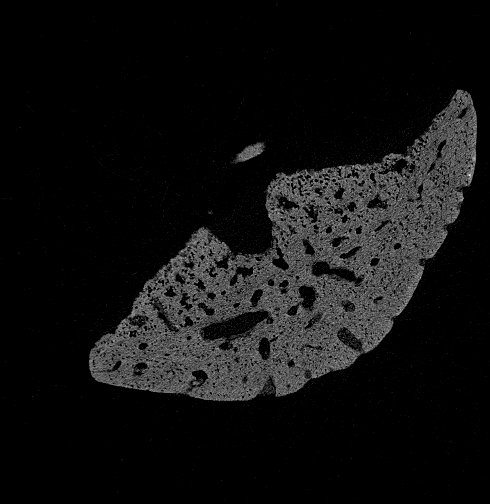

Supplement: S2 File — (ZIP) [file pone.0228610.s002.zip › 5_144/Br-16_IR_rec1143.jpg]

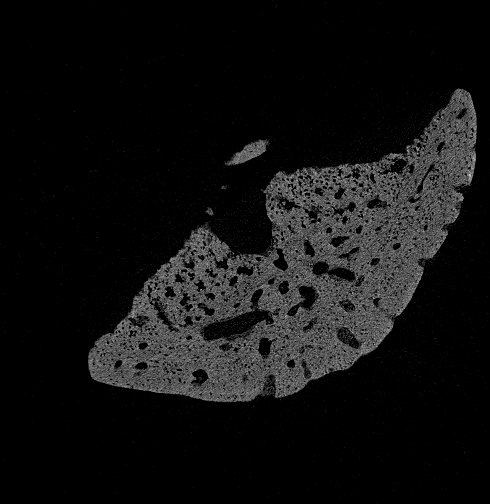

Supplement: S2 File — (ZIP) [file pone.0228610.s002.zip › 5_144/Br-16_IR_rec1147.jpg]

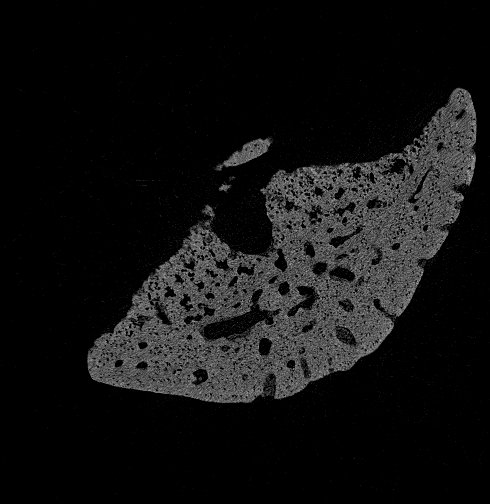

Supplement: S2 File — (ZIP) [file pone.0228610.s002.zip › 5_144/Br-16_IR_rec1151.jpg]

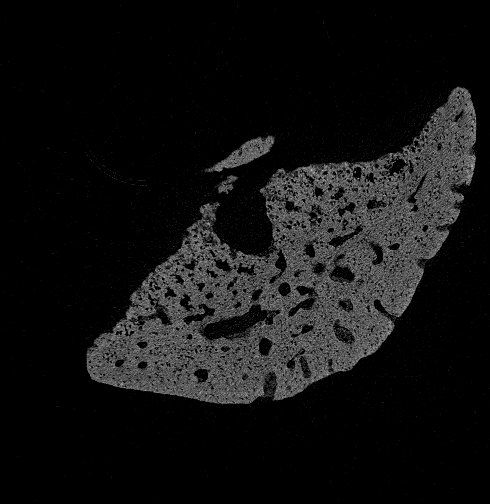

Supplement: S2 File — (ZIP) [file pone.0228610.s002.zip › 5_144/Br-16_IR_rec1155.jpg]

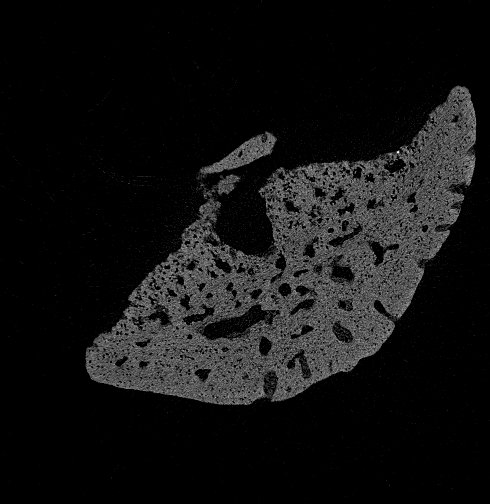

Supplement: S2 File — (ZIP) [file pone.0228610.s002.zip › 5_144/Br-16_IR_rec1159.jpg]

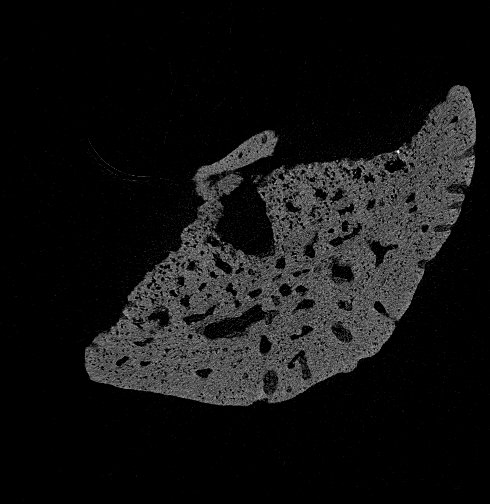

Supplement: S2 File — (ZIP) [file pone.0228610.s002.zip › 5_144/Br-16_IR_rec1163.jpg]

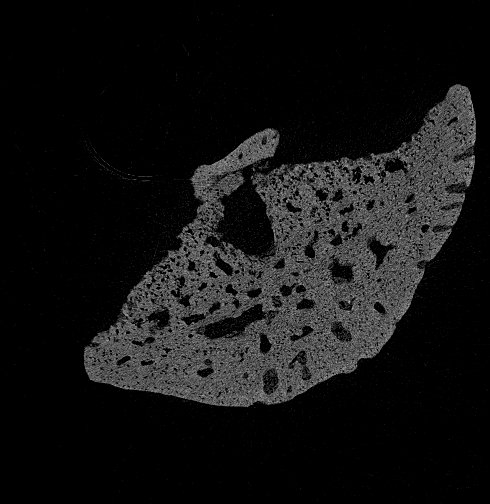

Supplement: S2 File — (ZIP) [file pone.0228610.s002.zip › 5_144/Br-16_IR_rec1167.jpg]

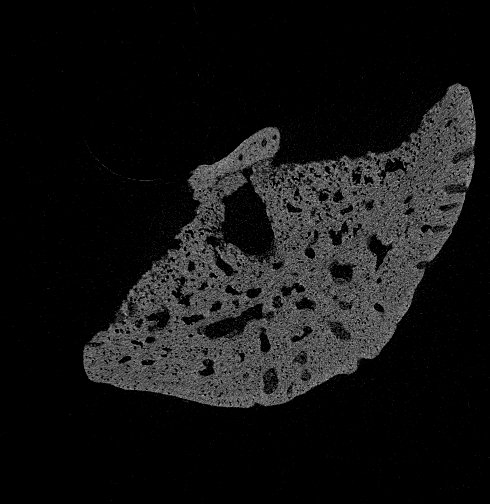

Supplement: S2 File — (ZIP) [file pone.0228610.s002.zip › 5_144/Br-16_IR_rec1171.jpg]

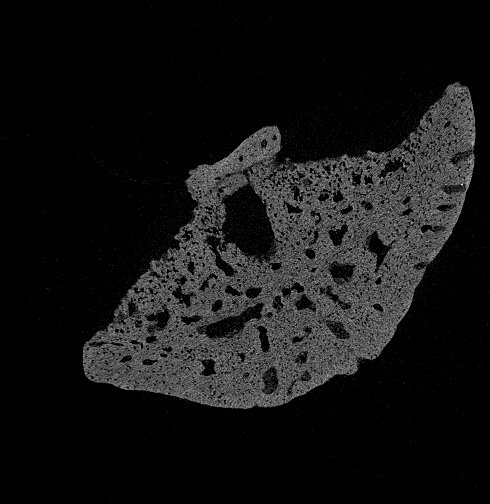

Supplement: S2 File — (ZIP) [file pone.0228610.s002.zip › 5_144/Br-16_IR_rec1175.jpg]

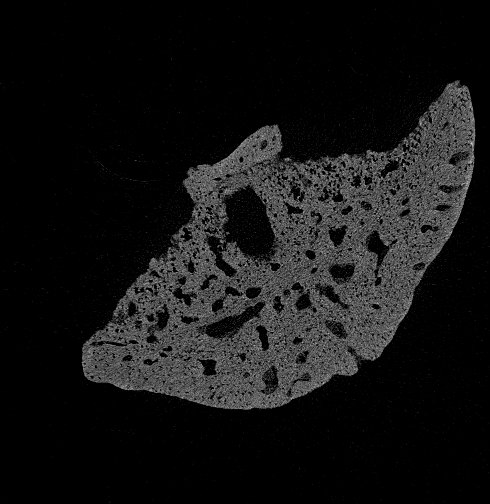

Supplement: S2 File — (ZIP) [file pone.0228610.s002.zip › 5_144/Br-16_IR_rec1179.jpg]

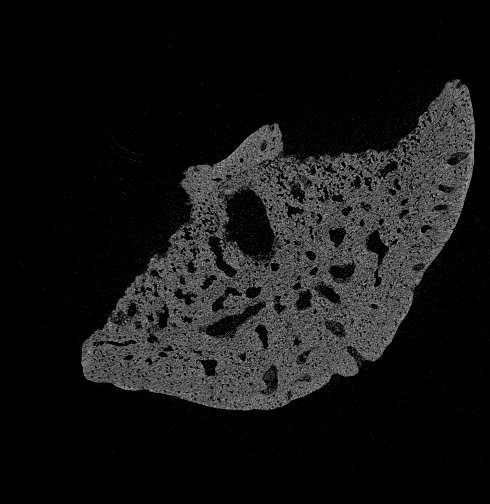

Supplement: S2 File — (ZIP) [file pone.0228610.s002.zip › 5_144/Br-16_IR_rec1183.jpg]

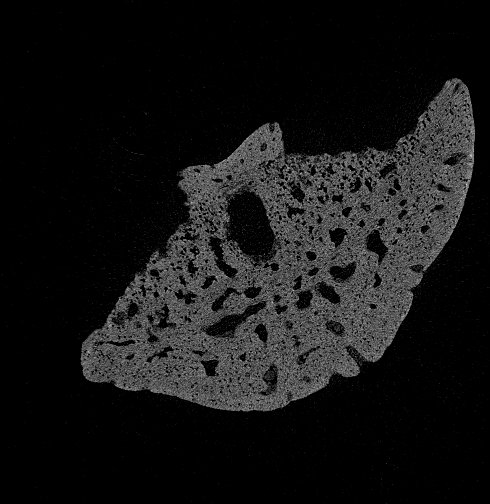

Supplement: S2 File — (ZIP) [file pone.0228610.s002.zip › 5_144/Br-16_IR_rec1187.jpg]

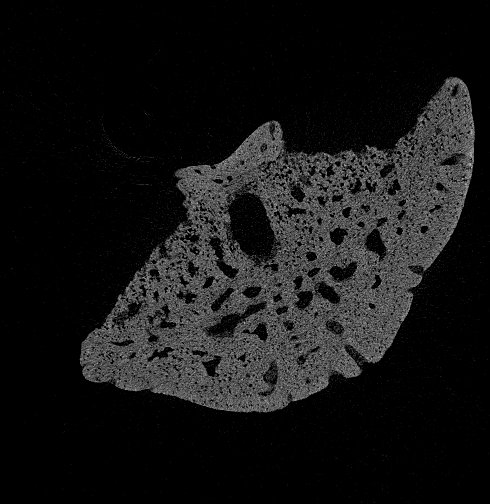

Supplement: S2 File — (ZIP) [file pone.0228610.s002.zip › 5_144/Br-16_IR_rec1191.jpg]

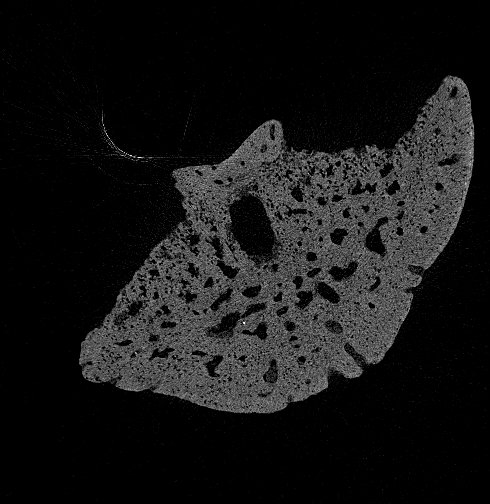

Supplement: S2 File — (ZIP) [file pone.0228610.s002.zip › 5_144/Br-16_IR_rec1195.jpg]

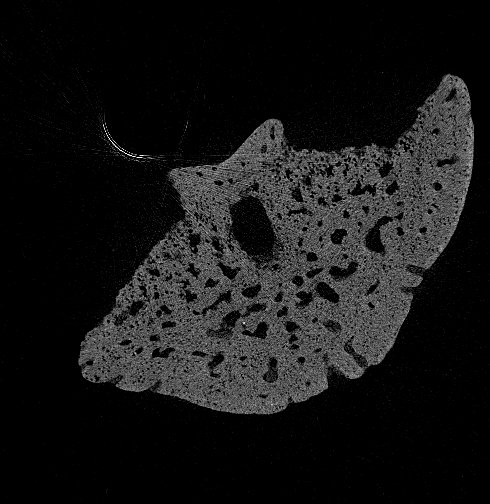

Supplement: S2 File — (ZIP) [file pone.0228610.s002.zip › 5_144/Br-16_IR_rec1199.jpg]

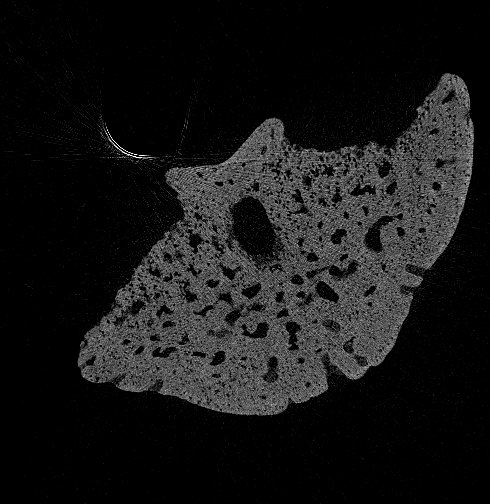

Supplement: S2 File — (ZIP) [file pone.0228610.s002.zip › 5_144/Br-16_IR_rec1203.jpg]

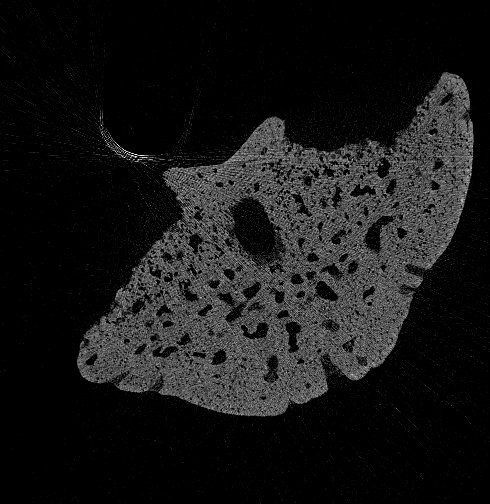

Supplement: S2 File — (ZIP) [file pone.0228610.s002.zip › 5_144/Br-16_IR_rec1207.jpg]

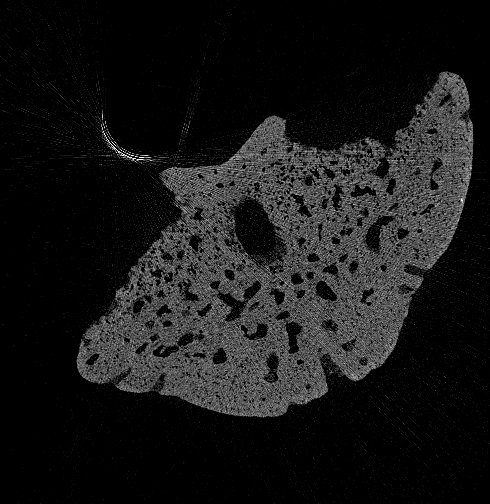

Supplement: S2 File — (ZIP) [file pone.0228610.s002.zip › 5_144/Br-16_IR_rec1211.jpg]

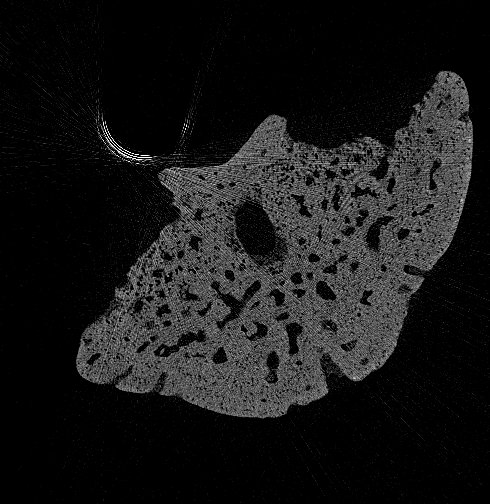

Supplement: S2 File — (ZIP) [file pone.0228610.s002.zip › 5_144/Br-16_IR_rec1215.jpg]

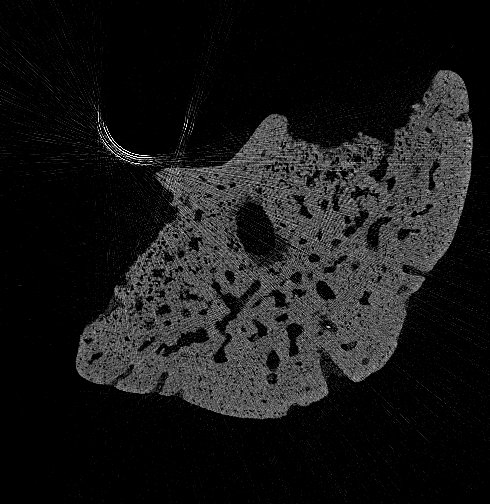

Supplement: S2 File — (ZIP) [file pone.0228610.s002.zip › 5_144/Br-16_IR_rec1219.jpg]

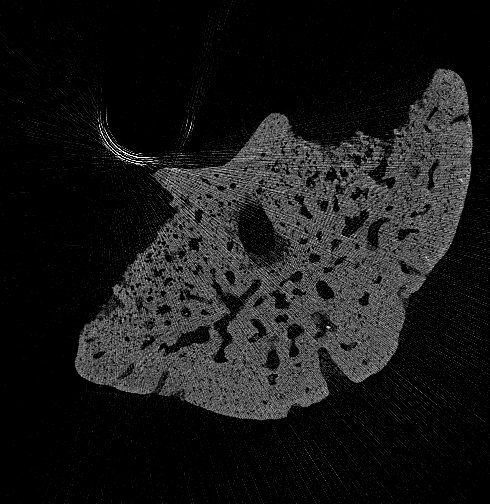

Supplement: S2 File — (ZIP) [file pone.0228610.s002.zip › 5_144/Br-16_IR_rec1223.jpg]

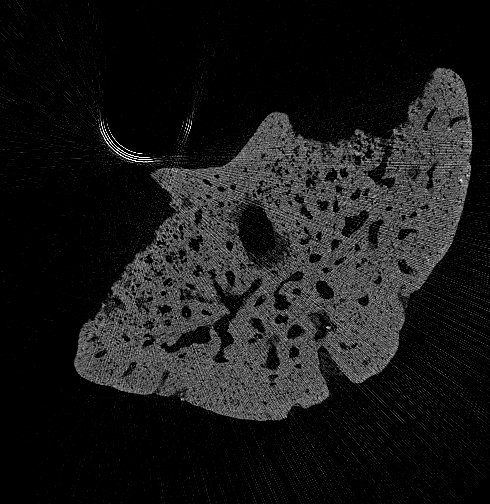

Supplement: S2 File — (ZIP) [file pone.0228610.s002.zip › 5_144/Br-16_IR_rec1227.jpg]

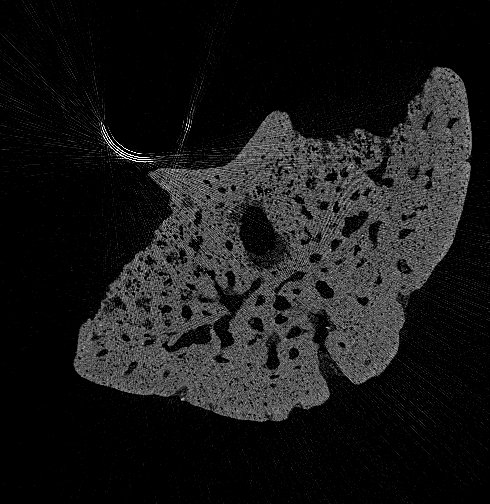

Supplement: S2 File — (ZIP) [file pone.0228610.s002.zip › 5_144/Br-16_IR_rec1231.jpg]

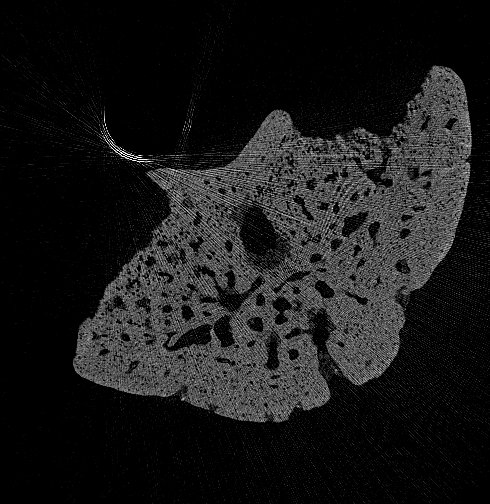

Supplement: S2 File — (ZIP) [file pone.0228610.s002.zip › 5_144/Br-16_IR_rec1235.jpg]

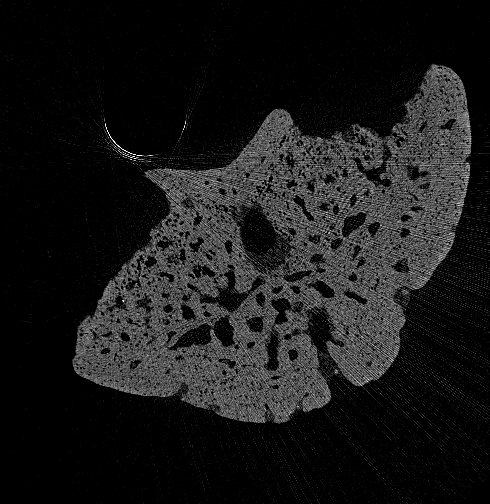

Supplement: S2 File — (ZIP) [file pone.0228610.s002.zip › 5_144/Br-16_IR_rec1239.jpg]

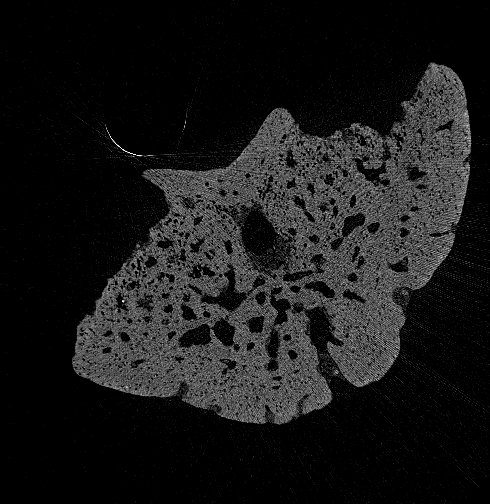

Supplement: S2 File — (ZIP) [file pone.0228610.s002.zip › 5_144/Br-16_IR_rec1243.jpg]

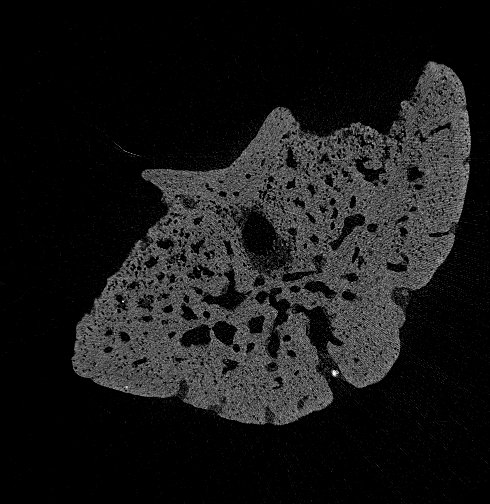

Supplement: S2 File — (ZIP) [file pone.0228610.s002.zip › 5_144/Br-16_IR_rec1247.jpg]

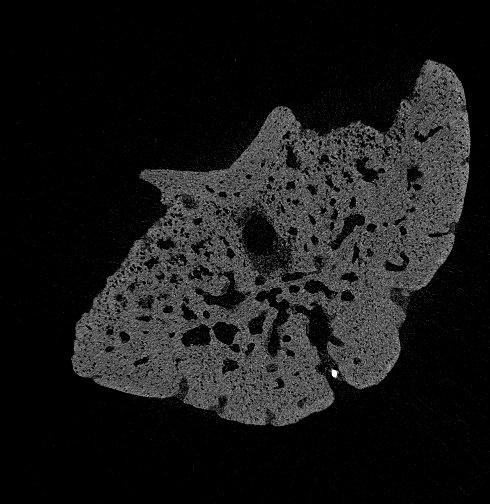

Supplement: S2 File — (ZIP) [file pone.0228610.s002.zip › 5_144/Br-16_IR_rec1251.jpg]

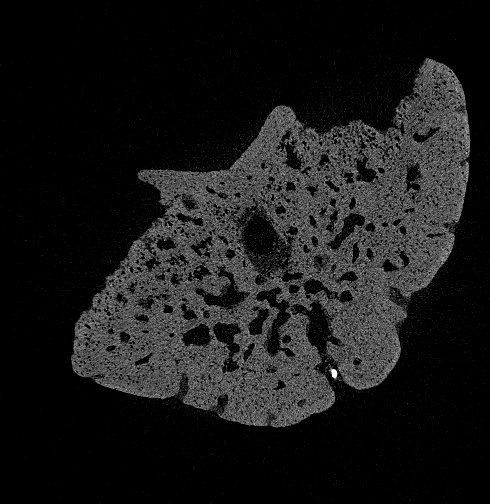

Supplement: S2 File — (ZIP) [file pone.0228610.s002.zip › 5_144/Br-16_IR_rec1255.jpg]

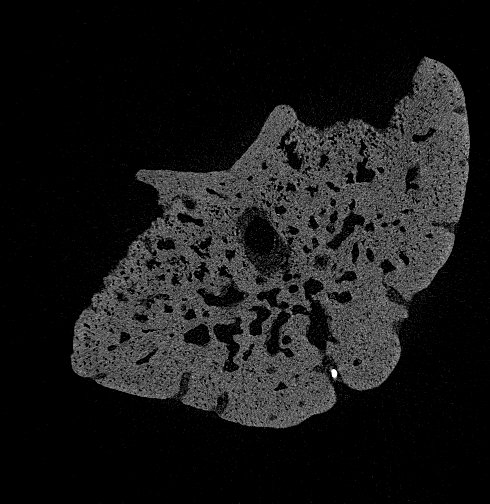

Supplement: S2 File — (ZIP) [file pone.0228610.s002.zip › 5_144/Br-16_IR_rec1259.jpg]

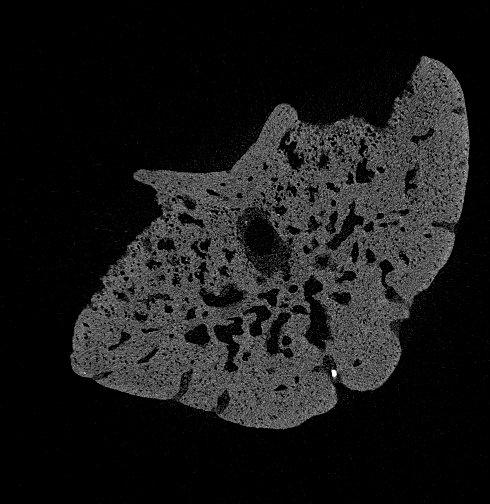

Supplement: S2 File — (ZIP) [file pone.0228610.s002.zip › 5_144/Br-16_IR_rec1263.jpg]

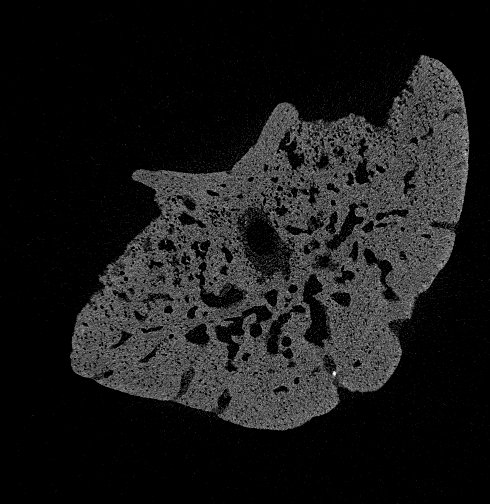

Supplement: S2 File — (ZIP) [file pone.0228610.s002.zip › 5_144/Br-16_IR_rec1267.jpg]

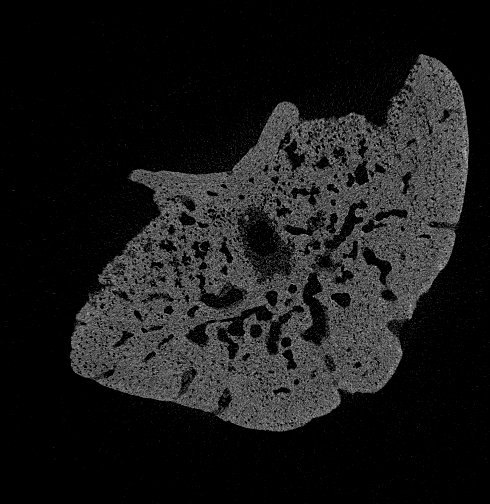

Supplement: S2 File — (ZIP) [file pone.0228610.s002.zip › 5_144/Br-16_IR_rec1271.jpg]

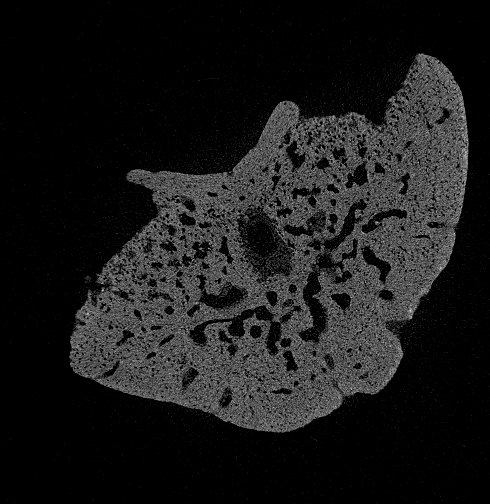

Supplement: S2 File — (ZIP) [file pone.0228610.s002.zip › 5_144/Br-16_IR_rec1275.jpg]

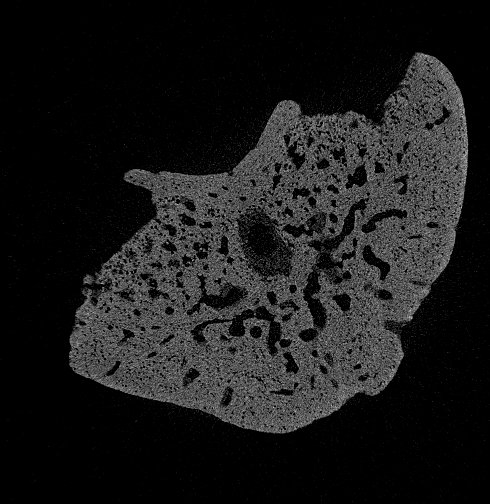

Supplement: S2 File — (ZIP) [file pone.0228610.s002.zip › 5_144/Br-16_IR_rec1279.jpg]

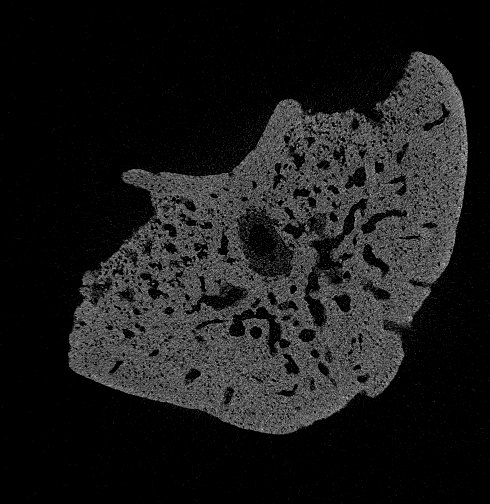

Supplement: S2 File — (ZIP) [file pone.0228610.s002.zip › 5_144/Br-16_IR_rec1283.jpg]

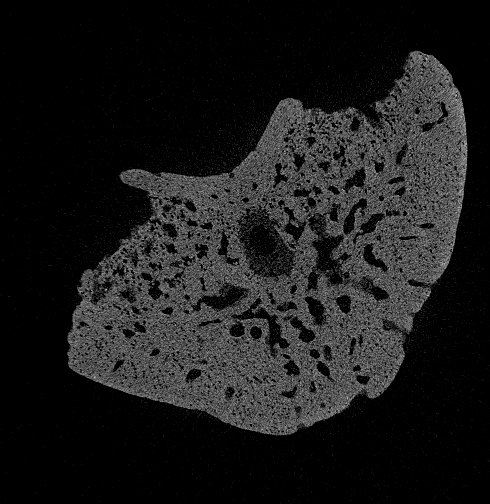

Supplement: S2 File — (ZIP) [file pone.0228610.s002.zip › 5_144/Br-16_IR_rec1287.jpg]

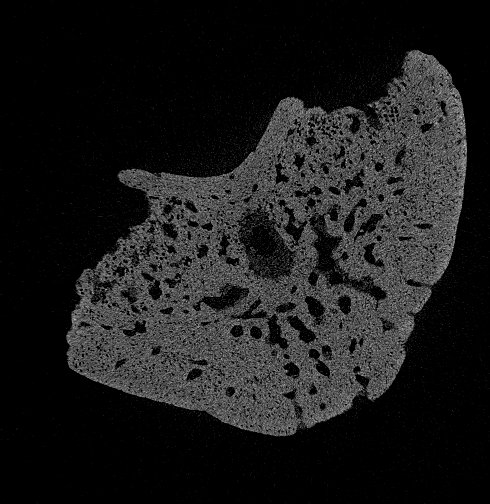

Supplement: S2 File — (ZIP) [file pone.0228610.s002.zip › 5_144/Br-16_IR_rec1291.jpg]

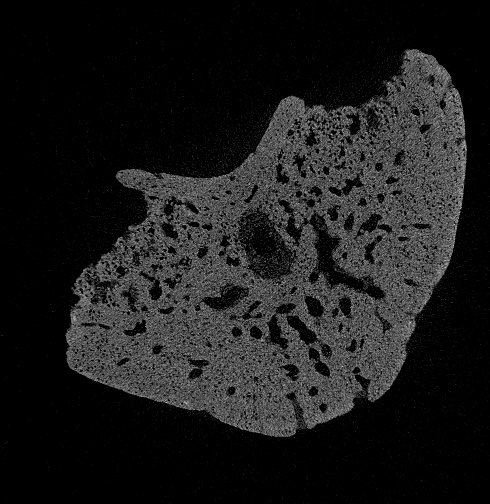

Supplement: S2 File — (ZIP) [file pone.0228610.s002.zip › 5_144/Br-16_IR_rec1295.jpg]

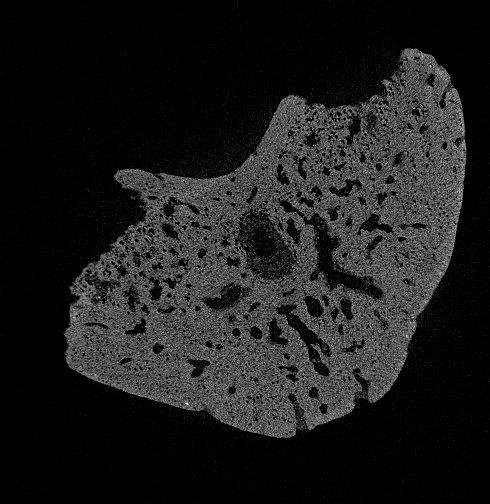

Supplement: S2 File — (ZIP) [file pone.0228610.s002.zip › 5_144/Br-16_IR_rec1299.jpg]

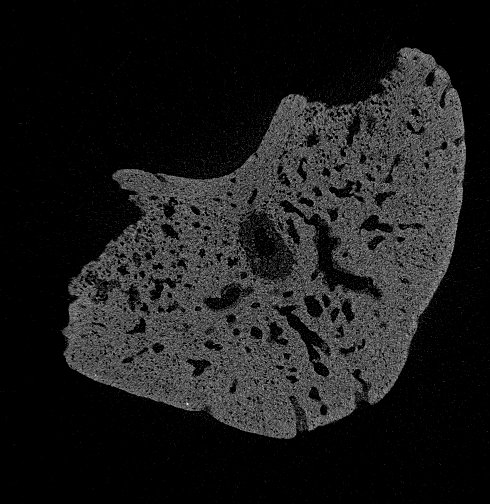

Supplement: S2 File — (ZIP) [file pone.0228610.s002.zip › 5_144/Br-16_IR_rec1303.jpg]

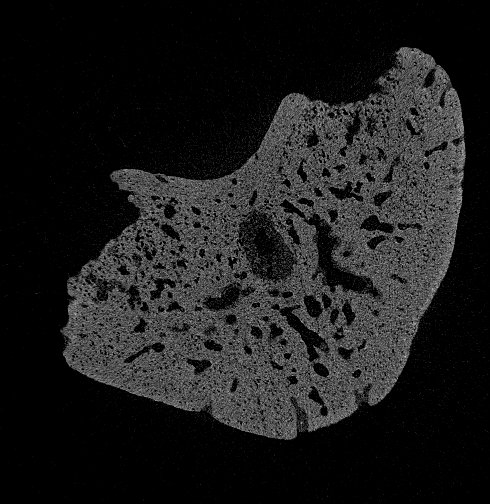

Supplement: S2 File — (ZIP) [file pone.0228610.s002.zip › 5_144/Br-16_IR_rec1307.jpg]

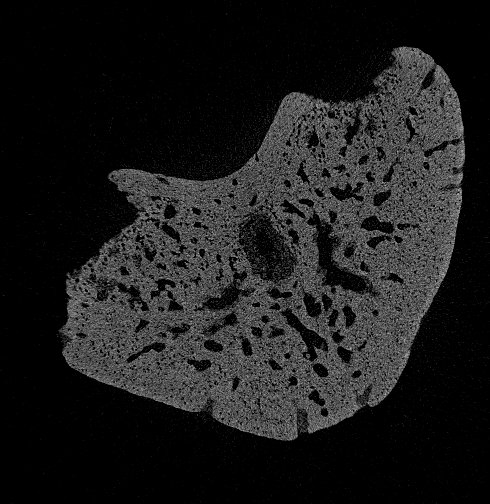

Supplement: S2 File — (ZIP) [file pone.0228610.s002.zip › 5_144/Br-16_IR_rec1311.jpg]

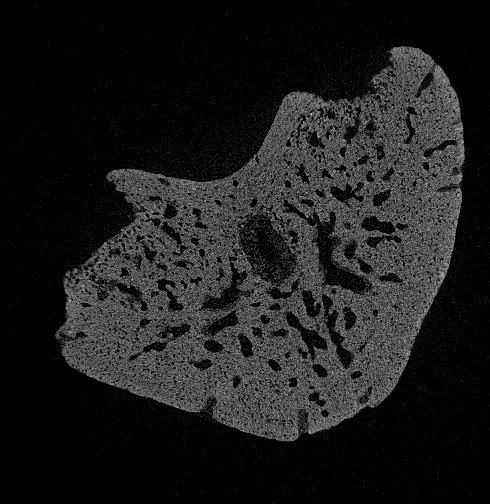

Supplement: S2 File — (ZIP) [file pone.0228610.s002.zip › 5_144/Br-16_IR_rec1315.jpg]

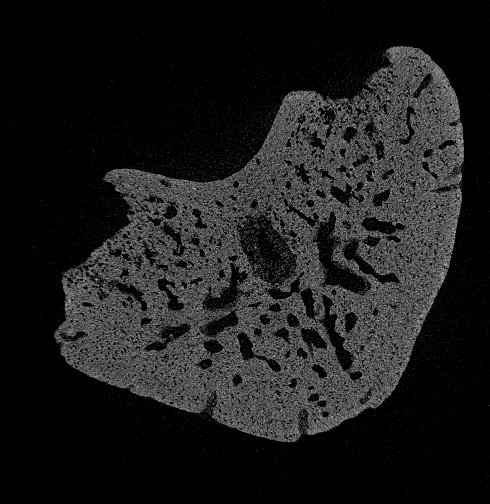

Supplement: S2 File — (ZIP) [file pone.0228610.s002.zip › 5_144/Br-16_IR_rec1319.jpg]

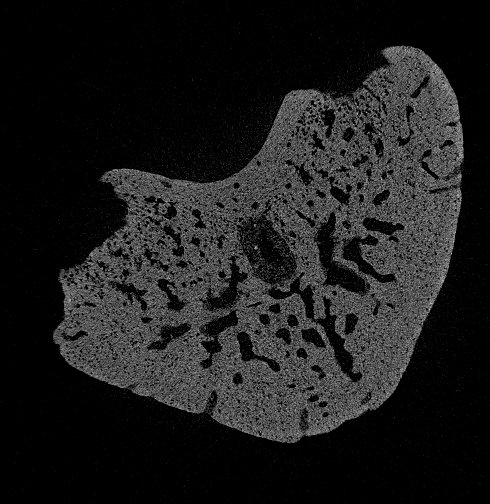

Supplement: S2 File — (ZIP) [file pone.0228610.s002.zip › 5_144/Br-16_IR_rec1323.jpg]

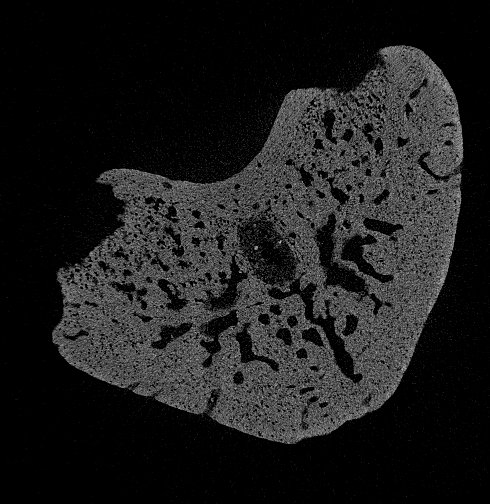

Supplement: S2 File — (ZIP) [file pone.0228610.s002.zip › 5_144/Br-16_IR_rec1327.jpg]

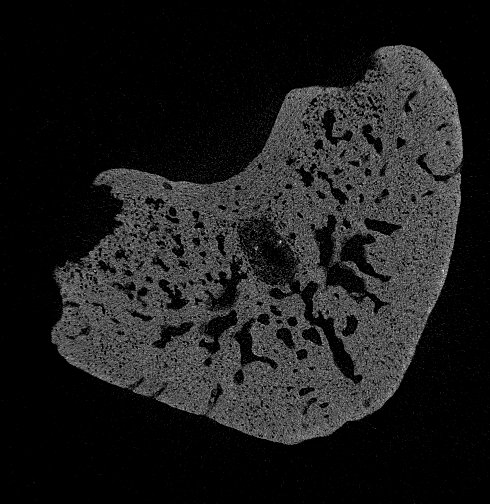

Supplement: S2 File — (ZIP) [file pone.0228610.s002.zip › 5_144/Br-16_IR_rec1331.jpg]

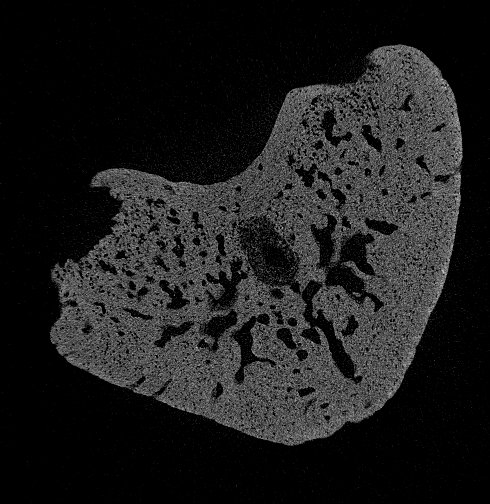

Supplement: S2 File — (ZIP) [file pone.0228610.s002.zip › 5_144/Br-16_IR_rec1335.jpg]

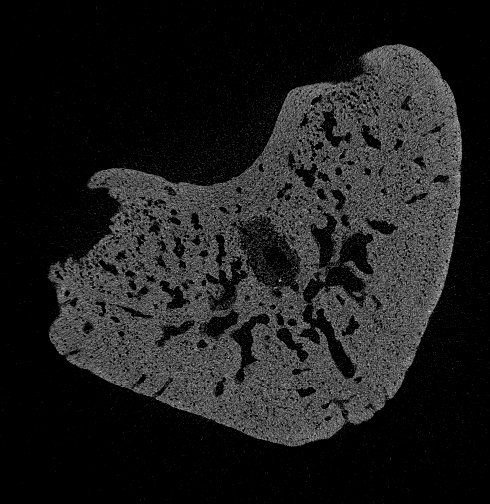

Supplement: S2 File — (ZIP) [file pone.0228610.s002.zip › 5_144/Br-16_IR_rec1339.jpg]

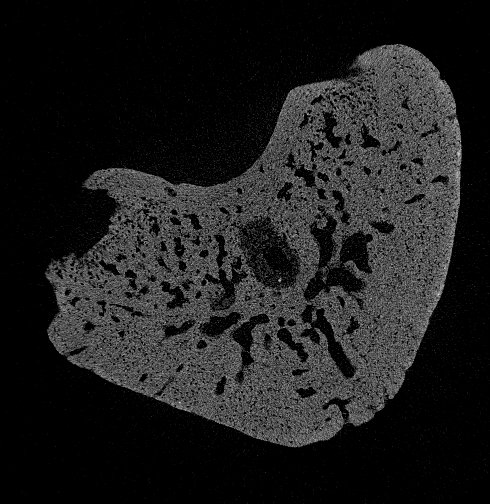

Supplement: S2 File — (ZIP) [file pone.0228610.s002.zip › 5_144/Br-16_IR_rec1343.jpg]

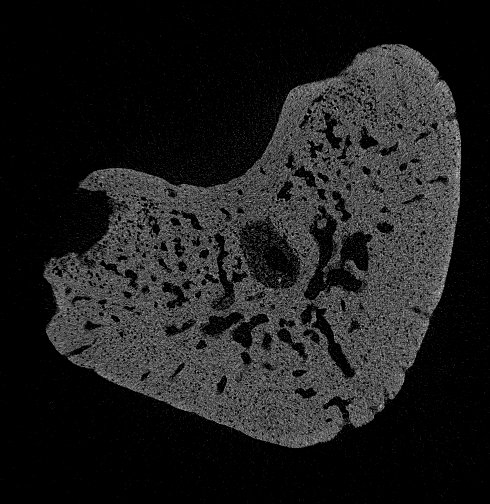

Supplement: S2 File — (ZIP) [file pone.0228610.s002.zip › 5_144/Br-16_IR_rec1347.jpg]

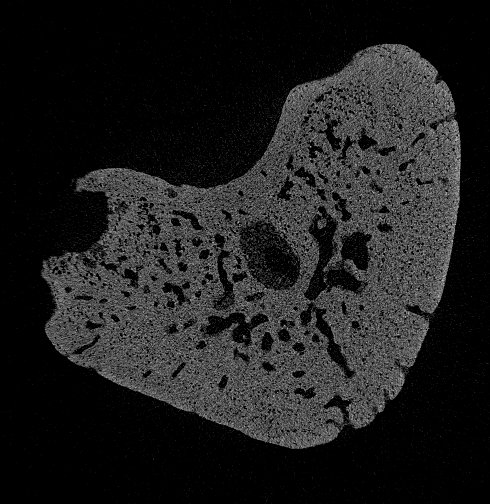

Supplement: S2 File — (ZIP) [file pone.0228610.s002.zip › 5_144/Br-16_IR_rec1351.jpg]

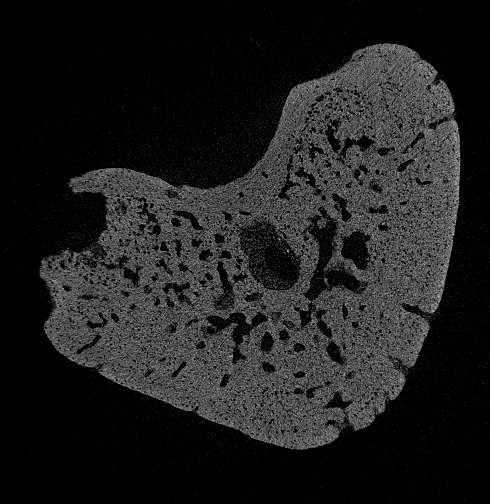

Supplement: S2 File — (ZIP) [file pone.0228610.s002.zip › 5_144/Br-16_IR_rec1355.jpg]

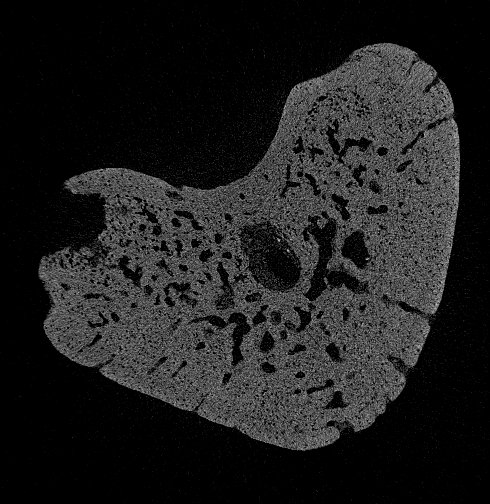

Supplement: S2 File — (ZIP) [file pone.0228610.s002.zip › 5_144/Br-16_IR_rec1359.jpg]

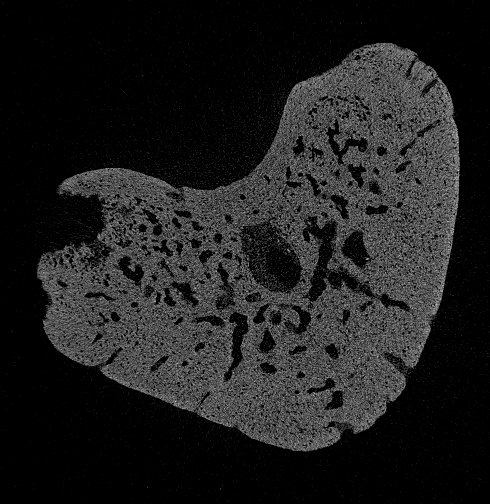

Supplement: S2 File — (ZIP) [file pone.0228610.s002.zip › 5_144/Br-16_IR_rec1363.jpg]

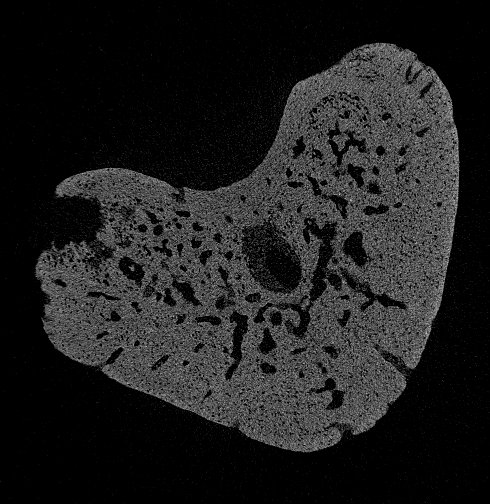

Supplement: S2 File — (ZIP) [file pone.0228610.s002.zip › 5_144/Br-16_IR_rec1367.jpg]

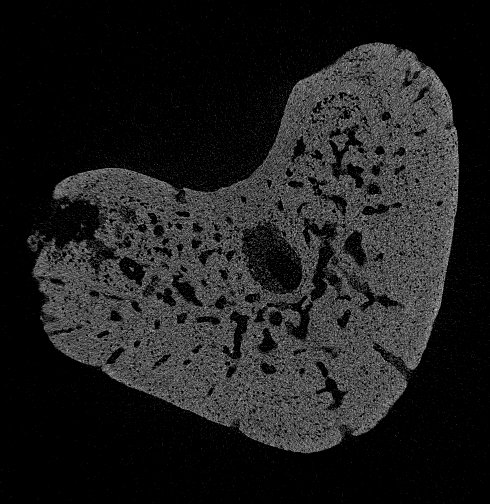

Supplement: S2 File — (ZIP) [file pone.0228610.s002.zip › 5_144/Br-16_IR_rec1371.jpg]

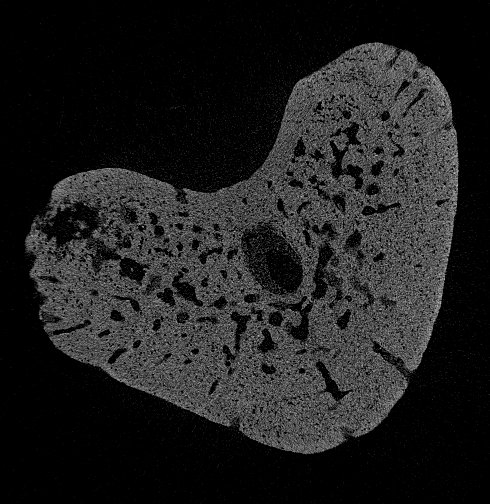

Supplement: S2 File — (ZIP) [file pone.0228610.s002.zip › 5_144/Br-16_IR_rec1375.jpg]

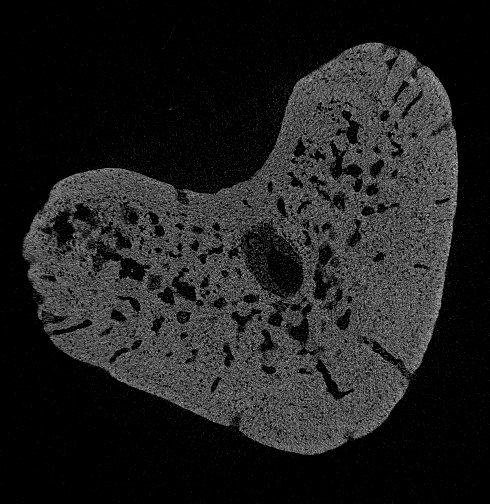

Supplement: S2 File — (ZIP) [file pone.0228610.s002.zip › 5_144/Br-16_IR_rec1379.jpg]

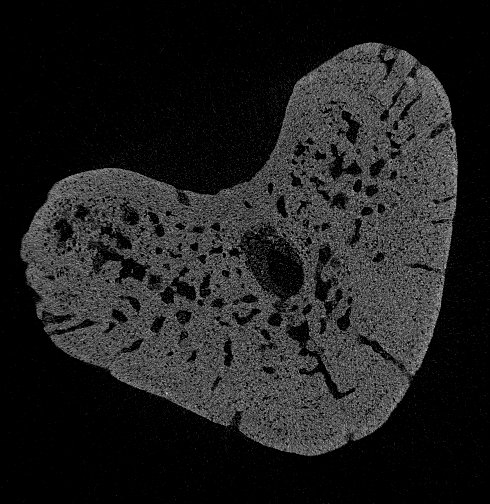

Supplement: S2 File — (ZIP) [file pone.0228610.s002.zip › 5_144/Br-16_IR_rec1383.jpg]

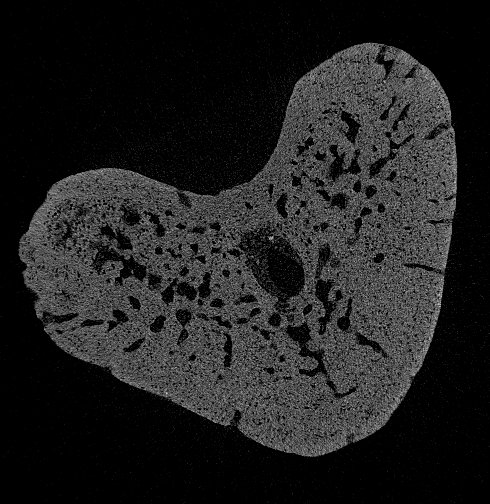

Supplement: S2 File — (ZIP) [file pone.0228610.s002.zip › 5_144/Br-16_IR_rec1387.jpg]

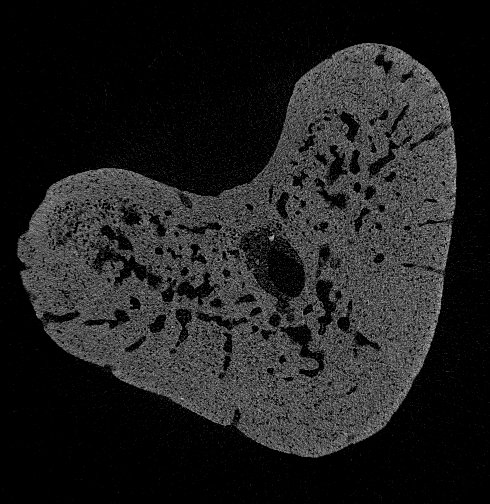

Supplement: S2 File — (ZIP) [file pone.0228610.s002.zip › 5_144/Br-16_IR_rec1391.jpg]

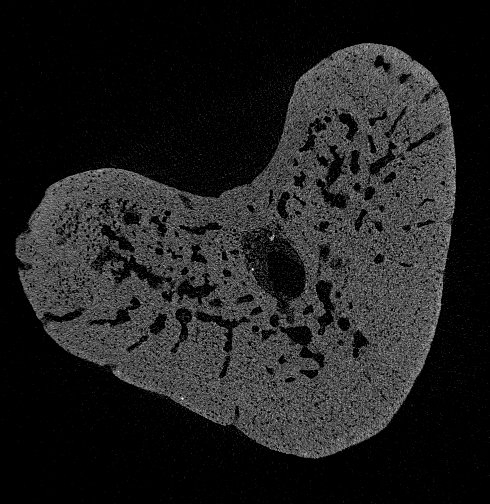

Supplement: S2 File — (ZIP) [file pone.0228610.s002.zip › 5_144/Br-16_IR_rec1395.jpg]

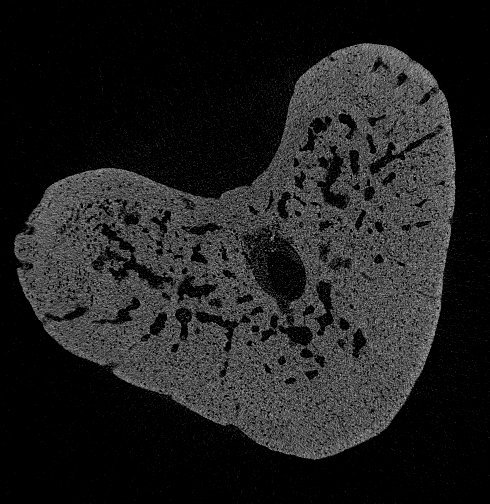

Supplement: S2 File — (ZIP) [file pone.0228610.s002.zip › 5_144/Br-16_IR_rec1399.jpg]

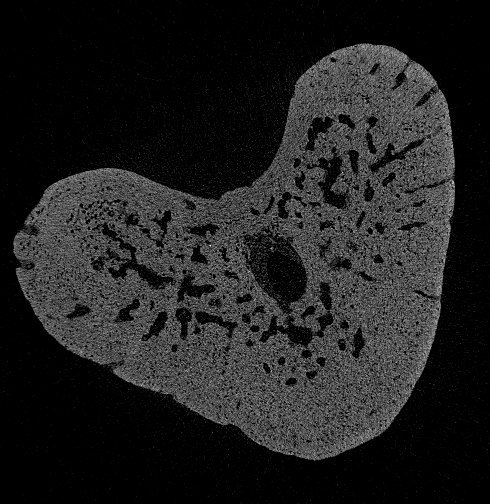

Supplement: S2 File — (ZIP) [file pone.0228610.s002.zip › 5_144/Br-16_IR_rec1403.jpg]

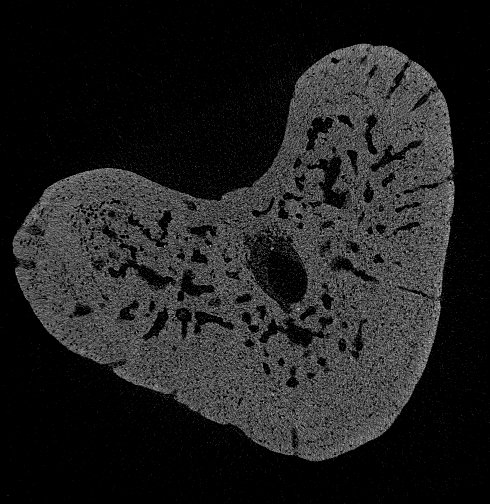

Supplement: S2 File — (ZIP) [file pone.0228610.s002.zip › 5_144/Br-16_IR_rec1407.jpg]

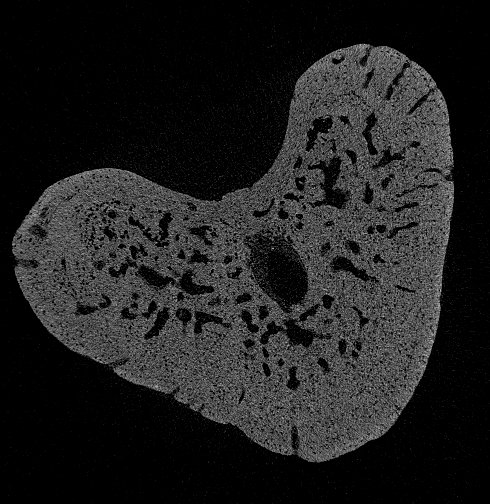

Supplement: S2 File — (ZIP) [file pone.0228610.s002.zip › 5_144/Br-16_IR_rec1411.jpg]

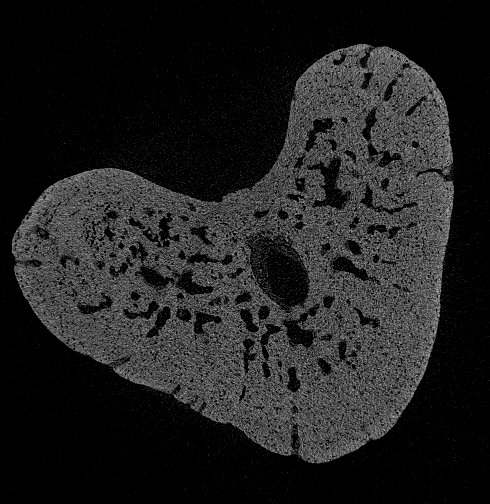

Supplement: S2 File — (ZIP) [file pone.0228610.s002.zip › 5_144/Br-16_IR_rec1415.jpg]

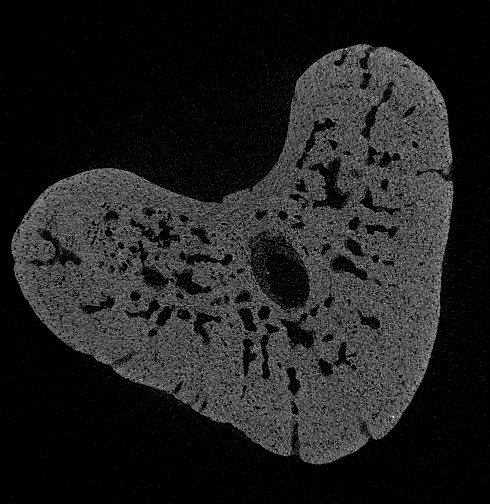

Supplement: S2 File — (ZIP) [file pone.0228610.s002.zip › 5_144/Br-16_IR_rec1419.jpg]

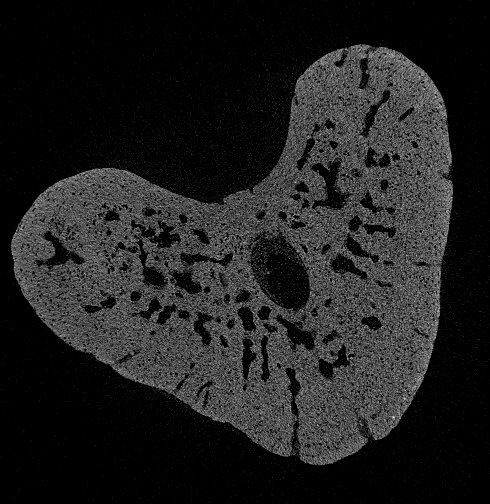

Supplement: S2 File — (ZIP) [file pone.0228610.s002.zip › 5_144/Br-16_IR_rec1423.jpg]

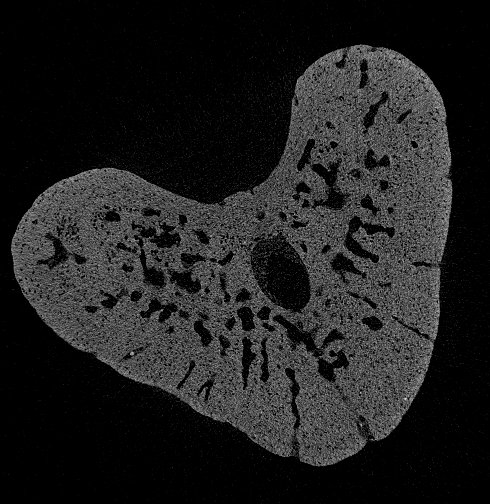

Supplement: S2 File — (ZIP) [file pone.0228610.s002.zip › 5_144/Br-16_IR_rec1427.jpg]

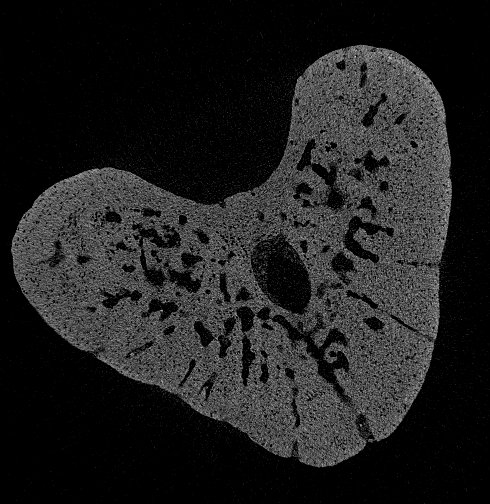

Supplement: S2 File — (ZIP) [file pone.0228610.s002.zip › 5_144/Br-16_IR_rec1431.jpg]

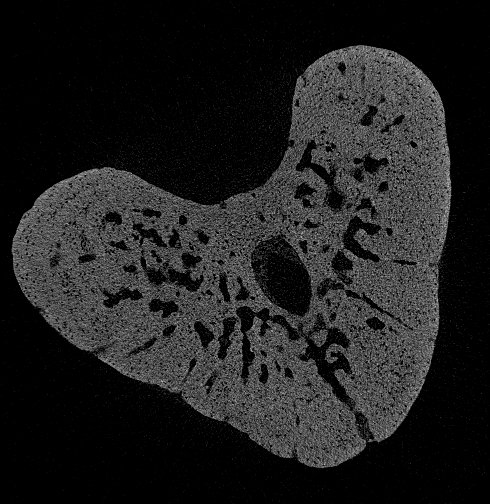

Supplement: S2 File — (ZIP) [file pone.0228610.s002.zip › 5_144/Br-16_IR_rec1435.jpg]

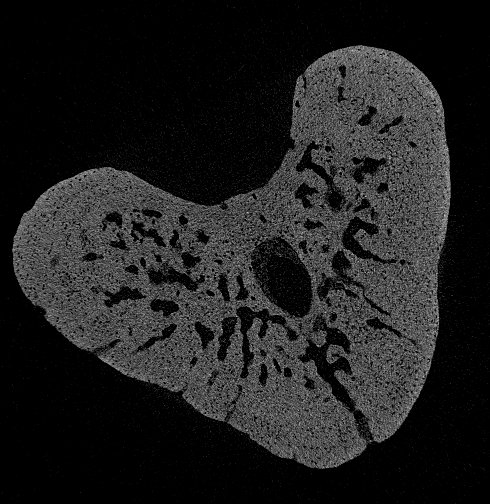

Supplement: S2 File — (ZIP) [file pone.0228610.s002.zip › 5_144/Br-16_IR_rec1439.jpg]

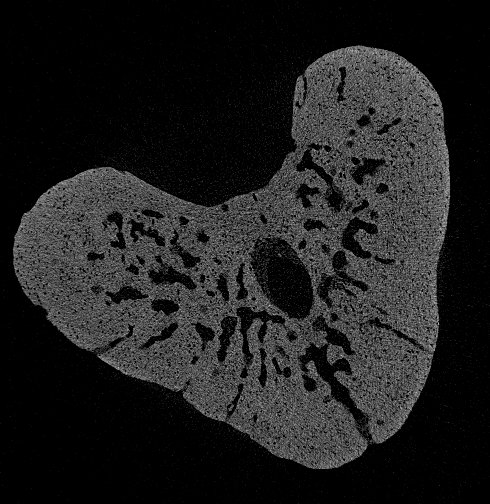

Supplement: S2 File — (ZIP) [file pone.0228610.s002.zip › 5_144/Br-16_IR_rec1443.jpg]

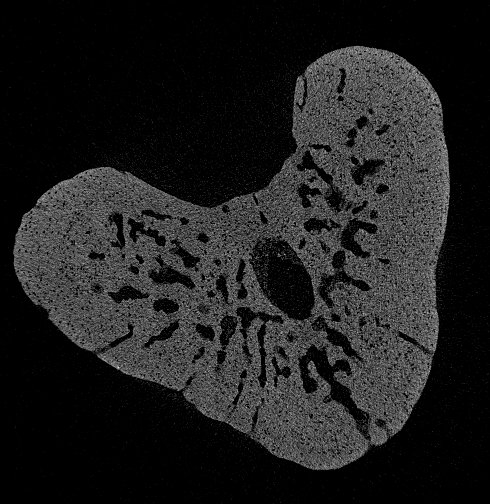

Supplement: S2 File — (ZIP) [file pone.0228610.s002.zip › 5_144/Br-16_IR_rec1447.jpg]

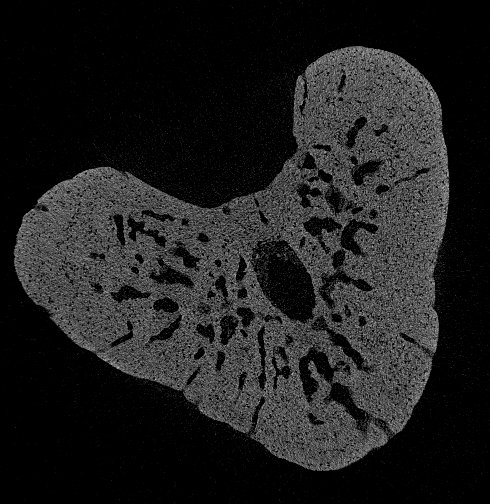

Supplement: S2 File — (ZIP) [file pone.0228610.s002.zip › 5_144/Br-16_IR_rec1451.jpg]

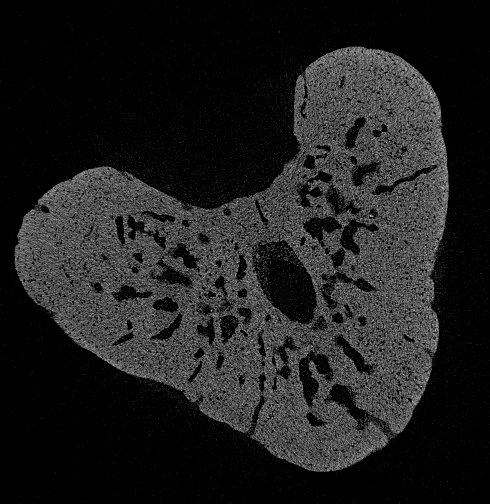

Supplement: S2 File — (ZIP) [file pone.0228610.s002.zip › 5_144/Br-16_IR_rec1455.jpg]

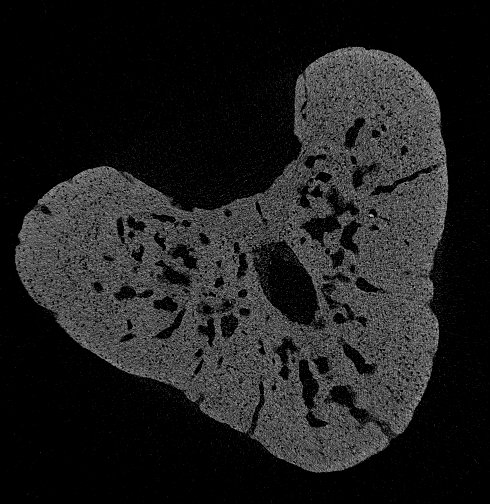

Supplement: S2 File — (ZIP) [file pone.0228610.s002.zip › 5_144/Br-16_IR_rec1459.jpg]
