# Supplementary figures and images for: A new small-sized stem salamander from the Middle Jurassic of Western Siberia, Russia (part 3 of 10)
Source: PLoS One. 2020 Feb 19;15(2):e0228610. doi: 10.1371/journal.pone.0228610 (PMC7029856; doi:10.1371/journal.pone.0228610)

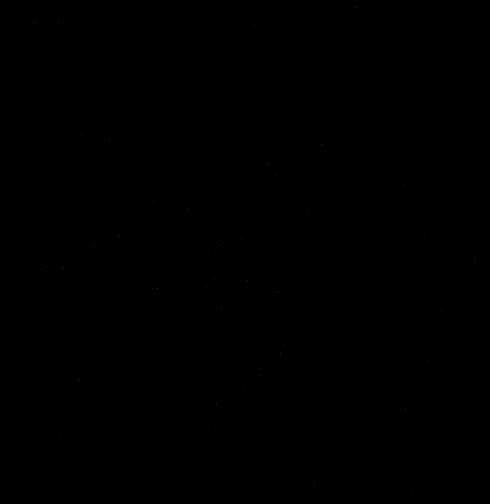

Supplement: S2 File — (ZIP) [file pone.0228610.s002.zip › 5_144/Br-16_IR_rec0663.jpg]

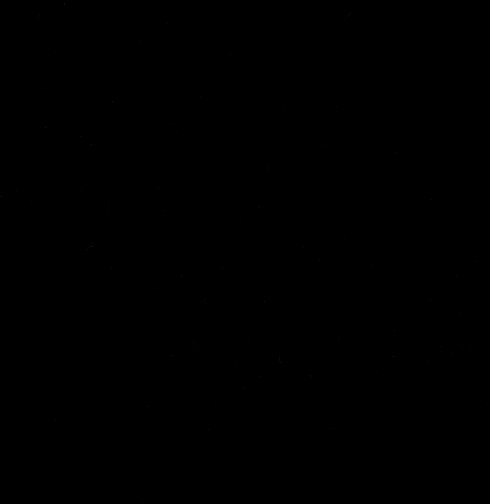

Supplement: S2 File — (ZIP) [file pone.0228610.s002.zip › 5_144/Br-16_IR_rec0667.jpg]

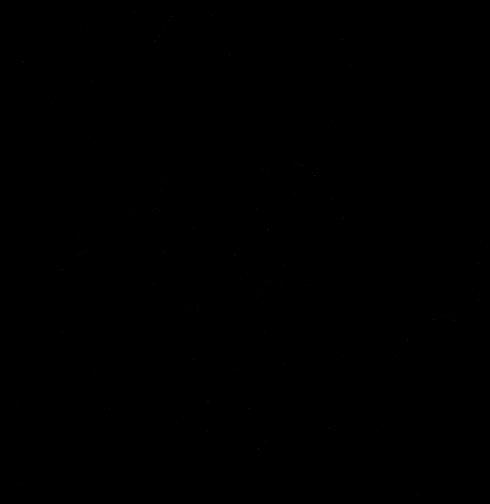

Supplement: S2 File — (ZIP) [file pone.0228610.s002.zip › 5_144/Br-16_IR_rec0671.jpg]

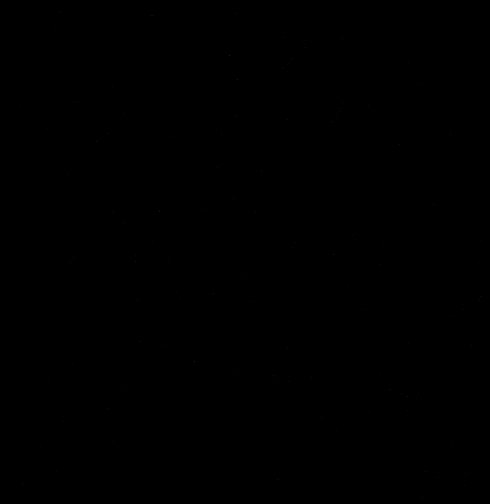

Supplement: S2 File — (ZIP) [file pone.0228610.s002.zip › 5_144/Br-16_IR_rec0675.jpg]

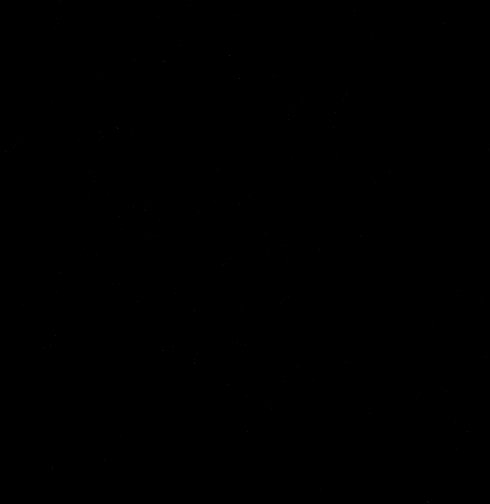

Supplement: S2 File — (ZIP) [file pone.0228610.s002.zip › 5_144/Br-16_IR_rec0679.jpg]

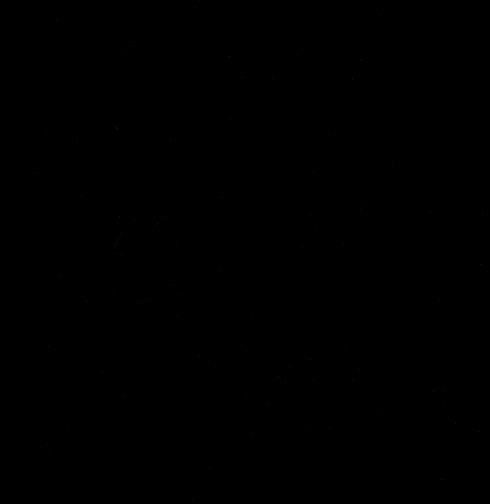

Supplement: S2 File — (ZIP) [file pone.0228610.s002.zip › 5_144/Br-16_IR_rec0683.jpg]

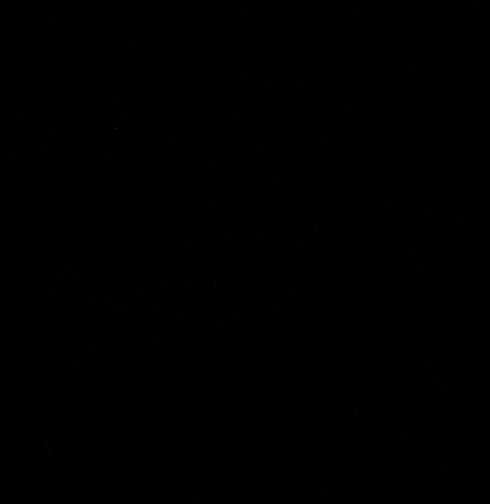

Supplement: S2 File — (ZIP) [file pone.0228610.s002.zip › 5_144/Br-16_IR_rec0687.jpg]

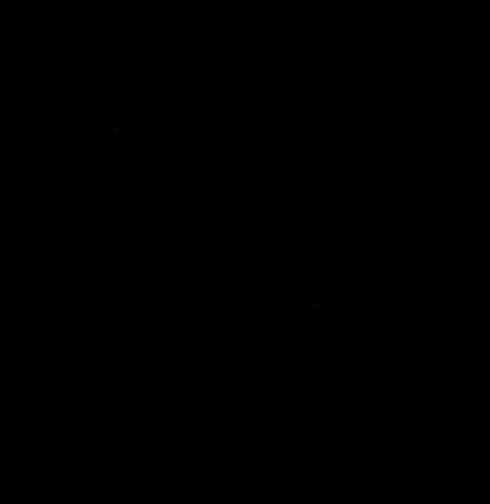

Supplement: S2 File — (ZIP) [file pone.0228610.s002.zip › 5_144/Br-16_IR_rec0691.jpg]

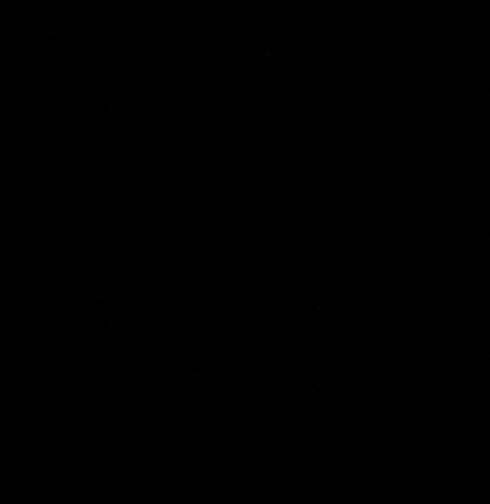

Supplement: S2 File — (ZIP) [file pone.0228610.s002.zip › 5_144/Br-16_IR_rec0695.jpg]

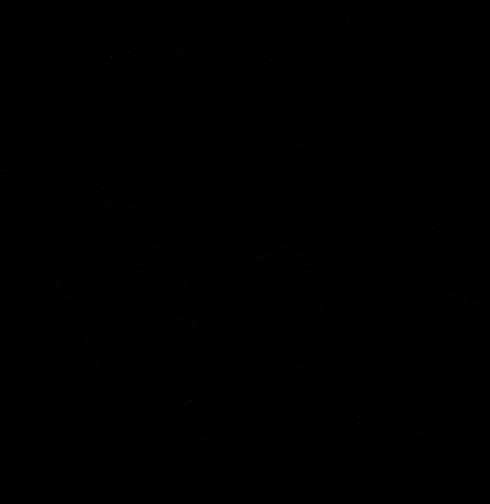

Supplement: S2 File — (ZIP) [file pone.0228610.s002.zip › 5_144/Br-16_IR_rec0699.jpg]

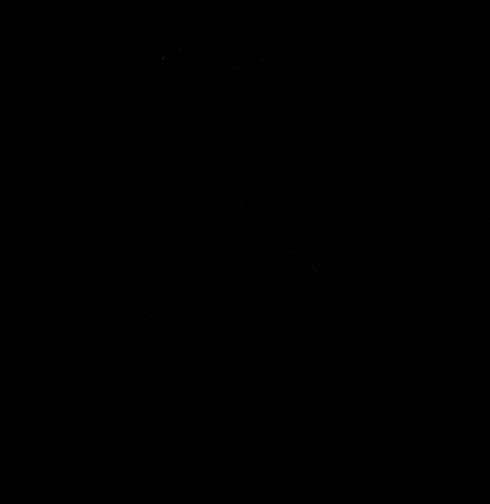

Supplement: S2 File — (ZIP) [file pone.0228610.s002.zip › 5_144/Br-16_IR_rec0703.jpg]

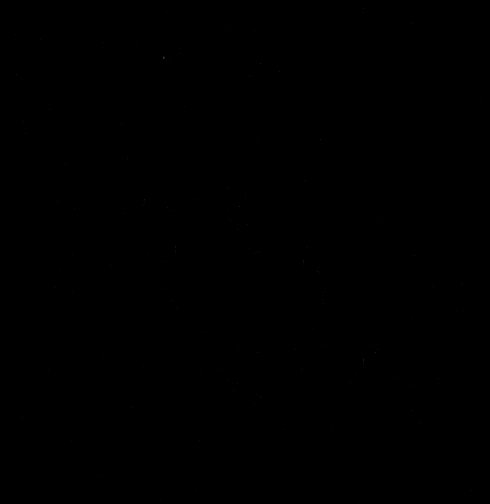

Supplement: S2 File — (ZIP) [file pone.0228610.s002.zip › 5_144/Br-16_IR_rec0707.jpg]

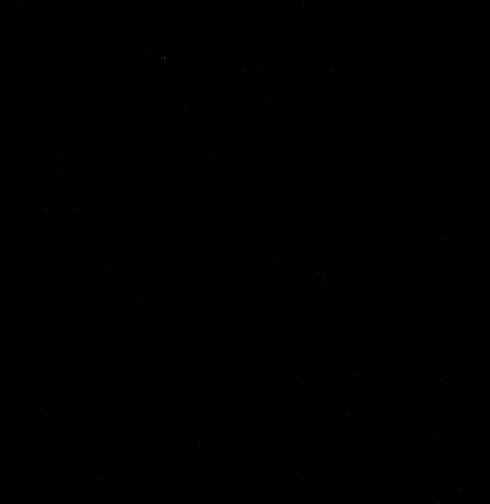

Supplement: S2 File — (ZIP) [file pone.0228610.s002.zip › 5_144/Br-16_IR_rec0711.jpg]

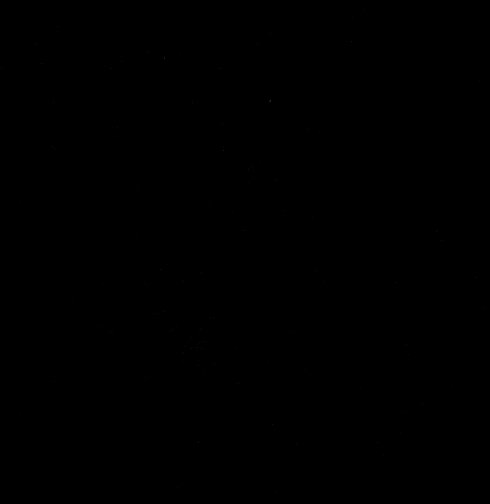

Supplement: S2 File — (ZIP) [file pone.0228610.s002.zip › 5_144/Br-16_IR_rec0715.jpg]

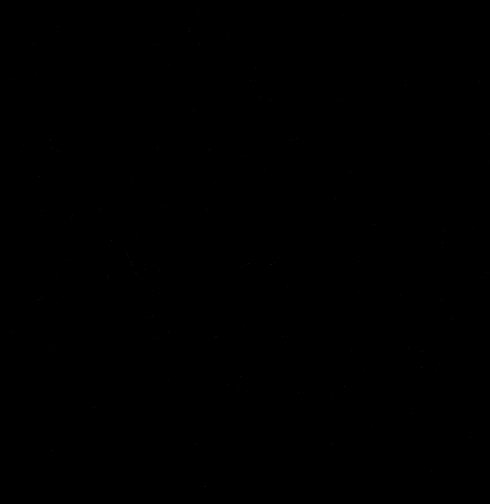

Supplement: S2 File — (ZIP) [file pone.0228610.s002.zip › 5_144/Br-16_IR_rec0719.jpg]

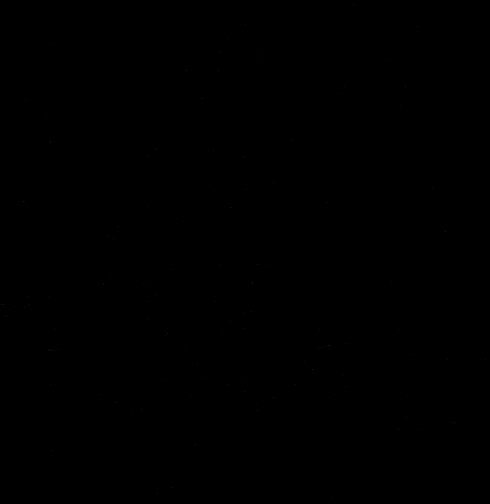

Supplement: S2 File — (ZIP) [file pone.0228610.s002.zip › 5_144/Br-16_IR_rec0723.jpg]

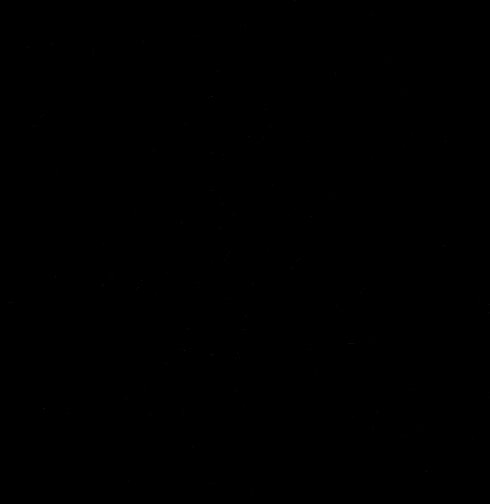

Supplement: S2 File — (ZIP) [file pone.0228610.s002.zip › 5_144/Br-16_IR_rec0727.jpg]

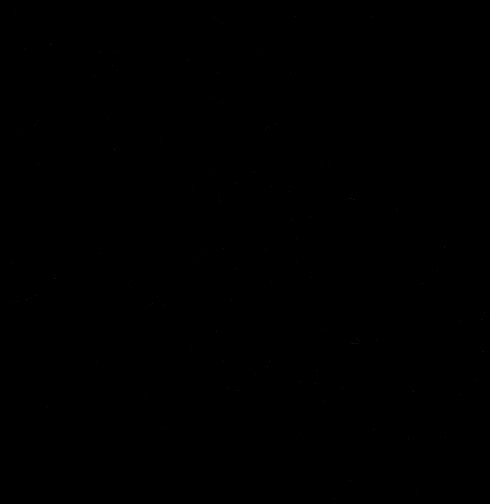

Supplement: S2 File — (ZIP) [file pone.0228610.s002.zip › 5_144/Br-16_IR_rec0731.jpg]

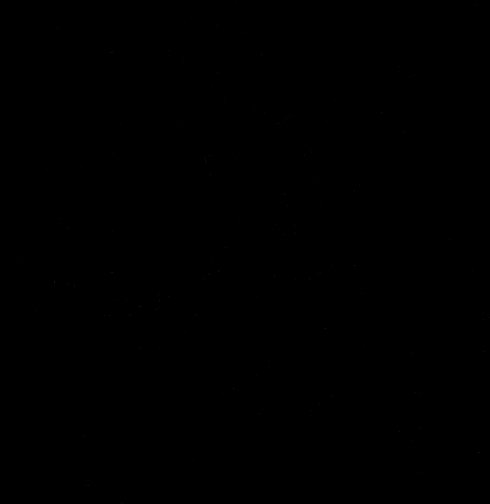

Supplement: S2 File — (ZIP) [file pone.0228610.s002.zip › 5_144/Br-16_IR_rec0735.jpg]

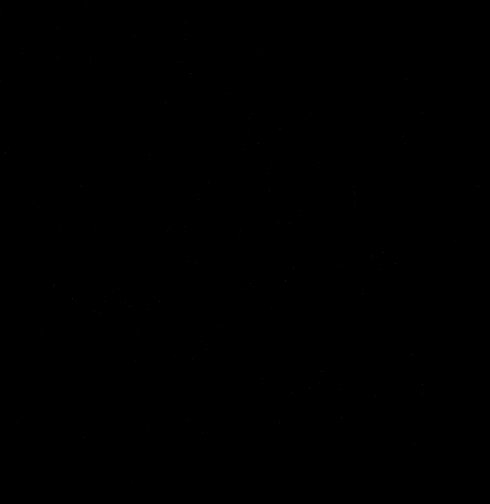

Supplement: S2 File — (ZIP) [file pone.0228610.s002.zip › 5_144/Br-16_IR_rec0739.jpg]

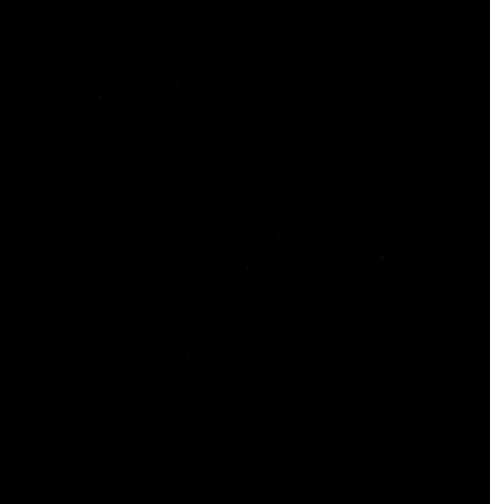

Supplement: S2 File — (ZIP) [file pone.0228610.s002.zip › 5_144/Br-16_IR_rec0743.jpg]

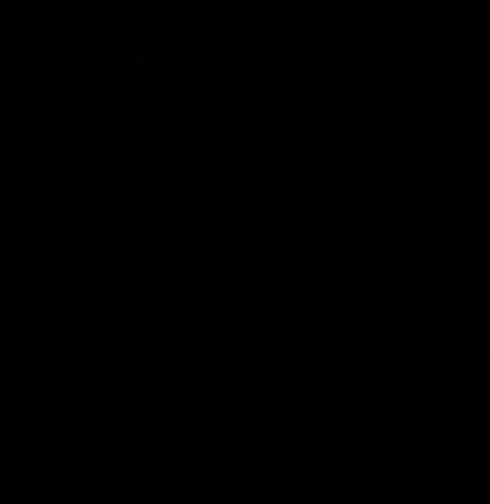

Supplement: S2 File — (ZIP) [file pone.0228610.s002.zip › 5_144/Br-16_IR_rec0747.jpg]

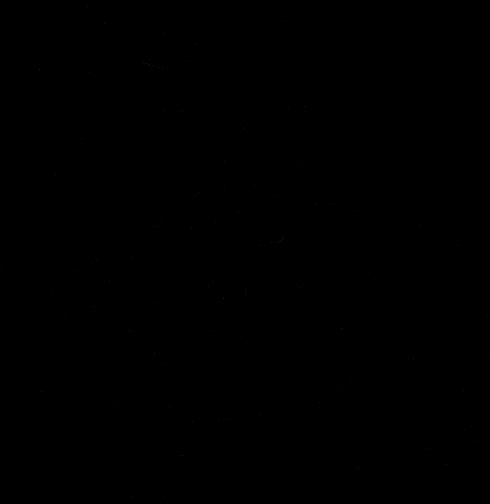

Supplement: S2 File — (ZIP) [file pone.0228610.s002.zip › 5_144/Br-16_IR_rec0751.jpg]

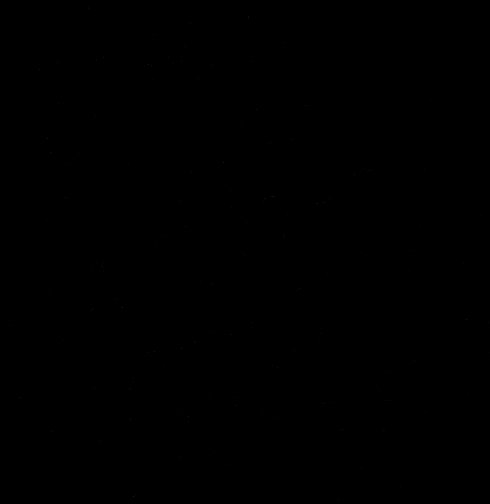

Supplement: S2 File — (ZIP) [file pone.0228610.s002.zip › 5_144/Br-16_IR_rec0755.jpg]

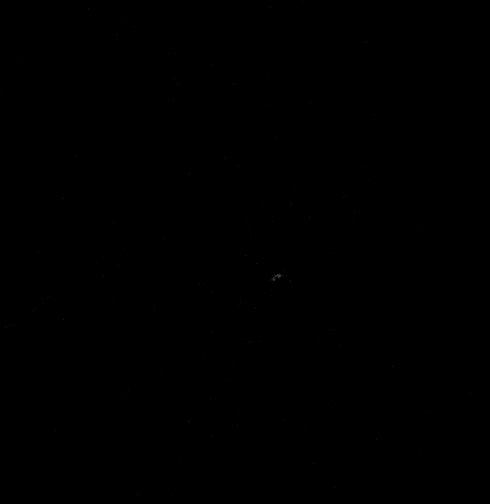

Supplement: S2 File — (ZIP) [file pone.0228610.s002.zip › 5_144/Br-16_IR_rec0759.jpg]

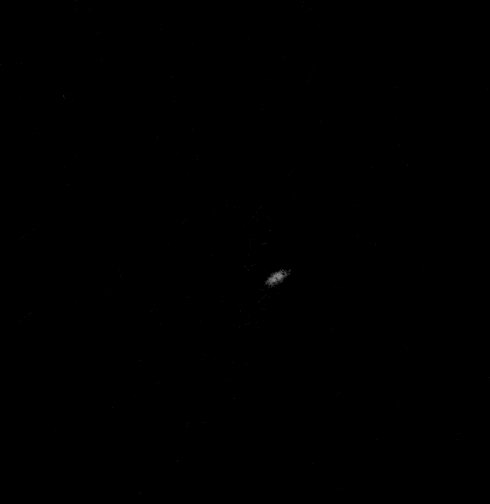

Supplement: S2 File — (ZIP) [file pone.0228610.s002.zip › 5_144/Br-16_IR_rec0763.jpg]

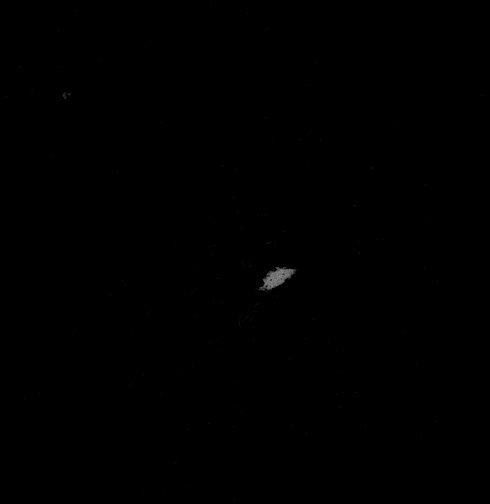

Supplement: S2 File — (ZIP) [file pone.0228610.s002.zip › 5_144/Br-16_IR_rec0767.jpg]

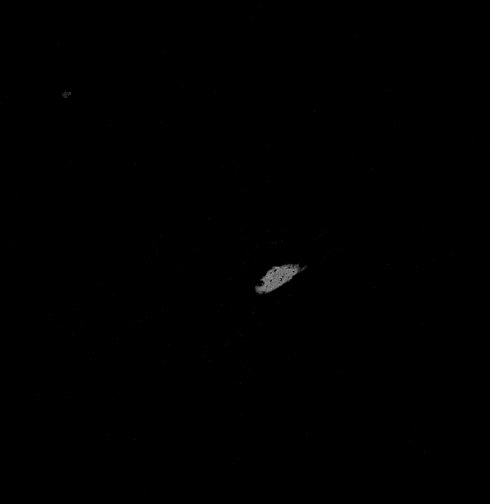

Supplement: S2 File — (ZIP) [file pone.0228610.s002.zip › 5_144/Br-16_IR_rec0771.jpg]

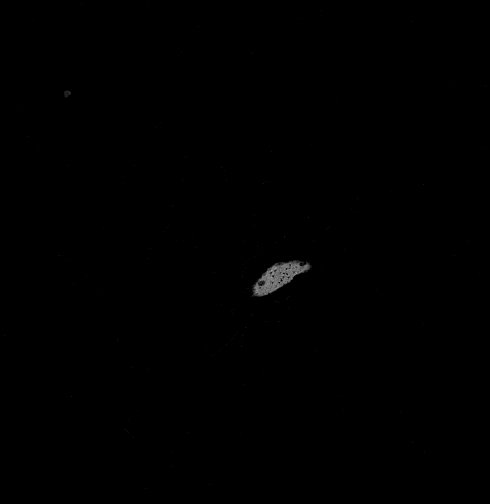

Supplement: S2 File — (ZIP) [file pone.0228610.s002.zip › 5_144/Br-16_IR_rec0775.jpg]

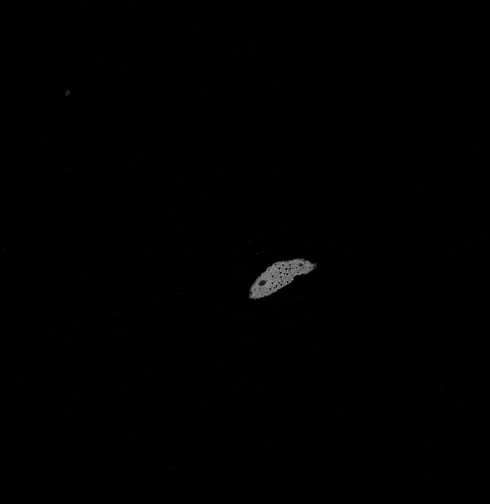

Supplement: S2 File — (ZIP) [file pone.0228610.s002.zip › 5_144/Br-16_IR_rec0779.jpg]

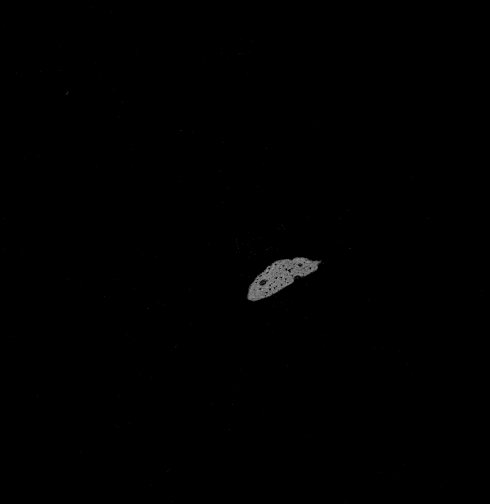

Supplement: S2 File — (ZIP) [file pone.0228610.s002.zip › 5_144/Br-16_IR_rec0783.jpg]

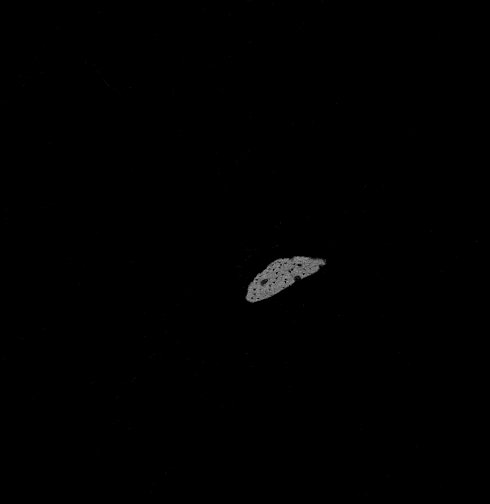

Supplement: S2 File — (ZIP) [file pone.0228610.s002.zip › 5_144/Br-16_IR_rec0787.jpg]

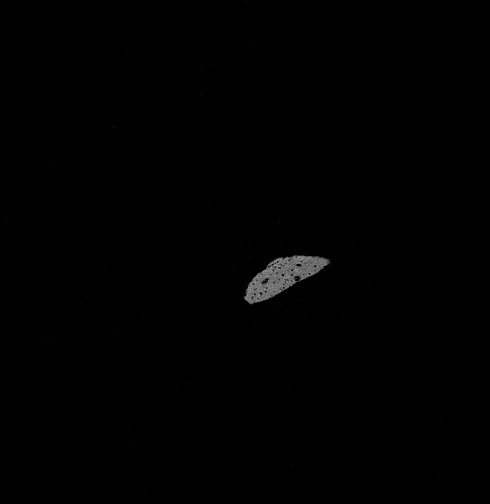

Supplement: S2 File — (ZIP) [file pone.0228610.s002.zip › 5_144/Br-16_IR_rec0791.jpg]

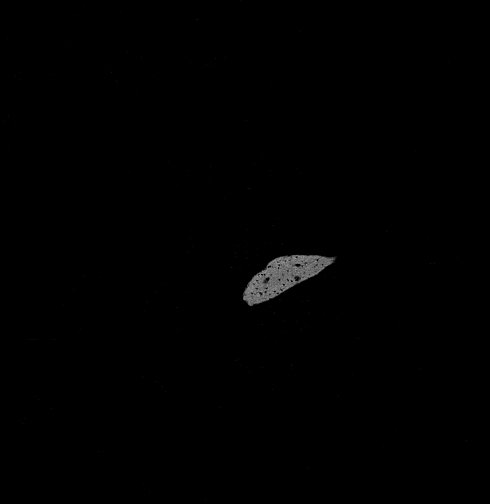

Supplement: S2 File — (ZIP) [file pone.0228610.s002.zip › 5_144/Br-16_IR_rec0795.jpg]

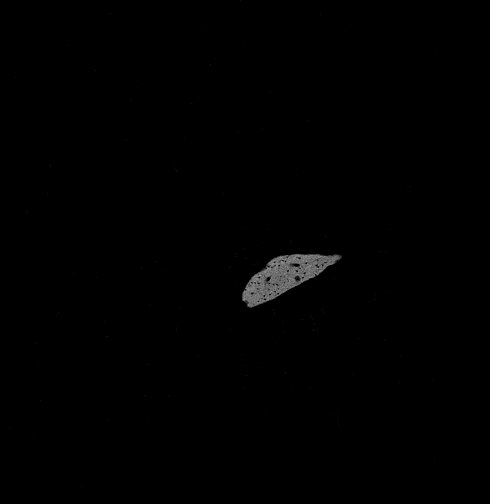

Supplement: S2 File — (ZIP) [file pone.0228610.s002.zip › 5_144/Br-16_IR_rec0799.jpg]

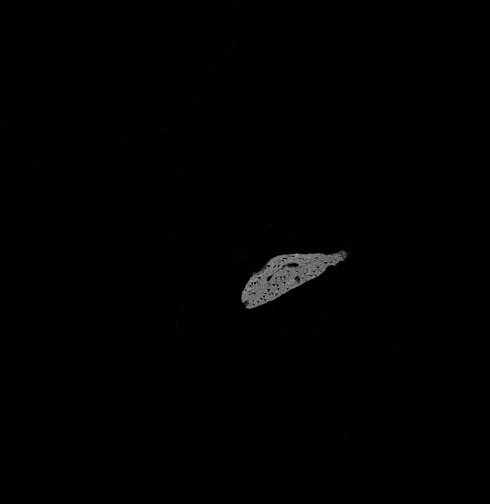

Supplement: S2 File — (ZIP) [file pone.0228610.s002.zip › 5_144/Br-16_IR_rec0803.jpg]

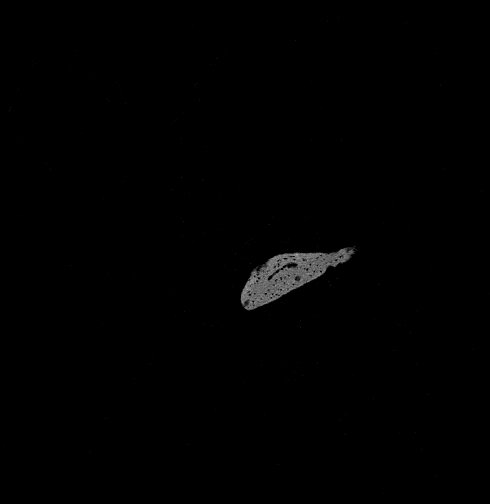

Supplement: S2 File — (ZIP) [file pone.0228610.s002.zip › 5_144/Br-16_IR_rec0807.jpg]

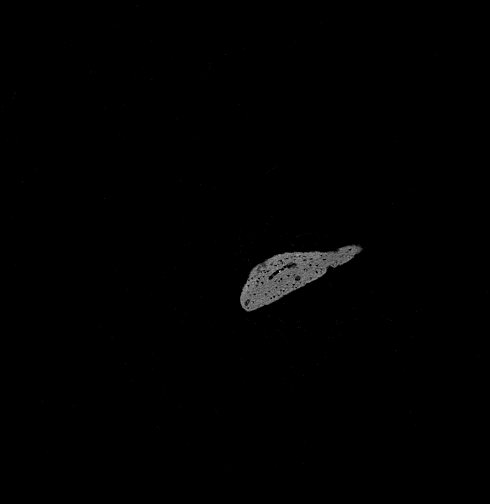

Supplement: S2 File — (ZIP) [file pone.0228610.s002.zip › 5_144/Br-16_IR_rec0811.jpg]

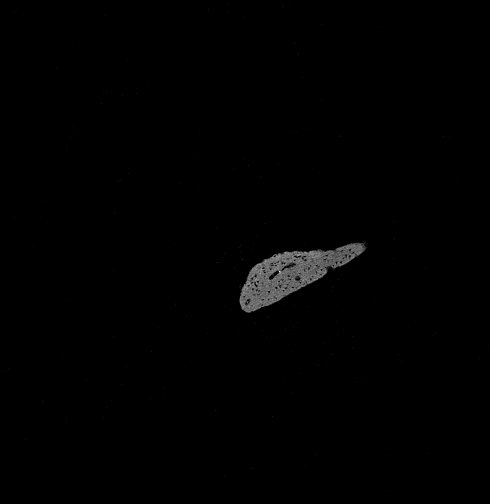

Supplement: S2 File — (ZIP) [file pone.0228610.s002.zip › 5_144/Br-16_IR_rec0815.jpg]

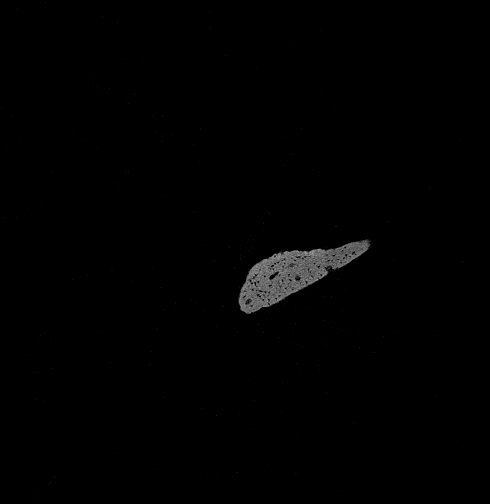

Supplement: S2 File — (ZIP) [file pone.0228610.s002.zip › 5_144/Br-16_IR_rec0819.jpg]

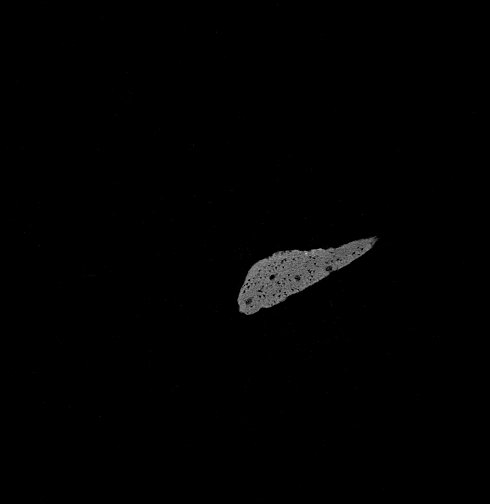

Supplement: S2 File — (ZIP) [file pone.0228610.s002.zip › 5_144/Br-16_IR_rec0823.jpg]

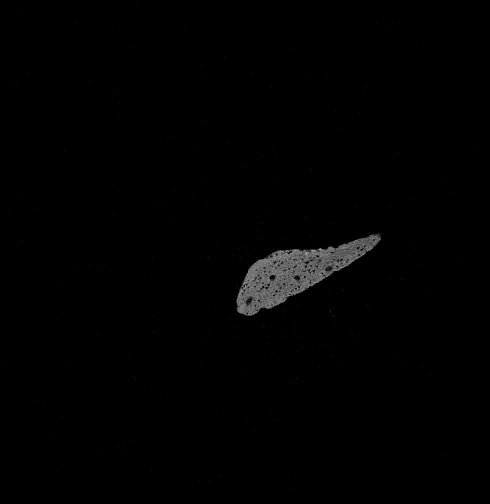

Supplement: S2 File — (ZIP) [file pone.0228610.s002.zip › 5_144/Br-16_IR_rec0827.jpg]

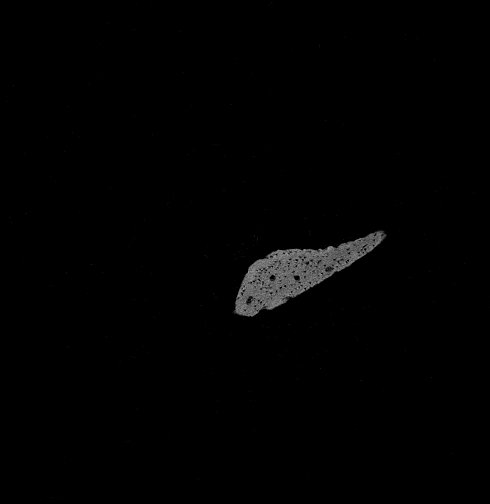

Supplement: S2 File — (ZIP) [file pone.0228610.s002.zip › 5_144/Br-16_IR_rec0831.jpg]

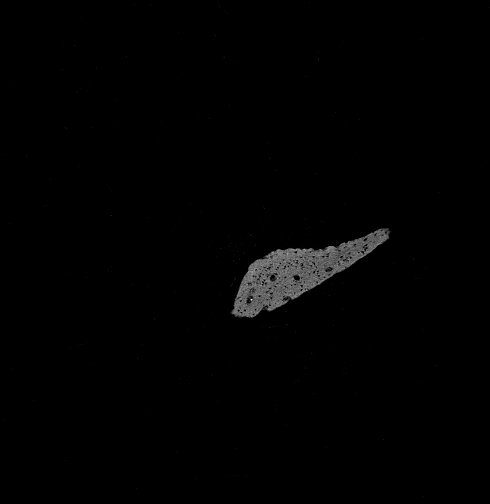

Supplement: S2 File — (ZIP) [file pone.0228610.s002.zip › 5_144/Br-16_IR_rec0835.jpg]

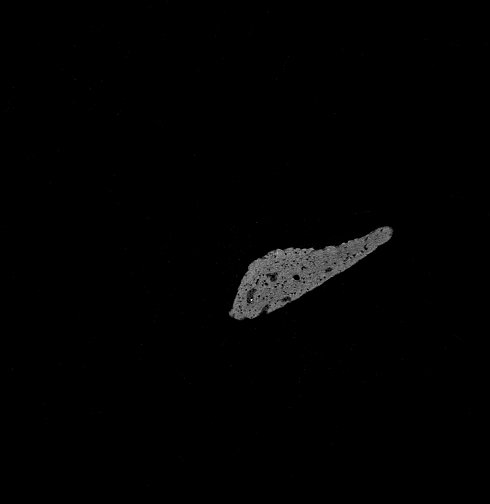

Supplement: S2 File — (ZIP) [file pone.0228610.s002.zip › 5_144/Br-16_IR_rec0839.jpg]

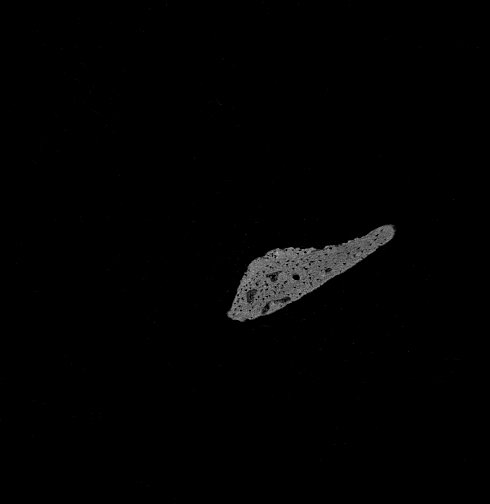

Supplement: S2 File — (ZIP) [file pone.0228610.s002.zip › 5_144/Br-16_IR_rec0843.jpg]

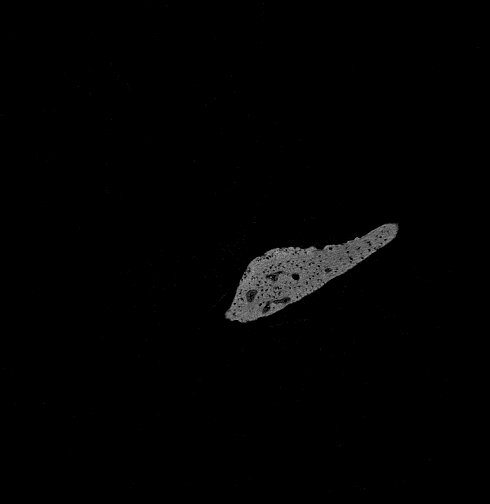

Supplement: S2 File — (ZIP) [file pone.0228610.s002.zip › 5_144/Br-16_IR_rec0847.jpg]

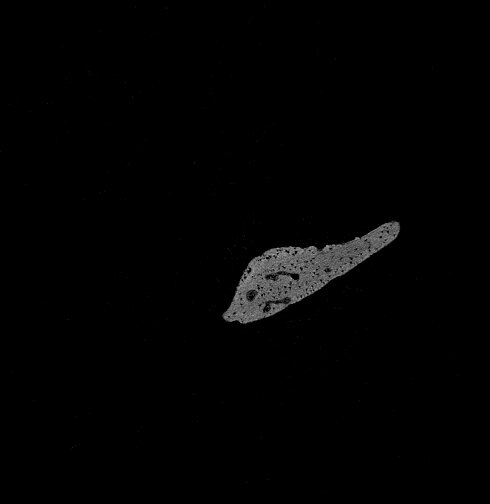

Supplement: S2 File — (ZIP) [file pone.0228610.s002.zip › 5_144/Br-16_IR_rec0851.jpg]

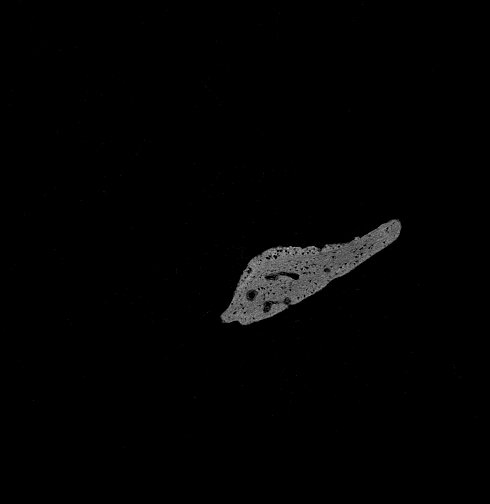

Supplement: S2 File — (ZIP) [file pone.0228610.s002.zip › 5_144/Br-16_IR_rec0855.jpg]

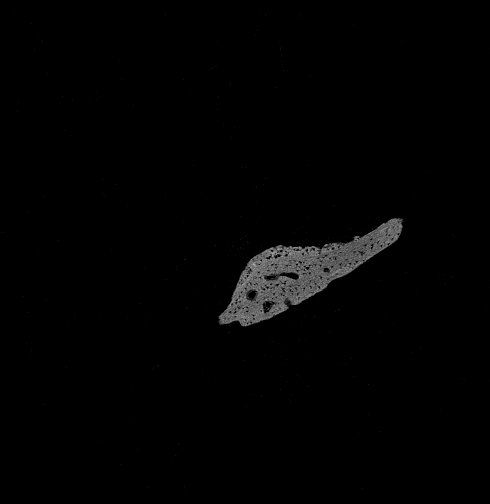

Supplement: S2 File — (ZIP) [file pone.0228610.s002.zip › 5_144/Br-16_IR_rec0859.jpg]

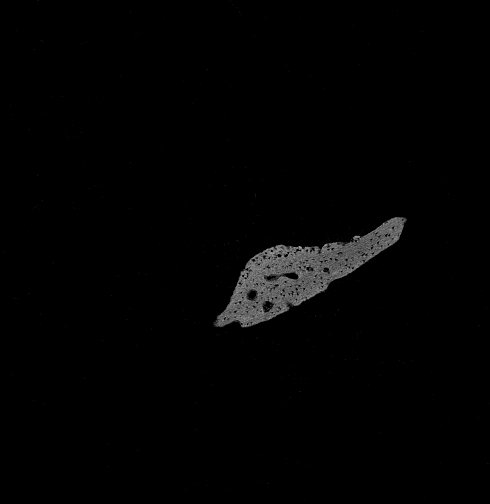

Supplement: S2 File — (ZIP) [file pone.0228610.s002.zip › 5_144/Br-16_IR_rec0863.jpg]

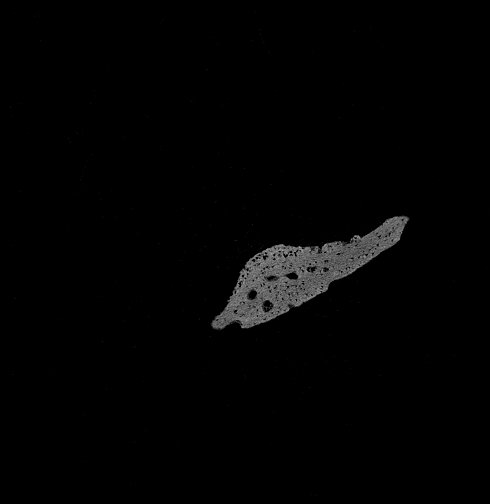

Supplement: S2 File — (ZIP) [file pone.0228610.s002.zip › 5_144/Br-16_IR_rec0867.jpg]

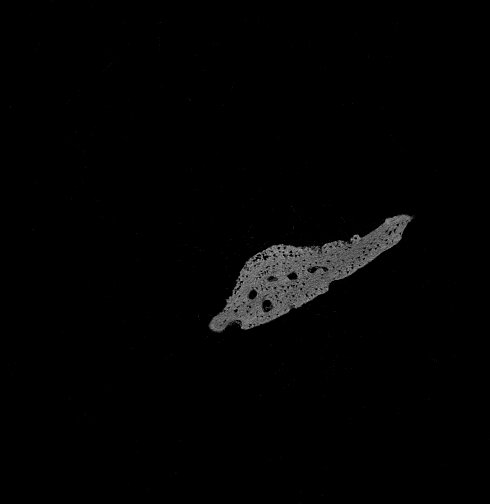

Supplement: S2 File — (ZIP) [file pone.0228610.s002.zip › 5_144/Br-16_IR_rec0871.jpg]

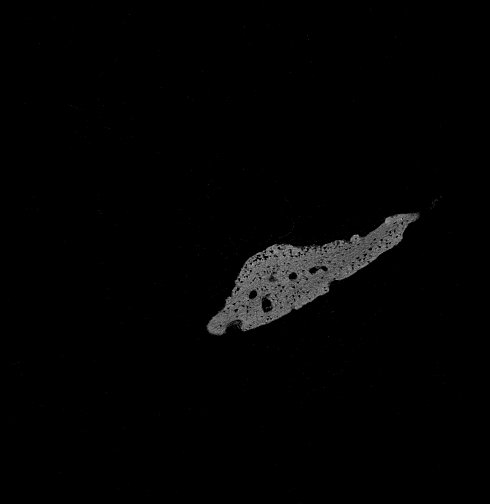

Supplement: S2 File — (ZIP) [file pone.0228610.s002.zip › 5_144/Br-16_IR_rec0875.jpg]

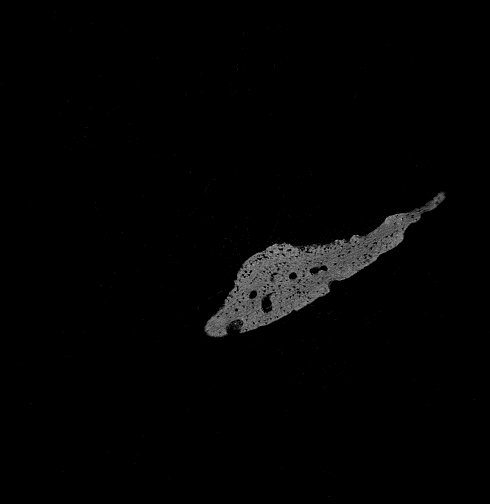

Supplement: S2 File — (ZIP) [file pone.0228610.s002.zip › 5_144/Br-16_IR_rec0879.jpg]

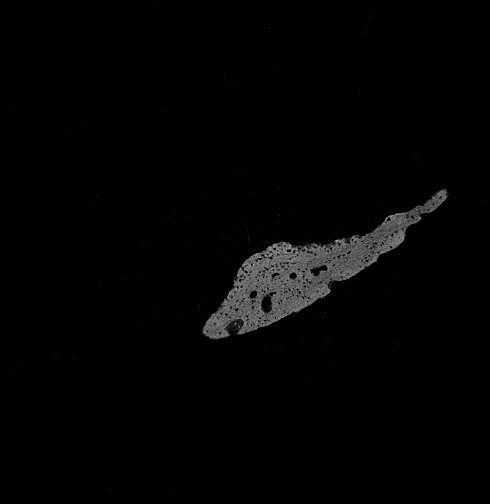

Supplement: S2 File — (ZIP) [file pone.0228610.s002.zip › 5_144/Br-16_IR_rec0883.jpg]

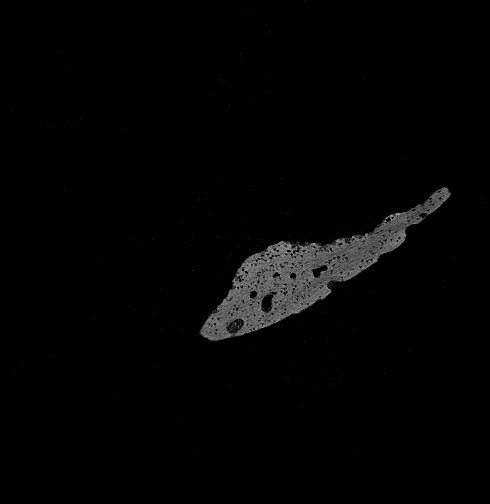

Supplement: S2 File — (ZIP) [file pone.0228610.s002.zip › 5_144/Br-16_IR_rec0887.jpg]

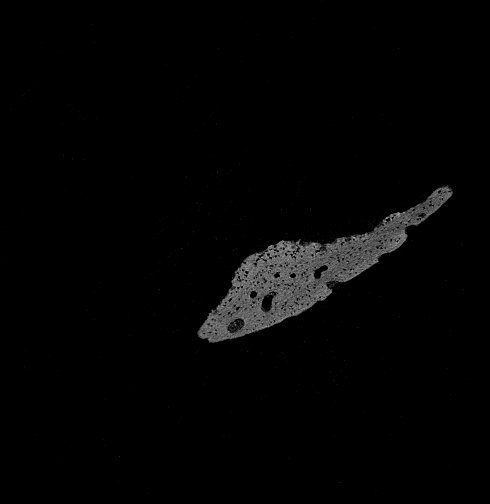

Supplement: S2 File — (ZIP) [file pone.0228610.s002.zip › 5_144/Br-16_IR_rec0891.jpg]

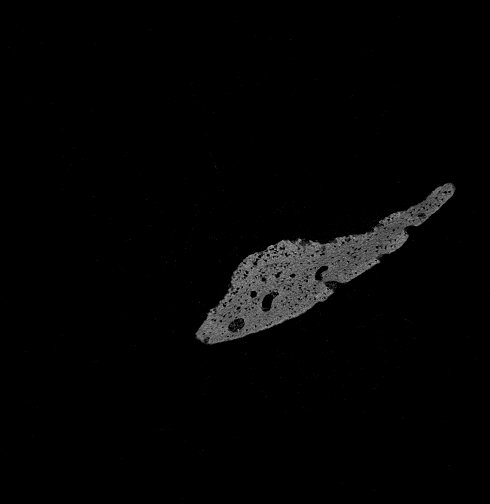

Supplement: S2 File — (ZIP) [file pone.0228610.s002.zip › 5_144/Br-16_IR_rec0895.jpg]

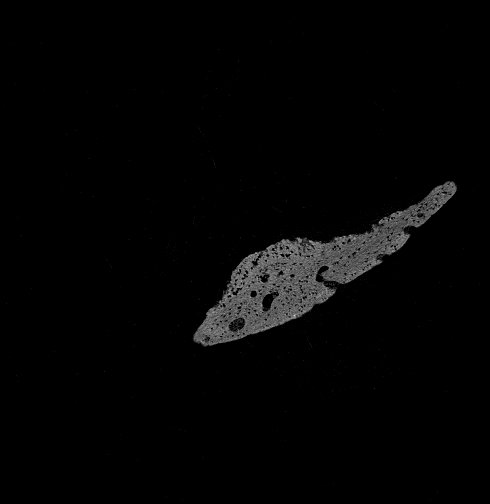

Supplement: S2 File — (ZIP) [file pone.0228610.s002.zip › 5_144/Br-16_IR_rec0899.jpg]

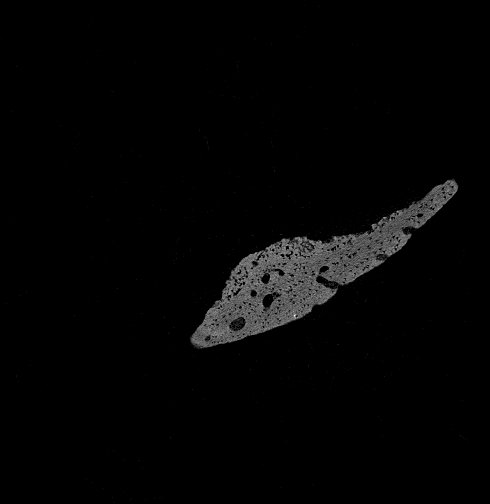

Supplement: S2 File — (ZIP) [file pone.0228610.s002.zip › 5_144/Br-16_IR_rec0903.jpg]

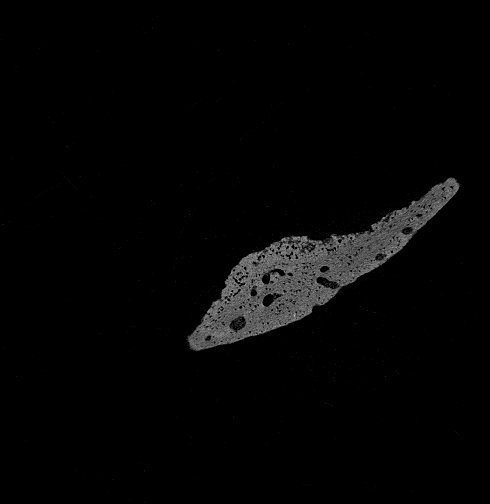

Supplement: S2 File — (ZIP) [file pone.0228610.s002.zip › 5_144/Br-16_IR_rec0907.jpg]

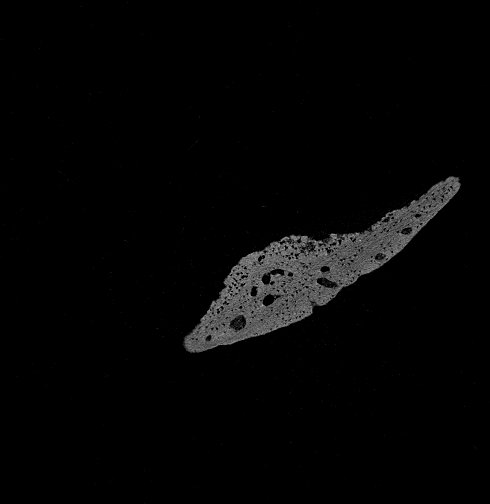

Supplement: S2 File — (ZIP) [file pone.0228610.s002.zip › 5_144/Br-16_IR_rec0911.jpg]

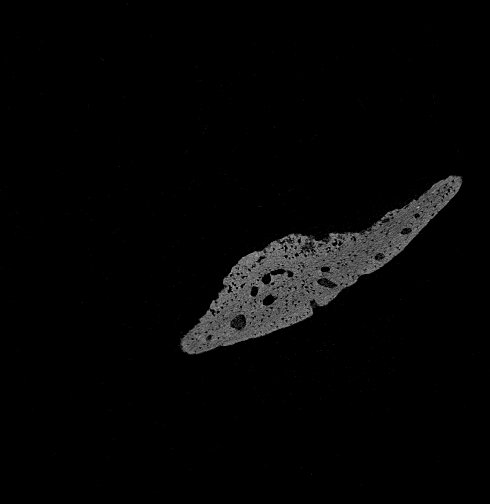

Supplement: S2 File — (ZIP) [file pone.0228610.s002.zip › 5_144/Br-16_IR_rec0915.jpg]

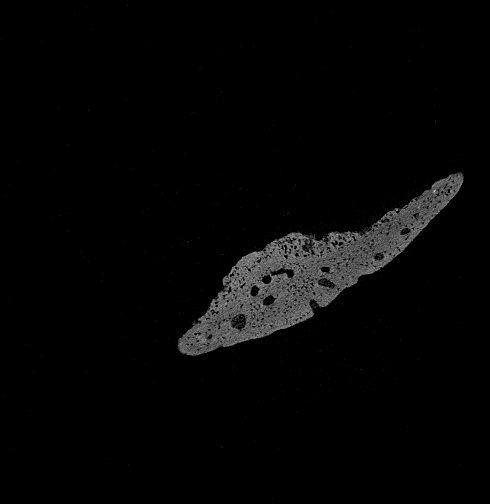

Supplement: S2 File — (ZIP) [file pone.0228610.s002.zip › 5_144/Br-16_IR_rec0919.jpg]

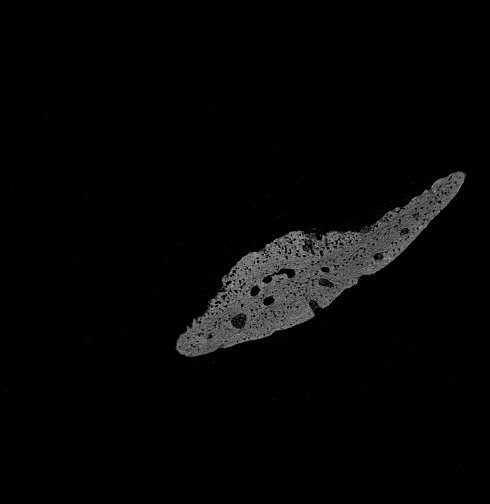

Supplement: S2 File — (ZIP) [file pone.0228610.s002.zip › 5_144/Br-16_IR_rec0923.jpg]

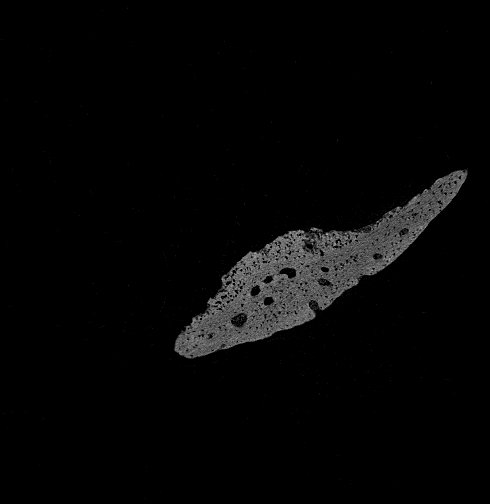

Supplement: S2 File — (ZIP) [file pone.0228610.s002.zip › 5_144/Br-16_IR_rec0927.jpg]

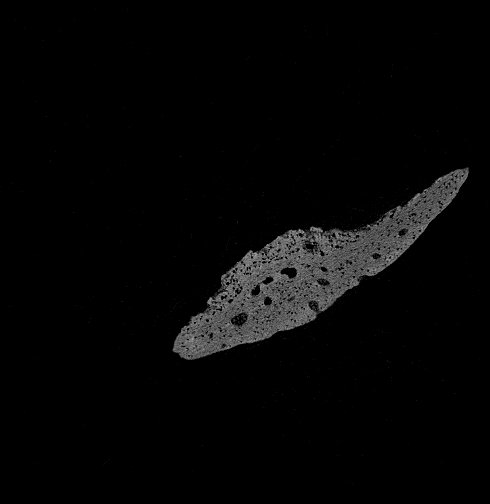

Supplement: S2 File — (ZIP) [file pone.0228610.s002.zip › 5_144/Br-16_IR_rec0931.jpg]

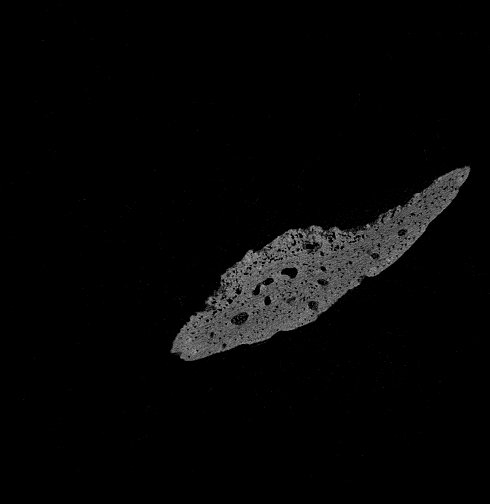

Supplement: S2 File — (ZIP) [file pone.0228610.s002.zip › 5_144/Br-16_IR_rec0935.jpg]

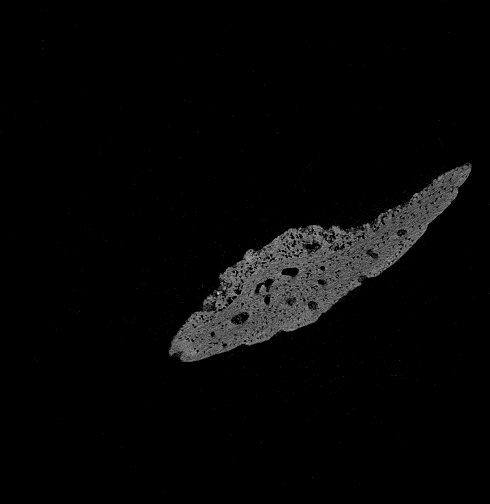

Supplement: S2 File — (ZIP) [file pone.0228610.s002.zip › 5_144/Br-16_IR_rec0939.jpg]

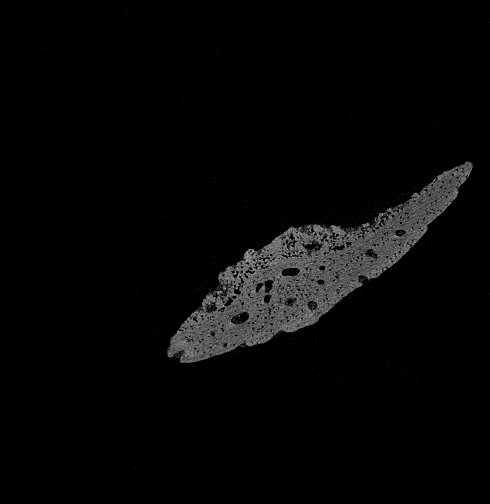

Supplement: S2 File — (ZIP) [file pone.0228610.s002.zip › 5_144/Br-16_IR_rec0943.jpg]

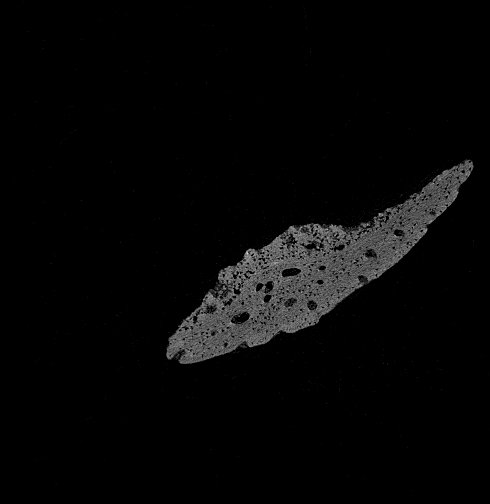

Supplement: S2 File — (ZIP) [file pone.0228610.s002.zip › 5_144/Br-16_IR_rec0947.jpg]

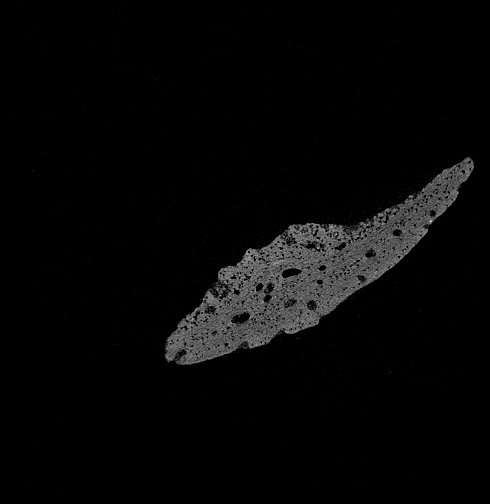

Supplement: S2 File — (ZIP) [file pone.0228610.s002.zip › 5_144/Br-16_IR_rec0951.jpg]

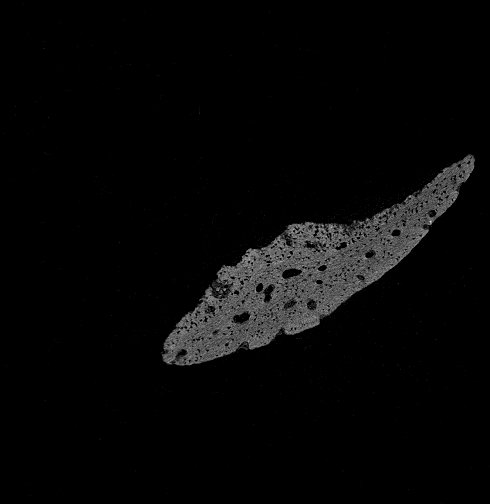

Supplement: S2 File — (ZIP) [file pone.0228610.s002.zip › 5_144/Br-16_IR_rec0955.jpg]

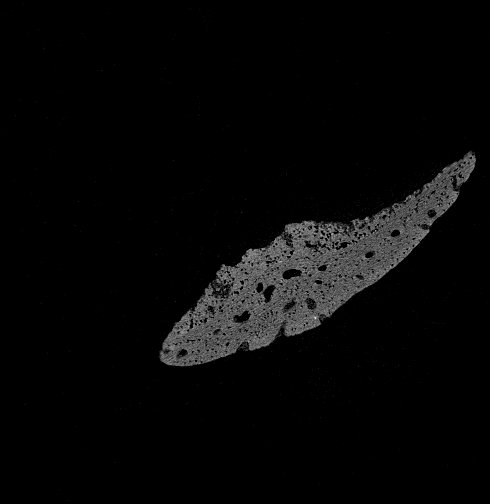

Supplement: S2 File — (ZIP) [file pone.0228610.s002.zip › 5_144/Br-16_IR_rec0959.jpg]

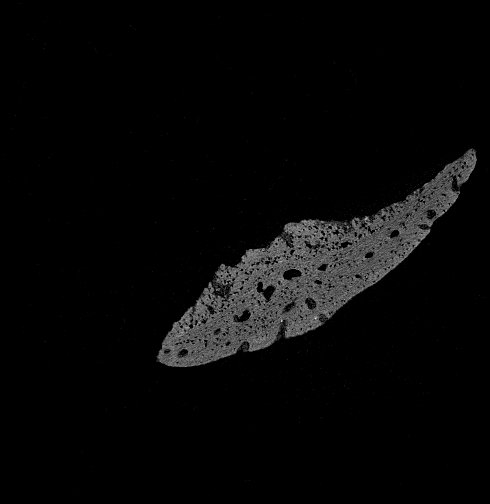

Supplement: S2 File — (ZIP) [file pone.0228610.s002.zip › 5_144/Br-16_IR_rec0963.jpg]

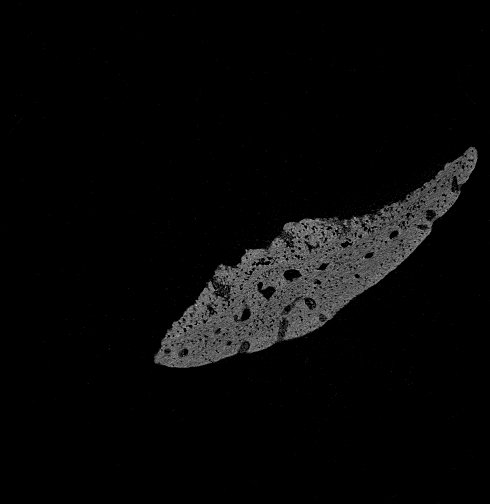

Supplement: S2 File — (ZIP) [file pone.0228610.s002.zip › 5_144/Br-16_IR_rec0967.jpg]

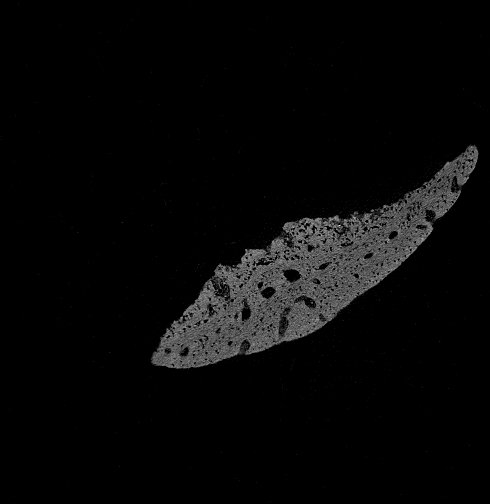

Supplement: S2 File — (ZIP) [file pone.0228610.s002.zip › 5_144/Br-16_IR_rec0971.jpg]

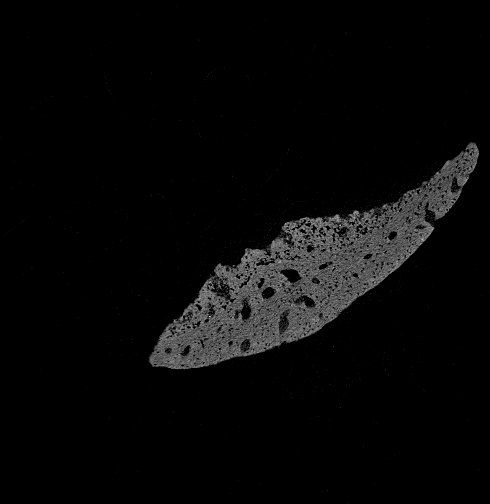

Supplement: S2 File — (ZIP) [file pone.0228610.s002.zip › 5_144/Br-16_IR_rec0975.jpg]

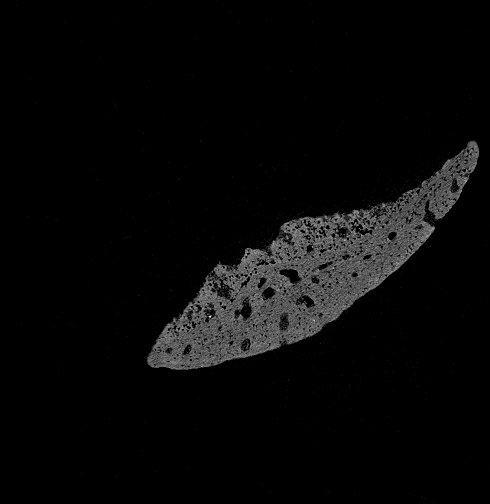

Supplement: S2 File — (ZIP) [file pone.0228610.s002.zip › 5_144/Br-16_IR_rec0979.jpg]

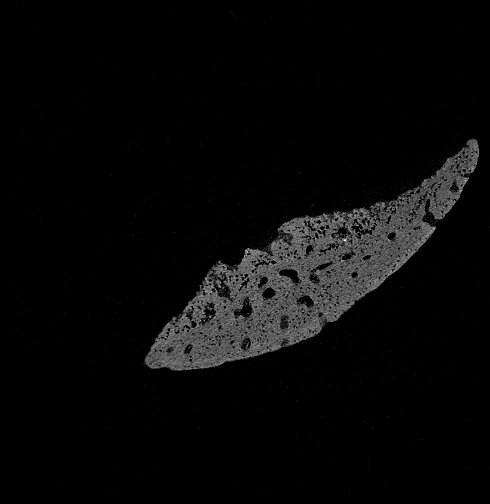

Supplement: S2 File — (ZIP) [file pone.0228610.s002.zip › 5_144/Br-16_IR_rec0983.jpg]

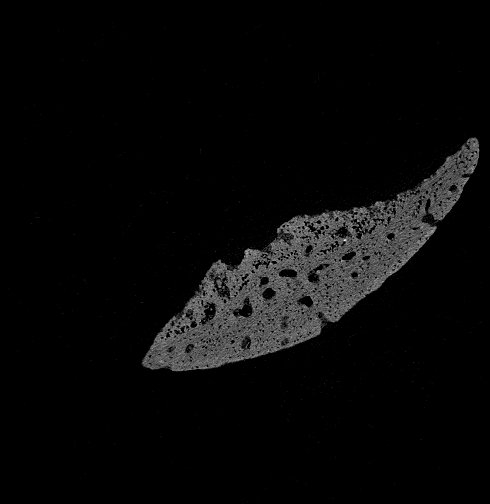

Supplement: S2 File — (ZIP) [file pone.0228610.s002.zip › 5_144/Br-16_IR_rec0987.jpg]

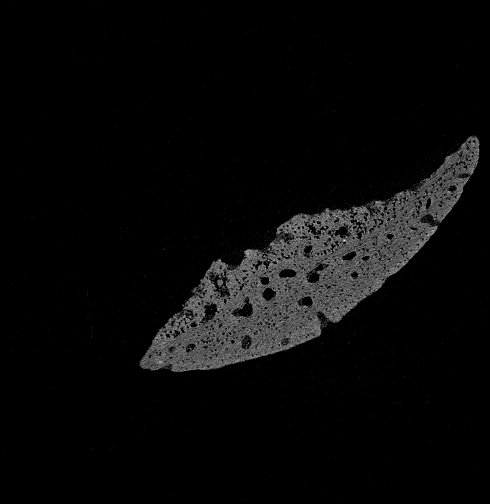

Supplement: S2 File — (ZIP) [file pone.0228610.s002.zip › 5_144/Br-16_IR_rec0991.jpg]

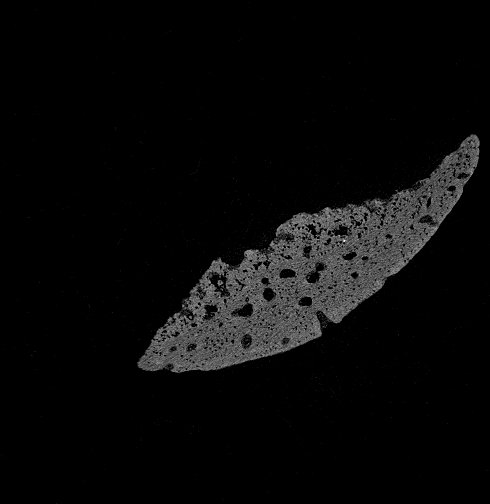

Supplement: S2 File — (ZIP) [file pone.0228610.s002.zip › 5_144/Br-16_IR_rec0995.jpg]

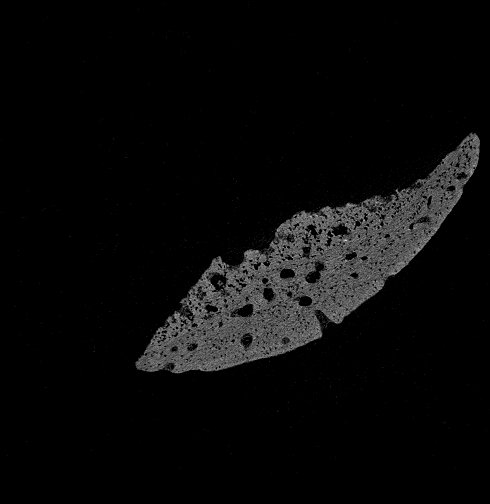

Supplement: S2 File — (ZIP) [file pone.0228610.s002.zip › 5_144/Br-16_IR_rec0999.jpg]

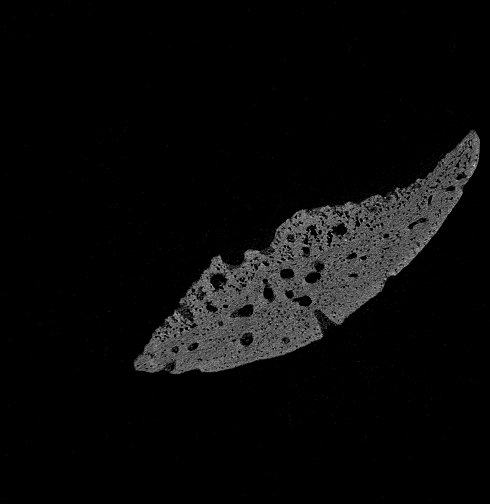

Supplement: S2 File — (ZIP) [file pone.0228610.s002.zip › 5_144/Br-16_IR_rec1003.jpg]

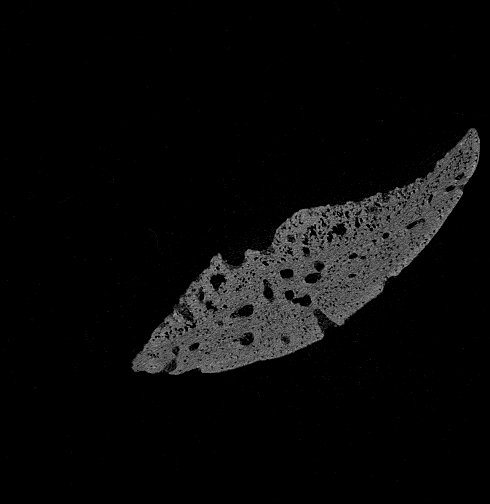

Supplement: S2 File — (ZIP) [file pone.0228610.s002.zip › 5_144/Br-16_IR_rec1007.jpg]

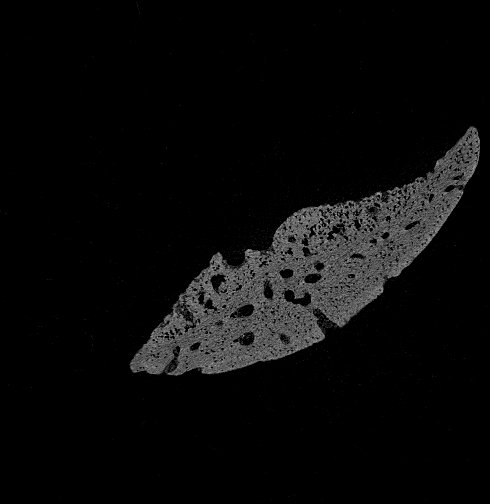

Supplement: S2 File — (ZIP) [file pone.0228610.s002.zip › 5_144/Br-16_IR_rec1011.jpg]

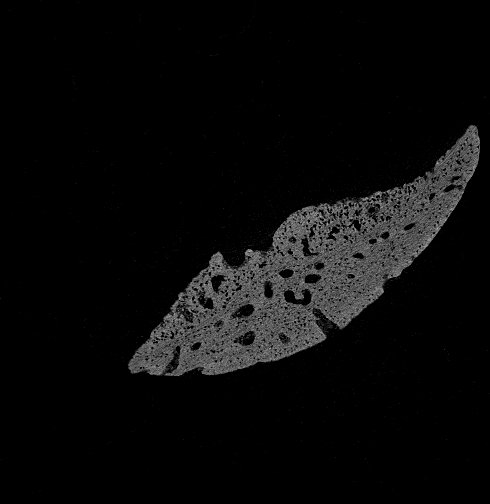

Supplement: S2 File — (ZIP) [file pone.0228610.s002.zip › 5_144/Br-16_IR_rec1015.jpg]

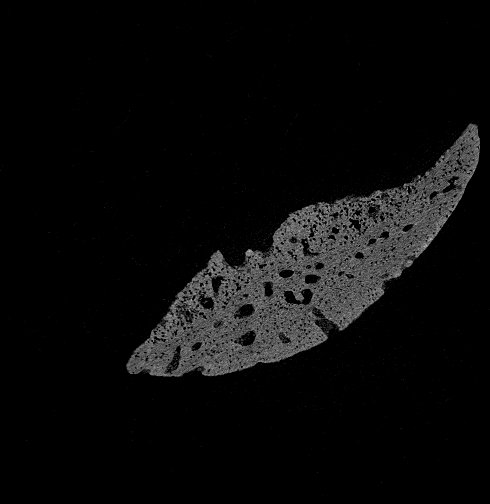

Supplement: S2 File — (ZIP) [file pone.0228610.s002.zip › 5_144/Br-16_IR_rec1019.jpg]

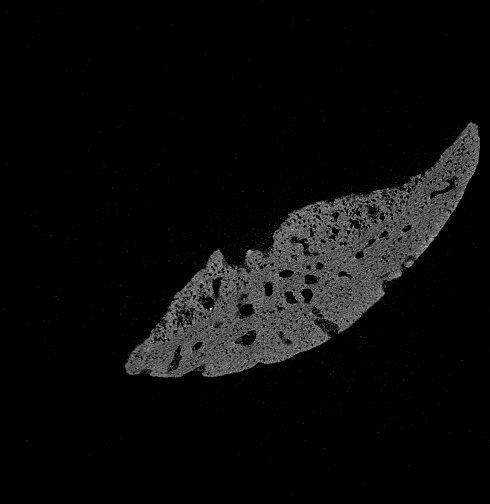

Supplement: S2 File — (ZIP) [file pone.0228610.s002.zip › 5_144/Br-16_IR_rec1023.jpg]

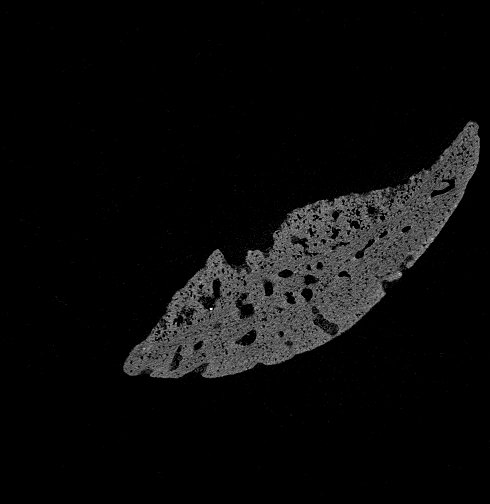

Supplement: S2 File — (ZIP) [file pone.0228610.s002.zip › 5_144/Br-16_IR_rec1027.jpg]

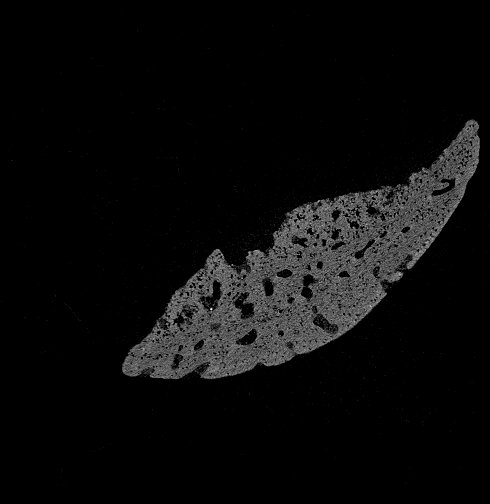

Supplement: S2 File — (ZIP) [file pone.0228610.s002.zip › 5_144/Br-16_IR_rec1031.jpg]

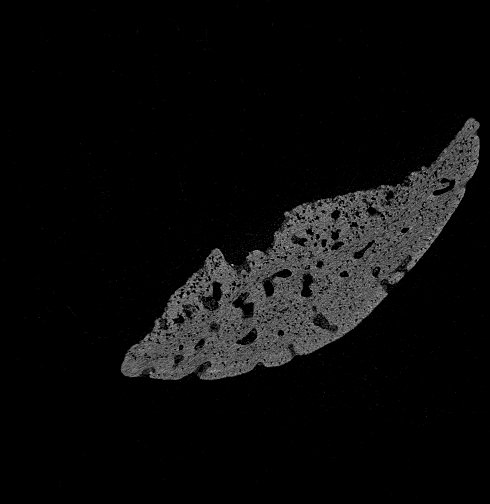

Supplement: S2 File — (ZIP) [file pone.0228610.s002.zip › 5_144/Br-16_IR_rec1035.jpg]

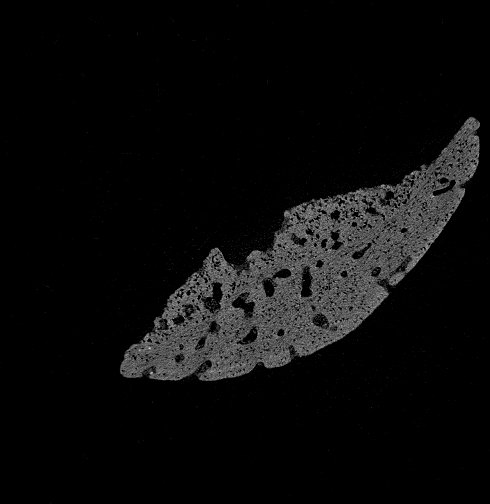

Supplement: S2 File — (ZIP) [file pone.0228610.s002.zip › 5_144/Br-16_IR_rec1039.jpg]

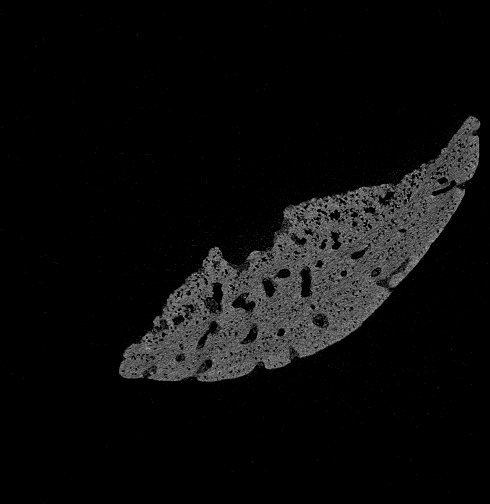

Supplement: S2 File — (ZIP) [file pone.0228610.s002.zip › 5_144/Br-16_IR_rec1043.jpg]

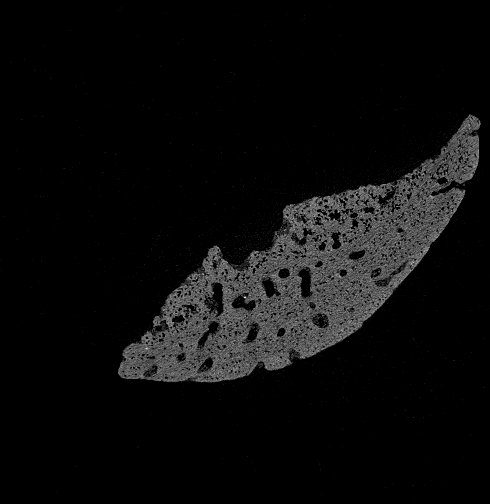

Supplement: S2 File — (ZIP) [file pone.0228610.s002.zip › 5_144/Br-16_IR_rec1047.jpg]

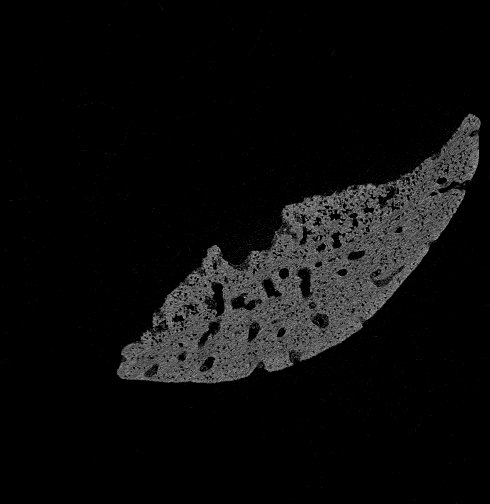

Supplement: S2 File — (ZIP) [file pone.0228610.s002.zip › 5_144/Br-16_IR_rec1051.jpg]

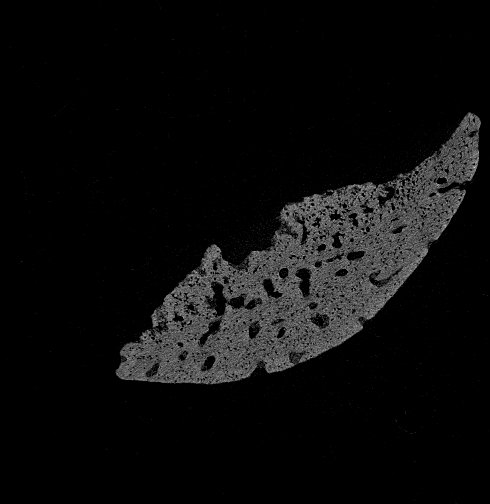

Supplement: S2 File — (ZIP) [file pone.0228610.s002.zip › 5_144/Br-16_IR_rec1055.jpg]

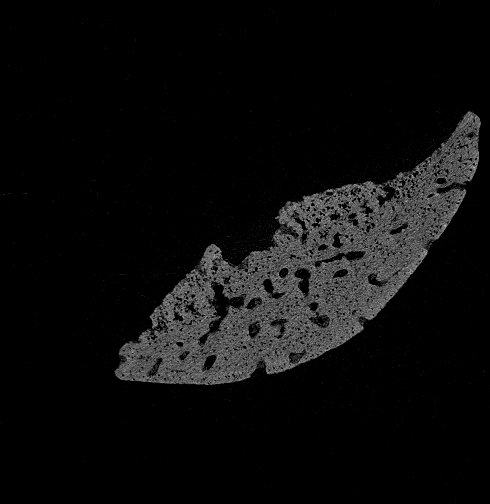

Supplement: S2 File — (ZIP) [file pone.0228610.s002.zip › 5_144/Br-16_IR_rec1059.jpg]
