# Supplementary figures and images for: A new small-sized stem salamander from the Middle Jurassic of Western Siberia, Russia (part 6 of 10)
Source: PLoS One. 2020 Feb 19;15(2):e0228610. doi: 10.1371/journal.pone.0228610 (PMC7029856; doi:10.1371/journal.pone.0228610)

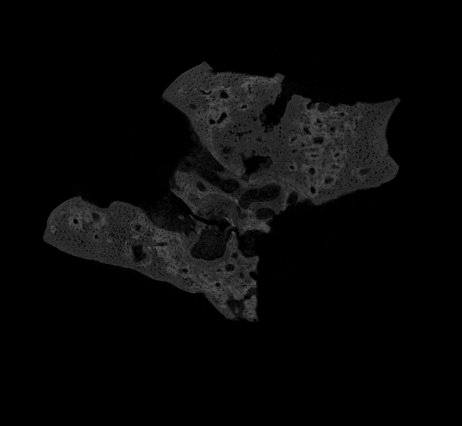

Supplement: S3 File — (ZIP) [file pone.0228610.s003.zip › 6_144/Br_II__IR_rec1054.jpg]

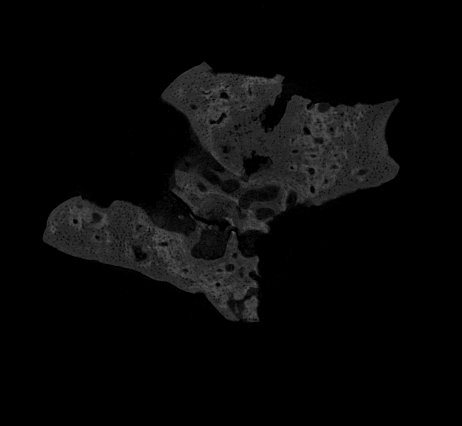

Supplement: S3 File — (ZIP) [file pone.0228610.s003.zip › 6_144/Br_II__IR_rec1058.jpg]

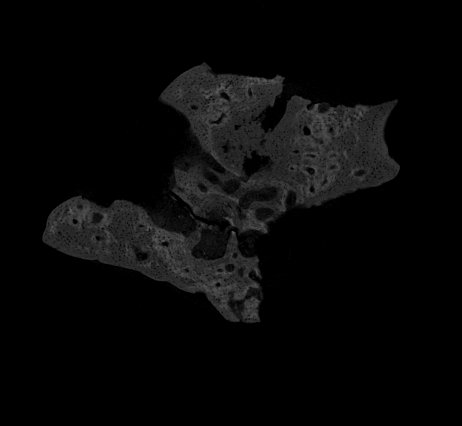

Supplement: S3 File — (ZIP) [file pone.0228610.s003.zip › 6_144/Br_II__IR_rec1062.jpg]

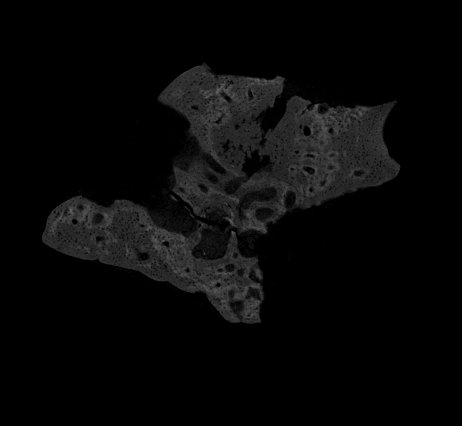

Supplement: S3 File — (ZIP) [file pone.0228610.s003.zip › 6_144/Br_II__IR_rec1066.jpg]

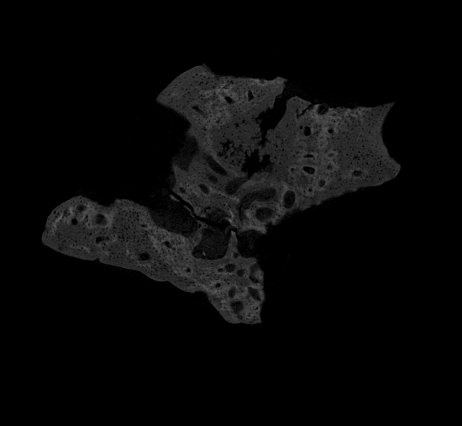

Supplement: S3 File — (ZIP) [file pone.0228610.s003.zip › 6_144/Br_II__IR_rec1070.jpg]

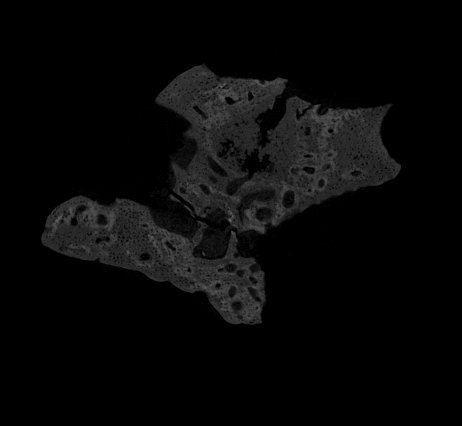

Supplement: S3 File — (ZIP) [file pone.0228610.s003.zip › 6_144/Br_II__IR_rec1074.jpg]

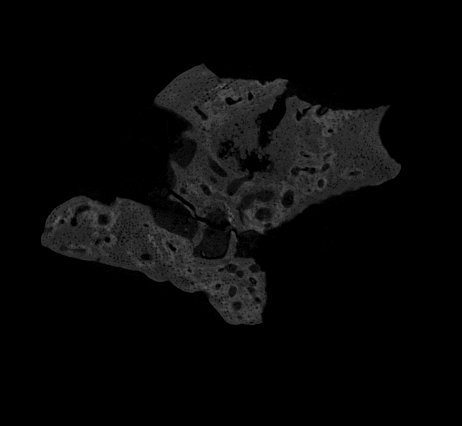

Supplement: S3 File — (ZIP) [file pone.0228610.s003.zip › 6_144/Br_II__IR_rec1078.jpg]

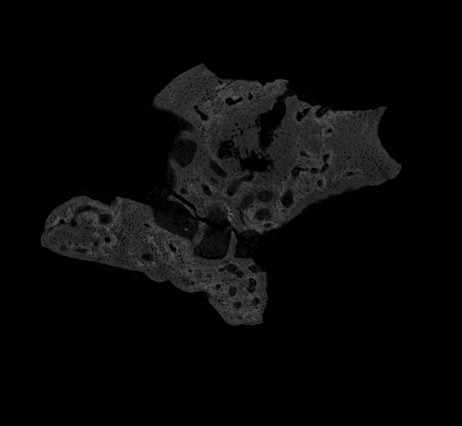

Supplement: S3 File — (ZIP) [file pone.0228610.s003.zip › 6_144/Br_II__IR_rec1082.jpg]

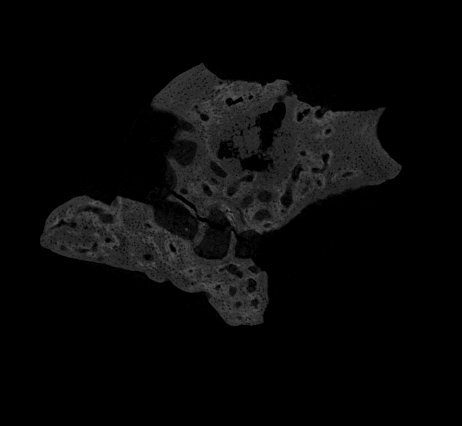

Supplement: S3 File — (ZIP) [file pone.0228610.s003.zip › 6_144/Br_II__IR_rec1086.jpg]

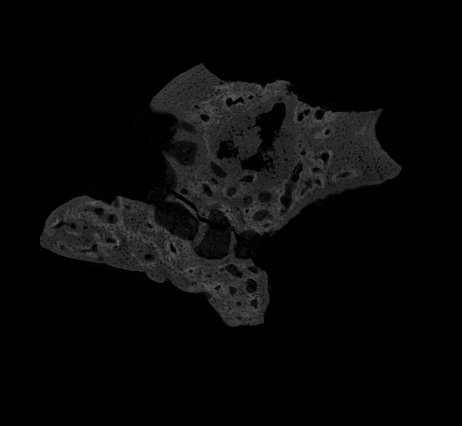

Supplement: S3 File — (ZIP) [file pone.0228610.s003.zip › 6_144/Br_II__IR_rec1090.jpg]

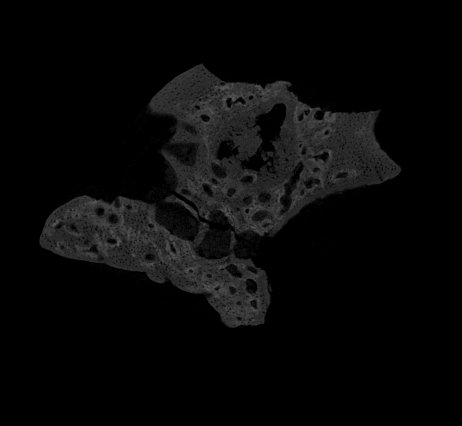

Supplement: S3 File — (ZIP) [file pone.0228610.s003.zip › 6_144/Br_II__IR_rec1094.jpg]

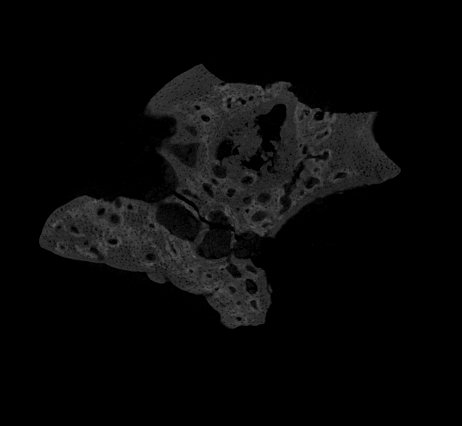

Supplement: S3 File — (ZIP) [file pone.0228610.s003.zip › 6_144/Br_II__IR_rec1098.jpg]

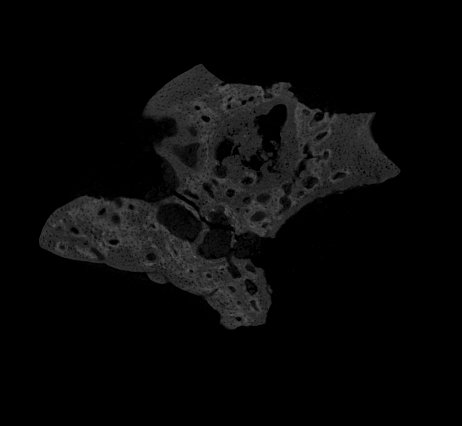

Supplement: S3 File — (ZIP) [file pone.0228610.s003.zip › 6_144/Br_II__IR_rec1102.jpg]

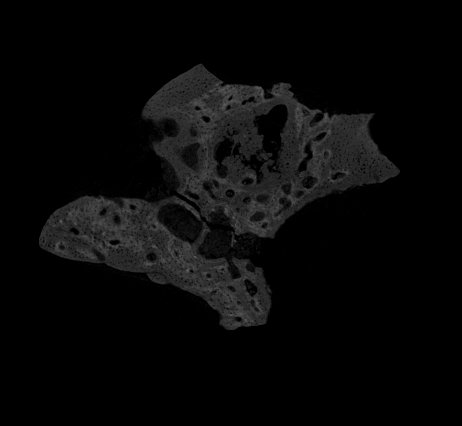

Supplement: S3 File — (ZIP) [file pone.0228610.s003.zip › 6_144/Br_II__IR_rec1106.jpg]

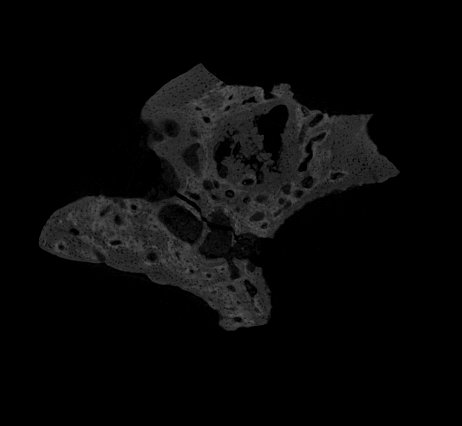

Supplement: S3 File — (ZIP) [file pone.0228610.s003.zip › 6_144/Br_II__IR_rec1110.jpg]

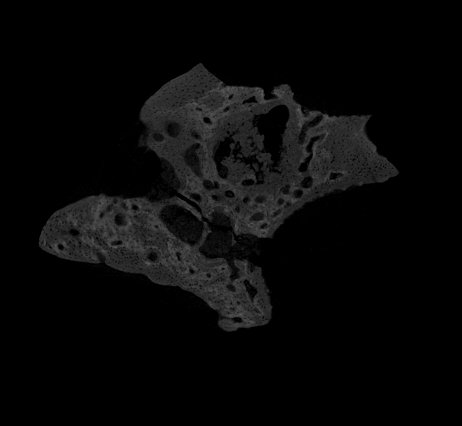

Supplement: S3 File — (ZIP) [file pone.0228610.s003.zip › 6_144/Br_II__IR_rec1114.jpg]

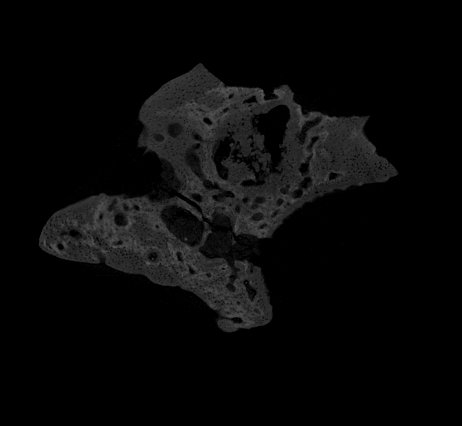

Supplement: S3 File — (ZIP) [file pone.0228610.s003.zip › 6_144/Br_II__IR_rec1118.jpg]

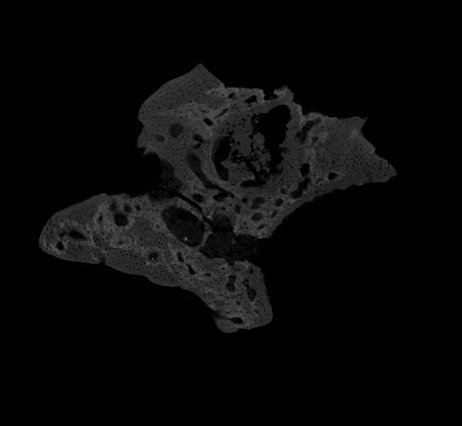

Supplement: S3 File — (ZIP) [file pone.0228610.s003.zip › 6_144/Br_II__IR_rec1122.jpg]

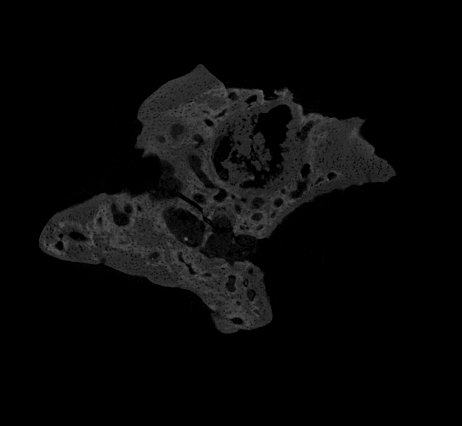

Supplement: S3 File — (ZIP) [file pone.0228610.s003.zip › 6_144/Br_II__IR_rec1126.jpg]

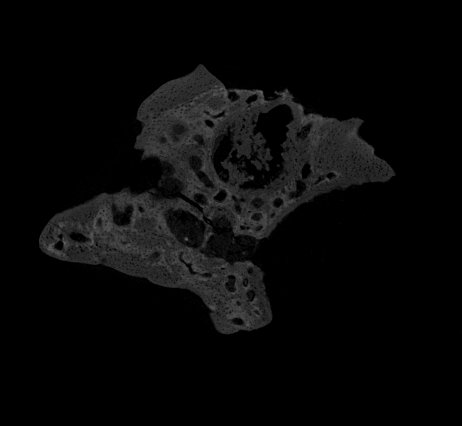

Supplement: S3 File — (ZIP) [file pone.0228610.s003.zip › 6_144/Br_II__IR_rec1130.jpg]

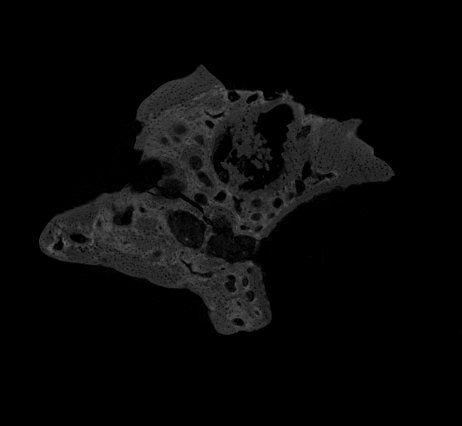

Supplement: S3 File — (ZIP) [file pone.0228610.s003.zip › 6_144/Br_II__IR_rec1134.jpg]

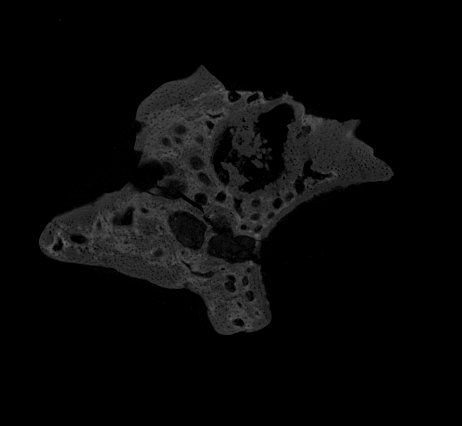

Supplement: S3 File — (ZIP) [file pone.0228610.s003.zip › 6_144/Br_II__IR_rec1138.jpg]

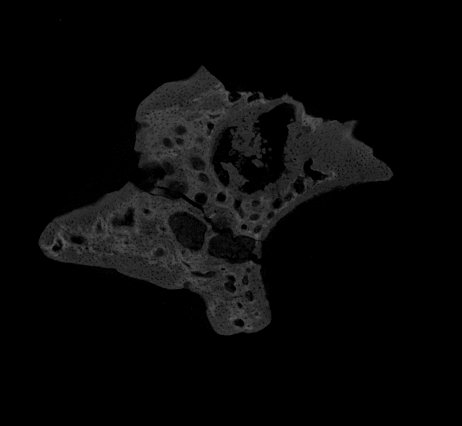

Supplement: S3 File — (ZIP) [file pone.0228610.s003.zip › 6_144/Br_II__IR_rec1142.jpg]

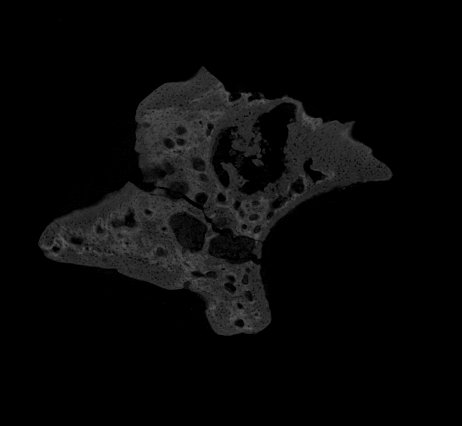

Supplement: S3 File — (ZIP) [file pone.0228610.s003.zip › 6_144/Br_II__IR_rec1146.jpg]

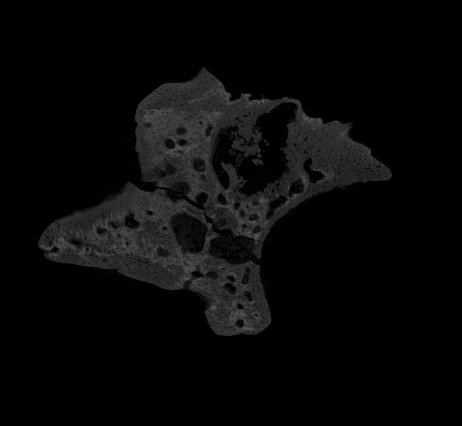

Supplement: S3 File — (ZIP) [file pone.0228610.s003.zip › 6_144/Br_II__IR_rec1150.jpg]

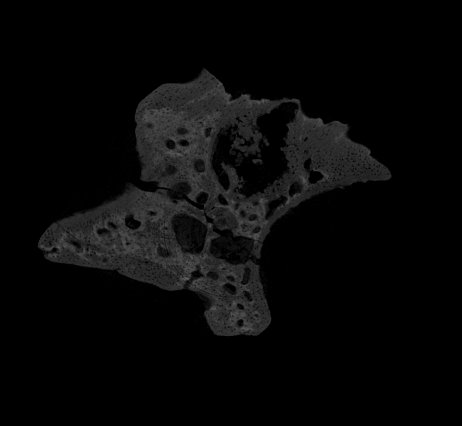

Supplement: S3 File — (ZIP) [file pone.0228610.s003.zip › 6_144/Br_II__IR_rec1154.jpg]

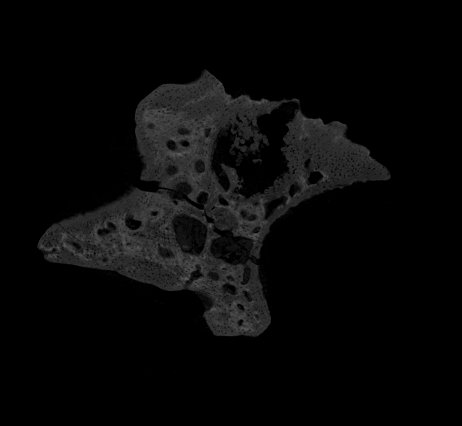

Supplement: S3 File — (ZIP) [file pone.0228610.s003.zip › 6_144/Br_II__IR_rec1158.jpg]

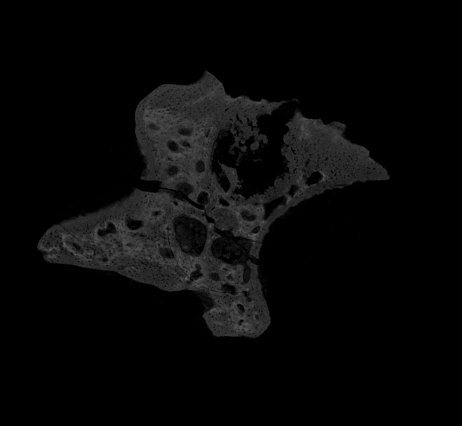

Supplement: S3 File — (ZIP) [file pone.0228610.s003.zip › 6_144/Br_II__IR_rec1162.jpg]

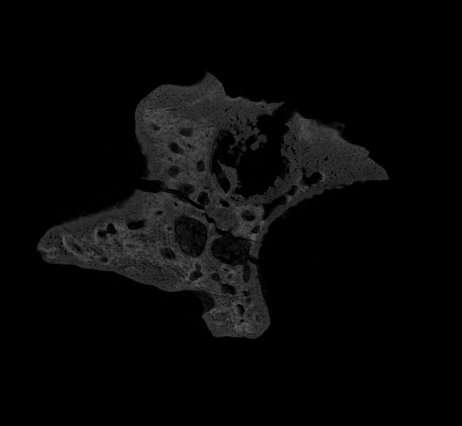

Supplement: S3 File — (ZIP) [file pone.0228610.s003.zip › 6_144/Br_II__IR_rec1166.jpg]

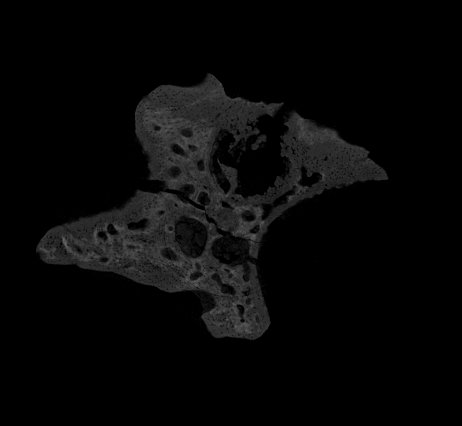

Supplement: S3 File — (ZIP) [file pone.0228610.s003.zip › 6_144/Br_II__IR_rec1170.jpg]

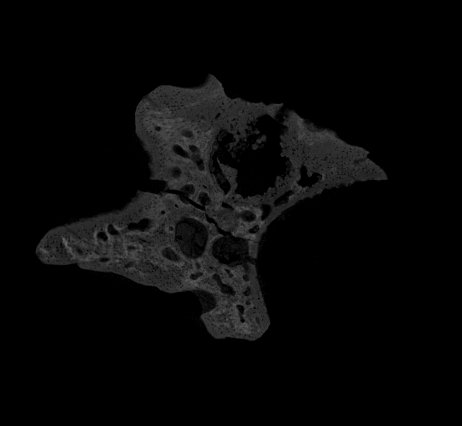

Supplement: S3 File — (ZIP) [file pone.0228610.s003.zip › 6_144/Br_II__IR_rec1174.jpg]

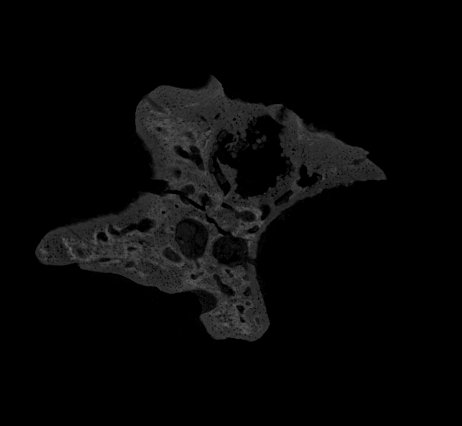

Supplement: S3 File — (ZIP) [file pone.0228610.s003.zip › 6_144/Br_II__IR_rec1178.jpg]

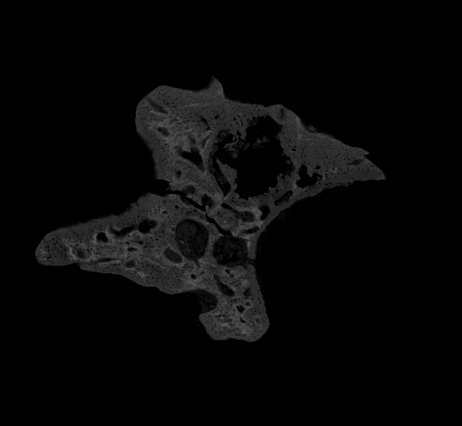

Supplement: S3 File — (ZIP) [file pone.0228610.s003.zip › 6_144/Br_II__IR_rec1182.jpg]

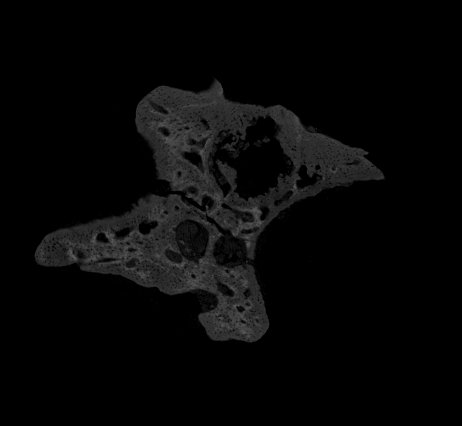

Supplement: S3 File — (ZIP) [file pone.0228610.s003.zip › 6_144/Br_II__IR_rec1186.jpg]

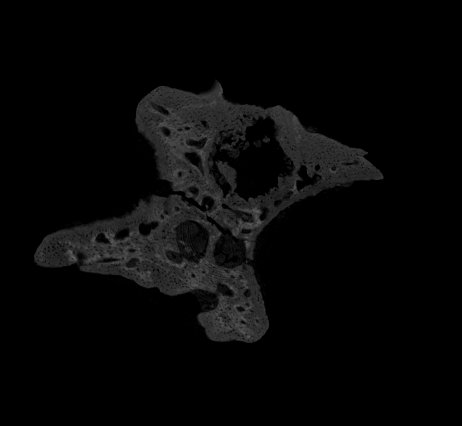

Supplement: S3 File — (ZIP) [file pone.0228610.s003.zip › 6_144/Br_II__IR_rec1190.jpg]

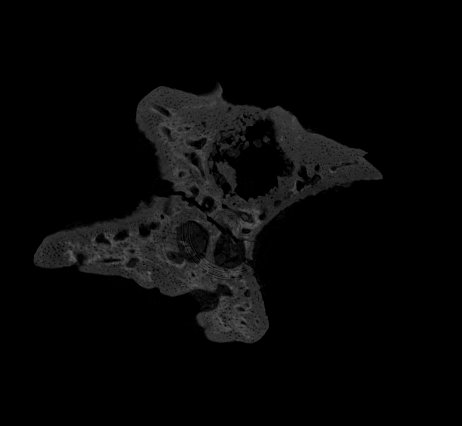

Supplement: S3 File — (ZIP) [file pone.0228610.s003.zip › 6_144/Br_II__IR_rec1194.jpg]

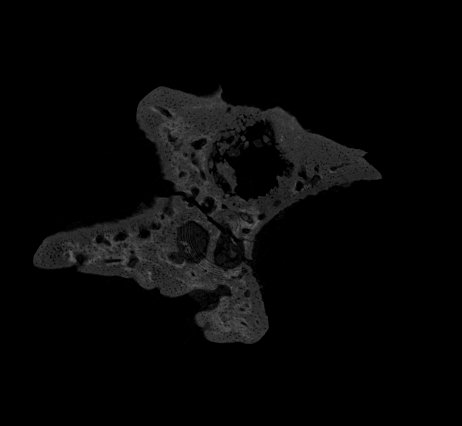

Supplement: S3 File — (ZIP) [file pone.0228610.s003.zip › 6_144/Br_II__IR_rec1198.jpg]

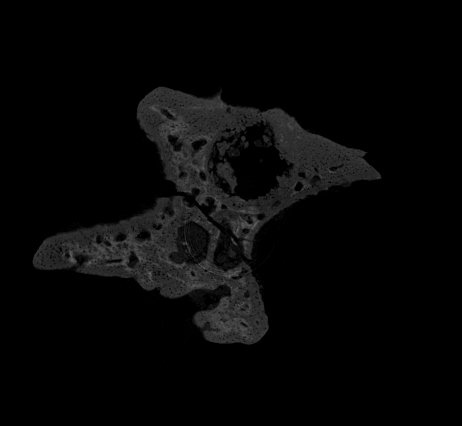

Supplement: S3 File — (ZIP) [file pone.0228610.s003.zip › 6_144/Br_II__IR_rec1202.jpg]

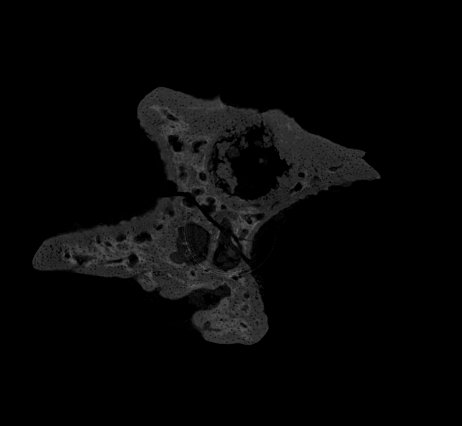

Supplement: S3 File — (ZIP) [file pone.0228610.s003.zip › 6_144/Br_II__IR_rec1206.jpg]

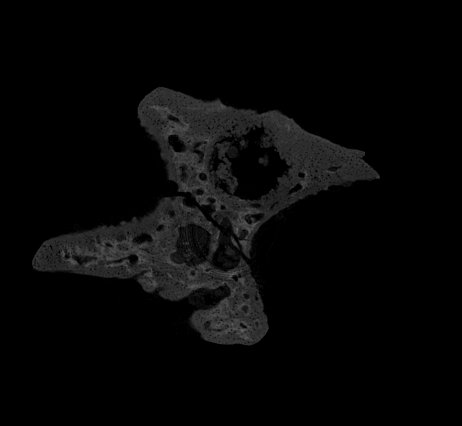

Supplement: S3 File — (ZIP) [file pone.0228610.s003.zip › 6_144/Br_II__IR_rec1210.jpg]

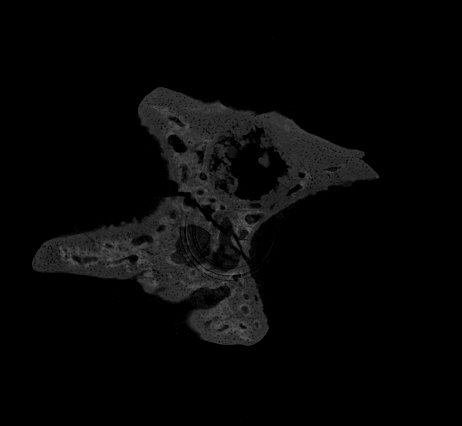

Supplement: S3 File — (ZIP) [file pone.0228610.s003.zip › 6_144/Br_II__IR_rec1214.jpg]

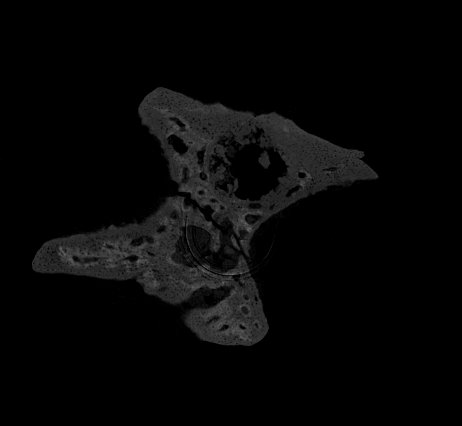

Supplement: S3 File — (ZIP) [file pone.0228610.s003.zip › 6_144/Br_II__IR_rec1218.jpg]

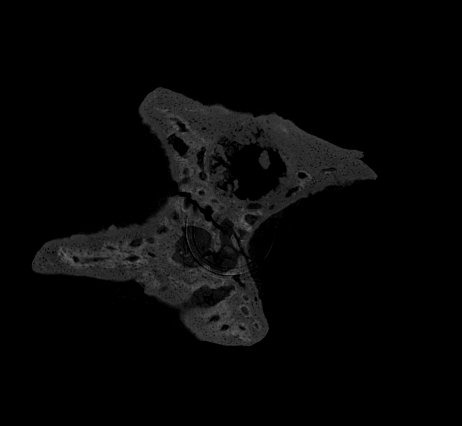

Supplement: S3 File — (ZIP) [file pone.0228610.s003.zip › 6_144/Br_II__IR_rec1222.jpg]

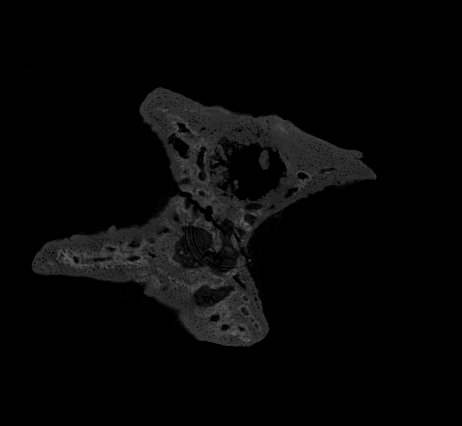

Supplement: S3 File — (ZIP) [file pone.0228610.s003.zip › 6_144/Br_II__IR_rec1226.jpg]

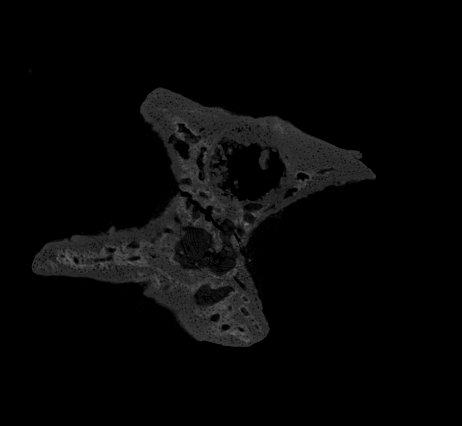

Supplement: S3 File — (ZIP) [file pone.0228610.s003.zip › 6_144/Br_II__IR_rec1230.jpg]

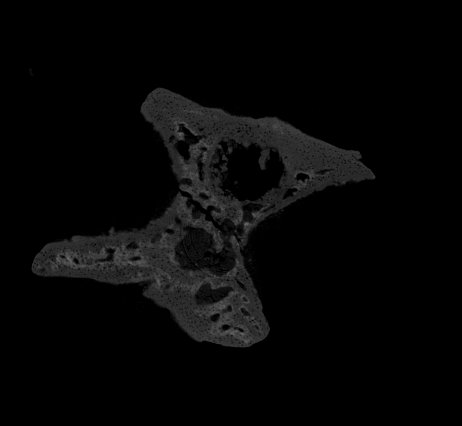

Supplement: S3 File — (ZIP) [file pone.0228610.s003.zip › 6_144/Br_II__IR_rec1234.jpg]

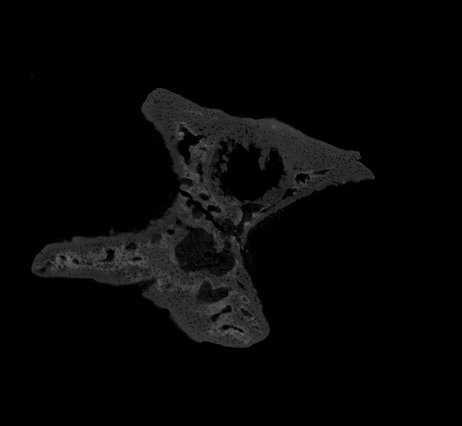

Supplement: S3 File — (ZIP) [file pone.0228610.s003.zip › 6_144/Br_II__IR_rec1238.jpg]

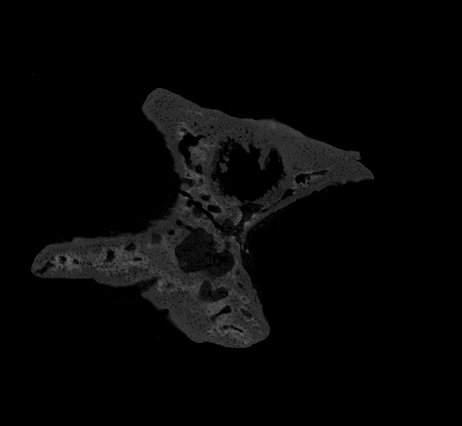

Supplement: S3 File — (ZIP) [file pone.0228610.s003.zip › 6_144/Br_II__IR_rec1242.jpg]

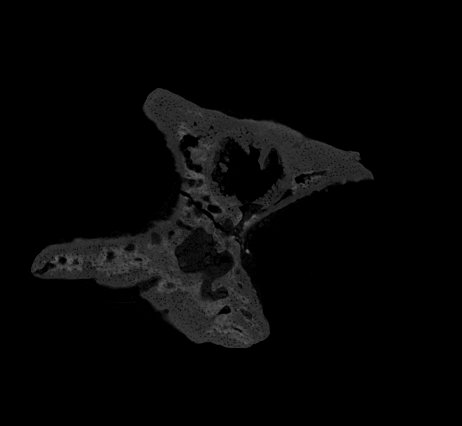

Supplement: S3 File — (ZIP) [file pone.0228610.s003.zip › 6_144/Br_II__IR_rec1246.jpg]

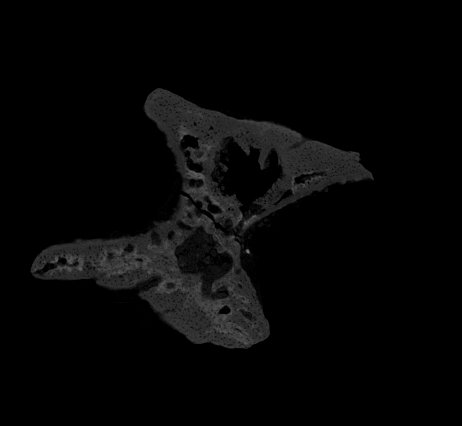

Supplement: S3 File — (ZIP) [file pone.0228610.s003.zip › 6_144/Br_II__IR_rec1250.jpg]

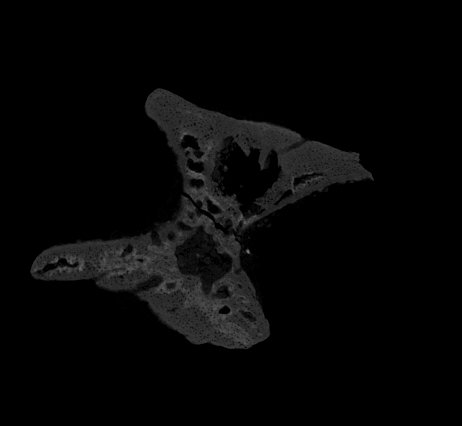

Supplement: S3 File — (ZIP) [file pone.0228610.s003.zip › 6_144/Br_II__IR_rec1254.jpg]

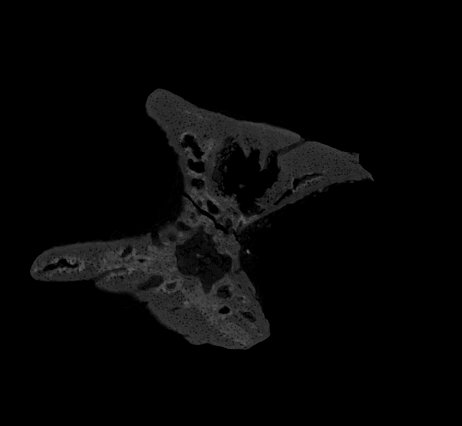

Supplement: S3 File — (ZIP) [file pone.0228610.s003.zip › 6_144/Br_II__IR_rec1258.jpg]

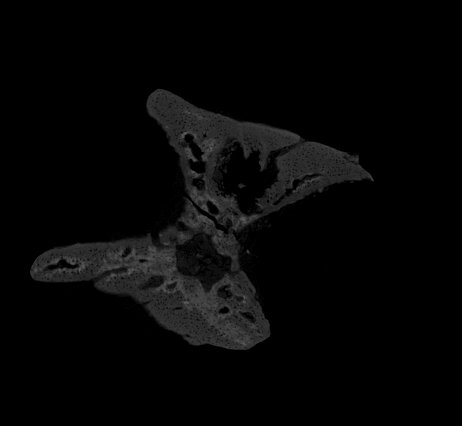

Supplement: S3 File — (ZIP) [file pone.0228610.s003.zip › 6_144/Br_II__IR_rec1262.jpg]

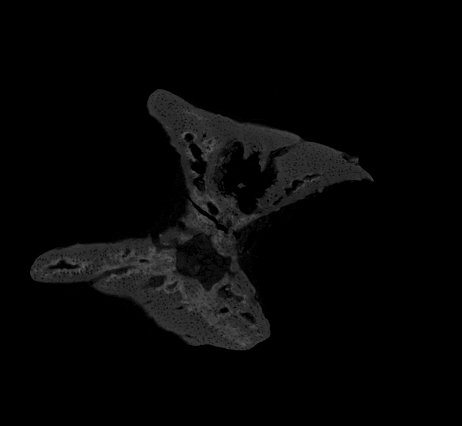

Supplement: S3 File — (ZIP) [file pone.0228610.s003.zip › 6_144/Br_II__IR_rec1266.jpg]

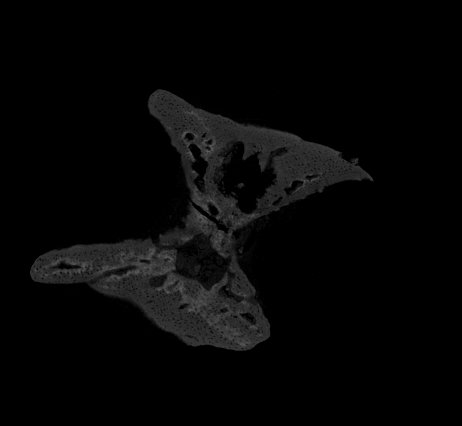

Supplement: S3 File — (ZIP) [file pone.0228610.s003.zip › 6_144/Br_II__IR_rec1270.jpg]

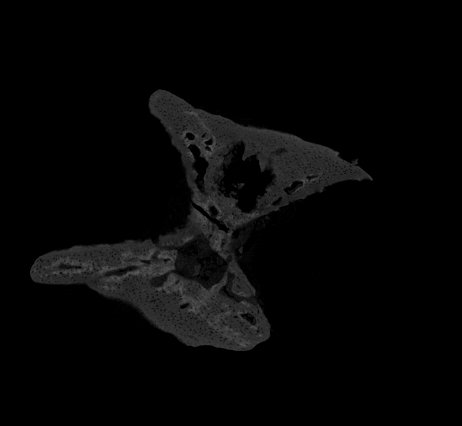

Supplement: S3 File — (ZIP) [file pone.0228610.s003.zip › 6_144/Br_II__IR_rec1274.jpg]

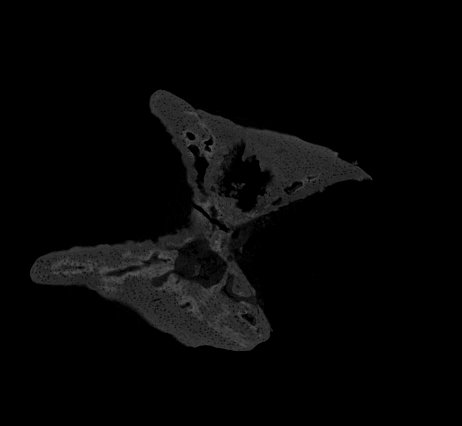

Supplement: S3 File — (ZIP) [file pone.0228610.s003.zip › 6_144/Br_II__IR_rec1278.jpg]

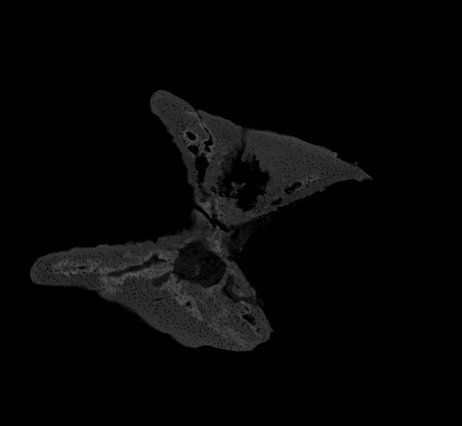

Supplement: S3 File — (ZIP) [file pone.0228610.s003.zip › 6_144/Br_II__IR_rec1282.jpg]

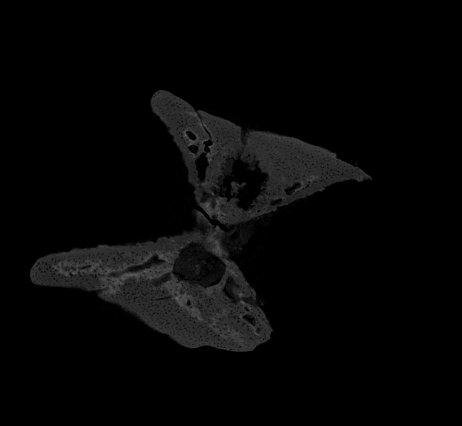

Supplement: S3 File — (ZIP) [file pone.0228610.s003.zip › 6_144/Br_II__IR_rec1286.jpg]

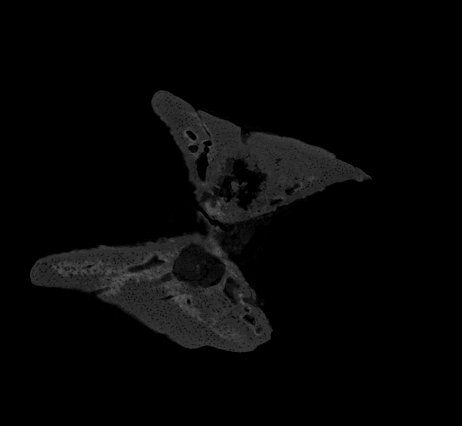

Supplement: S3 File — (ZIP) [file pone.0228610.s003.zip › 6_144/Br_II__IR_rec1290.jpg]

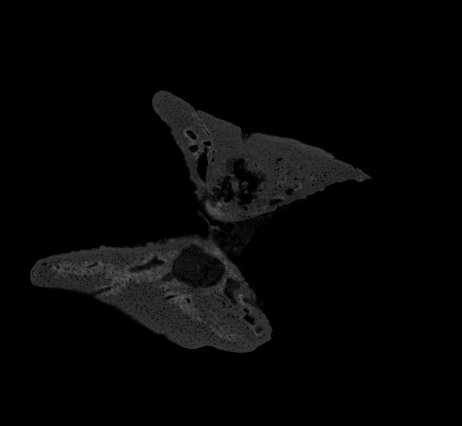

Supplement: S3 File — (ZIP) [file pone.0228610.s003.zip › 6_144/Br_II__IR_rec1294.jpg]

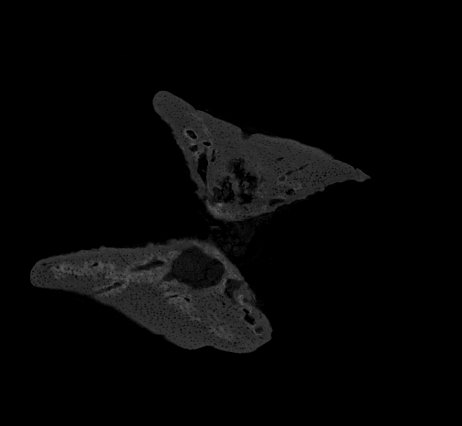

Supplement: S3 File — (ZIP) [file pone.0228610.s003.zip › 6_144/Br_II__IR_rec1298.jpg]

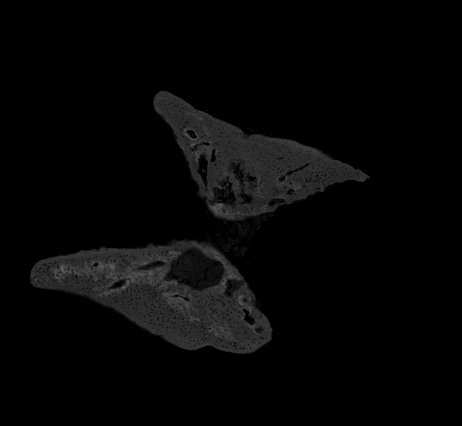

Supplement: S3 File — (ZIP) [file pone.0228610.s003.zip › 6_144/Br_II__IR_rec1302.jpg]

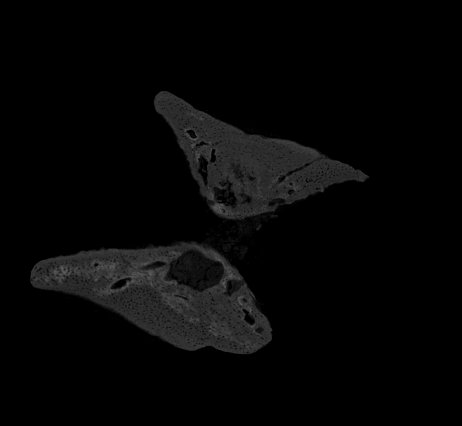

Supplement: S3 File — (ZIP) [file pone.0228610.s003.zip › 6_144/Br_II__IR_rec1306.jpg]

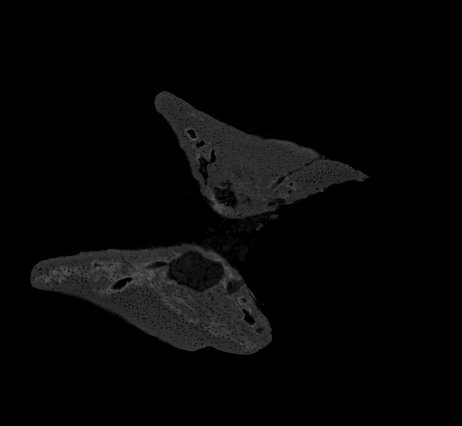

Supplement: S3 File — (ZIP) [file pone.0228610.s003.zip › 6_144/Br_II__IR_rec1310.jpg]

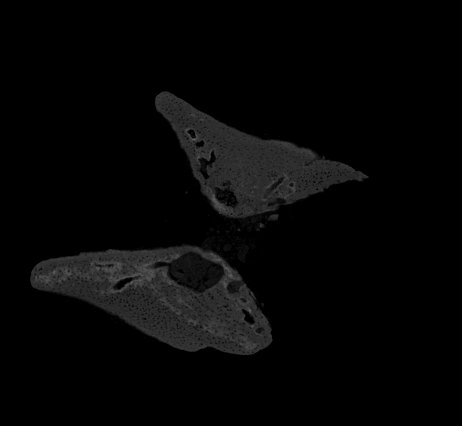

Supplement: S3 File — (ZIP) [file pone.0228610.s003.zip › 6_144/Br_II__IR_rec1314.jpg]

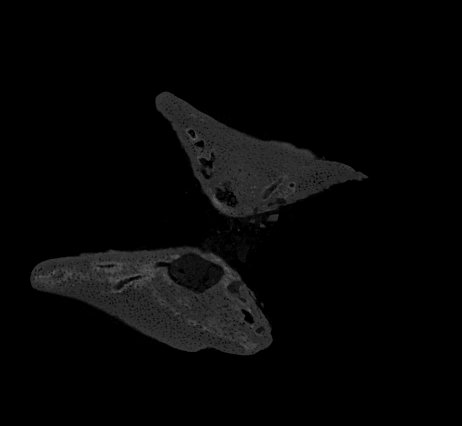

Supplement: S3 File — (ZIP) [file pone.0228610.s003.zip › 6_144/Br_II__IR_rec1318.jpg]

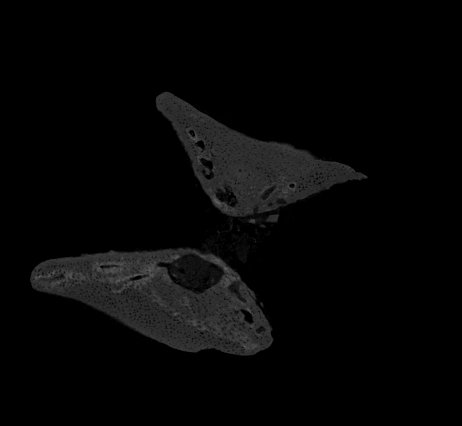

Supplement: S3 File — (ZIP) [file pone.0228610.s003.zip › 6_144/Br_II__IR_rec1322.jpg]

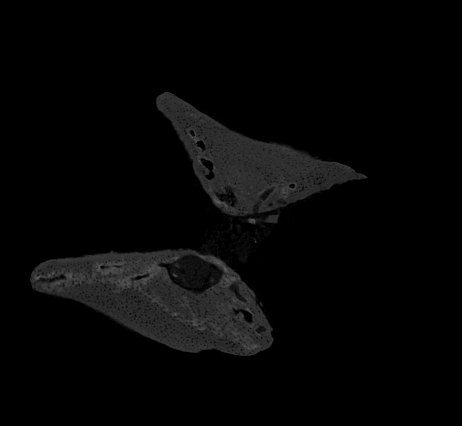

Supplement: S3 File — (ZIP) [file pone.0228610.s003.zip › 6_144/Br_II__IR_rec1326.jpg]

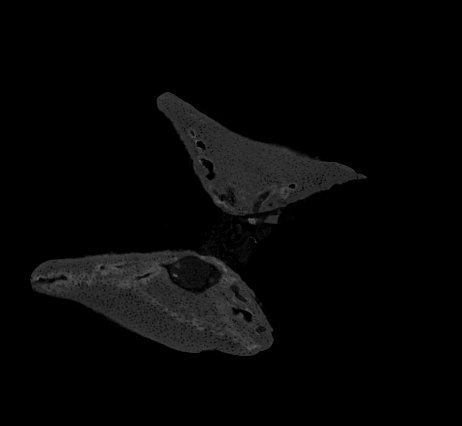

Supplement: S3 File — (ZIP) [file pone.0228610.s003.zip › 6_144/Br_II__IR_rec1330.jpg]

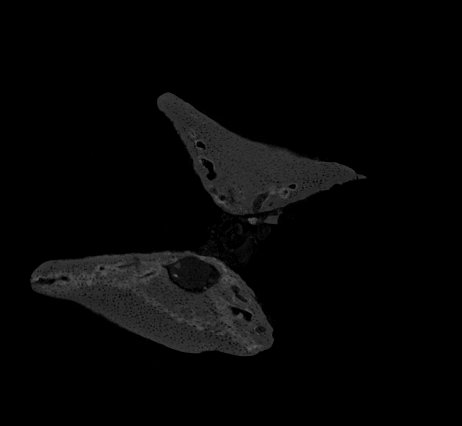

Supplement: S3 File — (ZIP) [file pone.0228610.s003.zip › 6_144/Br_II__IR_rec1334.jpg]

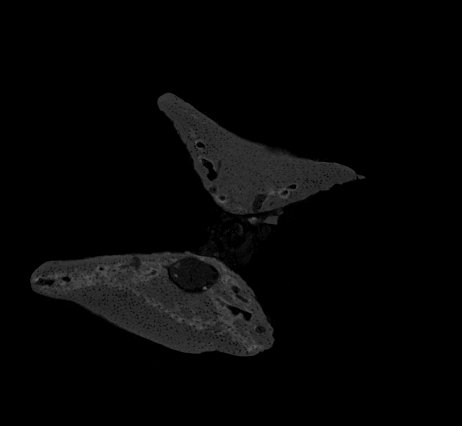

Supplement: S3 File — (ZIP) [file pone.0228610.s003.zip › 6_144/Br_II__IR_rec1338.jpg]

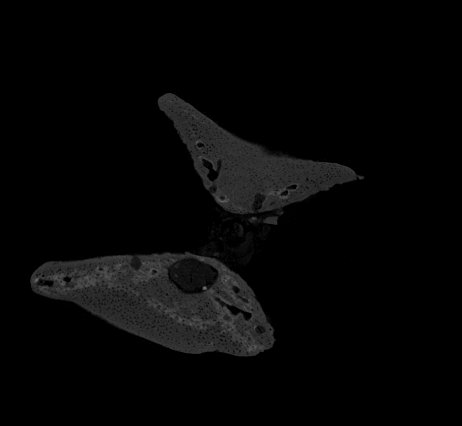

Supplement: S3 File — (ZIP) [file pone.0228610.s003.zip › 6_144/Br_II__IR_rec1342.jpg]

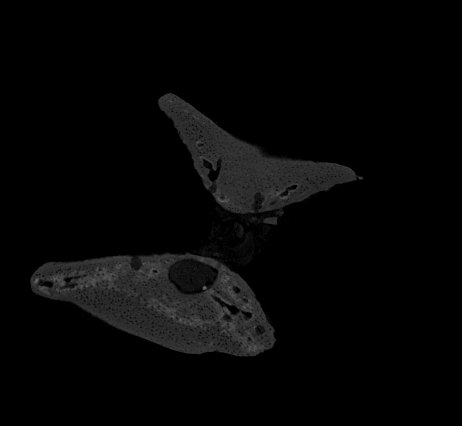

Supplement: S3 File — (ZIP) [file pone.0228610.s003.zip › 6_144/Br_II__IR_rec1346.jpg]

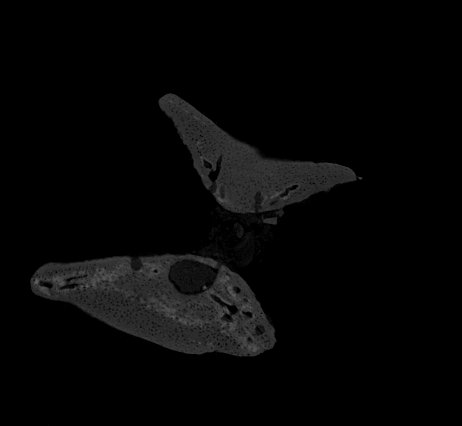

Supplement: S3 File — (ZIP) [file pone.0228610.s003.zip › 6_144/Br_II__IR_rec1350.jpg]

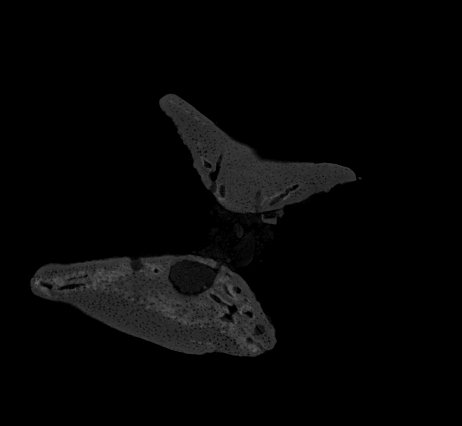

Supplement: S3 File — (ZIP) [file pone.0228610.s003.zip › 6_144/Br_II__IR_rec1354.jpg]

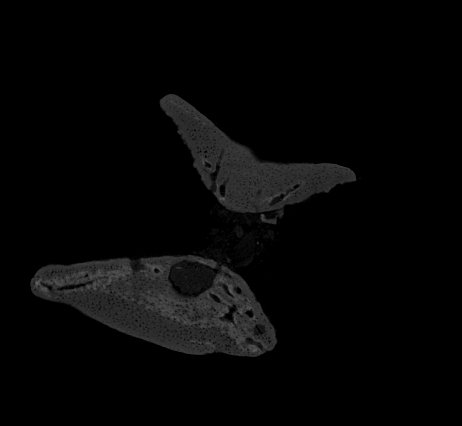

Supplement: S3 File — (ZIP) [file pone.0228610.s003.zip › 6_144/Br_II__IR_rec1358.jpg]

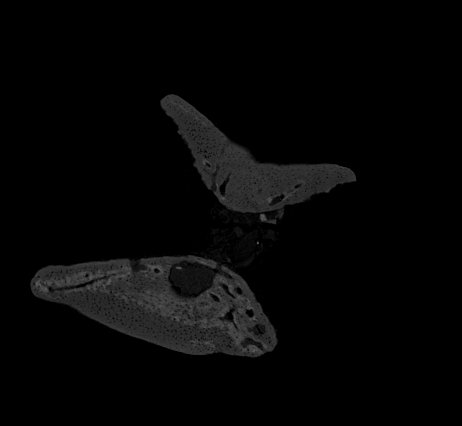

Supplement: S3 File — (ZIP) [file pone.0228610.s003.zip › 6_144/Br_II__IR_rec1362.jpg]

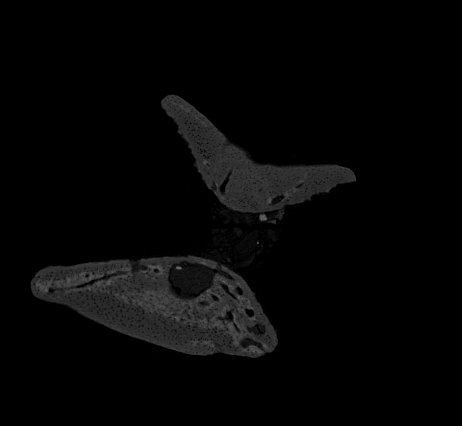

Supplement: S3 File — (ZIP) [file pone.0228610.s003.zip › 6_144/Br_II__IR_rec1366.jpg]

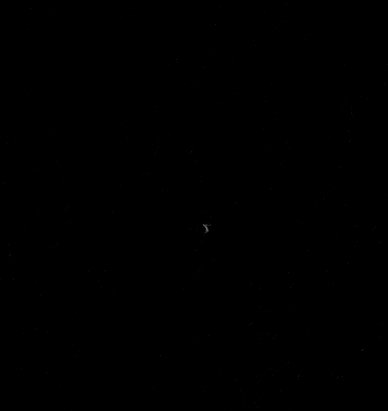

Supplement: S4 File — (ZIP) [file pone.0228610.s004.zip › 29_144/BrI_IR_rec0265.jpg]

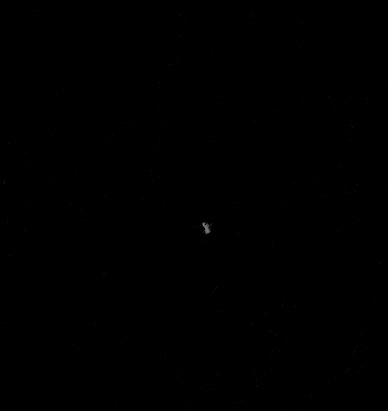

Supplement: S4 File — (ZIP) [file pone.0228610.s004.zip › 29_144/BrI_IR_rec0269.jpg]

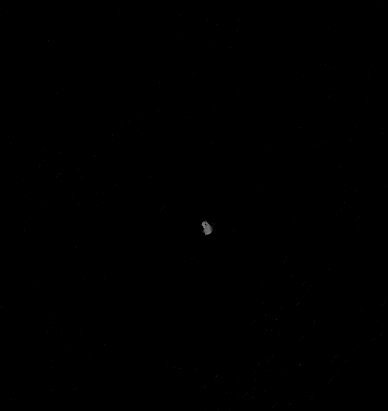

Supplement: S4 File — (ZIP) [file pone.0228610.s004.zip › 29_144/BrI_IR_rec0273.jpg]

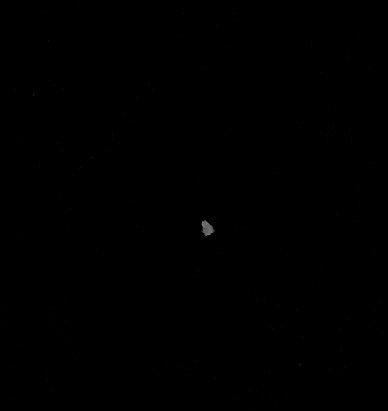

Supplement: S4 File — (ZIP) [file pone.0228610.s004.zip › 29_144/BrI_IR_rec0277.jpg]

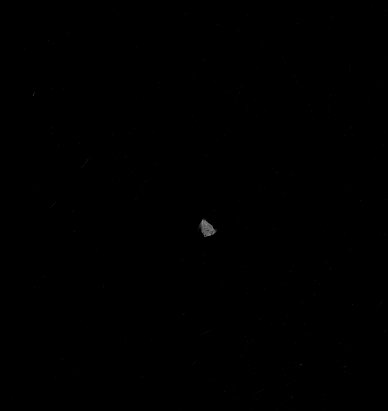

Supplement: S4 File — (ZIP) [file pone.0228610.s004.zip › 29_144/BrI_IR_rec0281.jpg]

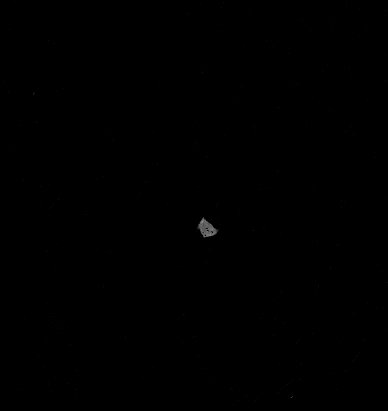

Supplement: S4 File — (ZIP) [file pone.0228610.s004.zip › 29_144/BrI_IR_rec0285.jpg]

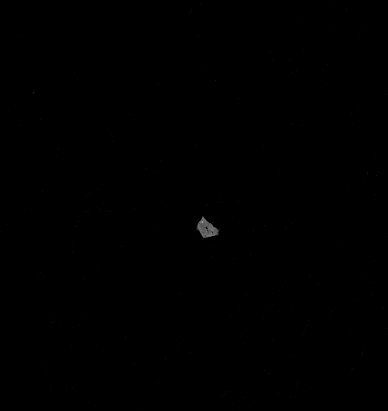

Supplement: S4 File — (ZIP) [file pone.0228610.s004.zip › 29_144/BrI_IR_rec0289.jpg]

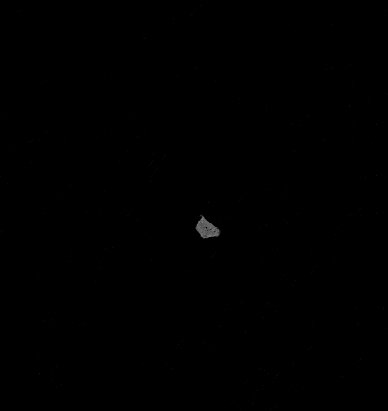

Supplement: S4 File — (ZIP) [file pone.0228610.s004.zip › 29_144/BrI_IR_rec0293.jpg]

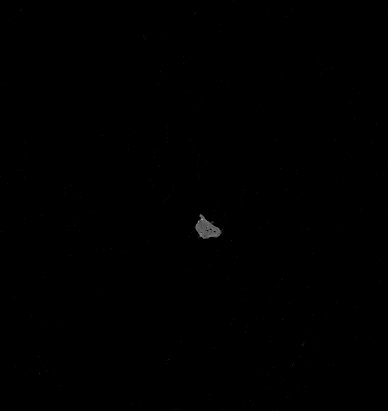

Supplement: S4 File — (ZIP) [file pone.0228610.s004.zip › 29_144/BrI_IR_rec0297.jpg]

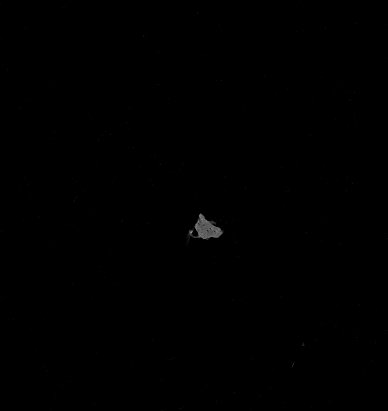

Supplement: S4 File — (ZIP) [file pone.0228610.s004.zip › 29_144/BrI_IR_rec0301.jpg]

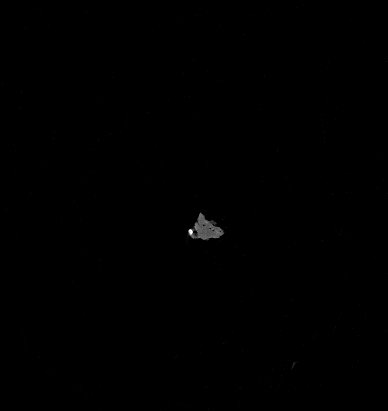

Supplement: S4 File — (ZIP) [file pone.0228610.s004.zip › 29_144/BrI_IR_rec0305.jpg]

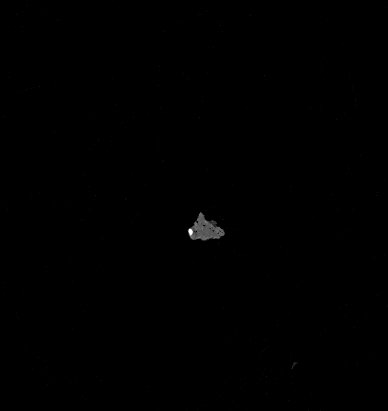

Supplement: S4 File — (ZIP) [file pone.0228610.s004.zip › 29_144/BrI_IR_rec0309.jpg]

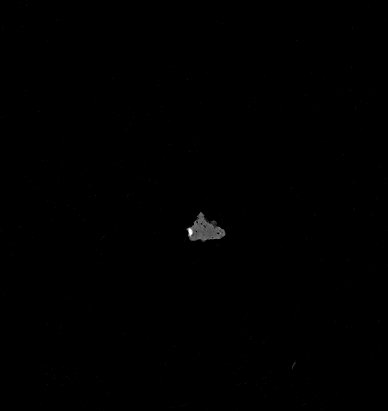

Supplement: S4 File — (ZIP) [file pone.0228610.s004.zip › 29_144/BrI_IR_rec0313.jpg]

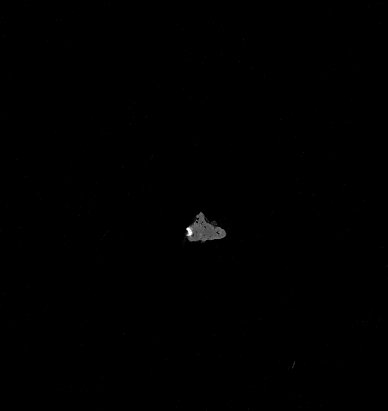

Supplement: S4 File — (ZIP) [file pone.0228610.s004.zip › 29_144/BrI_IR_rec0317.jpg]

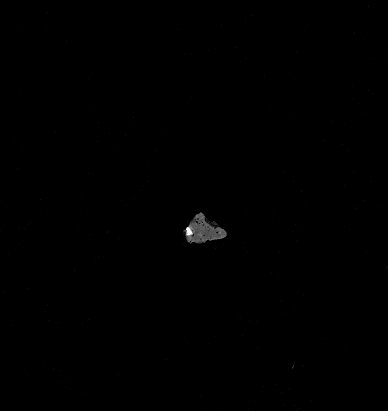

Supplement: S4 File — (ZIP) [file pone.0228610.s004.zip › 29_144/BrI_IR_rec0321.jpg]

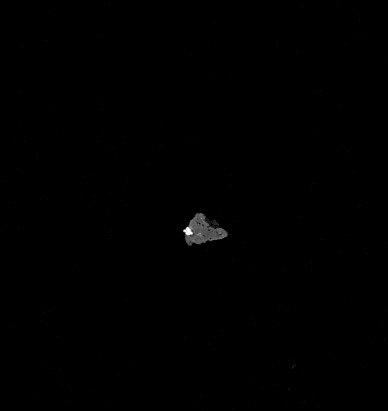

Supplement: S4 File — (ZIP) [file pone.0228610.s004.zip › 29_144/BrI_IR_rec0325.jpg]

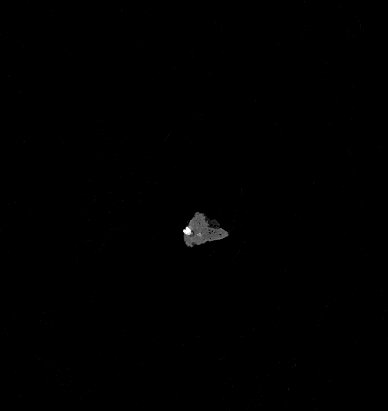

Supplement: S4 File — (ZIP) [file pone.0228610.s004.zip › 29_144/BrI_IR_rec0329.jpg]

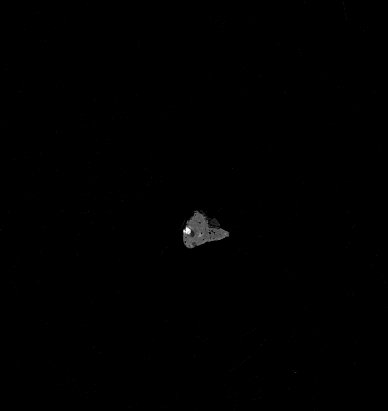

Supplement: S4 File — (ZIP) [file pone.0228610.s004.zip › 29_144/BrI_IR_rec0333.jpg]

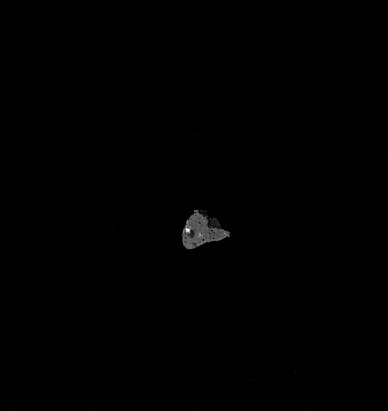

Supplement: S4 File — (ZIP) [file pone.0228610.s004.zip › 29_144/BrI_IR_rec0337.jpg]

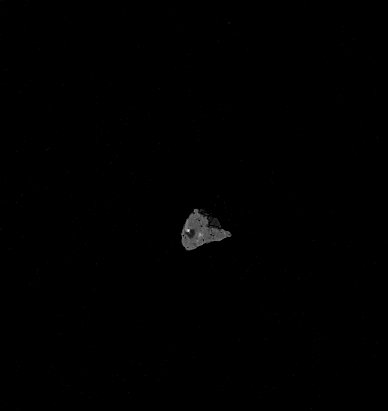

Supplement: S4 File — (ZIP) [file pone.0228610.s004.zip › 29_144/BrI_IR_rec0341.jpg]

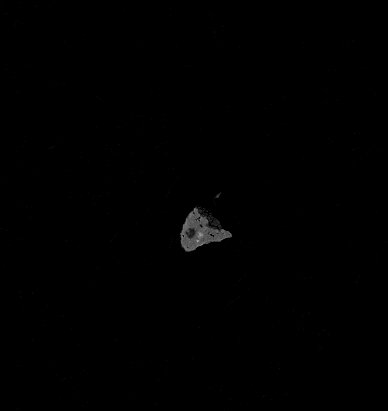

Supplement: S4 File — (ZIP) [file pone.0228610.s004.zip › 29_144/BrI_IR_rec0345.jpg]
