# Supplementary figures and images for: A new small-sized stem salamander from the Middle Jurassic of Western Siberia, Russia (part 8 of 10)
Source: PLoS One. 2020 Feb 19;15(2):e0228610. doi: 10.1371/journal.pone.0228610 (PMC7029856; doi:10.1371/journal.pone.0228610)

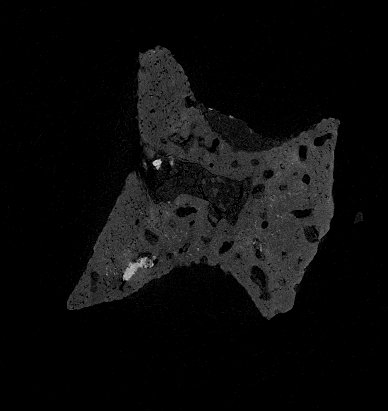

Supplement: S4 File — (ZIP) [file pone.0228610.s004.zip › 29_144/BrI_IR_rec0749.jpg]

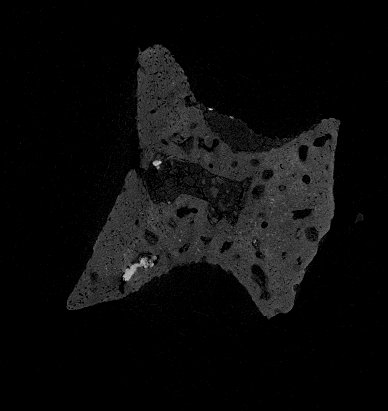

Supplement: S4 File — (ZIP) [file pone.0228610.s004.zip › 29_144/BrI_IR_rec0753.jpg]

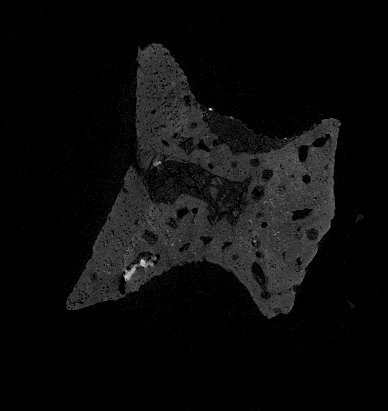

Supplement: S4 File — (ZIP) [file pone.0228610.s004.zip › 29_144/BrI_IR_rec0757.jpg]

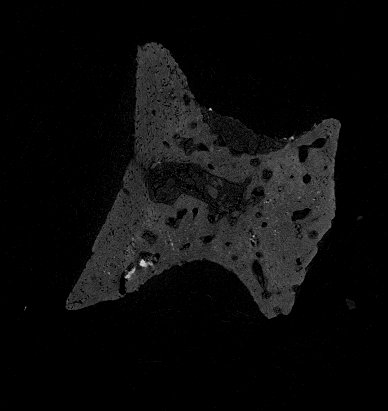

Supplement: S4 File — (ZIP) [file pone.0228610.s004.zip › 29_144/BrI_IR_rec0761.jpg]

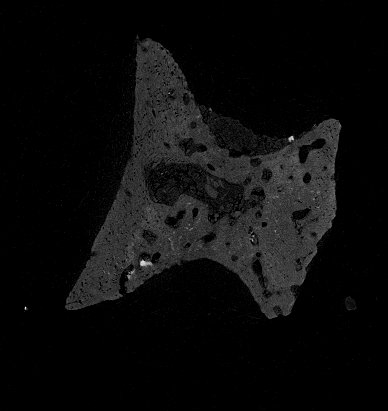

Supplement: S4 File — (ZIP) [file pone.0228610.s004.zip › 29_144/BrI_IR_rec0765.jpg]

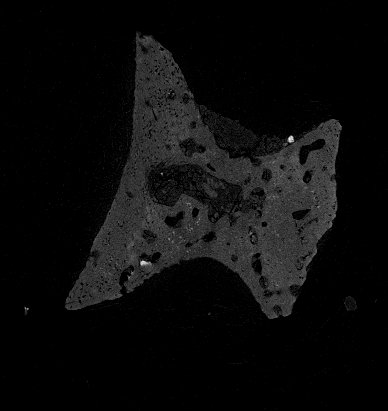

Supplement: S4 File — (ZIP) [file pone.0228610.s004.zip › 29_144/BrI_IR_rec0769.jpg]

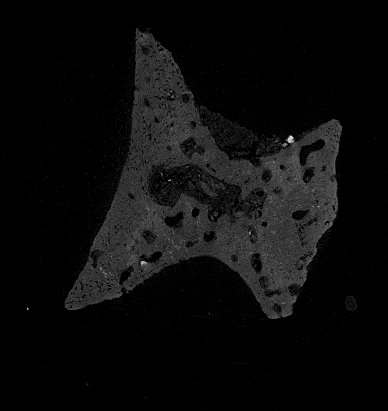

Supplement: S4 File — (ZIP) [file pone.0228610.s004.zip › 29_144/BrI_IR_rec0773.jpg]

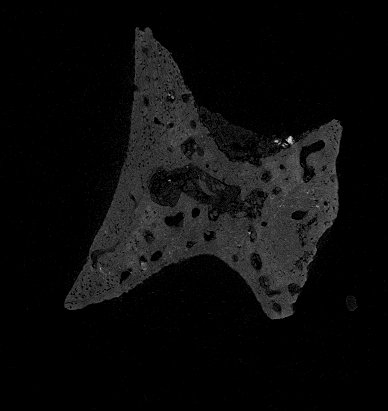

Supplement: S4 File — (ZIP) [file pone.0228610.s004.zip › 29_144/BrI_IR_rec0777.jpg]

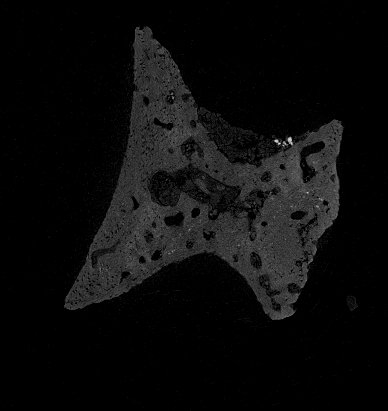

Supplement: S4 File — (ZIP) [file pone.0228610.s004.zip › 29_144/BrI_IR_rec0781.jpg]

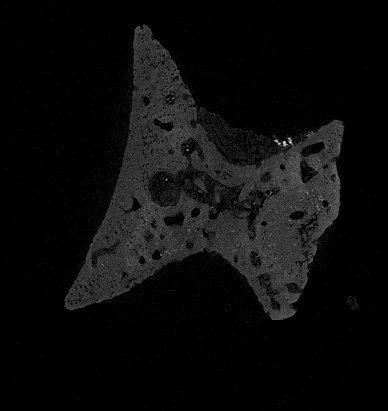

Supplement: S4 File — (ZIP) [file pone.0228610.s004.zip › 29_144/BrI_IR_rec0785.jpg]

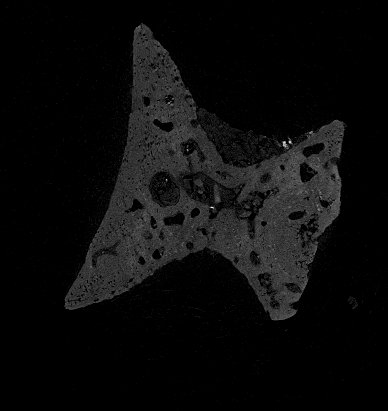

Supplement: S4 File — (ZIP) [file pone.0228610.s004.zip › 29_144/BrI_IR_rec0789.jpg]

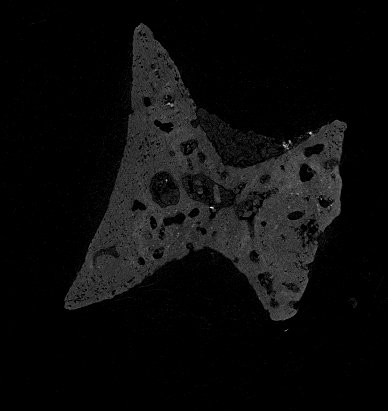

Supplement: S4 File — (ZIP) [file pone.0228610.s004.zip › 29_144/BrI_IR_rec0793.jpg]

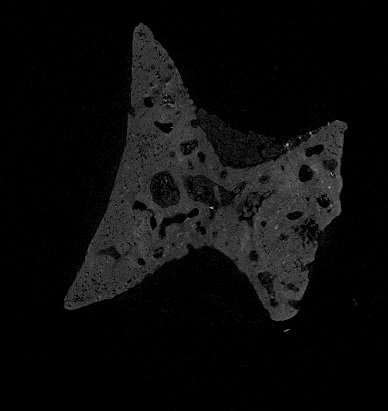

Supplement: S4 File — (ZIP) [file pone.0228610.s004.zip › 29_144/BrI_IR_rec0797.jpg]

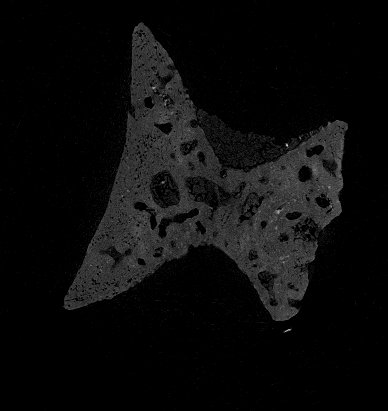

Supplement: S4 File — (ZIP) [file pone.0228610.s004.zip › 29_144/BrI_IR_rec0801.jpg]

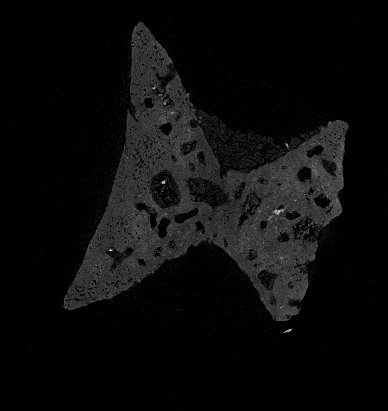

Supplement: S4 File — (ZIP) [file pone.0228610.s004.zip › 29_144/BrI_IR_rec0805.jpg]

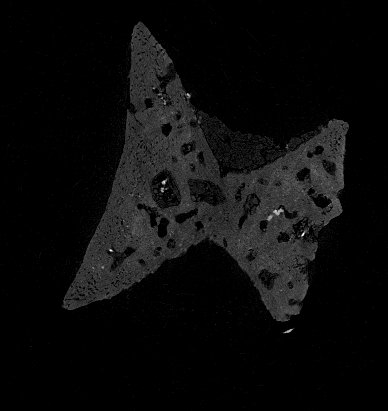

Supplement: S4 File — (ZIP) [file pone.0228610.s004.zip › 29_144/BrI_IR_rec0809.jpg]

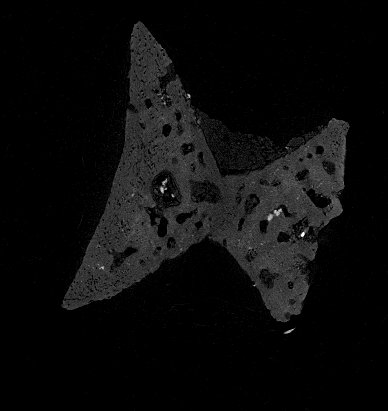

Supplement: S4 File — (ZIP) [file pone.0228610.s004.zip › 29_144/BrI_IR_rec0813.jpg]

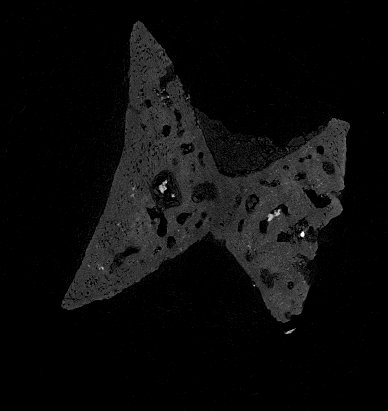

Supplement: S4 File — (ZIP) [file pone.0228610.s004.zip › 29_144/BrI_IR_rec0817.jpg]

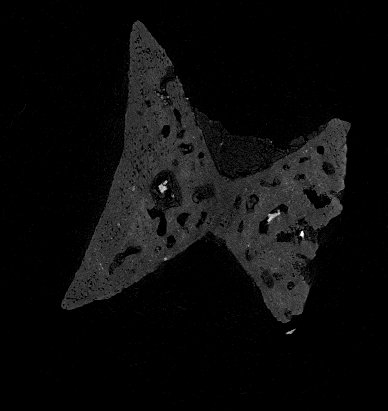

Supplement: S4 File — (ZIP) [file pone.0228610.s004.zip › 29_144/BrI_IR_rec0821.jpg]

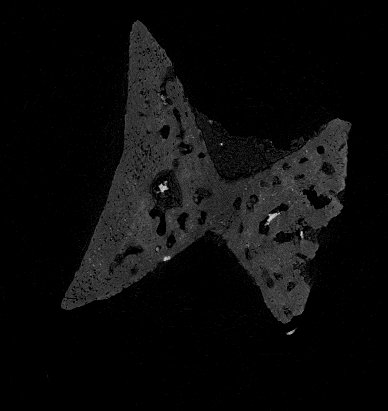

Supplement: S4 File — (ZIP) [file pone.0228610.s004.zip › 29_144/BrI_IR_rec0825.jpg]

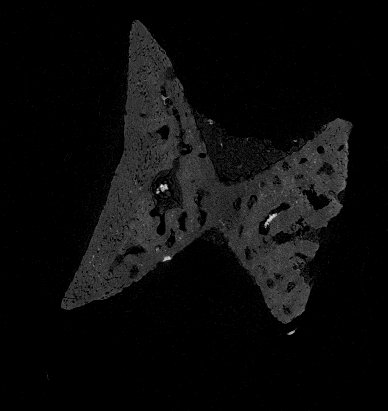

Supplement: S4 File — (ZIP) [file pone.0228610.s004.zip › 29_144/BrI_IR_rec0829.jpg]

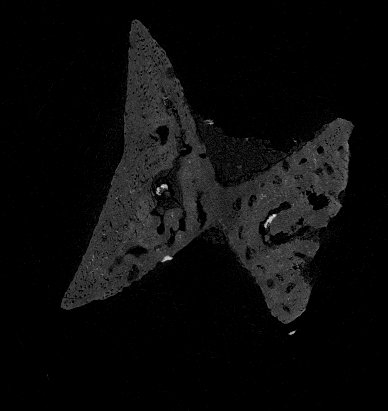

Supplement: S4 File — (ZIP) [file pone.0228610.s004.zip › 29_144/BrI_IR_rec0833.jpg]

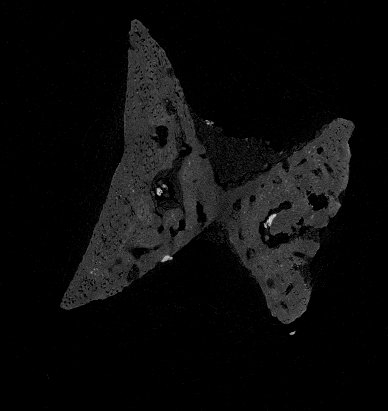

Supplement: S4 File — (ZIP) [file pone.0228610.s004.zip › 29_144/BrI_IR_rec0837.jpg]

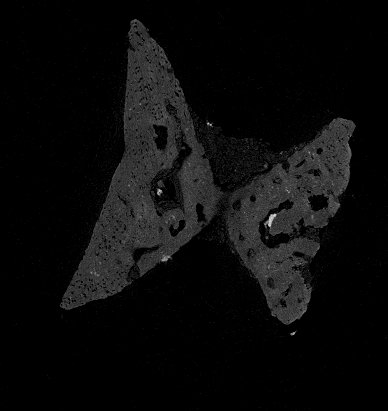

Supplement: S4 File — (ZIP) [file pone.0228610.s004.zip › 29_144/BrI_IR_rec0841.jpg]

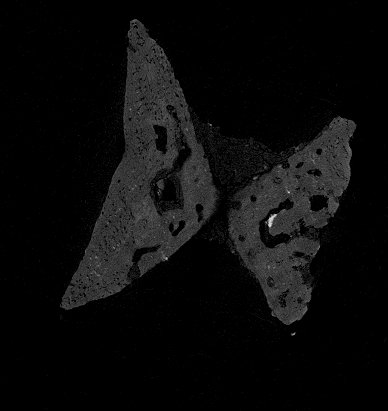

Supplement: S4 File — (ZIP) [file pone.0228610.s004.zip › 29_144/BrI_IR_rec0845.jpg]

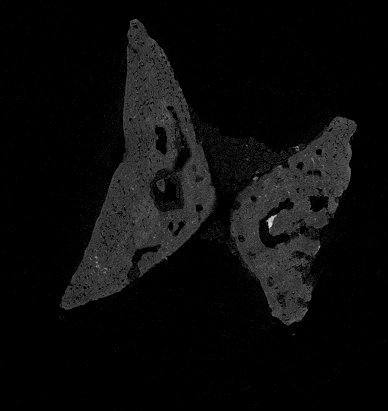

Supplement: S4 File — (ZIP) [file pone.0228610.s004.zip › 29_144/BrI_IR_rec0849.jpg]

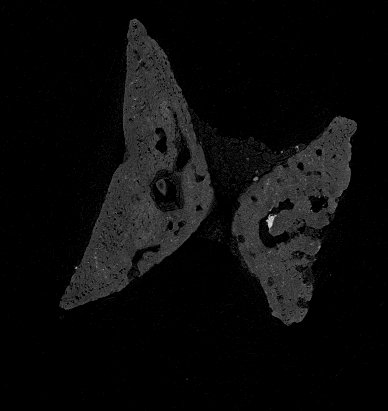

Supplement: S4 File — (ZIP) [file pone.0228610.s004.zip › 29_144/BrI_IR_rec0853.jpg]

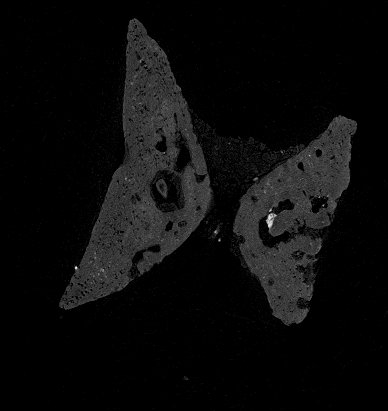

Supplement: S4 File — (ZIP) [file pone.0228610.s004.zip › 29_144/BrI_IR_rec0857.jpg]

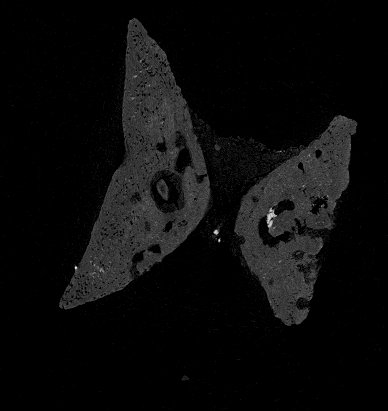

Supplement: S4 File — (ZIP) [file pone.0228610.s004.zip › 29_144/BrI_IR_rec0861.jpg]

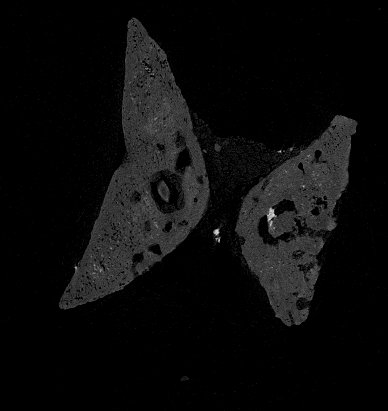

Supplement: S4 File — (ZIP) [file pone.0228610.s004.zip › 29_144/BrI_IR_rec0865.jpg]

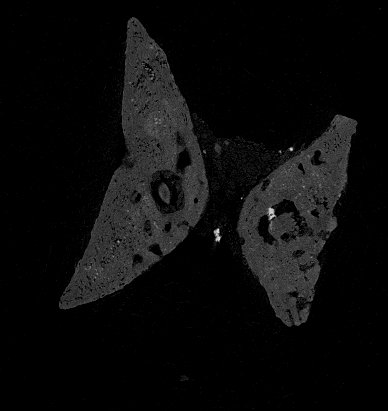

Supplement: S4 File — (ZIP) [file pone.0228610.s004.zip › 29_144/BrI_IR_rec0869.jpg]

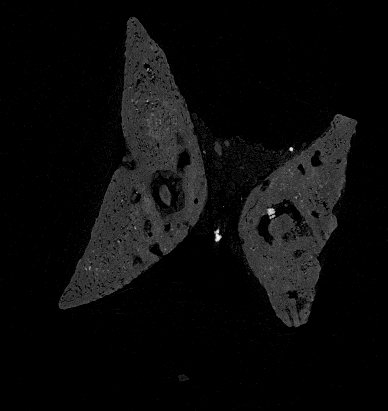

Supplement: S4 File — (ZIP) [file pone.0228610.s004.zip › 29_144/BrI_IR_rec0873.jpg]

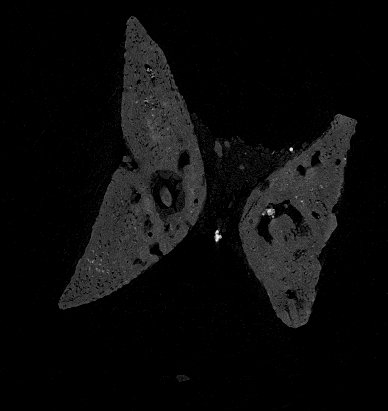

Supplement: S4 File — (ZIP) [file pone.0228610.s004.zip › 29_144/BrI_IR_rec0877.jpg]

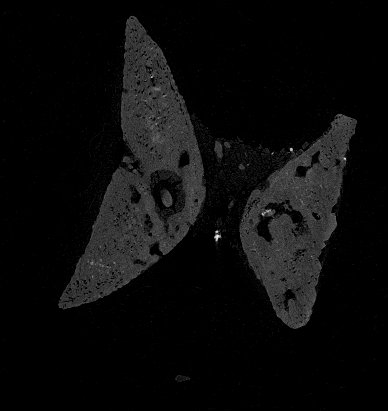

Supplement: S4 File — (ZIP) [file pone.0228610.s004.zip › 29_144/BrI_IR_rec0881.jpg]

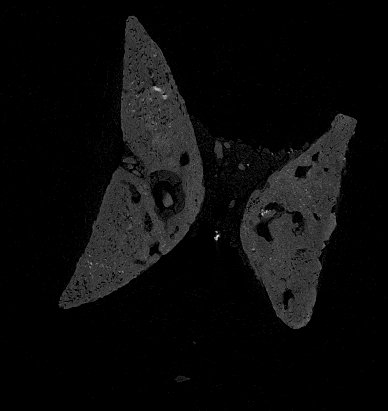

Supplement: S4 File — (ZIP) [file pone.0228610.s004.zip › 29_144/BrI_IR_rec0885.jpg]

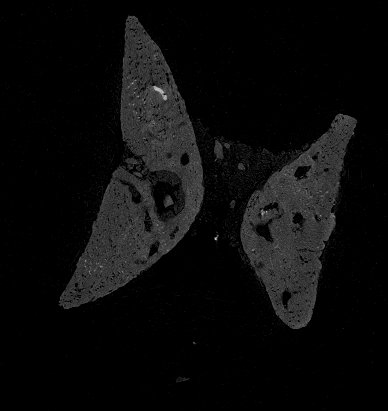

Supplement: S4 File — (ZIP) [file pone.0228610.s004.zip › 29_144/BrI_IR_rec0889.jpg]

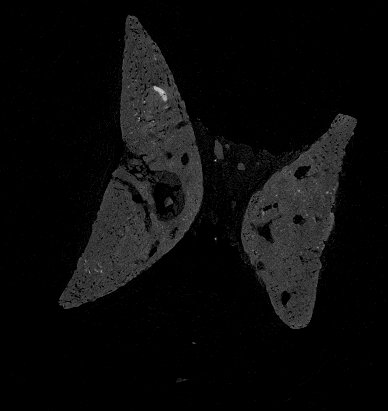

Supplement: S4 File — (ZIP) [file pone.0228610.s004.zip › 29_144/BrI_IR_rec0893.jpg]

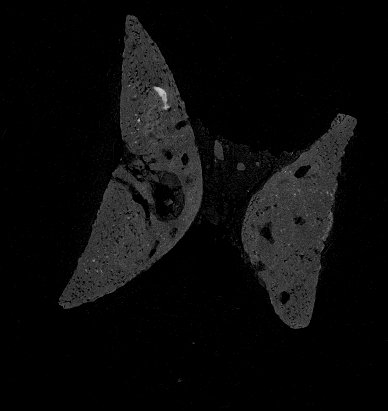

Supplement: S4 File — (ZIP) [file pone.0228610.s004.zip › 29_144/BrI_IR_rec0897.jpg]

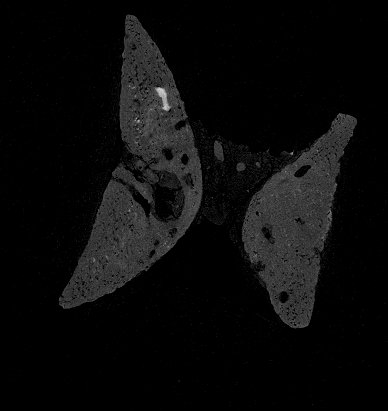

Supplement: S4 File — (ZIP) [file pone.0228610.s004.zip › 29_144/BrI_IR_rec0901.jpg]

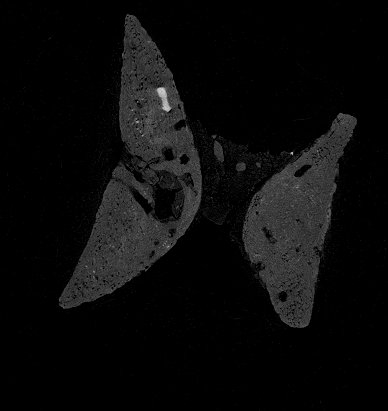

Supplement: S4 File — (ZIP) [file pone.0228610.s004.zip › 29_144/BrI_IR_rec0905.jpg]

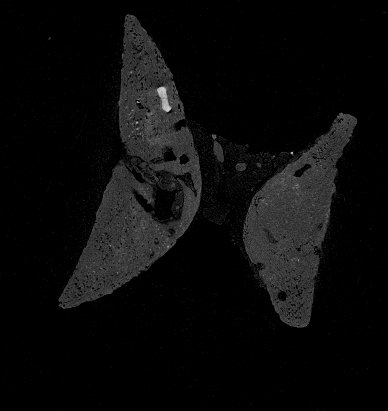

Supplement: S4 File — (ZIP) [file pone.0228610.s004.zip › 29_144/BrI_IR_rec0909.jpg]

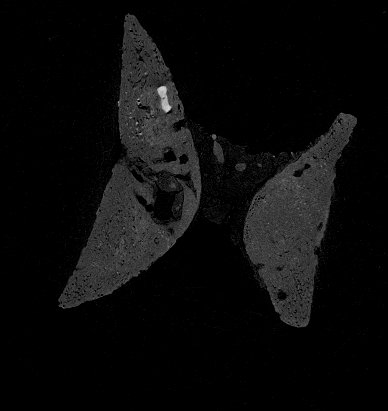

Supplement: S4 File — (ZIP) [file pone.0228610.s004.zip › 29_144/BrI_IR_rec0913.jpg]

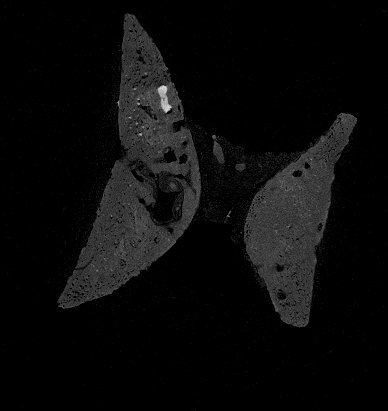

Supplement: S4 File — (ZIP) [file pone.0228610.s004.zip › 29_144/BrI_IR_rec0917.jpg]

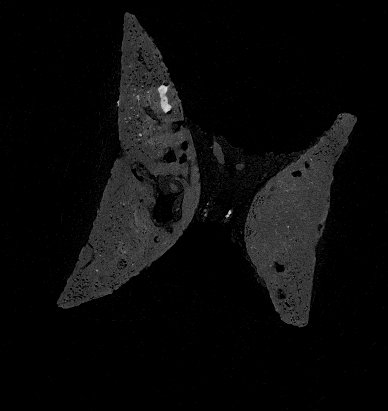

Supplement: S4 File — (ZIP) [file pone.0228610.s004.zip › 29_144/BrI_IR_rec0921.jpg]

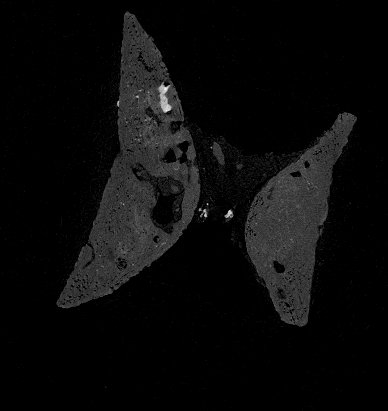

Supplement: S4 File — (ZIP) [file pone.0228610.s004.zip › 29_144/BrI_IR_rec0925.jpg]

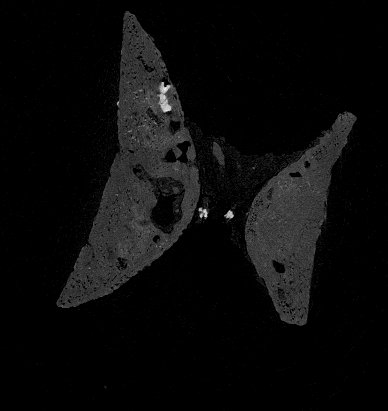

Supplement: S4 File — (ZIP) [file pone.0228610.s004.zip › 29_144/BrI_IR_rec0929.jpg]

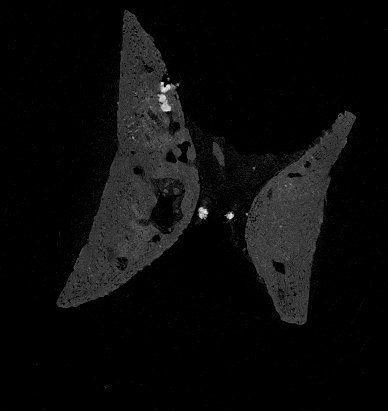

Supplement: S4 File — (ZIP) [file pone.0228610.s004.zip › 29_144/BrI_IR_rec0933.jpg]

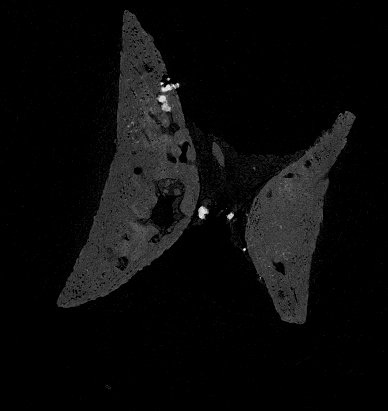

Supplement: S4 File — (ZIP) [file pone.0228610.s004.zip › 29_144/BrI_IR_rec0937.jpg]

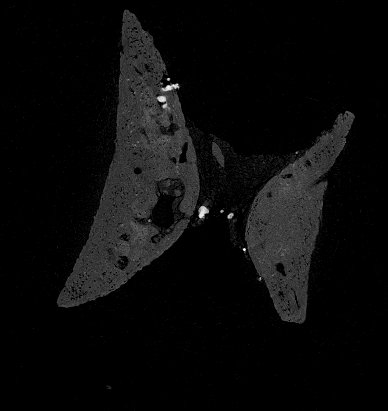

Supplement: S4 File — (ZIP) [file pone.0228610.s004.zip › 29_144/BrI_IR_rec0941.jpg]

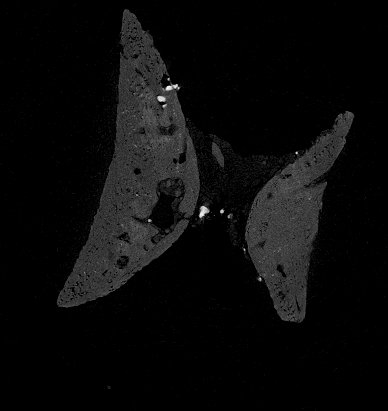

Supplement: S4 File — (ZIP) [file pone.0228610.s004.zip › 29_144/BrI_IR_rec0945.jpg]

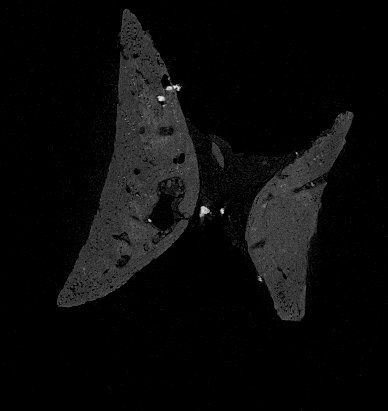

Supplement: S4 File — (ZIP) [file pone.0228610.s004.zip › 29_144/BrI_IR_rec0949.jpg]

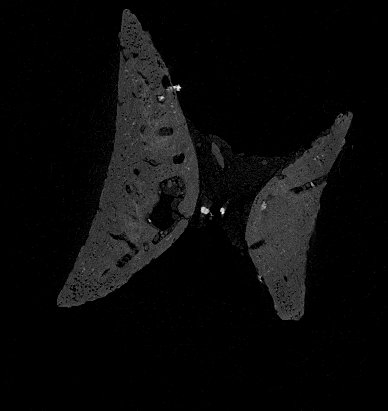

Supplement: S4 File — (ZIP) [file pone.0228610.s004.zip › 29_144/BrI_IR_rec0953.jpg]

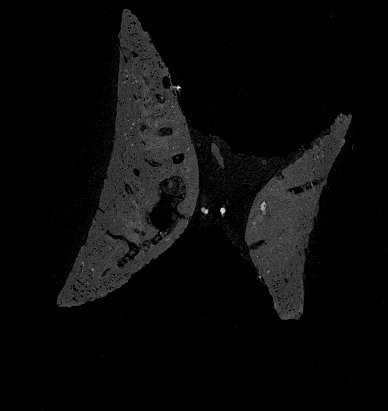

Supplement: S4 File — (ZIP) [file pone.0228610.s004.zip › 29_144/BrI_IR_rec0957.jpg]

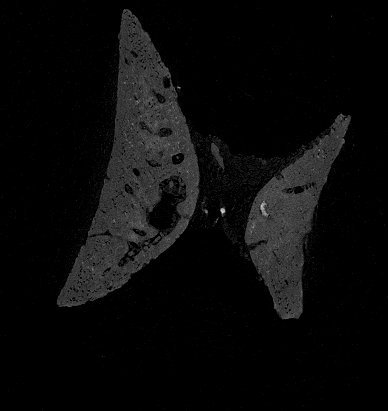

Supplement: S4 File — (ZIP) [file pone.0228610.s004.zip › 29_144/BrI_IR_rec0961.jpg]

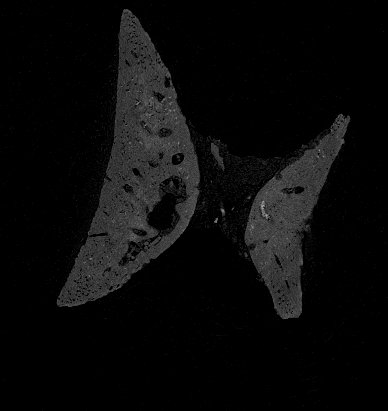

Supplement: S4 File — (ZIP) [file pone.0228610.s004.zip › 29_144/BrI_IR_rec0965.jpg]

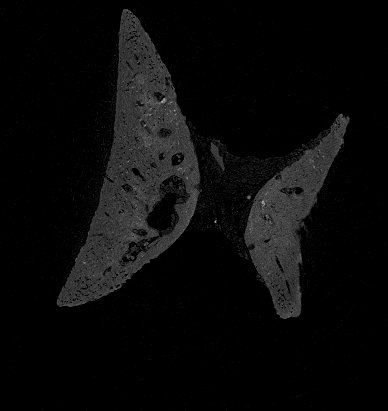

Supplement: S4 File — (ZIP) [file pone.0228610.s004.zip › 29_144/BrI_IR_rec0969.jpg]

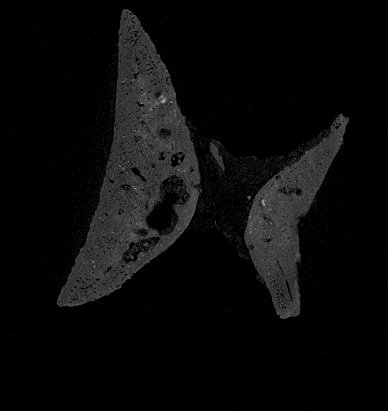

Supplement: S4 File — (ZIP) [file pone.0228610.s004.zip › 29_144/BrI_IR_rec0973.jpg]

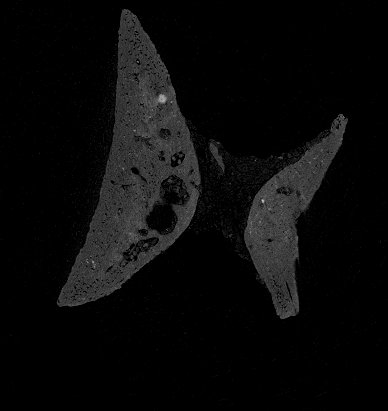

Supplement: S4 File — (ZIP) [file pone.0228610.s004.zip › 29_144/BrI_IR_rec0977.jpg]

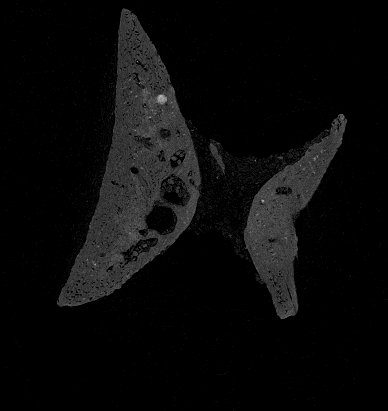

Supplement: S4 File — (ZIP) [file pone.0228610.s004.zip › 29_144/BrI_IR_rec0981.jpg]

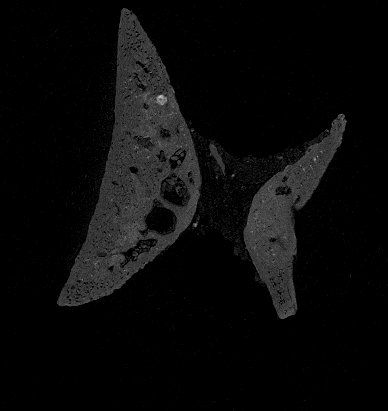

Supplement: S4 File — (ZIP) [file pone.0228610.s004.zip › 29_144/BrI_IR_rec0985.jpg]

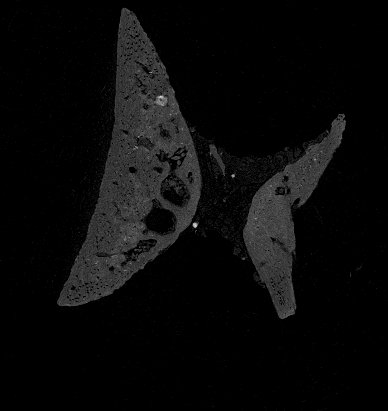

Supplement: S4 File — (ZIP) [file pone.0228610.s004.zip › 29_144/BrI_IR_rec0989.jpg]

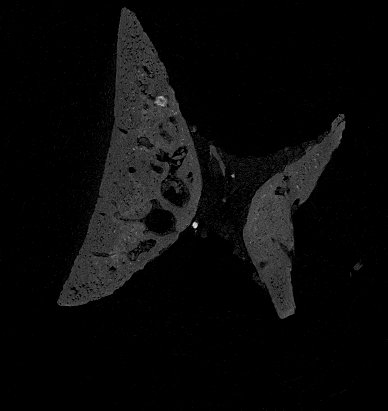

Supplement: S4 File — (ZIP) [file pone.0228610.s004.zip › 29_144/BrI_IR_rec0993.jpg]

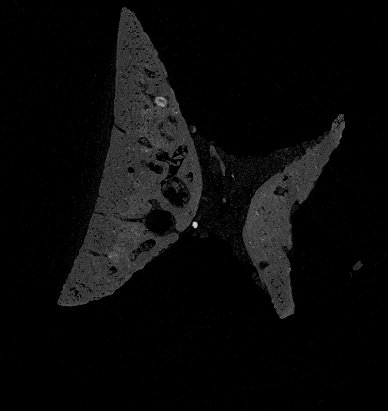

Supplement: S4 File — (ZIP) [file pone.0228610.s004.zip › 29_144/BrI_IR_rec0997.jpg]

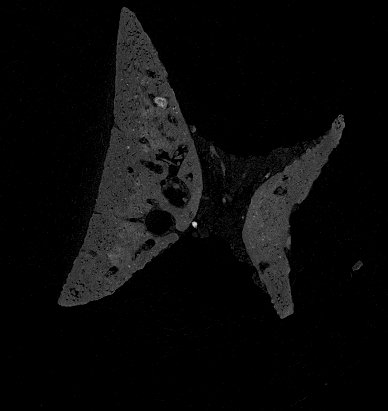

Supplement: S4 File — (ZIP) [file pone.0228610.s004.zip › 29_144/BrI_IR_rec1001.jpg]

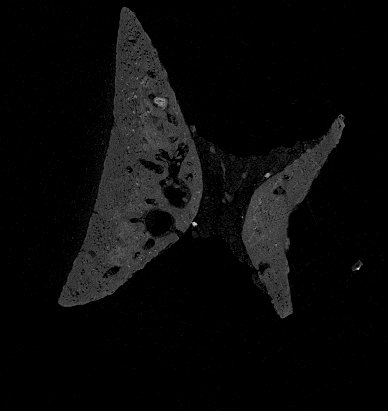

Supplement: S4 File — (ZIP) [file pone.0228610.s004.zip › 29_144/BrI_IR_rec1005.jpg]

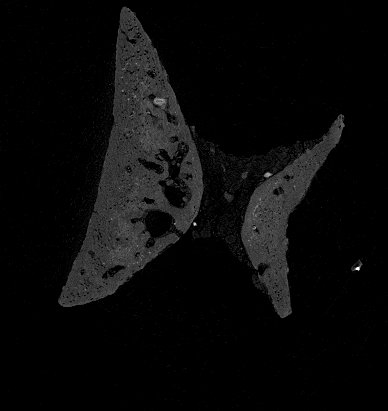

Supplement: S4 File — (ZIP) [file pone.0228610.s004.zip › 29_144/BrI_IR_rec1009.jpg]

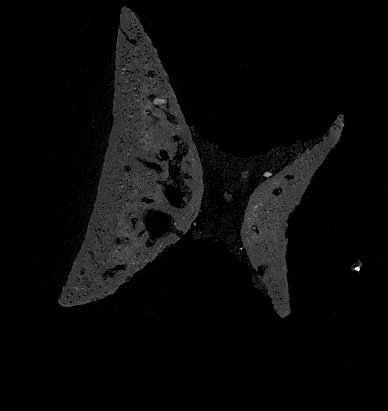

Supplement: S4 File — (ZIP) [file pone.0228610.s004.zip › 29_144/BrI_IR_rec1013.jpg]

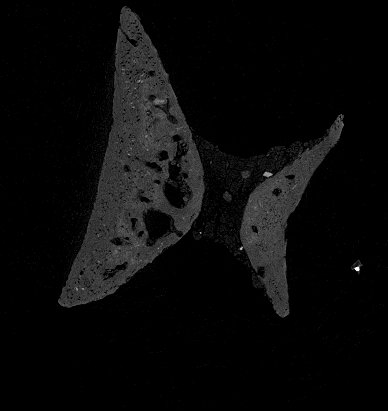

Supplement: S4 File — (ZIP) [file pone.0228610.s004.zip › 29_144/BrI_IR_rec1017.jpg]

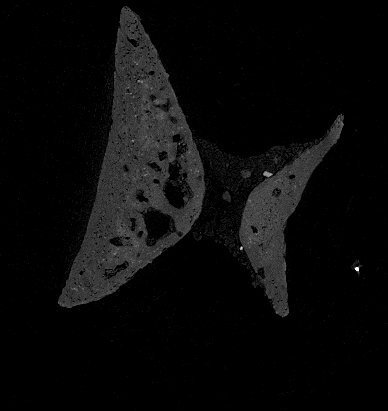

Supplement: S4 File — (ZIP) [file pone.0228610.s004.zip › 29_144/BrI_IR_rec1021.jpg]

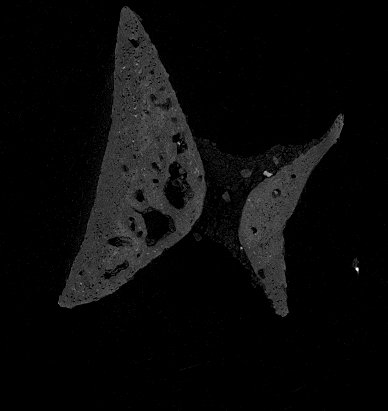

Supplement: S4 File — (ZIP) [file pone.0228610.s004.zip › 29_144/BrI_IR_rec1025.jpg]

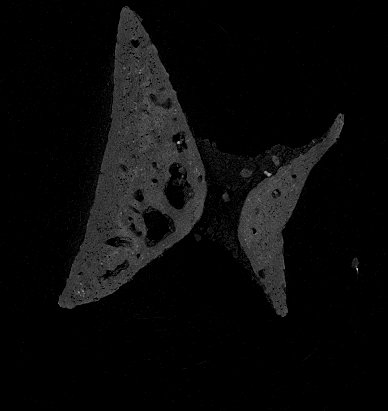

Supplement: S4 File — (ZIP) [file pone.0228610.s004.zip › 29_144/BrI_IR_rec1029.jpg]

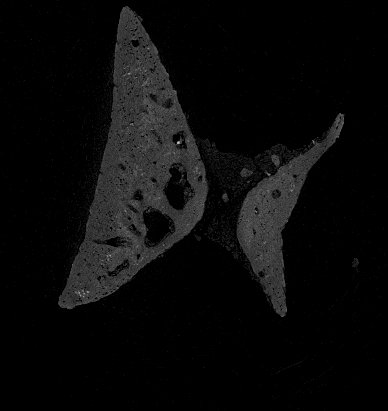

Supplement: S4 File — (ZIP) [file pone.0228610.s004.zip › 29_144/BrI_IR_rec1033.jpg]

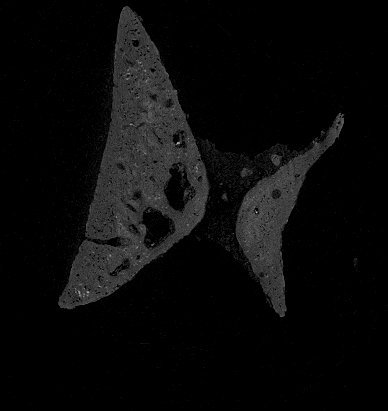

Supplement: S4 File — (ZIP) [file pone.0228610.s004.zip › 29_144/BrI_IR_rec1037.jpg]

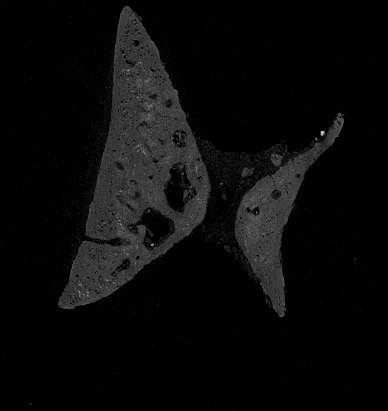

Supplement: S4 File — (ZIP) [file pone.0228610.s004.zip › 29_144/BrI_IR_rec1041.jpg]

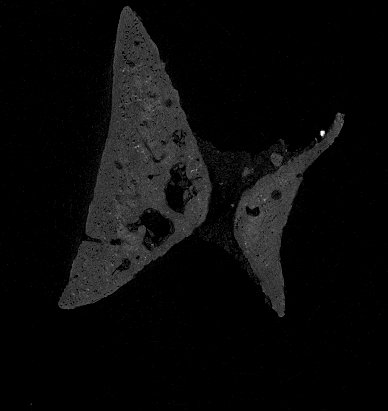

Supplement: S4 File — (ZIP) [file pone.0228610.s004.zip › 29_144/BrI_IR_rec1045.jpg]

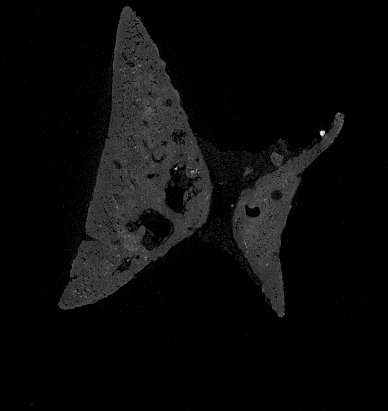

Supplement: S4 File — (ZIP) [file pone.0228610.s004.zip › 29_144/BrI_IR_rec1049.jpg]

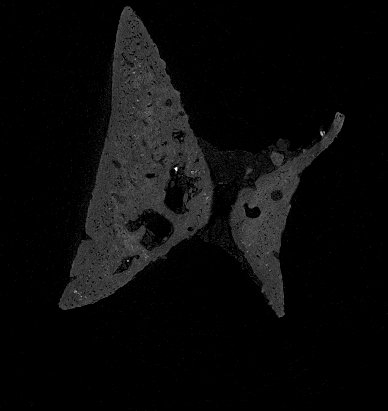

Supplement: S4 File — (ZIP) [file pone.0228610.s004.zip › 29_144/BrI_IR_rec1053.jpg]

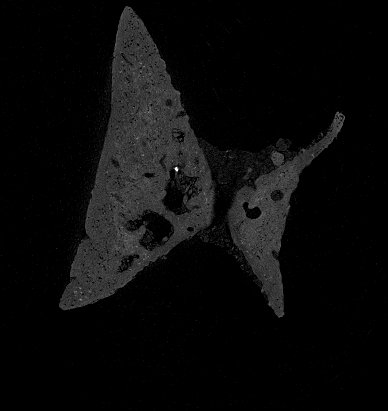

Supplement: S4 File — (ZIP) [file pone.0228610.s004.zip › 29_144/BrI_IR_rec1057.jpg]

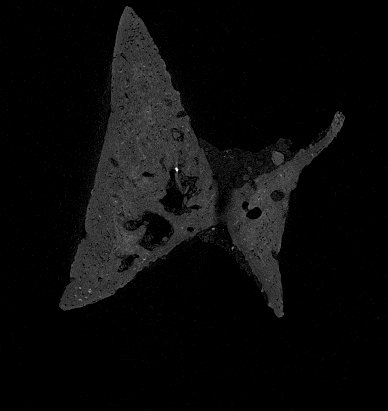

Supplement: S4 File — (ZIP) [file pone.0228610.s004.zip › 29_144/BrI_IR_rec1061.jpg]

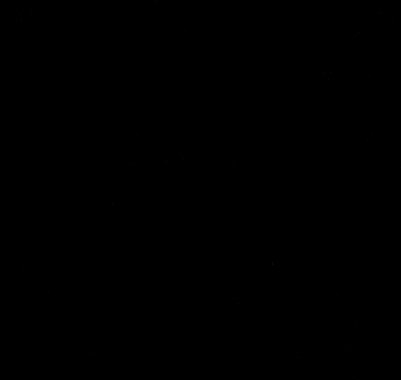

Supplement: S5 File — (ZIP) [file pone.0228610.s005.zip › 32_144/Br-2_IR_rec1334.jpg]

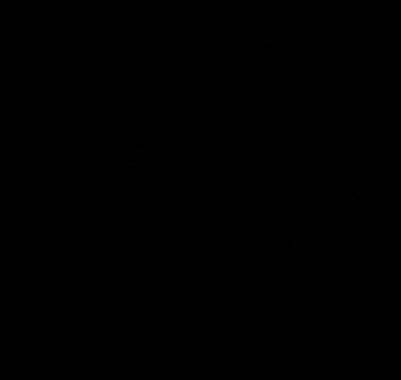

Supplement: S5 File — (ZIP) [file pone.0228610.s005.zip › 32_144/Br-2_IR_rec1338.jpg]

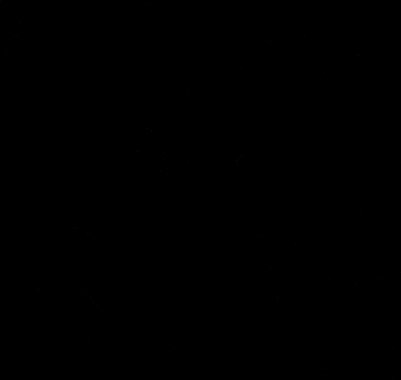

Supplement: S5 File — (ZIP) [file pone.0228610.s005.zip › 32_144/Br-2_IR_rec1342.jpg]

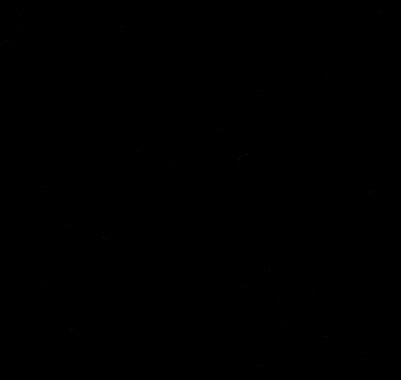

Supplement: S5 File — (ZIP) [file pone.0228610.s005.zip › 32_144/Br-2_IR_rec1346.jpg]

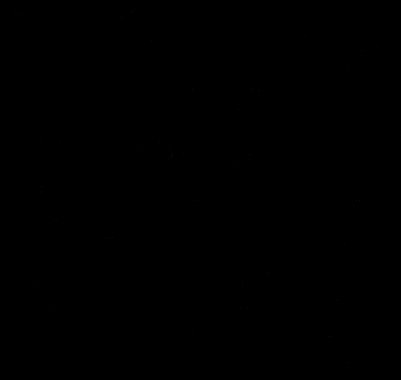

Supplement: S5 File — (ZIP) [file pone.0228610.s005.zip › 32_144/Br-2_IR_rec1350.jpg]

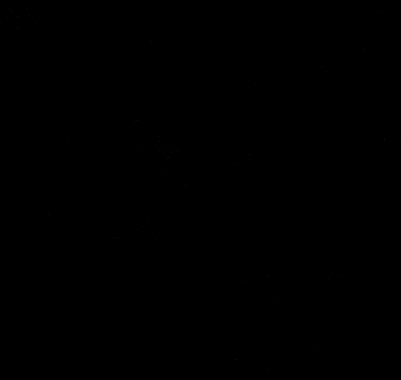

Supplement: S5 File — (ZIP) [file pone.0228610.s005.zip › 32_144/Br-2_IR_rec1354.jpg]

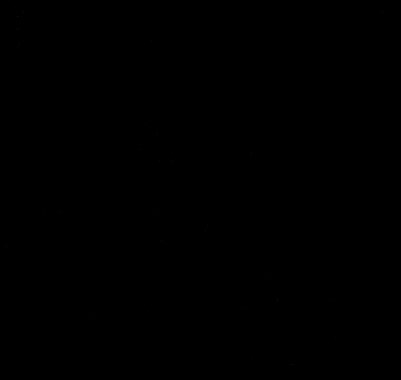

Supplement: S5 File — (ZIP) [file pone.0228610.s005.zip › 32_144/Br-2_IR_rec1358.jpg]

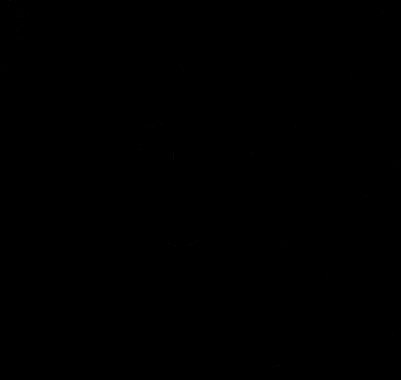

Supplement: S5 File — (ZIP) [file pone.0228610.s005.zip › 32_144/Br-2_IR_rec1362.jpg]

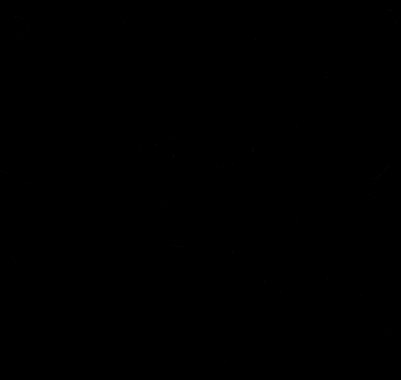

Supplement: S5 File — (ZIP) [file pone.0228610.s005.zip › 32_144/Br-2_IR_rec1366.jpg]

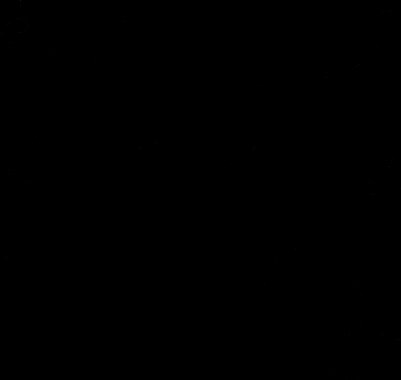

Supplement: S5 File — (ZIP) [file pone.0228610.s005.zip › 32_144/Br-2_IR_rec1370.jpg]

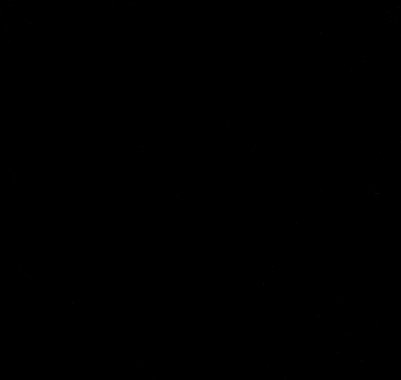

Supplement: S5 File — (ZIP) [file pone.0228610.s005.zip › 32_144/Br-2_IR_rec1374.jpg]

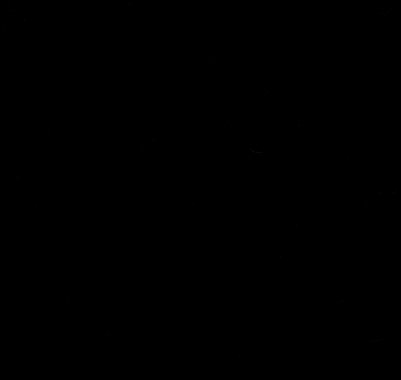

Supplement: S5 File — (ZIP) [file pone.0228610.s005.zip › 32_144/Br-2_IR_rec1378.jpg]

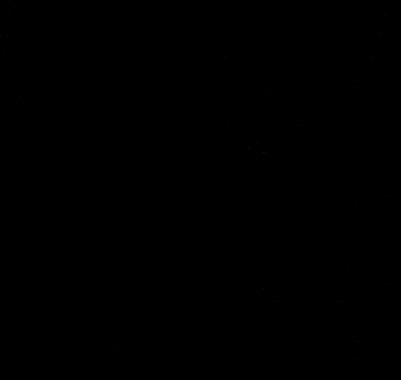

Supplement: S5 File — (ZIP) [file pone.0228610.s005.zip › 32_144/Br-2_IR_rec1382.jpg]

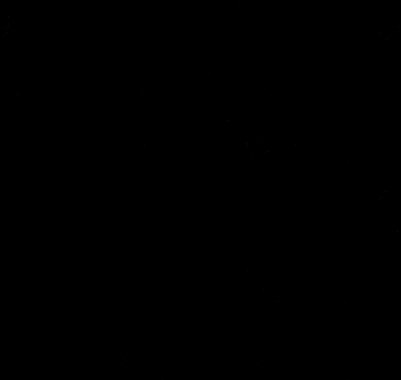

Supplement: S5 File — (ZIP) [file pone.0228610.s005.zip › 32_144/Br-2_IR_rec1386.jpg]

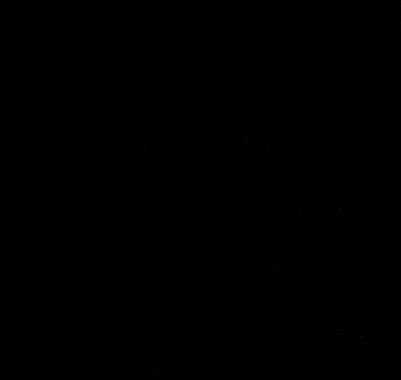

Supplement: S5 File — (ZIP) [file pone.0228610.s005.zip › 32_144/Br-2_IR_rec1390.jpg]

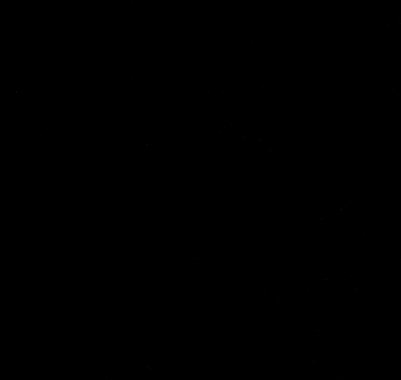

Supplement: S5 File — (ZIP) [file pone.0228610.s005.zip › 32_144/Br-2_IR_rec1394.jpg]

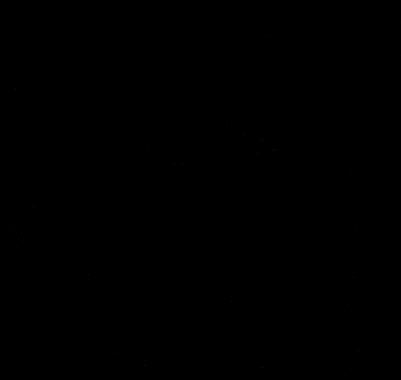

Supplement: S5 File — (ZIP) [file pone.0228610.s005.zip › 32_144/Br-2_IR_rec1398.jpg]

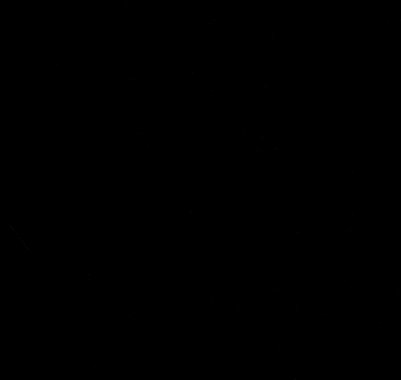

Supplement: S5 File — (ZIP) [file pone.0228610.s005.zip › 32_144/Br-2_IR_rec1402.jpg]

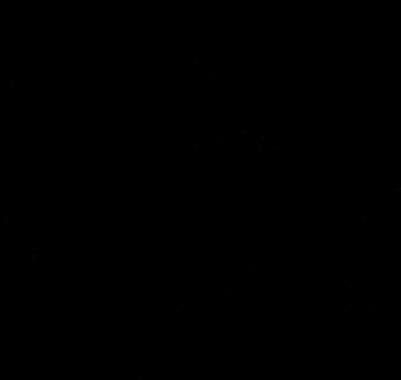

Supplement: S5 File — (ZIP) [file pone.0228610.s005.zip › 32_144/Br-2_IR_rec1406.jpg]

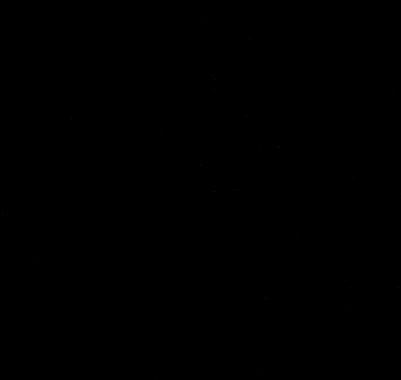

Supplement: S5 File — (ZIP) [file pone.0228610.s005.zip › 32_144/Br-2_IR_rec1410.jpg]

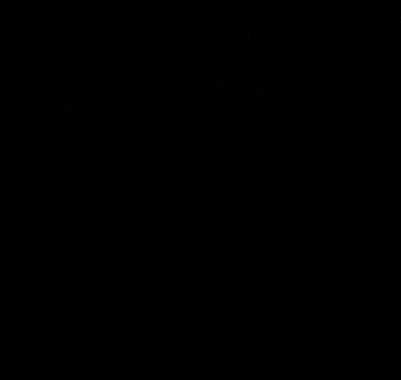

Supplement: S5 File — (ZIP) [file pone.0228610.s005.zip › 32_144/Br-2_IR_rec1414.jpg]
