# Supplementary figures and images for: A new small-sized stem salamander from the Middle Jurassic of Western Siberia, Russia (part 7 of 10)
Source: PLoS One. 2020 Feb 19;15(2):e0228610. doi: 10.1371/journal.pone.0228610 (PMC7029856; doi:10.1371/journal.pone.0228610)

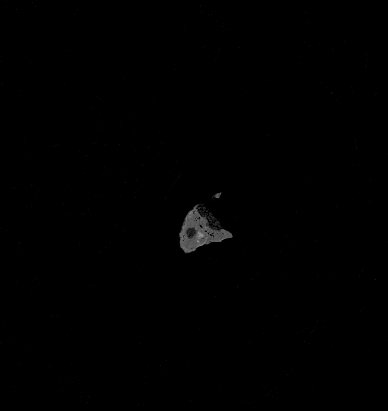

Supplement: S4 File — (ZIP) [file pone.0228610.s004.zip › 29_144/BrI_IR_rec0349.jpg]

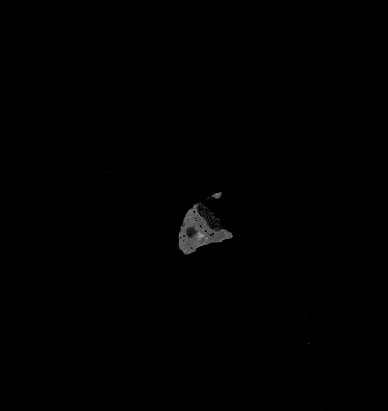

Supplement: S4 File — (ZIP) [file pone.0228610.s004.zip › 29_144/BrI_IR_rec0353.jpg]

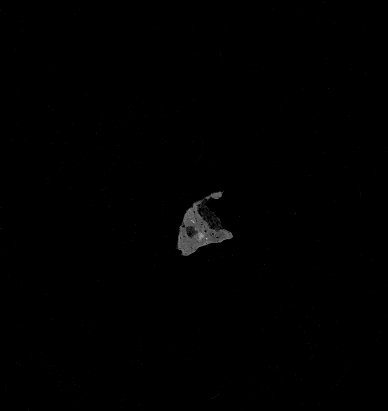

Supplement: S4 File — (ZIP) [file pone.0228610.s004.zip › 29_144/BrI_IR_rec0357.jpg]

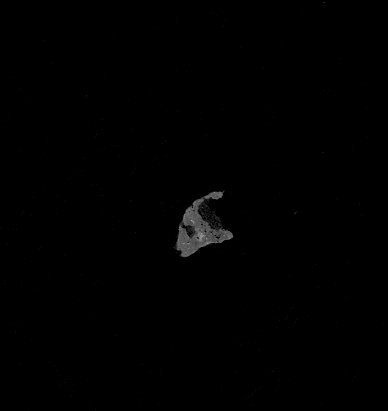

Supplement: S4 File — (ZIP) [file pone.0228610.s004.zip › 29_144/BrI_IR_rec0361.jpg]

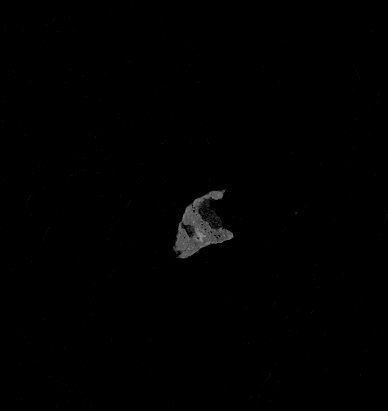

Supplement: S4 File — (ZIP) [file pone.0228610.s004.zip › 29_144/BrI_IR_rec0365.jpg]

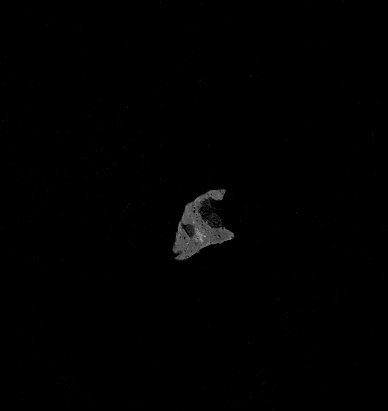

Supplement: S4 File — (ZIP) [file pone.0228610.s004.zip › 29_144/BrI_IR_rec0369.jpg]

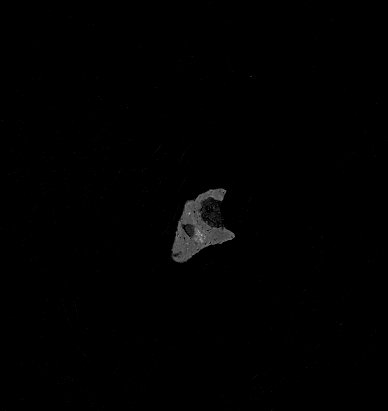

Supplement: S4 File — (ZIP) [file pone.0228610.s004.zip › 29_144/BrI_IR_rec0373.jpg]

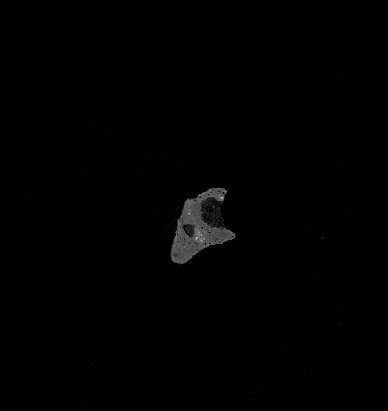

Supplement: S4 File — (ZIP) [file pone.0228610.s004.zip › 29_144/BrI_IR_rec0377.jpg]

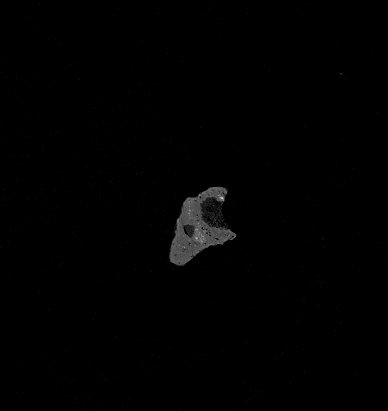

Supplement: S4 File — (ZIP) [file pone.0228610.s004.zip › 29_144/BrI_IR_rec0381.jpg]

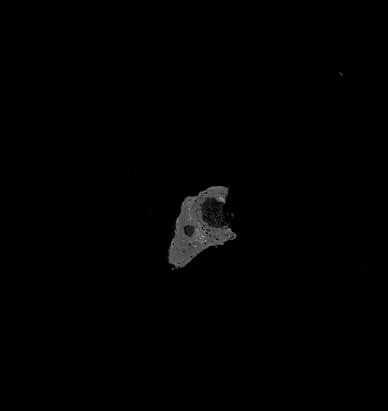

Supplement: S4 File — (ZIP) [file pone.0228610.s004.zip › 29_144/BrI_IR_rec0385.jpg]

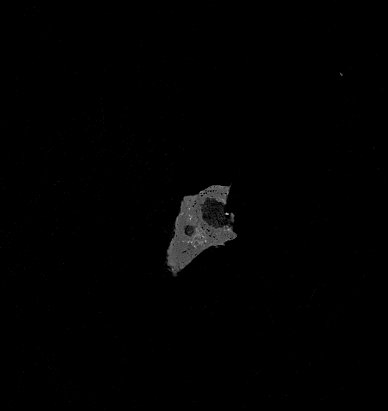

Supplement: S4 File — (ZIP) [file pone.0228610.s004.zip › 29_144/BrI_IR_rec0389.jpg]

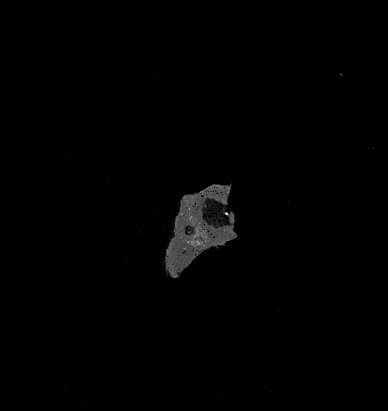

Supplement: S4 File — (ZIP) [file pone.0228610.s004.zip › 29_144/BrI_IR_rec0393.jpg]

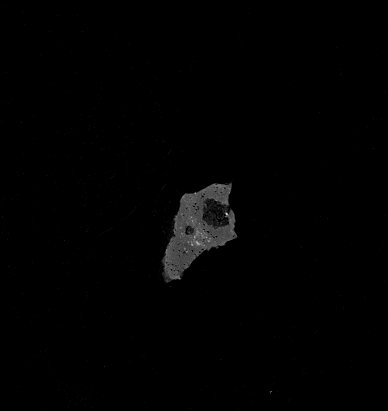

Supplement: S4 File — (ZIP) [file pone.0228610.s004.zip › 29_144/BrI_IR_rec0397.jpg]

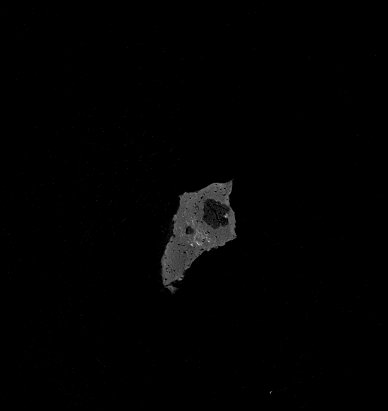

Supplement: S4 File — (ZIP) [file pone.0228610.s004.zip › 29_144/BrI_IR_rec0401.jpg]

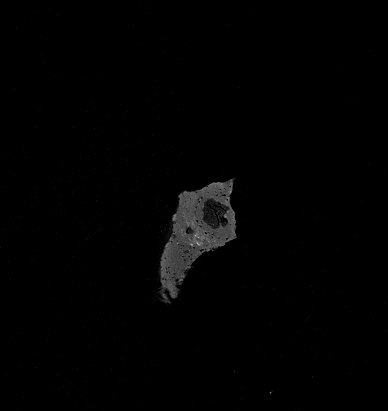

Supplement: S4 File — (ZIP) [file pone.0228610.s004.zip › 29_144/BrI_IR_rec0405.jpg]

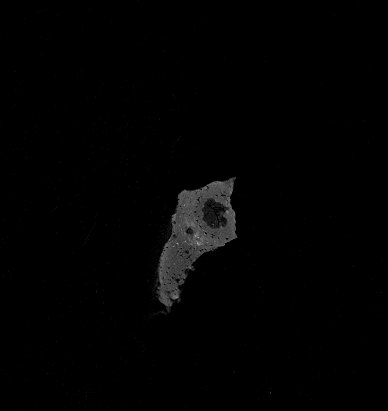

Supplement: S4 File — (ZIP) [file pone.0228610.s004.zip › 29_144/BrI_IR_rec0409.jpg]

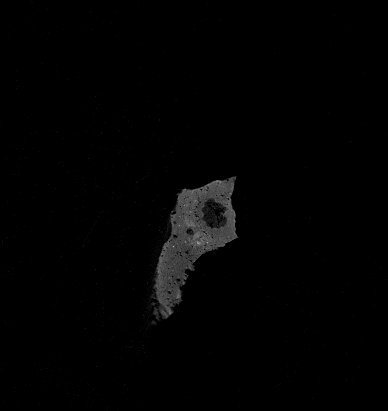

Supplement: S4 File — (ZIP) [file pone.0228610.s004.zip › 29_144/BrI_IR_rec0413.jpg]

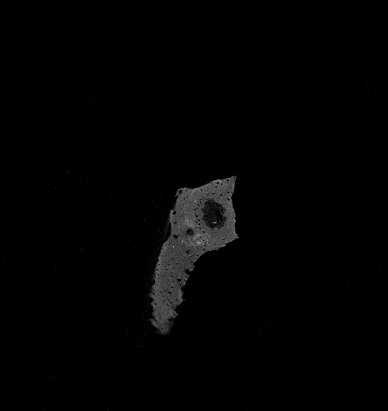

Supplement: S4 File — (ZIP) [file pone.0228610.s004.zip › 29_144/BrI_IR_rec0417.jpg]

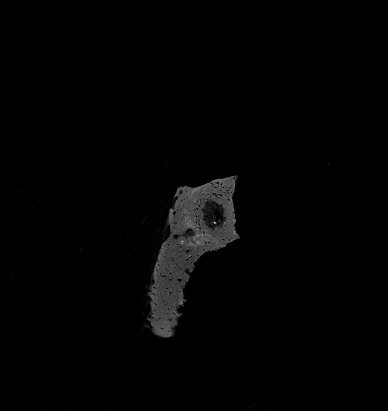

Supplement: S4 File — (ZIP) [file pone.0228610.s004.zip › 29_144/BrI_IR_rec0421.jpg]

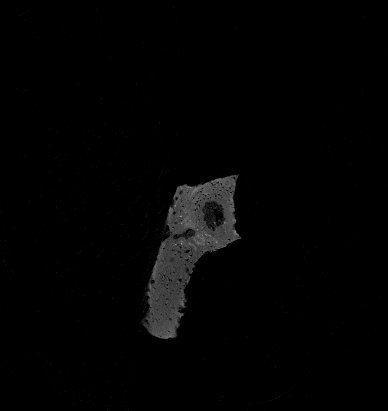

Supplement: S4 File — (ZIP) [file pone.0228610.s004.zip › 29_144/BrI_IR_rec0425.jpg]

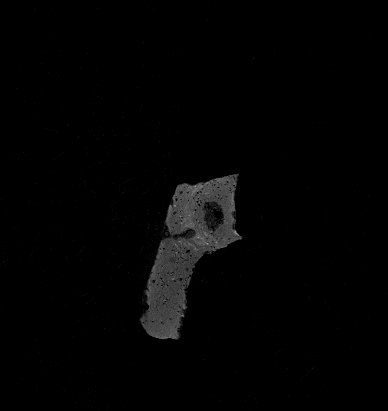

Supplement: S4 File — (ZIP) [file pone.0228610.s004.zip › 29_144/BrI_IR_rec0429.jpg]

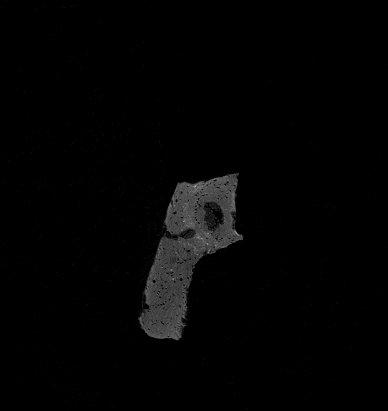

Supplement: S4 File — (ZIP) [file pone.0228610.s004.zip › 29_144/BrI_IR_rec0433.jpg]

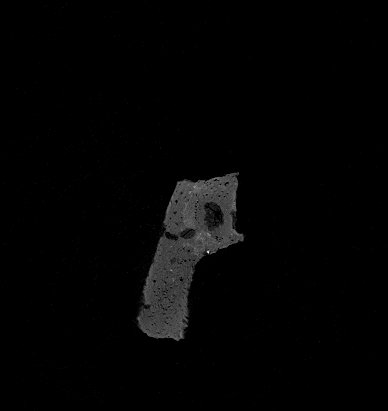

Supplement: S4 File — (ZIP) [file pone.0228610.s004.zip › 29_144/BrI_IR_rec0437.jpg]

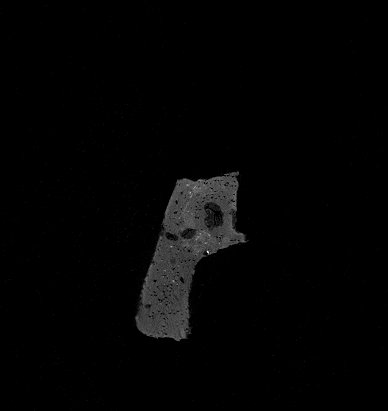

Supplement: S4 File — (ZIP) [file pone.0228610.s004.zip › 29_144/BrI_IR_rec0441.jpg]

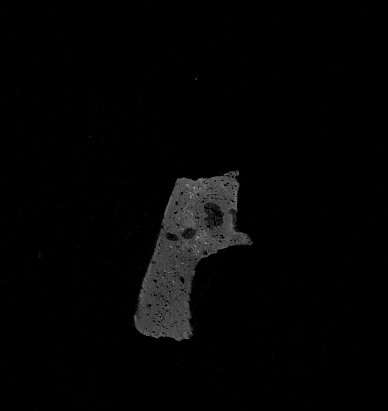

Supplement: S4 File — (ZIP) [file pone.0228610.s004.zip › 29_144/BrI_IR_rec0445.jpg]

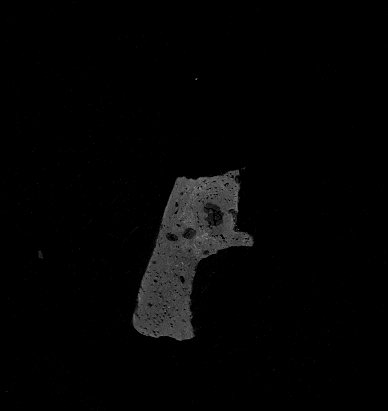

Supplement: S4 File — (ZIP) [file pone.0228610.s004.zip › 29_144/BrI_IR_rec0449.jpg]

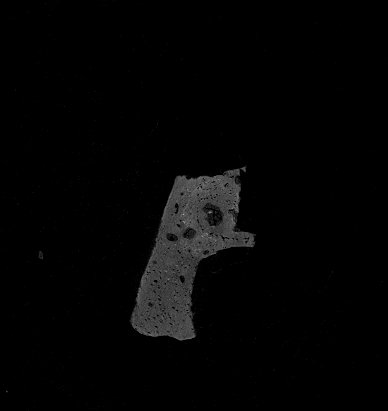

Supplement: S4 File — (ZIP) [file pone.0228610.s004.zip › 29_144/BrI_IR_rec0453.jpg]

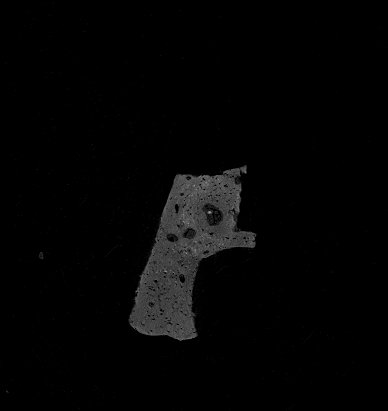

Supplement: S4 File — (ZIP) [file pone.0228610.s004.zip › 29_144/BrI_IR_rec0457.jpg]

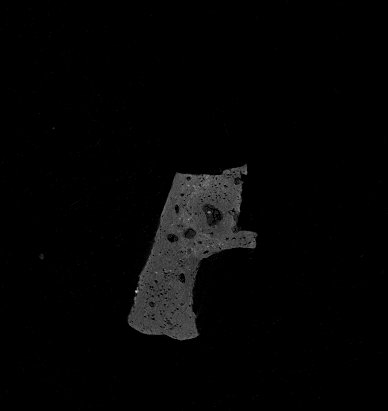

Supplement: S4 File — (ZIP) [file pone.0228610.s004.zip › 29_144/BrI_IR_rec0461.jpg]

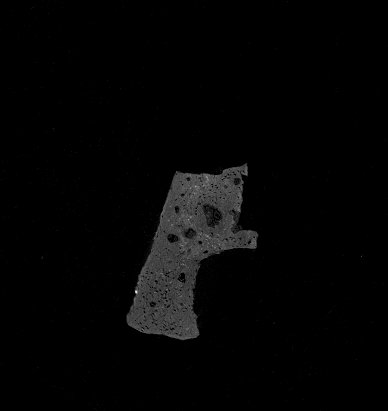

Supplement: S4 File — (ZIP) [file pone.0228610.s004.zip › 29_144/BrI_IR_rec0465.jpg]

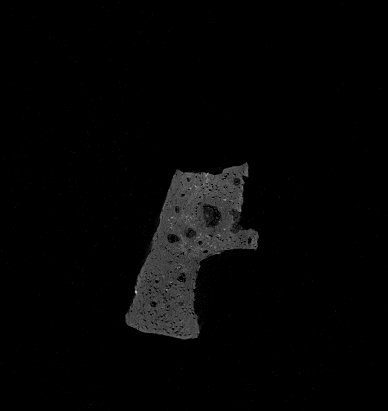

Supplement: S4 File — (ZIP) [file pone.0228610.s004.zip › 29_144/BrI_IR_rec0469.jpg]

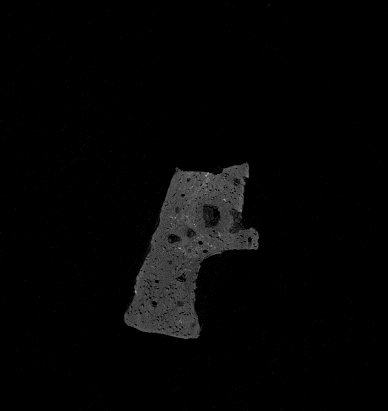

Supplement: S4 File — (ZIP) [file pone.0228610.s004.zip › 29_144/BrI_IR_rec0473.jpg]

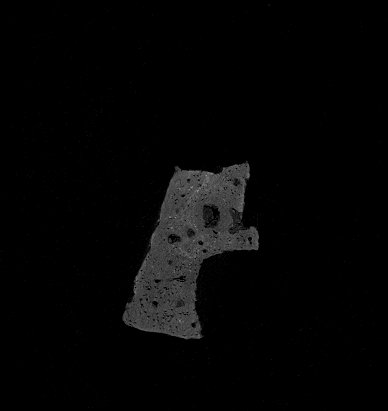

Supplement: S4 File — (ZIP) [file pone.0228610.s004.zip › 29_144/BrI_IR_rec0477.jpg]

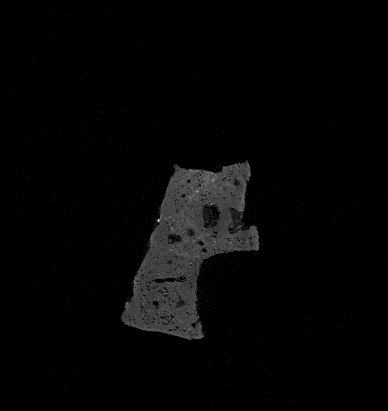

Supplement: S4 File — (ZIP) [file pone.0228610.s004.zip › 29_144/BrI_IR_rec0481.jpg]

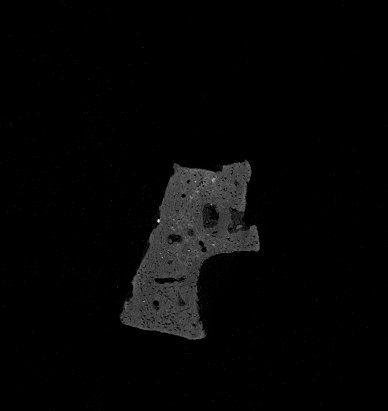

Supplement: S4 File — (ZIP) [file pone.0228610.s004.zip › 29_144/BrI_IR_rec0485.jpg]

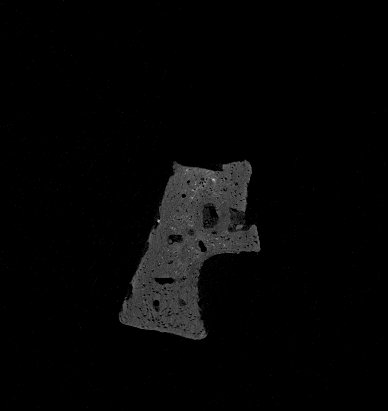

Supplement: S4 File — (ZIP) [file pone.0228610.s004.zip › 29_144/BrI_IR_rec0489.jpg]

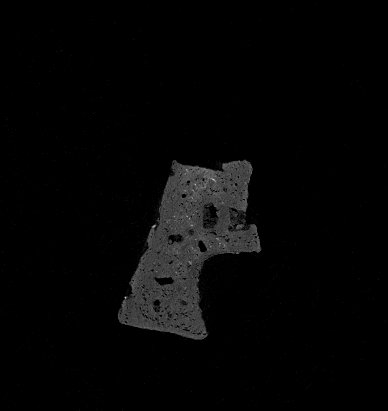

Supplement: S4 File — (ZIP) [file pone.0228610.s004.zip › 29_144/BrI_IR_rec0493.jpg]

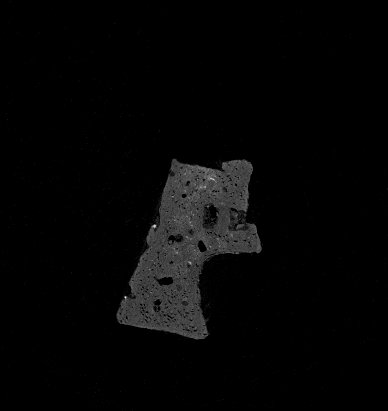

Supplement: S4 File — (ZIP) [file pone.0228610.s004.zip › 29_144/BrI_IR_rec0497.jpg]

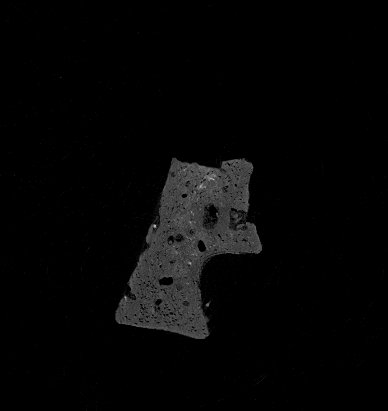

Supplement: S4 File — (ZIP) [file pone.0228610.s004.zip › 29_144/BrI_IR_rec0501.jpg]

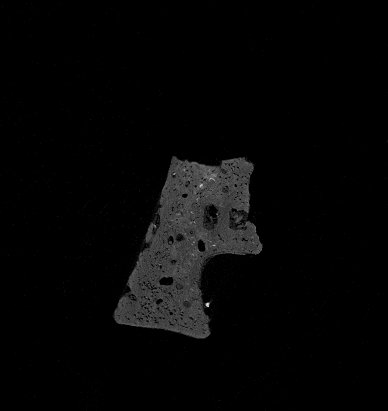

Supplement: S4 File — (ZIP) [file pone.0228610.s004.zip › 29_144/BrI_IR_rec0505.jpg]

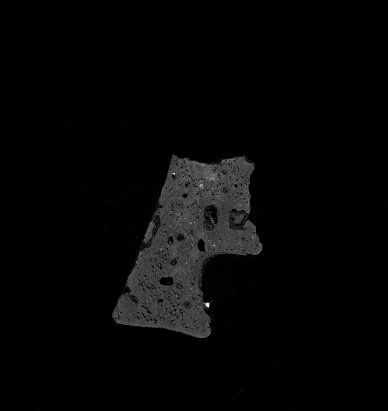

Supplement: S4 File — (ZIP) [file pone.0228610.s004.zip › 29_144/BrI_IR_rec0509.jpg]

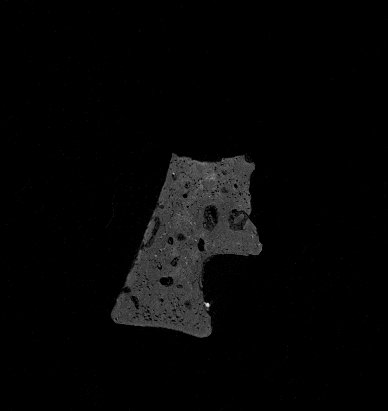

Supplement: S4 File — (ZIP) [file pone.0228610.s004.zip › 29_144/BrI_IR_rec0513.jpg]

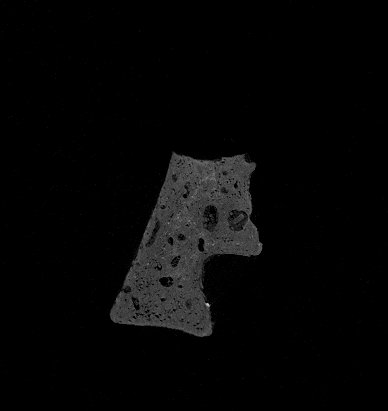

Supplement: S4 File — (ZIP) [file pone.0228610.s004.zip › 29_144/BrI_IR_rec0517.jpg]

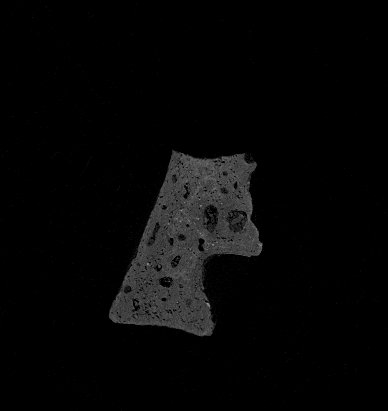

Supplement: S4 File — (ZIP) [file pone.0228610.s004.zip › 29_144/BrI_IR_rec0521.jpg]

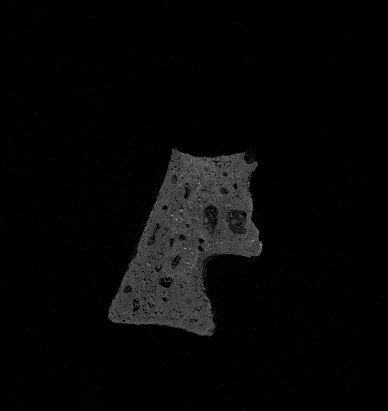

Supplement: S4 File — (ZIP) [file pone.0228610.s004.zip › 29_144/BrI_IR_rec0525.jpg]

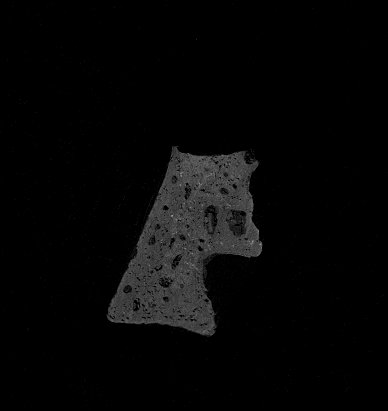

Supplement: S4 File — (ZIP) [file pone.0228610.s004.zip › 29_144/BrI_IR_rec0529.jpg]

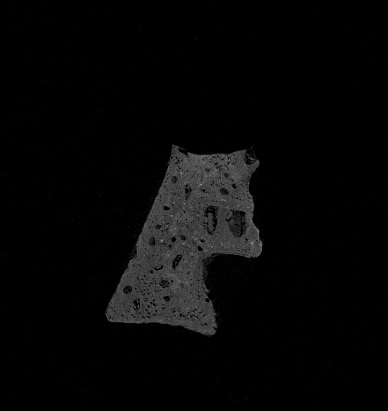

Supplement: S4 File — (ZIP) [file pone.0228610.s004.zip › 29_144/BrI_IR_rec0533.jpg]

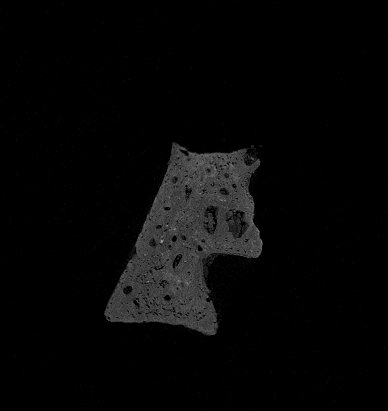

Supplement: S4 File — (ZIP) [file pone.0228610.s004.zip › 29_144/BrI_IR_rec0537.jpg]

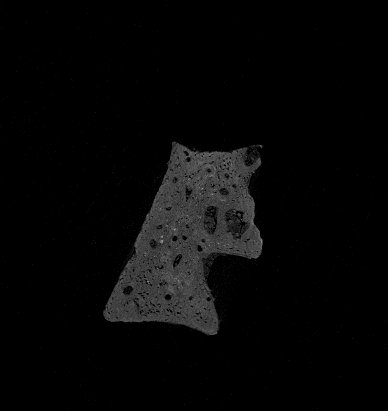

Supplement: S4 File — (ZIP) [file pone.0228610.s004.zip › 29_144/BrI_IR_rec0541.jpg]

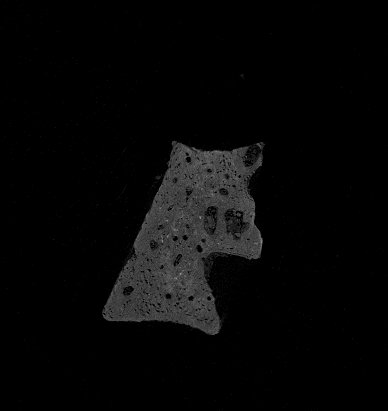

Supplement: S4 File — (ZIP) [file pone.0228610.s004.zip › 29_144/BrI_IR_rec0545.jpg]

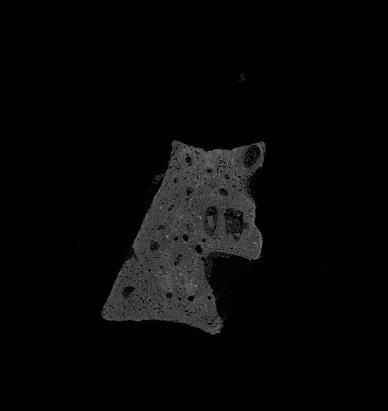

Supplement: S4 File — (ZIP) [file pone.0228610.s004.zip › 29_144/BrI_IR_rec0549.jpg]

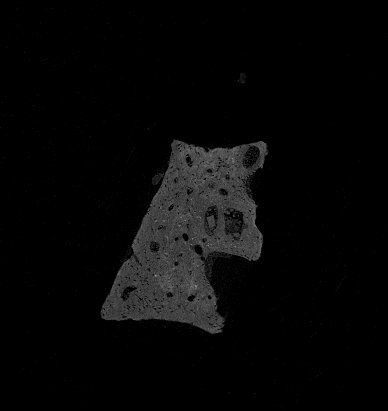

Supplement: S4 File — (ZIP) [file pone.0228610.s004.zip › 29_144/BrI_IR_rec0553.jpg]

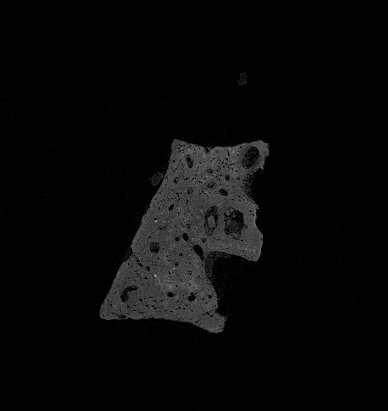

Supplement: S4 File — (ZIP) [file pone.0228610.s004.zip › 29_144/BrI_IR_rec0557.jpg]

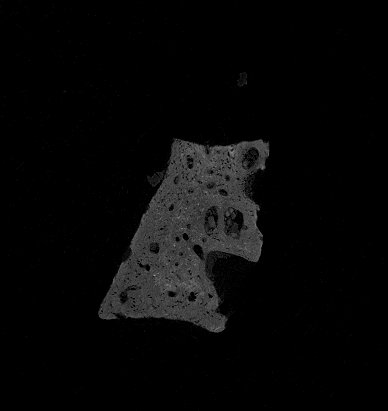

Supplement: S4 File — (ZIP) [file pone.0228610.s004.zip › 29_144/BrI_IR_rec0561.jpg]

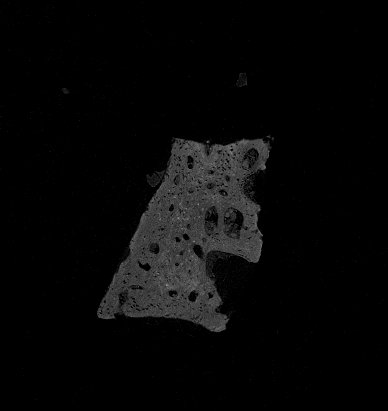

Supplement: S4 File — (ZIP) [file pone.0228610.s004.zip › 29_144/BrI_IR_rec0565.jpg]

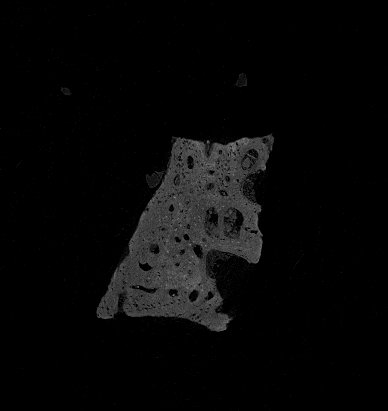

Supplement: S4 File — (ZIP) [file pone.0228610.s004.zip › 29_144/BrI_IR_rec0569.jpg]

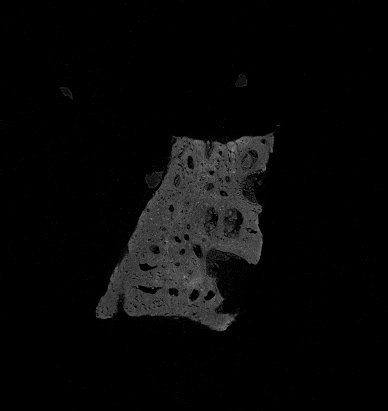

Supplement: S4 File — (ZIP) [file pone.0228610.s004.zip › 29_144/BrI_IR_rec0573.jpg]

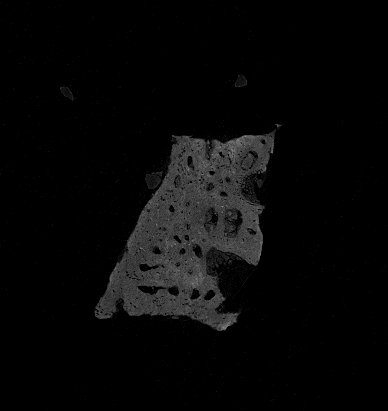

Supplement: S4 File — (ZIP) [file pone.0228610.s004.zip › 29_144/BrI_IR_rec0577.jpg]

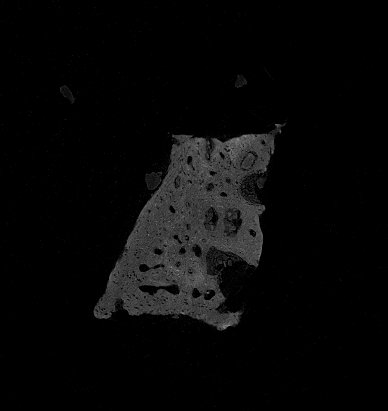

Supplement: S4 File — (ZIP) [file pone.0228610.s004.zip › 29_144/BrI_IR_rec0581.jpg]

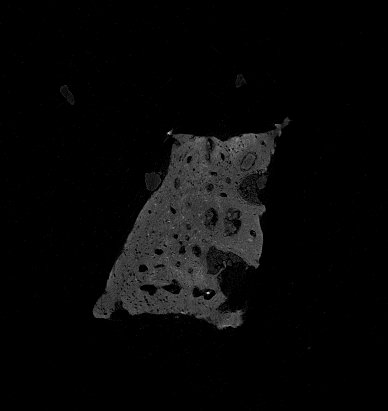

Supplement: S4 File — (ZIP) [file pone.0228610.s004.zip › 29_144/BrI_IR_rec0585.jpg]

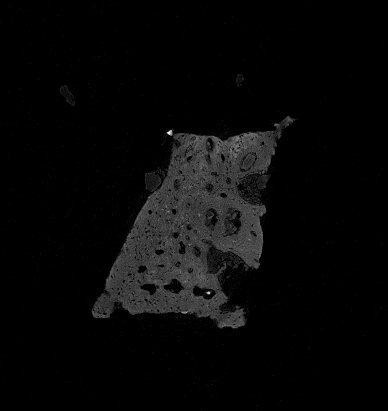

Supplement: S4 File — (ZIP) [file pone.0228610.s004.zip › 29_144/BrI_IR_rec0589.jpg]

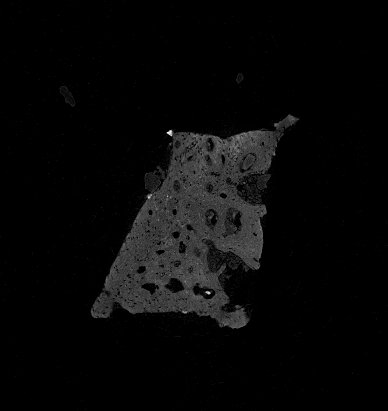

Supplement: S4 File — (ZIP) [file pone.0228610.s004.zip › 29_144/BrI_IR_rec0593.jpg]

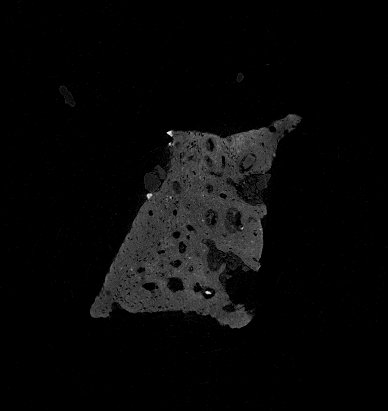

Supplement: S4 File — (ZIP) [file pone.0228610.s004.zip › 29_144/BrI_IR_rec0597.jpg]

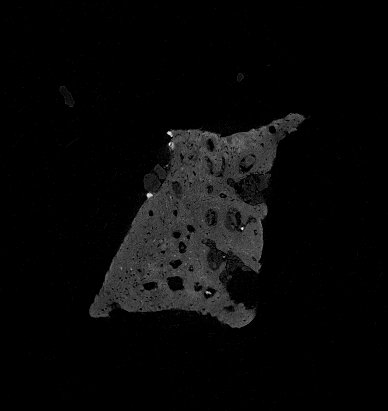

Supplement: S4 File — (ZIP) [file pone.0228610.s004.zip › 29_144/BrI_IR_rec0601.jpg]

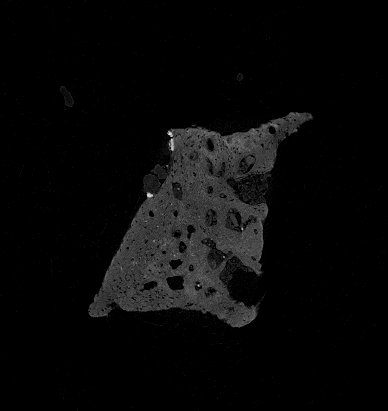

Supplement: S4 File — (ZIP) [file pone.0228610.s004.zip › 29_144/BrI_IR_rec0605.jpg]

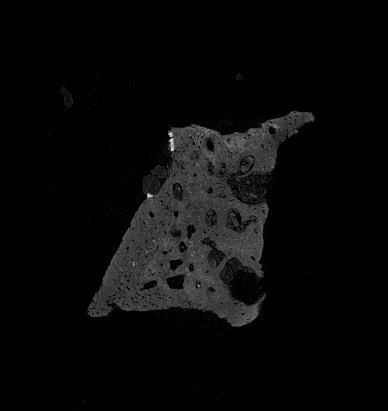

Supplement: S4 File — (ZIP) [file pone.0228610.s004.zip › 29_144/BrI_IR_rec0609.jpg]

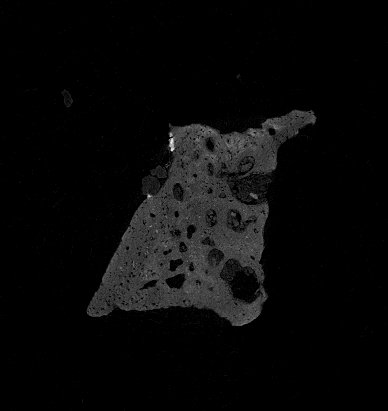

Supplement: S4 File — (ZIP) [file pone.0228610.s004.zip › 29_144/BrI_IR_rec0613.jpg]

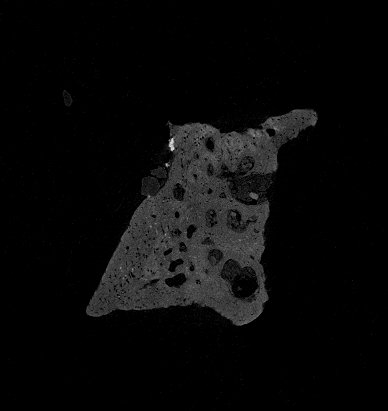

Supplement: S4 File — (ZIP) [file pone.0228610.s004.zip › 29_144/BrI_IR_rec0617.jpg]

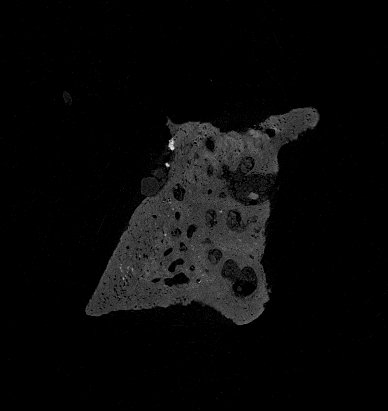

Supplement: S4 File — (ZIP) [file pone.0228610.s004.zip › 29_144/BrI_IR_rec0621.jpg]

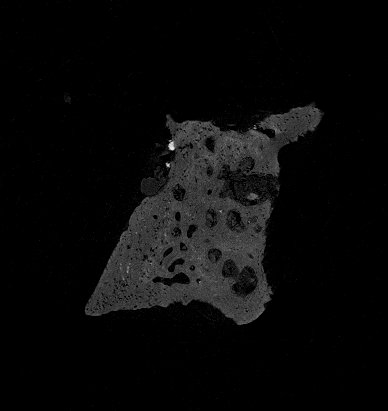

Supplement: S4 File — (ZIP) [file pone.0228610.s004.zip › 29_144/BrI_IR_rec0625.jpg]

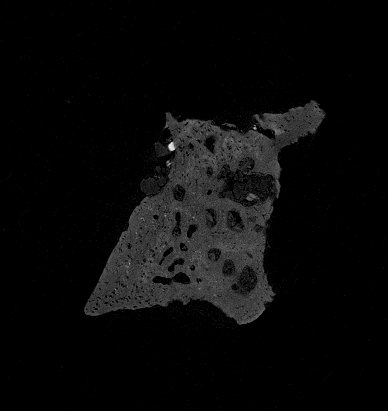

Supplement: S4 File — (ZIP) [file pone.0228610.s004.zip › 29_144/BrI_IR_rec0629.jpg]

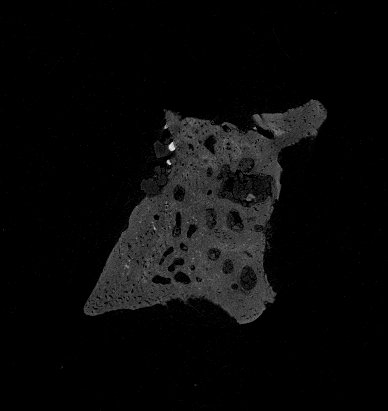

Supplement: S4 File — (ZIP) [file pone.0228610.s004.zip › 29_144/BrI_IR_rec0633.jpg]

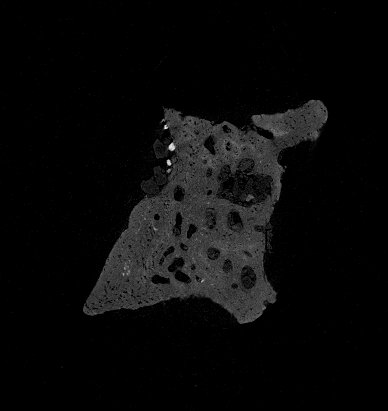

Supplement: S4 File — (ZIP) [file pone.0228610.s004.zip › 29_144/BrI_IR_rec0637.jpg]

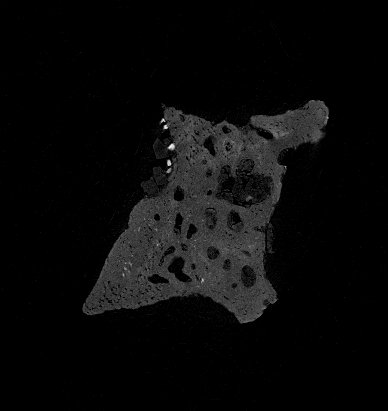

Supplement: S4 File — (ZIP) [file pone.0228610.s004.zip › 29_144/BrI_IR_rec0641.jpg]

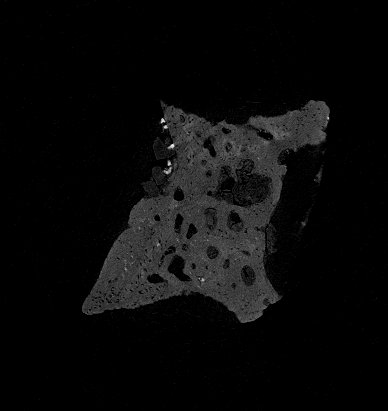

Supplement: S4 File — (ZIP) [file pone.0228610.s004.zip › 29_144/BrI_IR_rec0645.jpg]

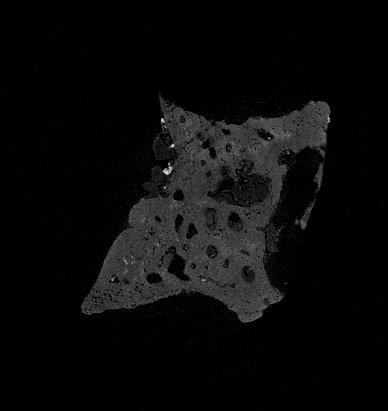

Supplement: S4 File — (ZIP) [file pone.0228610.s004.zip › 29_144/BrI_IR_rec0649.jpg]

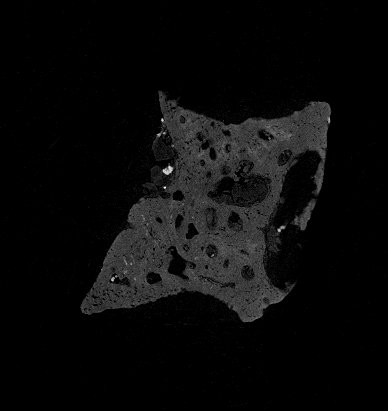

Supplement: S4 File — (ZIP) [file pone.0228610.s004.zip › 29_144/BrI_IR_rec0653.jpg]

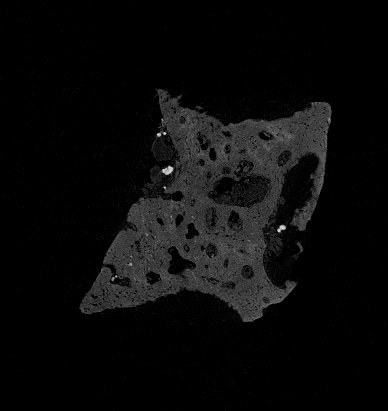

Supplement: S4 File — (ZIP) [file pone.0228610.s004.zip › 29_144/BrI_IR_rec0657.jpg]

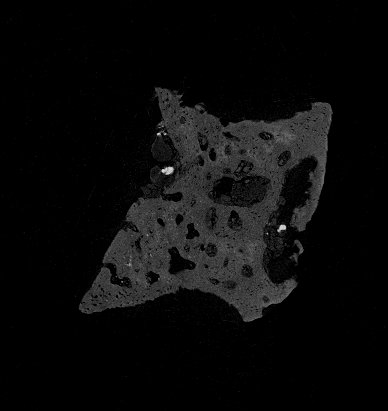

Supplement: S4 File — (ZIP) [file pone.0228610.s004.zip › 29_144/BrI_IR_rec0661.jpg]

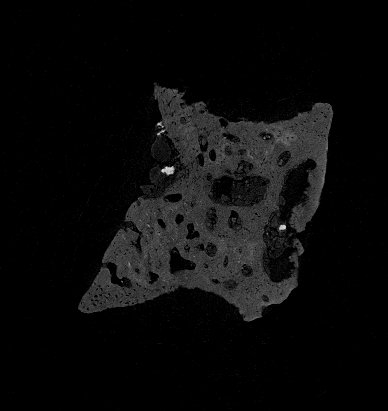

Supplement: S4 File — (ZIP) [file pone.0228610.s004.zip › 29_144/BrI_IR_rec0665.jpg]

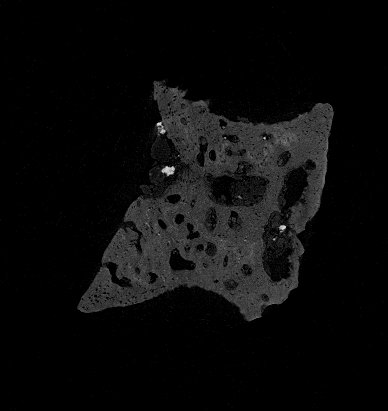

Supplement: S4 File — (ZIP) [file pone.0228610.s004.zip › 29_144/BrI_IR_rec0669.jpg]

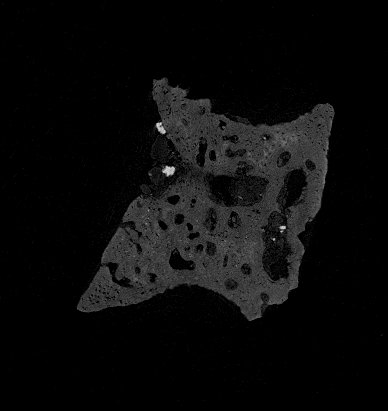

Supplement: S4 File — (ZIP) [file pone.0228610.s004.zip › 29_144/BrI_IR_rec0673.jpg]

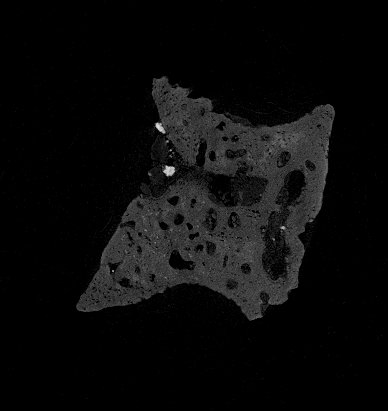

Supplement: S4 File — (ZIP) [file pone.0228610.s004.zip › 29_144/BrI_IR_rec0677.jpg]

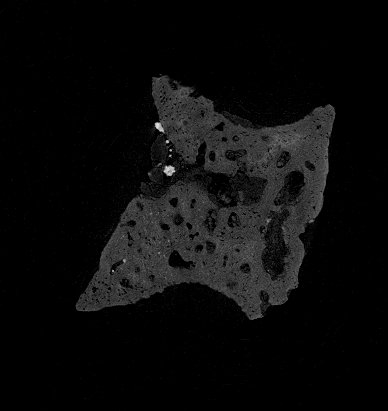

Supplement: S4 File — (ZIP) [file pone.0228610.s004.zip › 29_144/BrI_IR_rec0681.jpg]

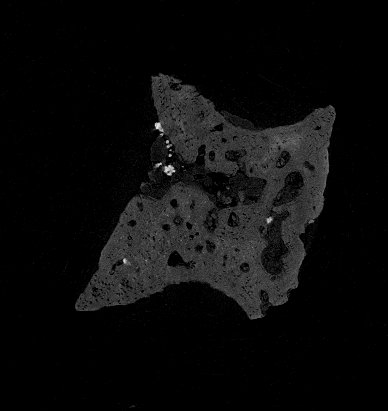

Supplement: S4 File — (ZIP) [file pone.0228610.s004.zip › 29_144/BrI_IR_rec0685.jpg]

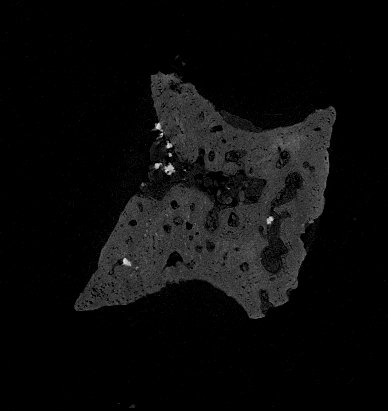

Supplement: S4 File — (ZIP) [file pone.0228610.s004.zip › 29_144/BrI_IR_rec0689.jpg]

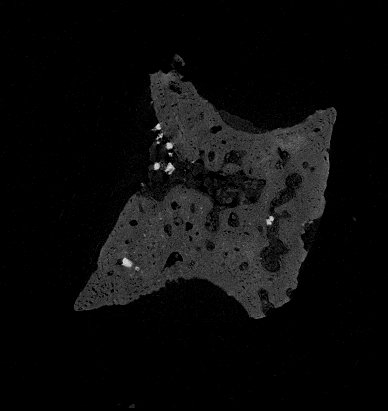

Supplement: S4 File — (ZIP) [file pone.0228610.s004.zip › 29_144/BrI_IR_rec0693.jpg]

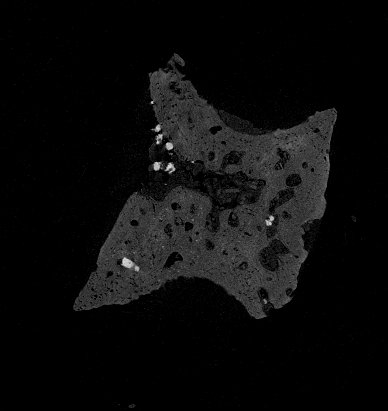

Supplement: S4 File — (ZIP) [file pone.0228610.s004.zip › 29_144/BrI_IR_rec0697.jpg]

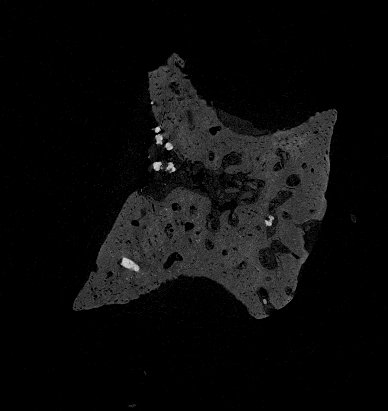

Supplement: S4 File — (ZIP) [file pone.0228610.s004.zip › 29_144/BrI_IR_rec0701.jpg]

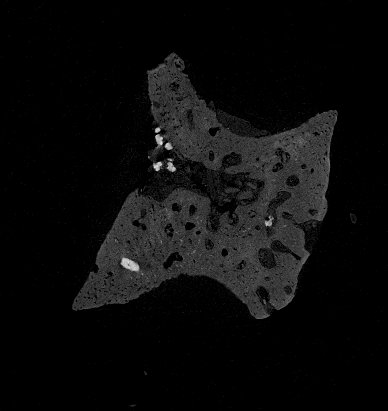

Supplement: S4 File — (ZIP) [file pone.0228610.s004.zip › 29_144/BrI_IR_rec0705.jpg]

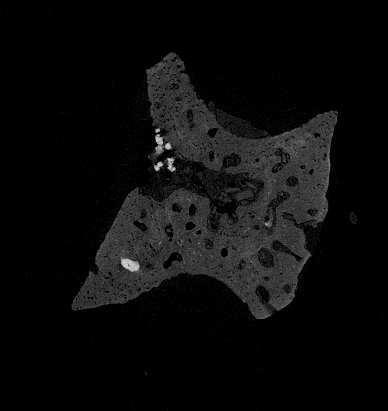

Supplement: S4 File — (ZIP) [file pone.0228610.s004.zip › 29_144/BrI_IR_rec0709.jpg]

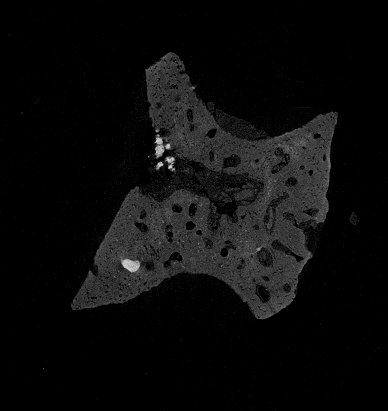

Supplement: S4 File — (ZIP) [file pone.0228610.s004.zip › 29_144/BrI_IR_rec0713.jpg]

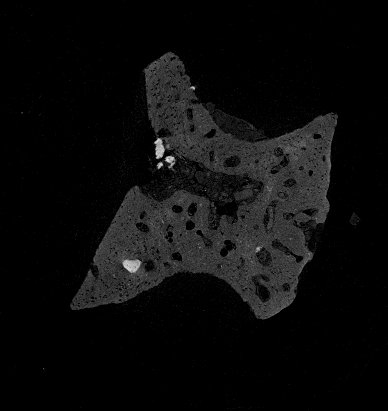

Supplement: S4 File — (ZIP) [file pone.0228610.s004.zip › 29_144/BrI_IR_rec0717.jpg]

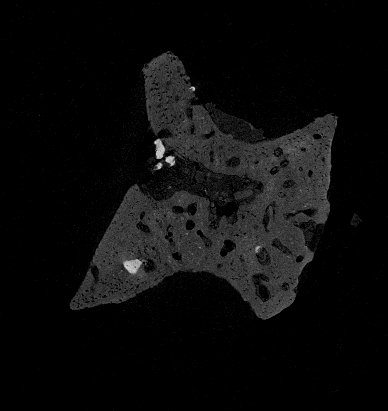

Supplement: S4 File — (ZIP) [file pone.0228610.s004.zip › 29_144/BrI_IR_rec0721.jpg]

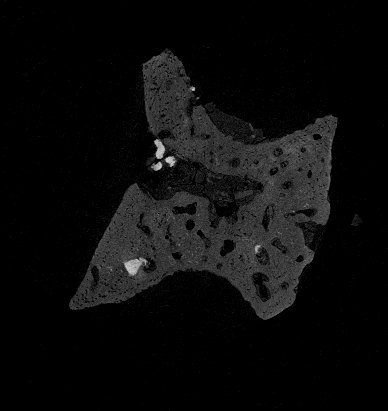

Supplement: S4 File — (ZIP) [file pone.0228610.s004.zip › 29_144/BrI_IR_rec0725.jpg]

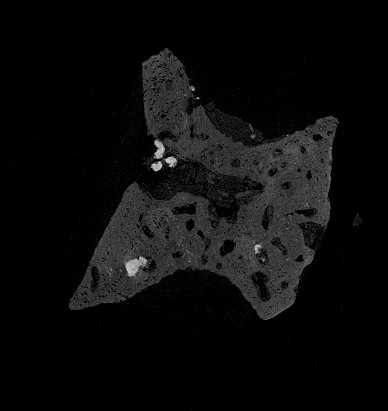

Supplement: S4 File — (ZIP) [file pone.0228610.s004.zip › 29_144/BrI_IR_rec0729.jpg]

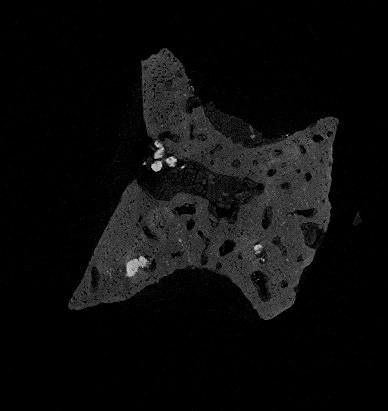

Supplement: S4 File — (ZIP) [file pone.0228610.s004.zip › 29_144/BrI_IR_rec0733.jpg]

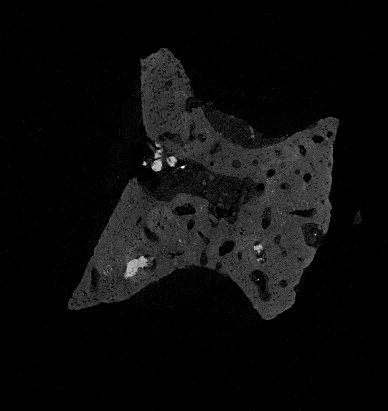

Supplement: S4 File — (ZIP) [file pone.0228610.s004.zip › 29_144/BrI_IR_rec0737.jpg]

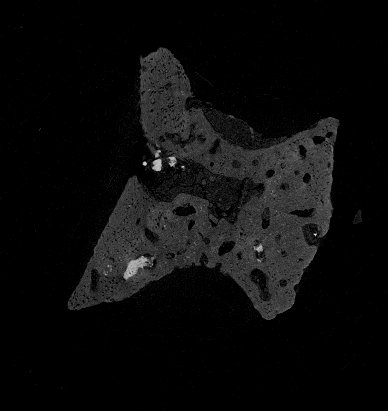

Supplement: S4 File — (ZIP) [file pone.0228610.s004.zip › 29_144/BrI_IR_rec0741.jpg]

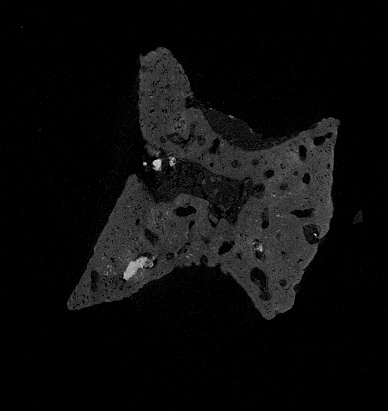

Supplement: S4 File — (ZIP) [file pone.0228610.s004.zip › 29_144/BrI_IR_rec0745.jpg]
