# Supplementary figures and images for: A new small-sized stem salamander from the Middle Jurassic of Western Siberia, Russia (part 10 of 10)
Source: PLoS One. 2020 Feb 19;15(2):e0228610. doi: 10.1371/journal.pone.0228610 (PMC7029856; doi:10.1371/journal.pone.0228610)

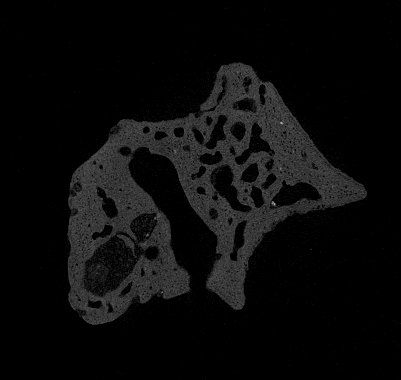

Supplement: S5 File — (ZIP) [file pone.0228610.s005.zip › 32_144/Br-2_IR_rec1818.jpg]

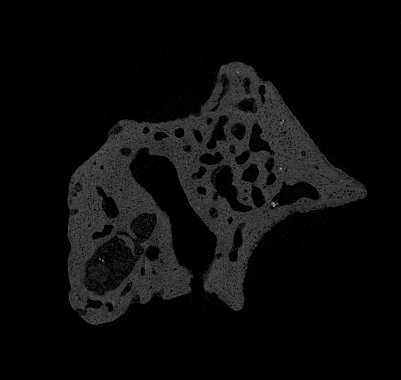

Supplement: S5 File — (ZIP) [file pone.0228610.s005.zip › 32_144/Br-2_IR_rec1822.jpg]

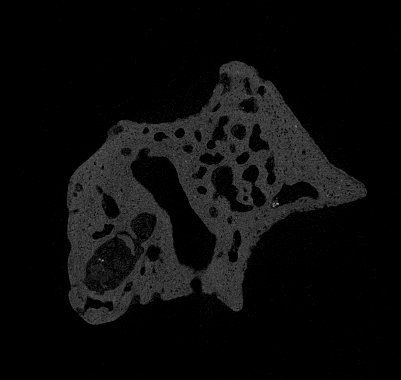

Supplement: S5 File — (ZIP) [file pone.0228610.s005.zip › 32_144/Br-2_IR_rec1826.jpg]

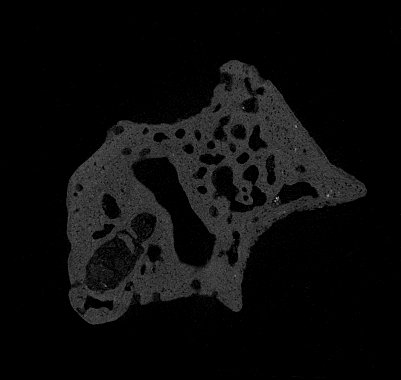

Supplement: S5 File — (ZIP) [file pone.0228610.s005.zip › 32_144/Br-2_IR_rec1830.jpg]

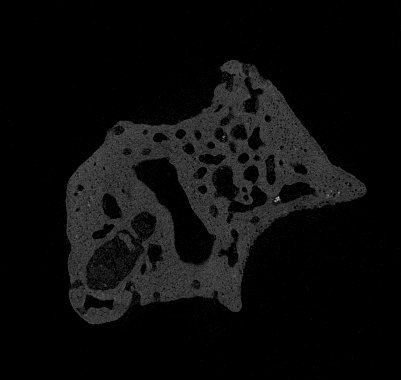

Supplement: S5 File — (ZIP) [file pone.0228610.s005.zip › 32_144/Br-2_IR_rec1834.jpg]

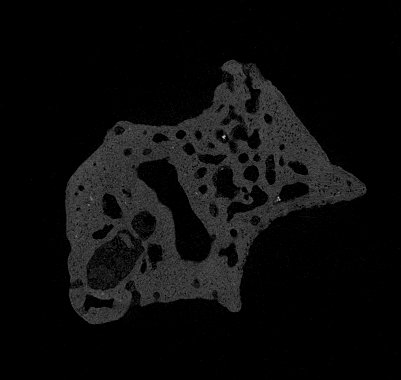

Supplement: S5 File — (ZIP) [file pone.0228610.s005.zip › 32_144/Br-2_IR_rec1838.jpg]

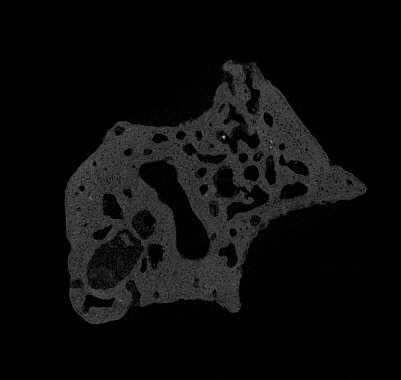

Supplement: S5 File — (ZIP) [file pone.0228610.s005.zip › 32_144/Br-2_IR_rec1842.jpg]

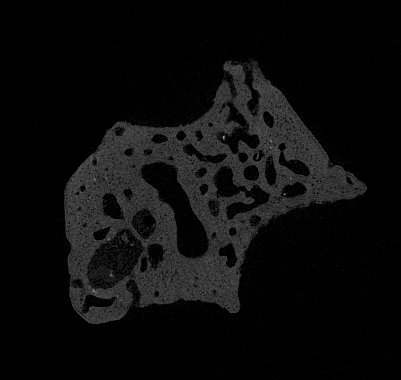

Supplement: S5 File — (ZIP) [file pone.0228610.s005.zip › 32_144/Br-2_IR_rec1846.jpg]

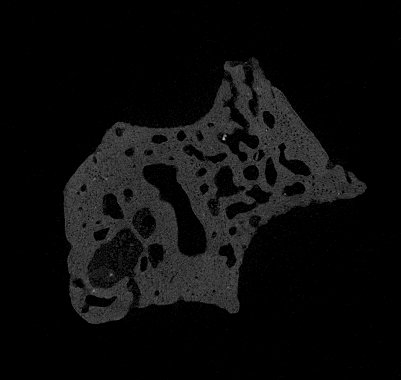

Supplement: S5 File — (ZIP) [file pone.0228610.s005.zip › 32_144/Br-2_IR_rec1850.jpg]

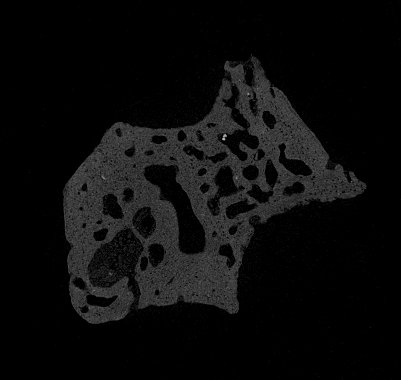

Supplement: S5 File — (ZIP) [file pone.0228610.s005.zip › 32_144/Br-2_IR_rec1854.jpg]

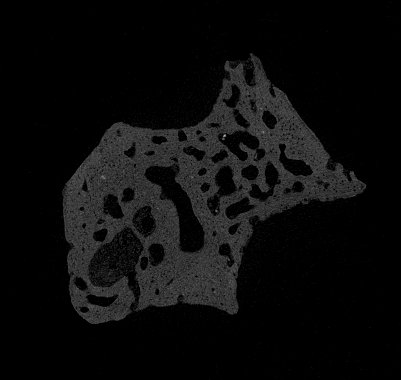

Supplement: S5 File — (ZIP) [file pone.0228610.s005.zip › 32_144/Br-2_IR_rec1858.jpg]

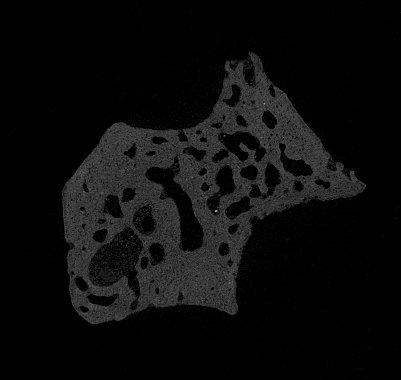

Supplement: S5 File — (ZIP) [file pone.0228610.s005.zip › 32_144/Br-2_IR_rec1862.jpg]

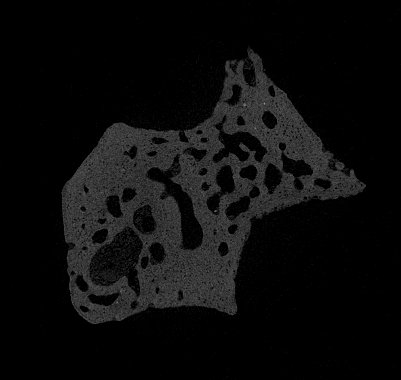

Supplement: S5 File — (ZIP) [file pone.0228610.s005.zip › 32_144/Br-2_IR_rec1866.jpg]

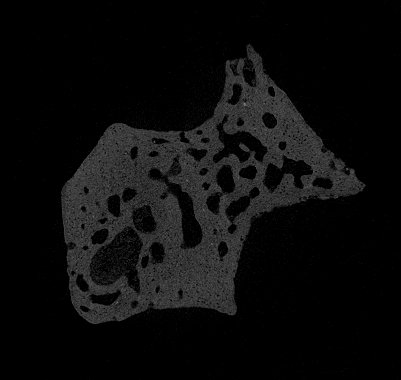

Supplement: S5 File — (ZIP) [file pone.0228610.s005.zip › 32_144/Br-2_IR_rec1870.jpg]

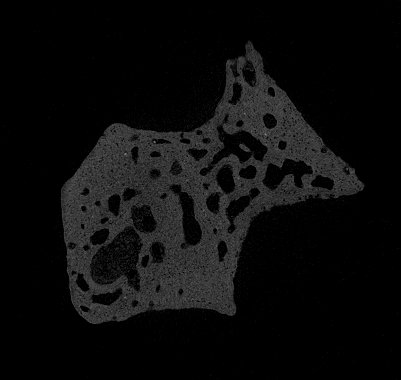

Supplement: S5 File — (ZIP) [file pone.0228610.s005.zip › 32_144/Br-2_IR_rec1874.jpg]

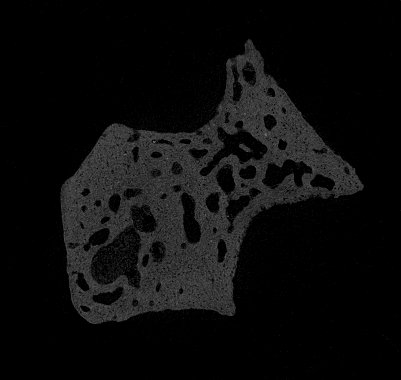

Supplement: S5 File — (ZIP) [file pone.0228610.s005.zip › 32_144/Br-2_IR_rec1878.jpg]

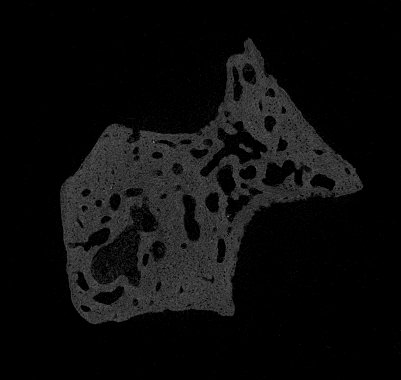

Supplement: S5 File — (ZIP) [file pone.0228610.s005.zip › 32_144/Br-2_IR_rec1882.jpg]

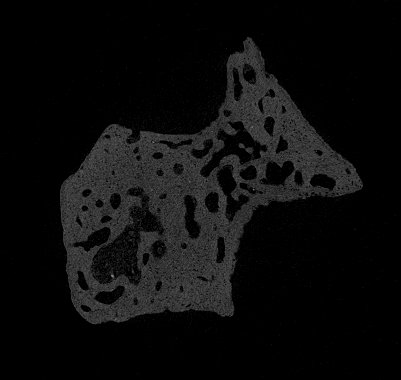

Supplement: S5 File — (ZIP) [file pone.0228610.s005.zip › 32_144/Br-2_IR_rec1886.jpg]

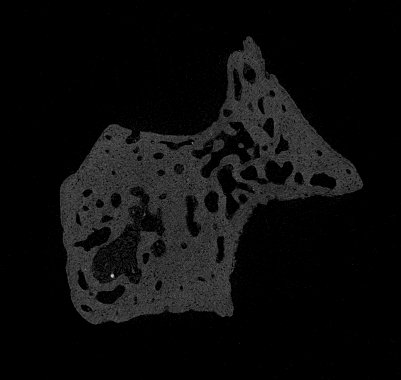

Supplement: S5 File — (ZIP) [file pone.0228610.s005.zip › 32_144/Br-2_IR_rec1890.jpg]

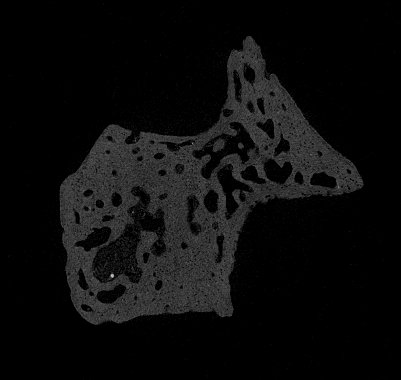

Supplement: S5 File — (ZIP) [file pone.0228610.s005.zip › 32_144/Br-2_IR_rec1894.jpg]

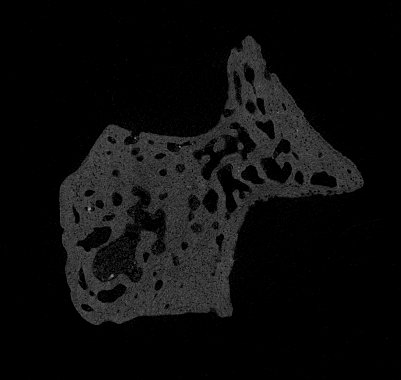

Supplement: S5 File — (ZIP) [file pone.0228610.s005.zip › 32_144/Br-2_IR_rec1898.jpg]

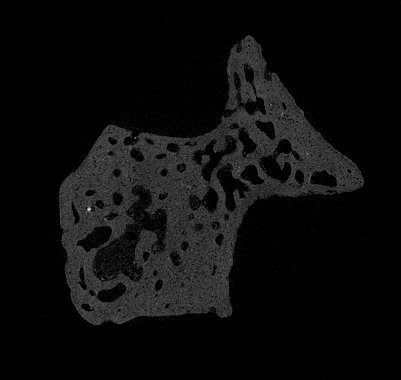

Supplement: S5 File — (ZIP) [file pone.0228610.s005.zip › 32_144/Br-2_IR_rec1902.jpg]

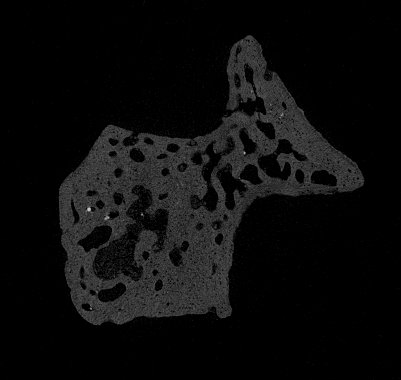

Supplement: S5 File — (ZIP) [file pone.0228610.s005.zip › 32_144/Br-2_IR_rec1906.jpg]

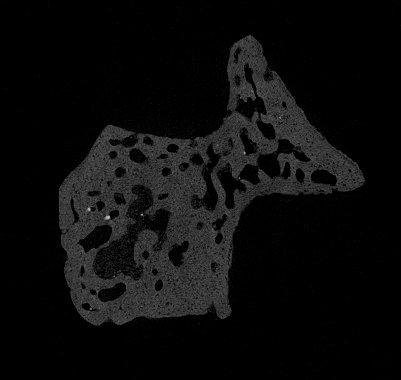

Supplement: S5 File — (ZIP) [file pone.0228610.s005.zip › 32_144/Br-2_IR_rec1910.jpg]

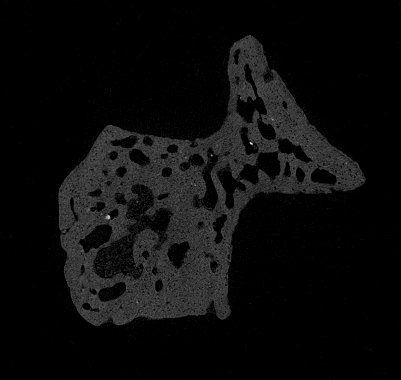

Supplement: S5 File — (ZIP) [file pone.0228610.s005.zip › 32_144/Br-2_IR_rec1914.jpg]

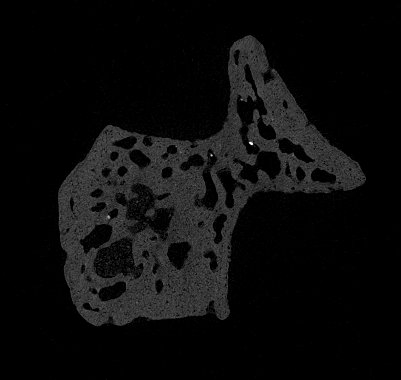

Supplement: S5 File — (ZIP) [file pone.0228610.s005.zip › 32_144/Br-2_IR_rec1918.jpg]

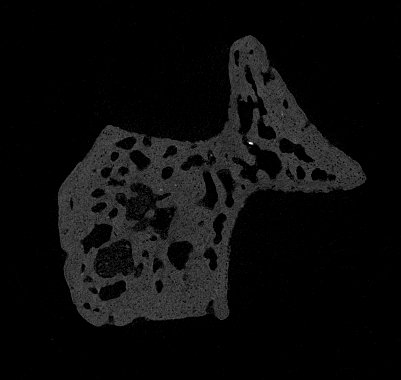

Supplement: S5 File — (ZIP) [file pone.0228610.s005.zip › 32_144/Br-2_IR_rec1922.jpg]

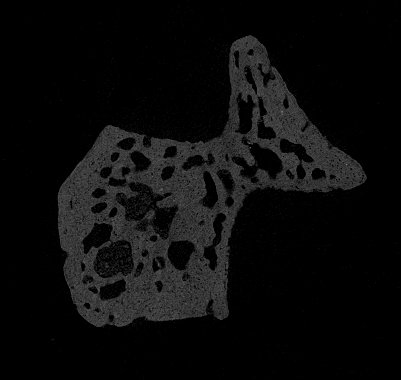

Supplement: S5 File — (ZIP) [file pone.0228610.s005.zip › 32_144/Br-2_IR_rec1926.jpg]

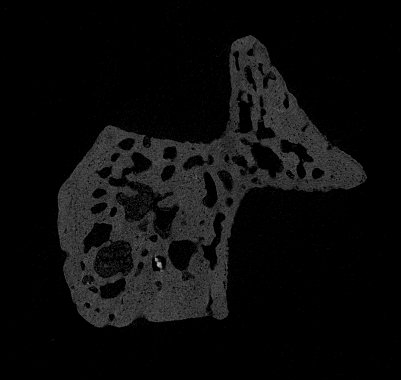

Supplement: S5 File — (ZIP) [file pone.0228610.s005.zip › 32_144/Br-2_IR_rec1930.jpg]

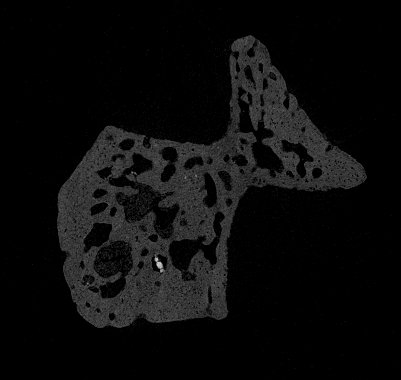

Supplement: S5 File — (ZIP) [file pone.0228610.s005.zip › 32_144/Br-2_IR_rec1934.jpg]

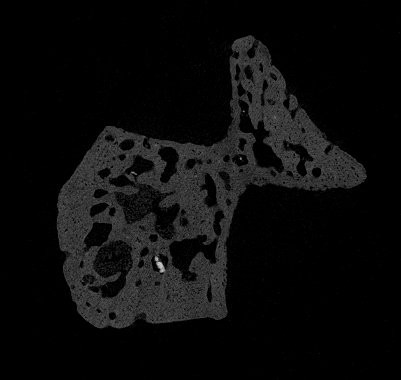

Supplement: S5 File — (ZIP) [file pone.0228610.s005.zip › 32_144/Br-2_IR_rec1938.jpg]

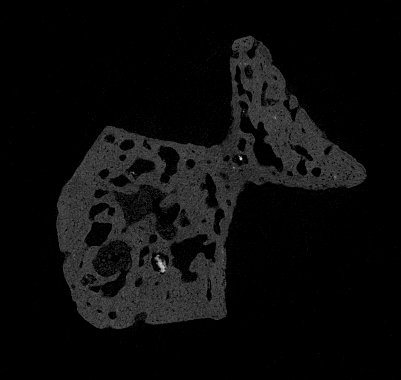

Supplement: S5 File — (ZIP) [file pone.0228610.s005.zip › 32_144/Br-2_IR_rec1942.jpg]

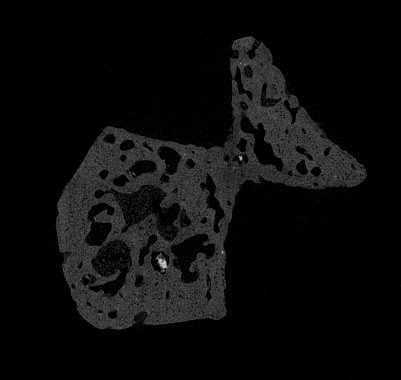

Supplement: S5 File — (ZIP) [file pone.0228610.s005.zip › 32_144/Br-2_IR_rec1946.jpg]

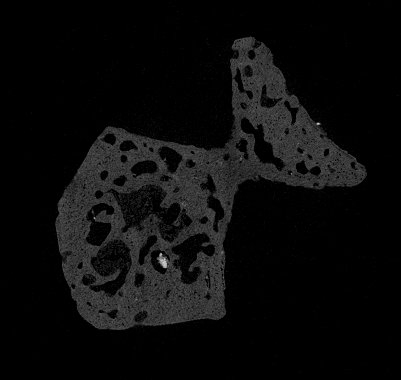

Supplement: S5 File — (ZIP) [file pone.0228610.s005.zip › 32_144/Br-2_IR_rec1950.jpg]

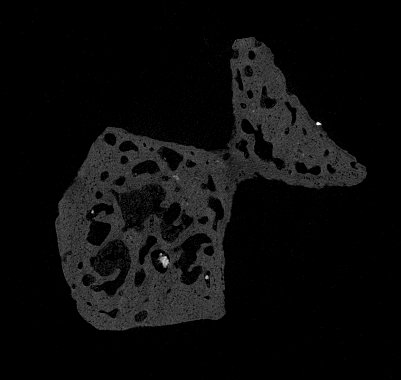

Supplement: S5 File — (ZIP) [file pone.0228610.s005.zip › 32_144/Br-2_IR_rec1954.jpg]

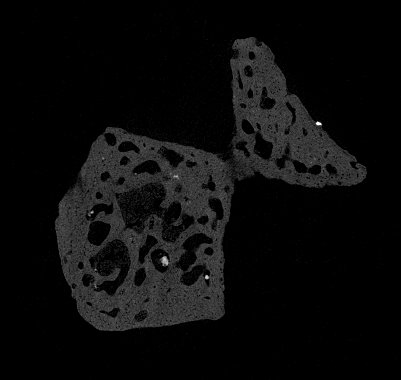

Supplement: S5 File — (ZIP) [file pone.0228610.s005.zip › 32_144/Br-2_IR_rec1958.jpg]

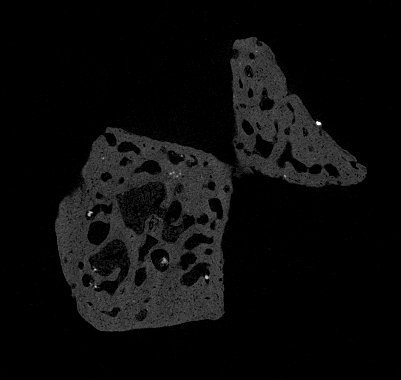

Supplement: S5 File — (ZIP) [file pone.0228610.s005.zip › 32_144/Br-2_IR_rec1962.jpg]

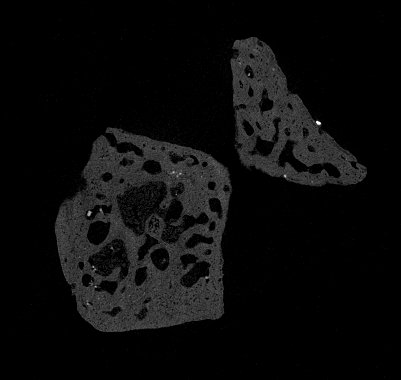

Supplement: S5 File — (ZIP) [file pone.0228610.s005.zip › 32_144/Br-2_IR_rec1966.jpg]

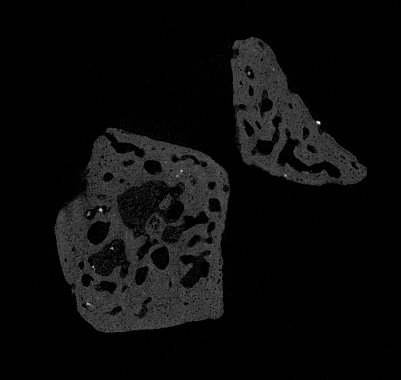

Supplement: S5 File — (ZIP) [file pone.0228610.s005.zip › 32_144/Br-2_IR_rec1970.jpg]

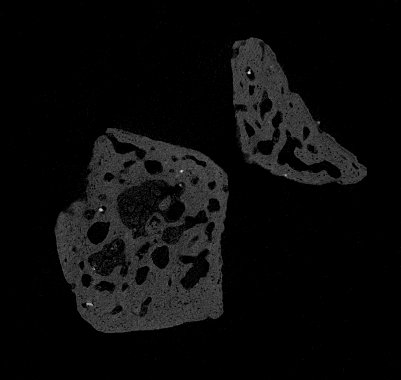

Supplement: S5 File — (ZIP) [file pone.0228610.s005.zip › 32_144/Br-2_IR_rec1974.jpg]

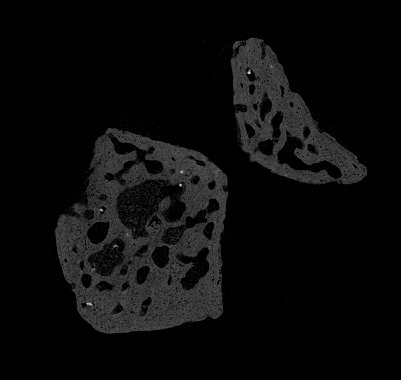

Supplement: S5 File — (ZIP) [file pone.0228610.s005.zip › 32_144/Br-2_IR_rec1978.jpg]

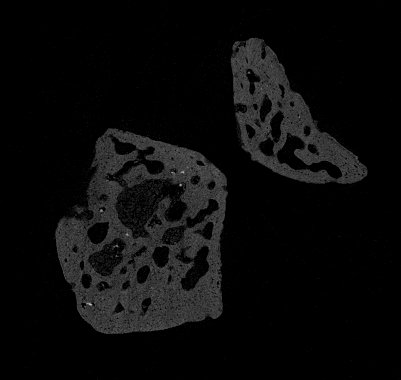

Supplement: S5 File — (ZIP) [file pone.0228610.s005.zip › 32_144/Br-2_IR_rec1982.jpg]

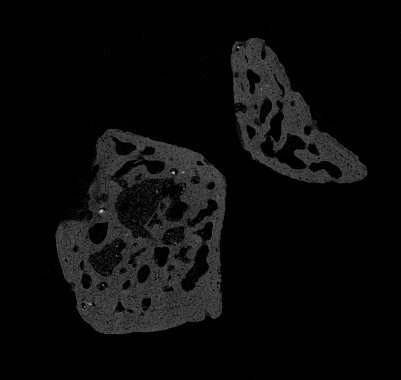

Supplement: S5 File — (ZIP) [file pone.0228610.s005.zip › 32_144/Br-2_IR_rec1986.jpg]

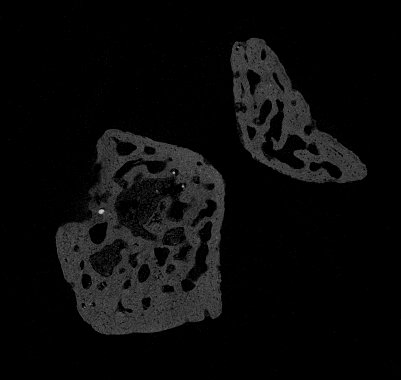

Supplement: S5 File — (ZIP) [file pone.0228610.s005.zip › 32_144/Br-2_IR_rec1990.jpg]

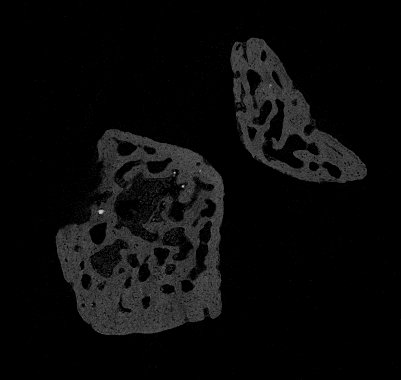

Supplement: S5 File — (ZIP) [file pone.0228610.s005.zip › 32_144/Br-2_IR_rec1994.jpg]

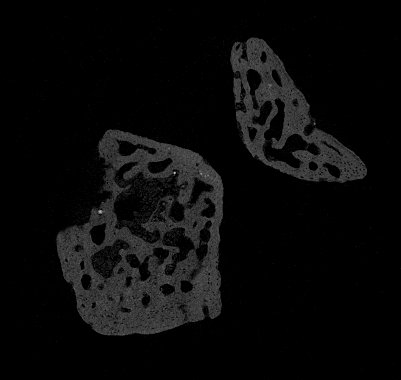

Supplement: S5 File — (ZIP) [file pone.0228610.s005.zip › 32_144/Br-2_IR_rec1998.jpg]

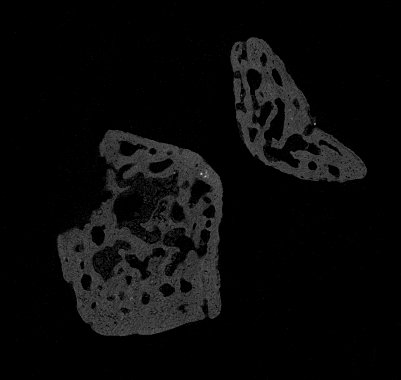

Supplement: S5 File — (ZIP) [file pone.0228610.s005.zip › 32_144/Br-2_IR_rec2002.jpg]

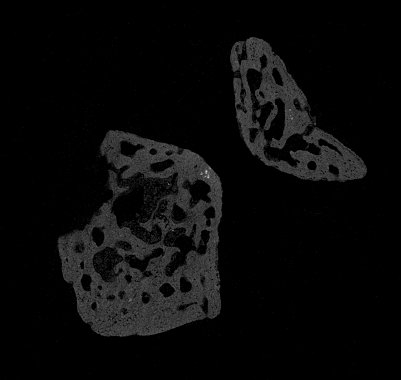

Supplement: S5 File — (ZIP) [file pone.0228610.s005.zip › 32_144/Br-2_IR_rec2006.jpg]

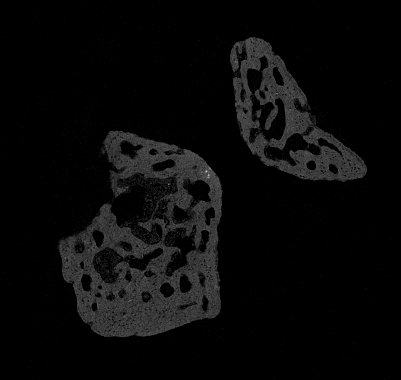

Supplement: S5 File — (ZIP) [file pone.0228610.s005.zip › 32_144/Br-2_IR_rec2010.jpg]

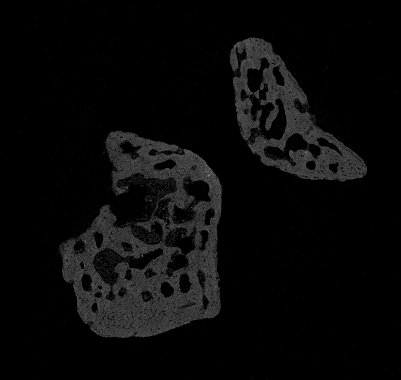

Supplement: S5 File — (ZIP) [file pone.0228610.s005.zip › 32_144/Br-2_IR_rec2014.jpg]

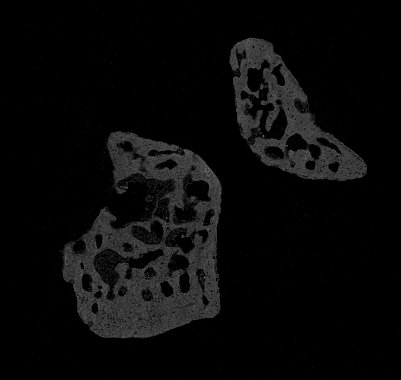

Supplement: S5 File — (ZIP) [file pone.0228610.s005.zip › 32_144/Br-2_IR_rec2018.jpg]

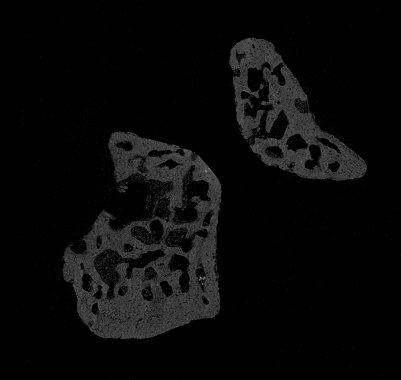

Supplement: S5 File — (ZIP) [file pone.0228610.s005.zip › 32_144/Br-2_IR_rec2022.jpg]

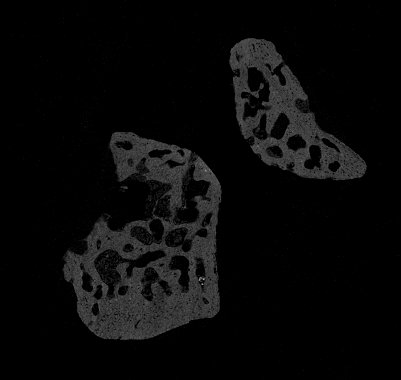

Supplement: S5 File — (ZIP) [file pone.0228610.s005.zip › 32_144/Br-2_IR_rec2026.jpg]

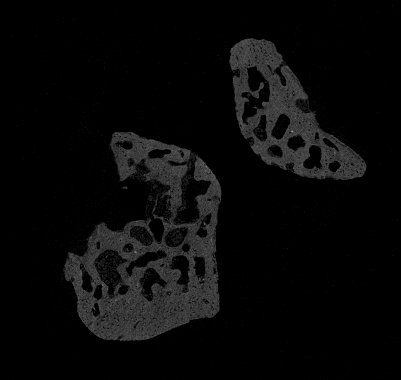

Supplement: S5 File — (ZIP) [file pone.0228610.s005.zip › 32_144/Br-2_IR_rec2030.jpg]

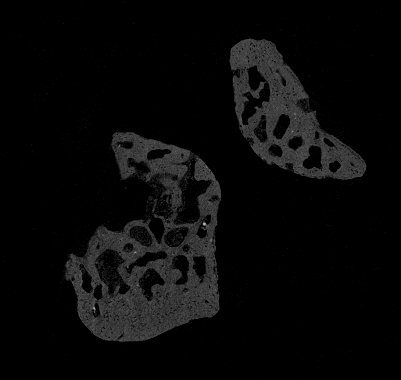

Supplement: S5 File — (ZIP) [file pone.0228610.s005.zip › 32_144/Br-2_IR_rec2034.jpg]

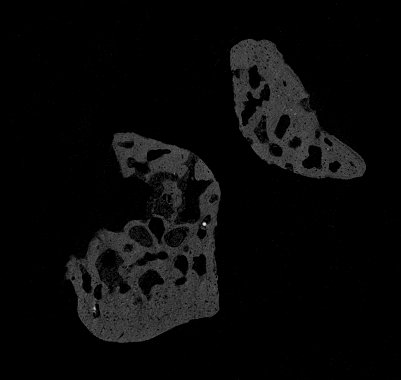

Supplement: S5 File — (ZIP) [file pone.0228610.s005.zip › 32_144/Br-2_IR_rec2038.jpg]

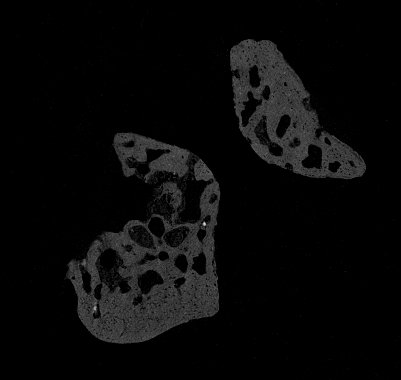

Supplement: S5 File — (ZIP) [file pone.0228610.s005.zip › 32_144/Br-2_IR_rec2042.jpg]

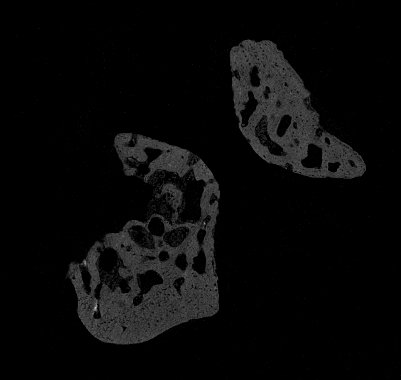

Supplement: S5 File — (ZIP) [file pone.0228610.s005.zip › 32_144/Br-2_IR_rec2046.jpg]

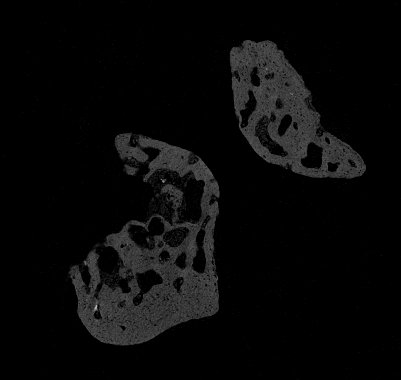

Supplement: S5 File — (ZIP) [file pone.0228610.s005.zip › 32_144/Br-2_IR_rec2050.jpg]

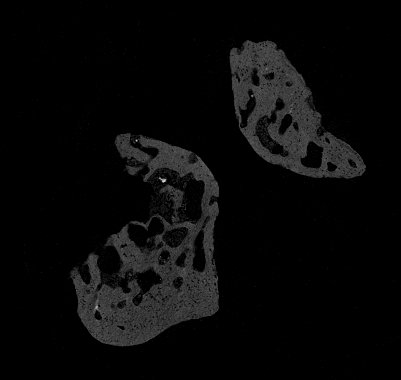

Supplement: S5 File — (ZIP) [file pone.0228610.s005.zip › 32_144/Br-2_IR_rec2054.jpg]

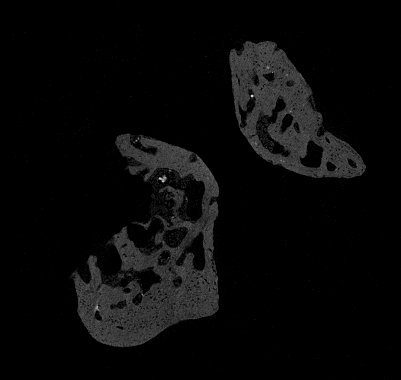

Supplement: S5 File — (ZIP) [file pone.0228610.s005.zip › 32_144/Br-2_IR_rec2058.jpg]

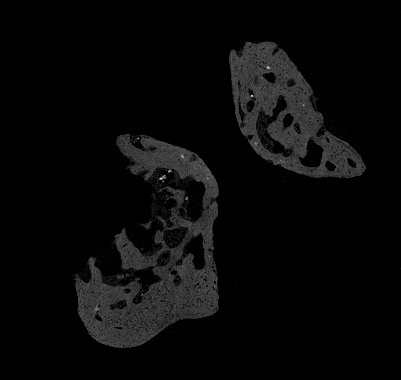

Supplement: S5 File — (ZIP) [file pone.0228610.s005.zip › 32_144/Br-2_IR_rec2062.jpg]

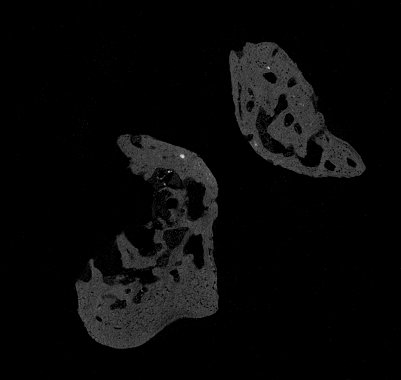

Supplement: S5 File — (ZIP) [file pone.0228610.s005.zip › 32_144/Br-2_IR_rec2066.jpg]

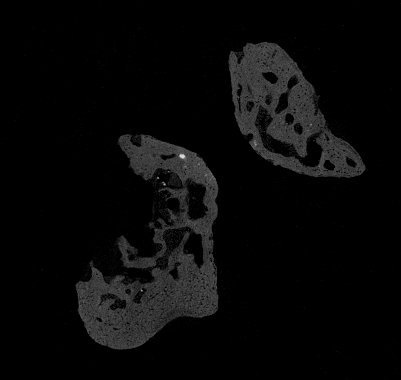

Supplement: S5 File — (ZIP) [file pone.0228610.s005.zip › 32_144/Br-2_IR_rec2070.jpg]

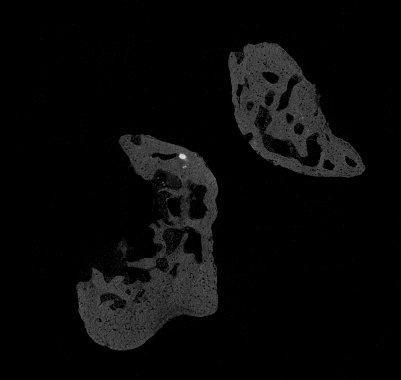

Supplement: S5 File — (ZIP) [file pone.0228610.s005.zip › 32_144/Br-2_IR_rec2074.jpg]

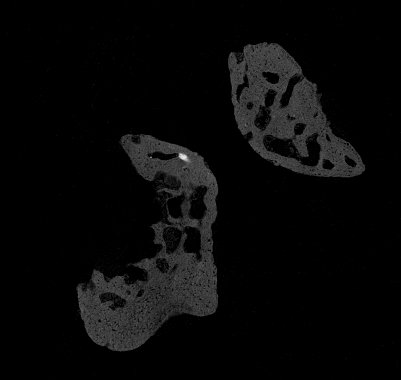

Supplement: S5 File — (ZIP) [file pone.0228610.s005.zip › 32_144/Br-2_IR_rec2078.jpg]

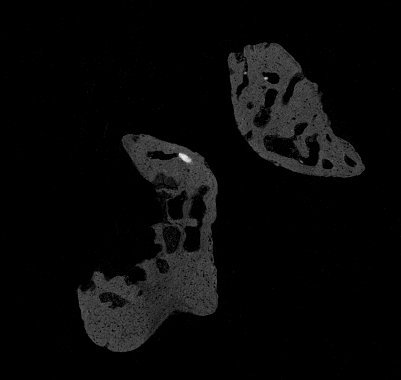

Supplement: S5 File — (ZIP) [file pone.0228610.s005.zip › 32_144/Br-2_IR_rec2082.jpg]

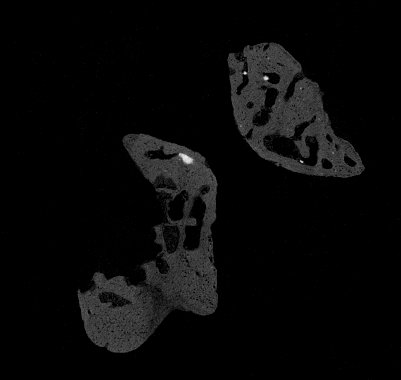

Supplement: S5 File — (ZIP) [file pone.0228610.s005.zip › 32_144/Br-2_IR_rec2086.jpg]

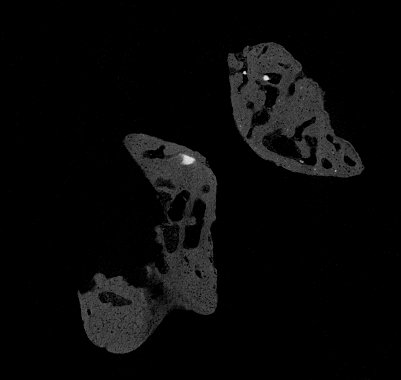

Supplement: S5 File — (ZIP) [file pone.0228610.s005.zip › 32_144/Br-2_IR_rec2090.jpg]

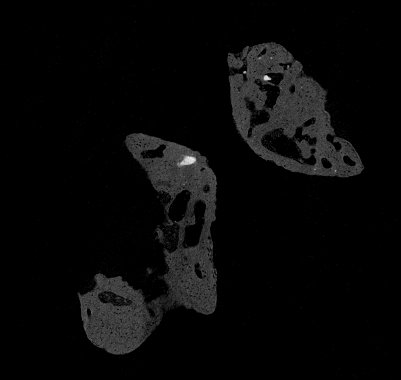

Supplement: S5 File — (ZIP) [file pone.0228610.s005.zip › 32_144/Br-2_IR_rec2094.jpg]

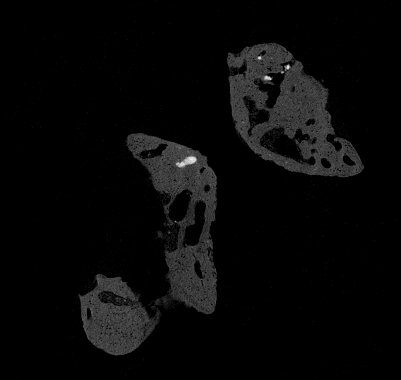

Supplement: S5 File — (ZIP) [file pone.0228610.s005.zip › 32_144/Br-2_IR_rec2098.jpg]

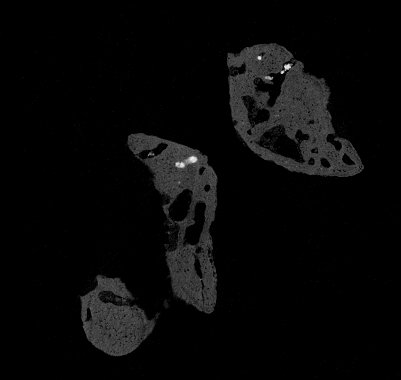

Supplement: S5 File — (ZIP) [file pone.0228610.s005.zip › 32_144/Br-2_IR_rec2102.jpg]

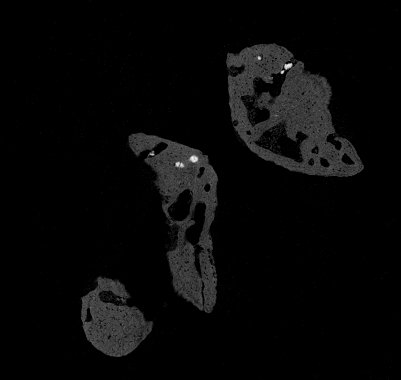

Supplement: S5 File — (ZIP) [file pone.0228610.s005.zip › 32_144/Br-2_IR_rec2106.jpg]

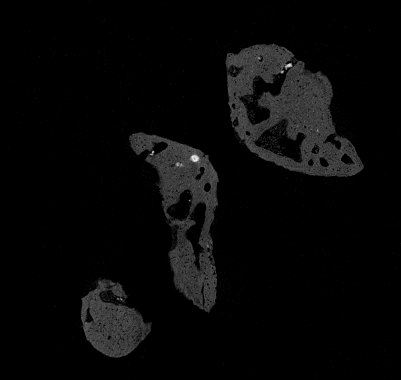

Supplement: S5 File — (ZIP) [file pone.0228610.s005.zip › 32_144/Br-2_IR_rec2110.jpg]

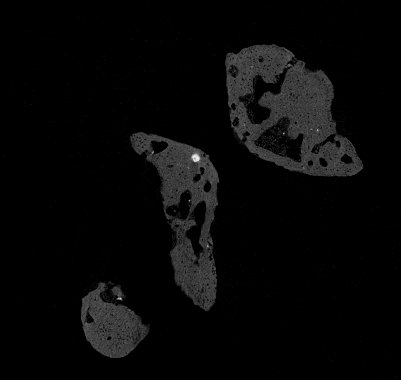

Supplement: S5 File — (ZIP) [file pone.0228610.s005.zip › 32_144/Br-2_IR_rec2114.jpg]

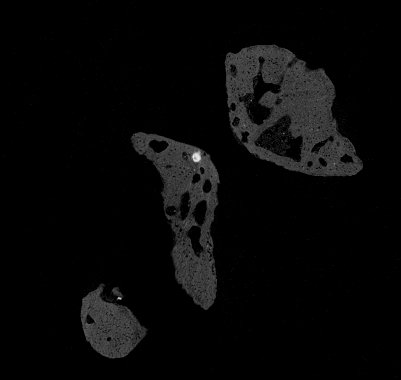

Supplement: S5 File — (ZIP) [file pone.0228610.s005.zip › 32_144/Br-2_IR_rec2118.jpg]

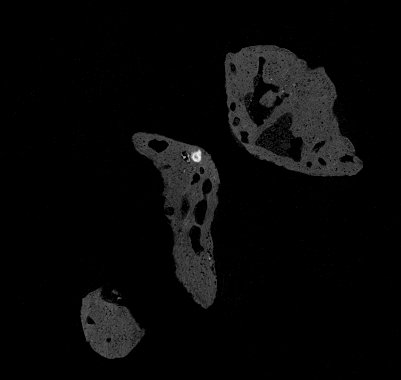

Supplement: S5 File — (ZIP) [file pone.0228610.s005.zip › 32_144/Br-2_IR_rec2122.jpg]

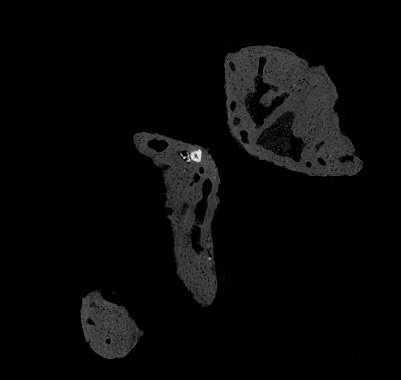

Supplement: S5 File — (ZIP) [file pone.0228610.s005.zip › 32_144/Br-2_IR_rec2126.jpg]

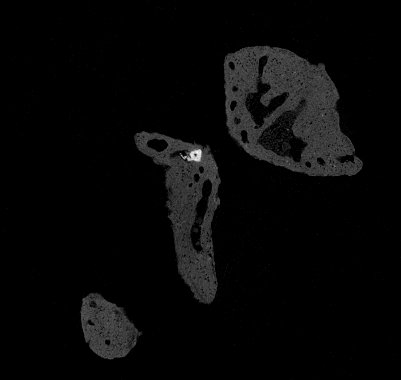

Supplement: S5 File — (ZIP) [file pone.0228610.s005.zip › 32_144/Br-2_IR_rec2130.jpg]
