# Supplementary figures and images for: A new small-sized stem salamander from the Middle Jurassic of Western Siberia, Russia (part 9 of 10)
Source: PLoS One. 2020 Feb 19;15(2):e0228610. doi: 10.1371/journal.pone.0228610 (PMC7029856; doi:10.1371/journal.pone.0228610)

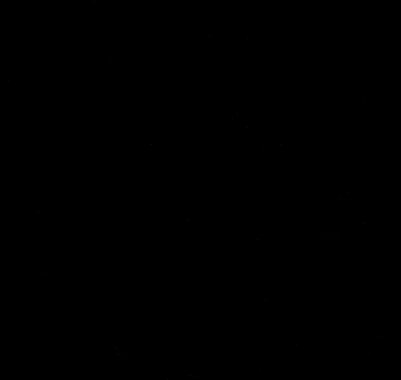

Supplement: S5 File — (ZIP) [file pone.0228610.s005.zip › 32_144/Br-2_IR_rec1418.jpg]

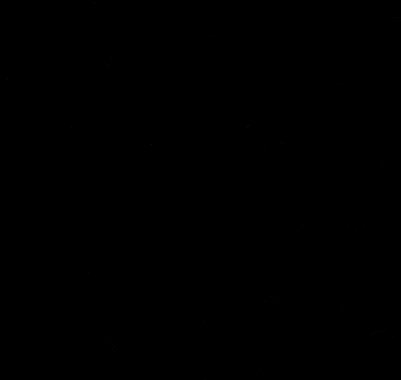

Supplement: S5 File — (ZIP) [file pone.0228610.s005.zip › 32_144/Br-2_IR_rec1422.jpg]

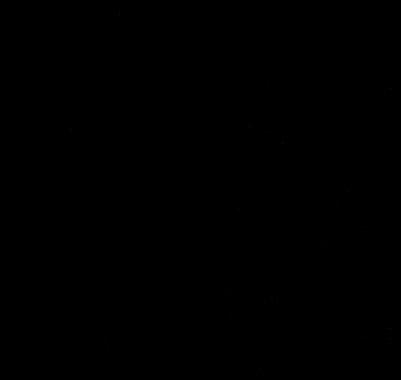

Supplement: S5 File — (ZIP) [file pone.0228610.s005.zip › 32_144/Br-2_IR_rec1426.jpg]

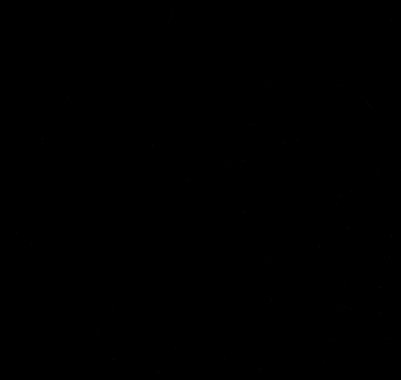

Supplement: S5 File — (ZIP) [file pone.0228610.s005.zip › 32_144/Br-2_IR_rec1430.jpg]

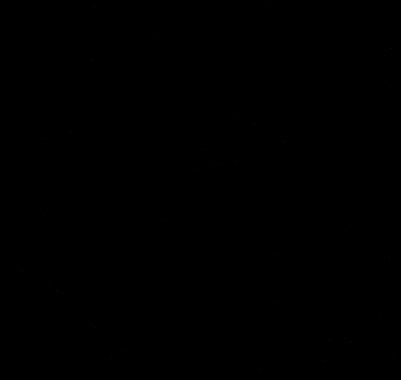

Supplement: S5 File — (ZIP) [file pone.0228610.s005.zip › 32_144/Br-2_IR_rec1434.jpg]

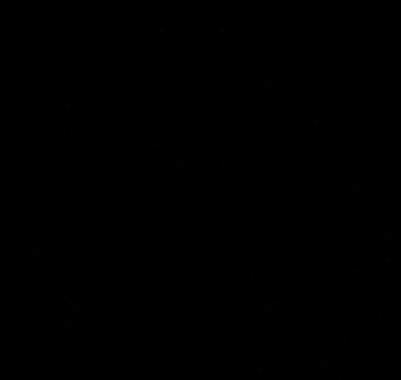

Supplement: S5 File — (ZIP) [file pone.0228610.s005.zip › 32_144/Br-2_IR_rec1438.jpg]

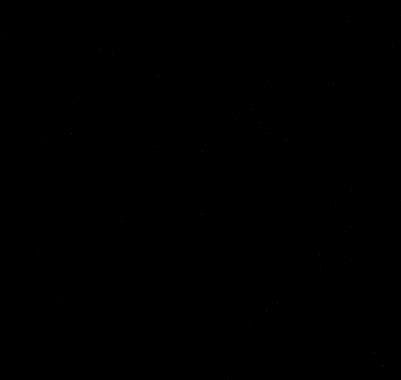

Supplement: S5 File — (ZIP) [file pone.0228610.s005.zip › 32_144/Br-2_IR_rec1442.jpg]

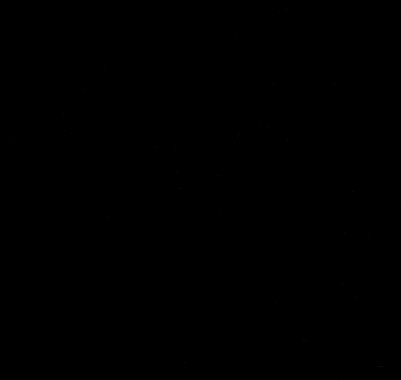

Supplement: S5 File — (ZIP) [file pone.0228610.s005.zip › 32_144/Br-2_IR_rec1446.jpg]

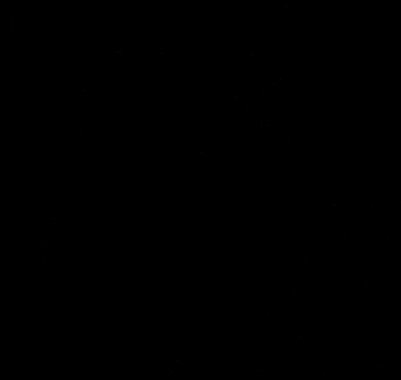

Supplement: S5 File — (ZIP) [file pone.0228610.s005.zip › 32_144/Br-2_IR_rec1450.jpg]

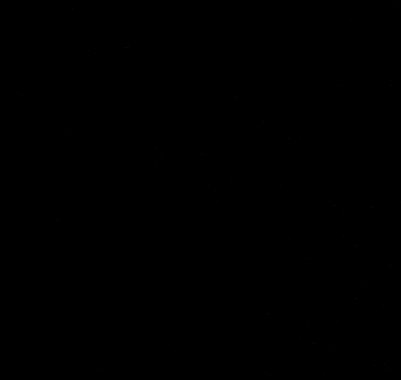

Supplement: S5 File — (ZIP) [file pone.0228610.s005.zip › 32_144/Br-2_IR_rec1454.jpg]

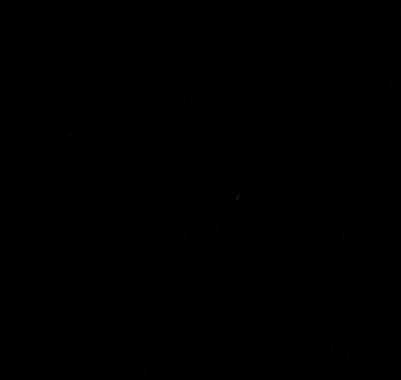

Supplement: S5 File — (ZIP) [file pone.0228610.s005.zip › 32_144/Br-2_IR_rec1458.jpg]

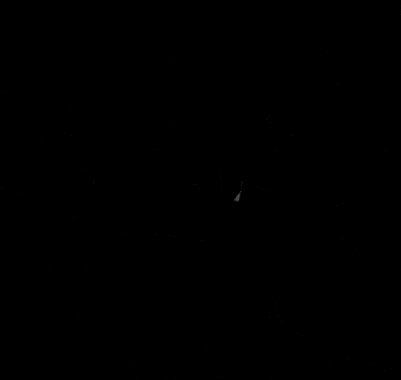

Supplement: S5 File — (ZIP) [file pone.0228610.s005.zip › 32_144/Br-2_IR_rec1462.jpg]

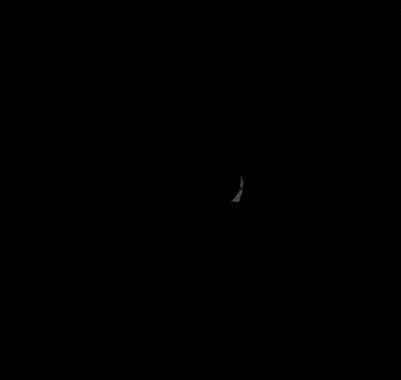

Supplement: S5 File — (ZIP) [file pone.0228610.s005.zip › 32_144/Br-2_IR_rec1466.jpg]

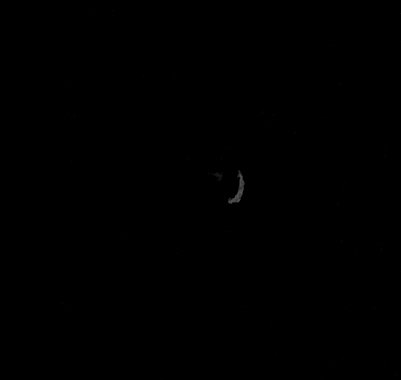

Supplement: S5 File — (ZIP) [file pone.0228610.s005.zip › 32_144/Br-2_IR_rec1470.jpg]

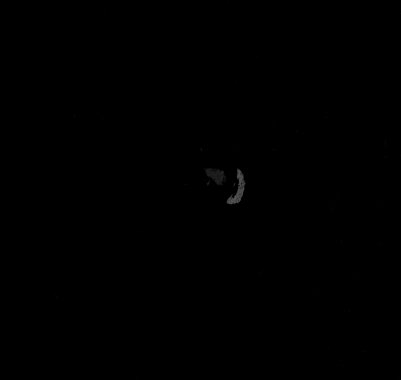

Supplement: S5 File — (ZIP) [file pone.0228610.s005.zip › 32_144/Br-2_IR_rec1474.jpg]

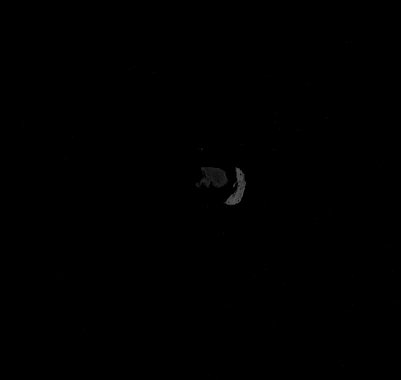

Supplement: S5 File — (ZIP) [file pone.0228610.s005.zip › 32_144/Br-2_IR_rec1478.jpg]

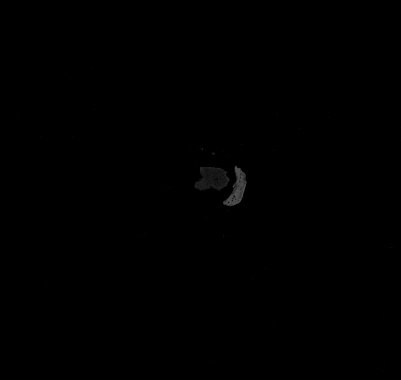

Supplement: S5 File — (ZIP) [file pone.0228610.s005.zip › 32_144/Br-2_IR_rec1482.jpg]

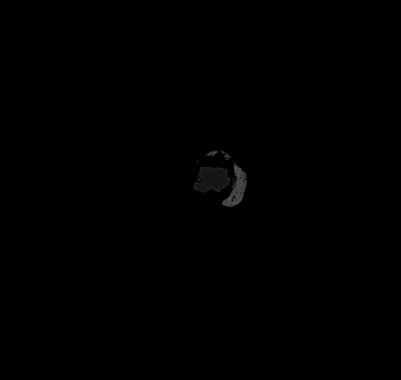

Supplement: S5 File — (ZIP) [file pone.0228610.s005.zip › 32_144/Br-2_IR_rec1486.jpg]

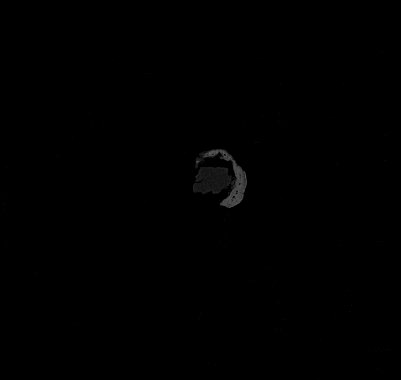

Supplement: S5 File — (ZIP) [file pone.0228610.s005.zip › 32_144/Br-2_IR_rec1490.jpg]

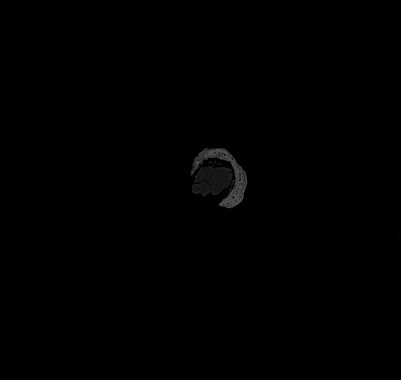

Supplement: S5 File — (ZIP) [file pone.0228610.s005.zip › 32_144/Br-2_IR_rec1494.jpg]

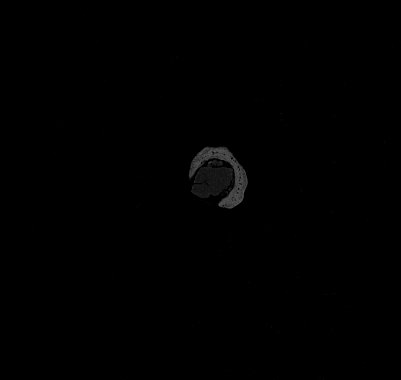

Supplement: S5 File — (ZIP) [file pone.0228610.s005.zip › 32_144/Br-2_IR_rec1498.jpg]

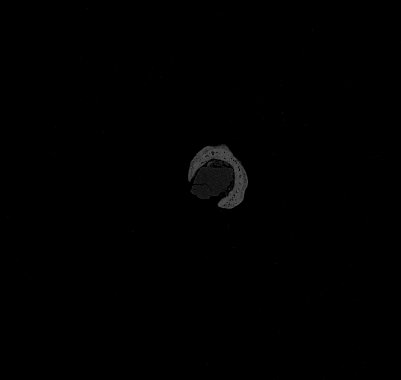

Supplement: S5 File — (ZIP) [file pone.0228610.s005.zip › 32_144/Br-2_IR_rec1502.jpg]

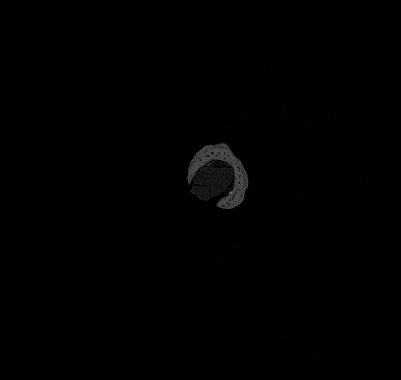

Supplement: S5 File — (ZIP) [file pone.0228610.s005.zip › 32_144/Br-2_IR_rec1506.jpg]

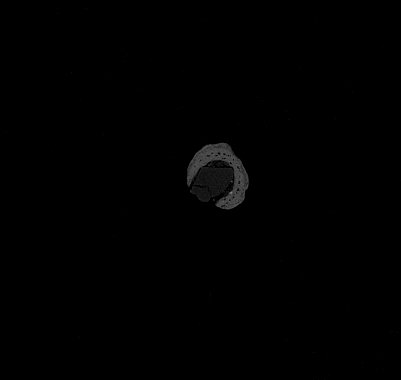

Supplement: S5 File — (ZIP) [file pone.0228610.s005.zip › 32_144/Br-2_IR_rec1510.jpg]

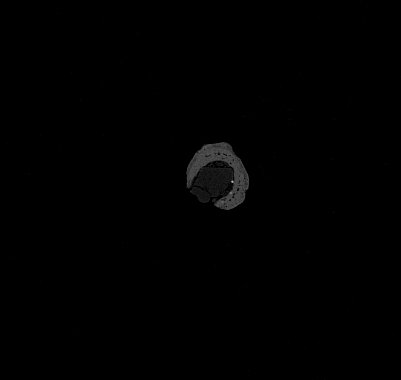

Supplement: S5 File — (ZIP) [file pone.0228610.s005.zip › 32_144/Br-2_IR_rec1514.jpg]

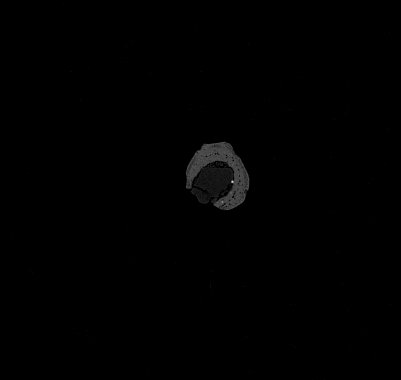

Supplement: S5 File — (ZIP) [file pone.0228610.s005.zip › 32_144/Br-2_IR_rec1518.jpg]

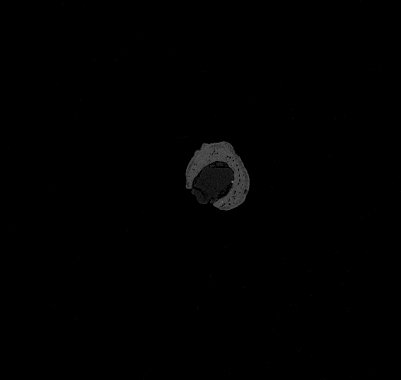

Supplement: S5 File — (ZIP) [file pone.0228610.s005.zip › 32_144/Br-2_IR_rec1522.jpg]

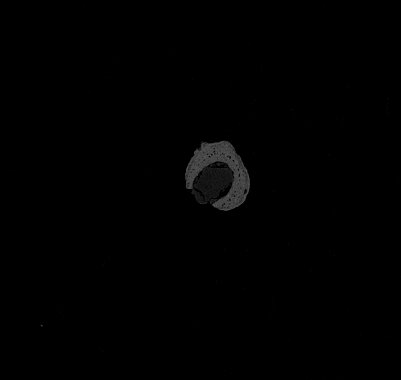

Supplement: S5 File — (ZIP) [file pone.0228610.s005.zip › 32_144/Br-2_IR_rec1526.jpg]

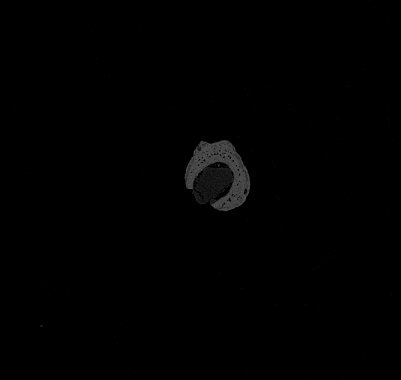

Supplement: S5 File — (ZIP) [file pone.0228610.s005.zip › 32_144/Br-2_IR_rec1530.jpg]

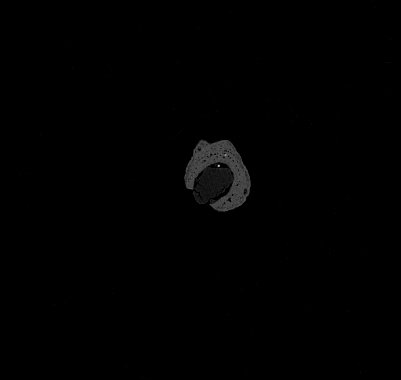

Supplement: S5 File — (ZIP) [file pone.0228610.s005.zip › 32_144/Br-2_IR_rec1534.jpg]

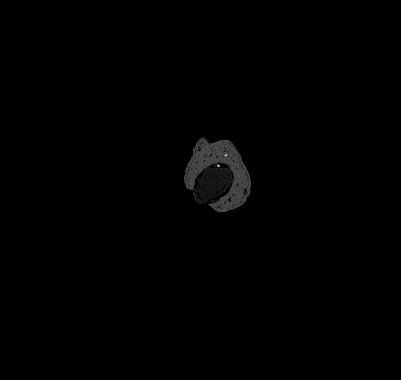

Supplement: S5 File — (ZIP) [file pone.0228610.s005.zip › 32_144/Br-2_IR_rec1538.jpg]

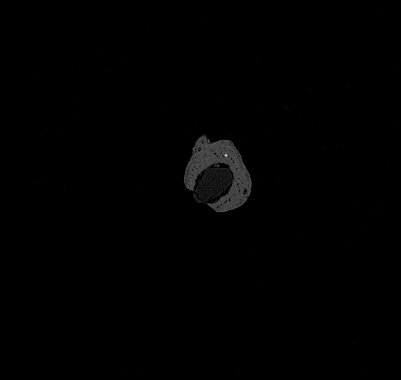

Supplement: S5 File — (ZIP) [file pone.0228610.s005.zip › 32_144/Br-2_IR_rec1542.jpg]

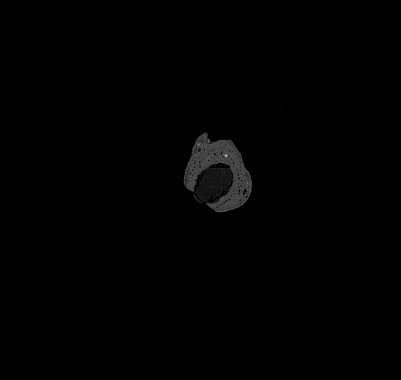

Supplement: S5 File — (ZIP) [file pone.0228610.s005.zip › 32_144/Br-2_IR_rec1546.jpg]

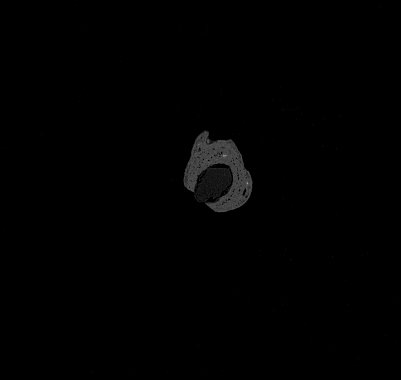

Supplement: S5 File — (ZIP) [file pone.0228610.s005.zip › 32_144/Br-2_IR_rec1550.jpg]

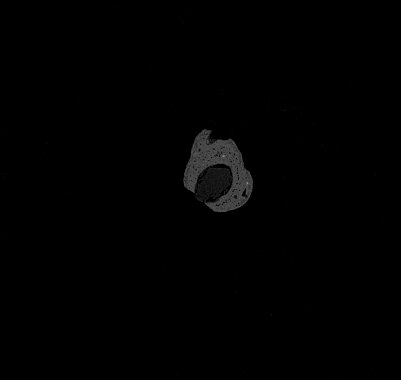

Supplement: S5 File — (ZIP) [file pone.0228610.s005.zip › 32_144/Br-2_IR_rec1554.jpg]

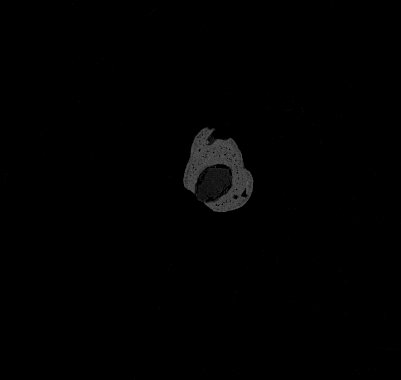

Supplement: S5 File — (ZIP) [file pone.0228610.s005.zip › 32_144/Br-2_IR_rec1558.jpg]

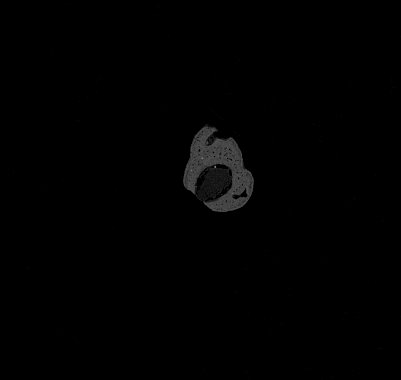

Supplement: S5 File — (ZIP) [file pone.0228610.s005.zip › 32_144/Br-2_IR_rec1562.jpg]

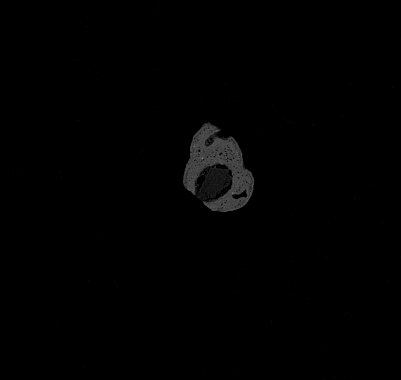

Supplement: S5 File — (ZIP) [file pone.0228610.s005.zip › 32_144/Br-2_IR_rec1566.jpg]

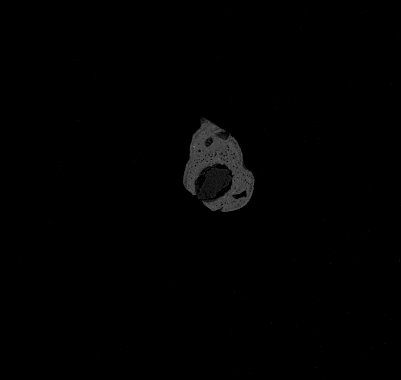

Supplement: S5 File — (ZIP) [file pone.0228610.s005.zip › 32_144/Br-2_IR_rec1570.jpg]

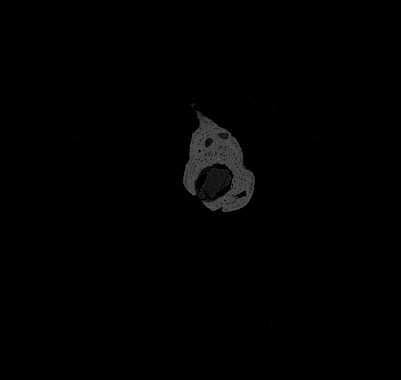

Supplement: S5 File — (ZIP) [file pone.0228610.s005.zip › 32_144/Br-2_IR_rec1574.jpg]

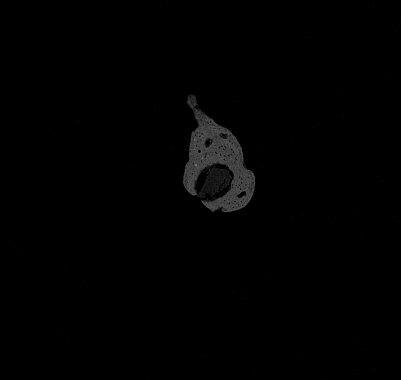

Supplement: S5 File — (ZIP) [file pone.0228610.s005.zip › 32_144/Br-2_IR_rec1578.jpg]

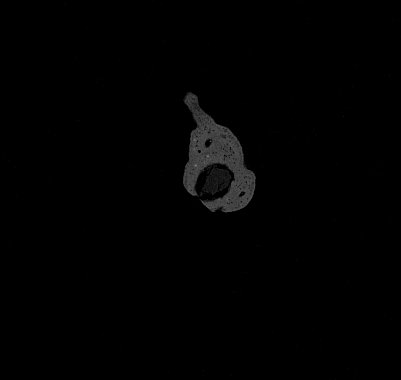

Supplement: S5 File — (ZIP) [file pone.0228610.s005.zip › 32_144/Br-2_IR_rec1582.jpg]

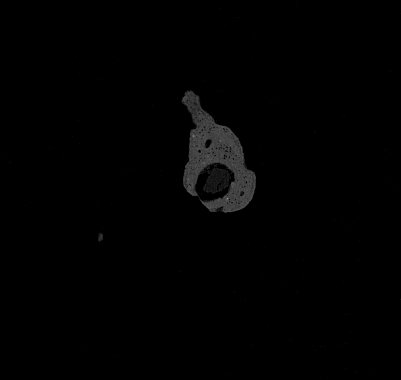

Supplement: S5 File — (ZIP) [file pone.0228610.s005.zip › 32_144/Br-2_IR_rec1586.jpg]

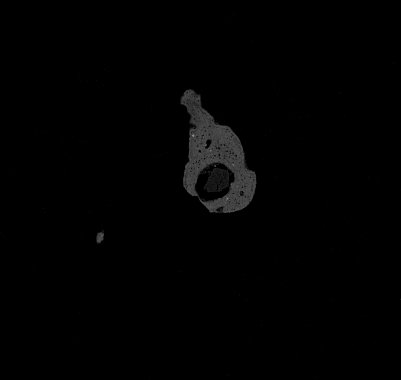

Supplement: S5 File — (ZIP) [file pone.0228610.s005.zip › 32_144/Br-2_IR_rec1590.jpg]

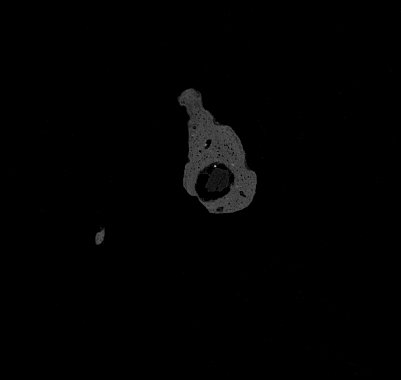

Supplement: S5 File — (ZIP) [file pone.0228610.s005.zip › 32_144/Br-2_IR_rec1594.jpg]

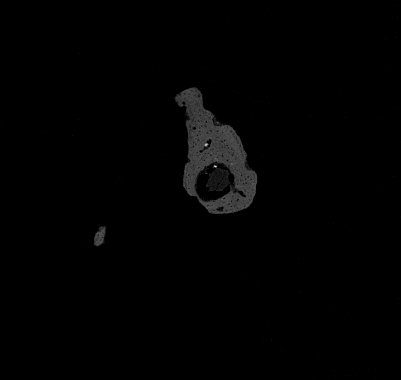

Supplement: S5 File — (ZIP) [file pone.0228610.s005.zip › 32_144/Br-2_IR_rec1598.jpg]

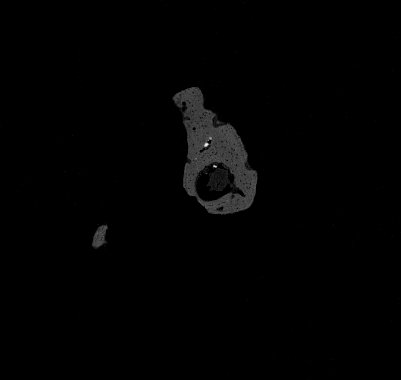

Supplement: S5 File — (ZIP) [file pone.0228610.s005.zip › 32_144/Br-2_IR_rec1602.jpg]

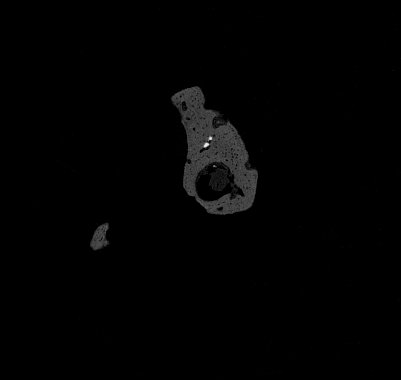

Supplement: S5 File — (ZIP) [file pone.0228610.s005.zip › 32_144/Br-2_IR_rec1606.jpg]

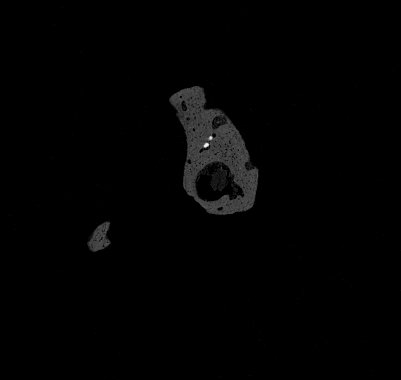

Supplement: S5 File — (ZIP) [file pone.0228610.s005.zip › 32_144/Br-2_IR_rec1610.jpg]

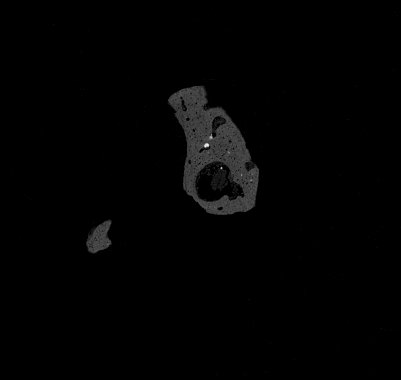

Supplement: S5 File — (ZIP) [file pone.0228610.s005.zip › 32_144/Br-2_IR_rec1614.jpg]

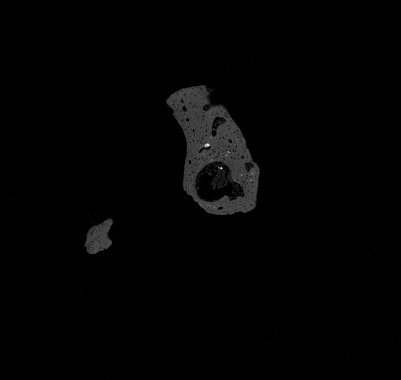

Supplement: S5 File — (ZIP) [file pone.0228610.s005.zip › 32_144/Br-2_IR_rec1618.jpg]

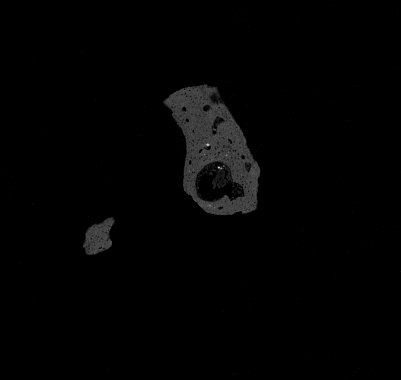

Supplement: S5 File — (ZIP) [file pone.0228610.s005.zip › 32_144/Br-2_IR_rec1622.jpg]

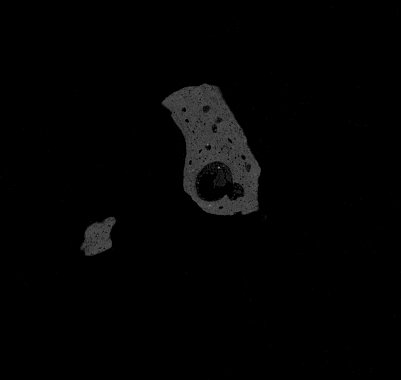

Supplement: S5 File — (ZIP) [file pone.0228610.s005.zip › 32_144/Br-2_IR_rec1626.jpg]

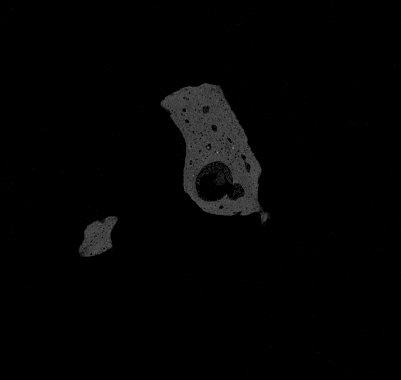

Supplement: S5 File — (ZIP) [file pone.0228610.s005.zip › 32_144/Br-2_IR_rec1630.jpg]

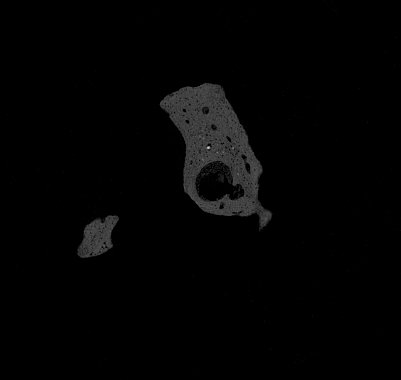

Supplement: S5 File — (ZIP) [file pone.0228610.s005.zip › 32_144/Br-2_IR_rec1634.jpg]

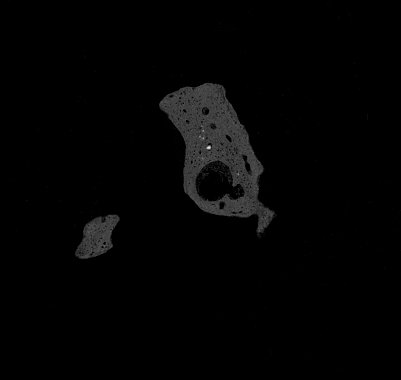

Supplement: S5 File — (ZIP) [file pone.0228610.s005.zip › 32_144/Br-2_IR_rec1638.jpg]

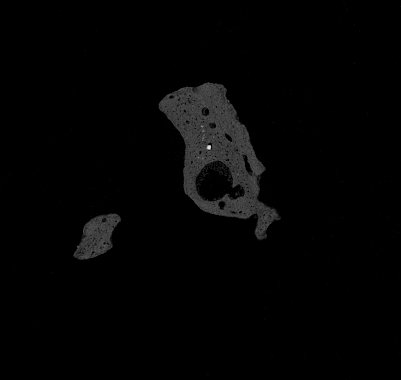

Supplement: S5 File — (ZIP) [file pone.0228610.s005.zip › 32_144/Br-2_IR_rec1642.jpg]

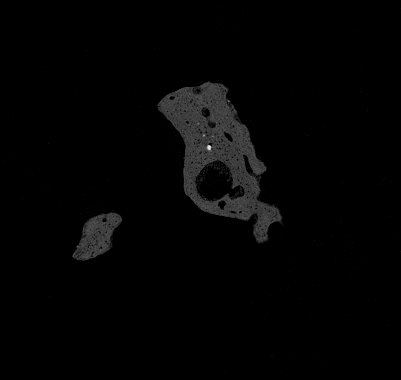

Supplement: S5 File — (ZIP) [file pone.0228610.s005.zip › 32_144/Br-2_IR_rec1646.jpg]

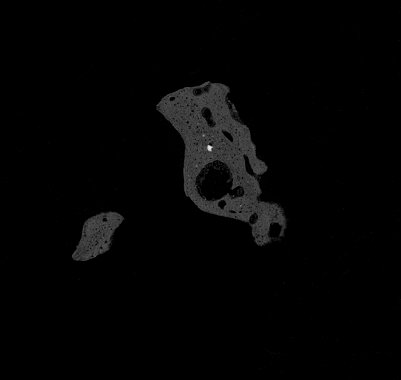

Supplement: S5 File — (ZIP) [file pone.0228610.s005.zip › 32_144/Br-2_IR_rec1650.jpg]

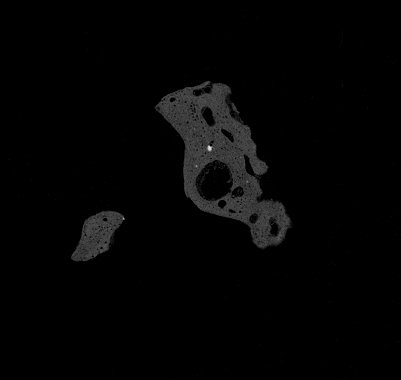

Supplement: S5 File — (ZIP) [file pone.0228610.s005.zip › 32_144/Br-2_IR_rec1654.jpg]

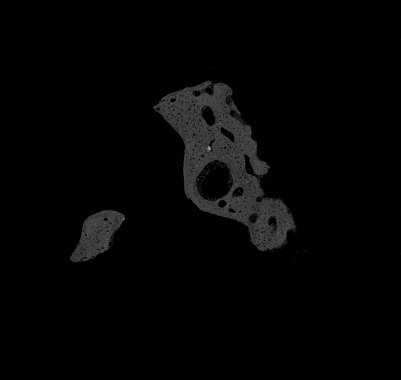

Supplement: S5 File — (ZIP) [file pone.0228610.s005.zip › 32_144/Br-2_IR_rec1658.jpg]

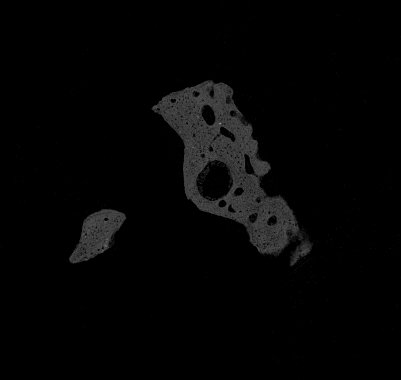

Supplement: S5 File — (ZIP) [file pone.0228610.s005.zip › 32_144/Br-2_IR_rec1662.jpg]

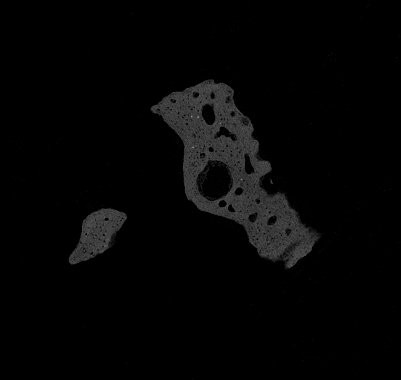

Supplement: S5 File — (ZIP) [file pone.0228610.s005.zip › 32_144/Br-2_IR_rec1666.jpg]

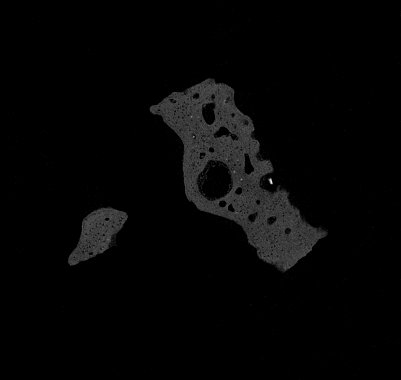

Supplement: S5 File — (ZIP) [file pone.0228610.s005.zip › 32_144/Br-2_IR_rec1670.jpg]

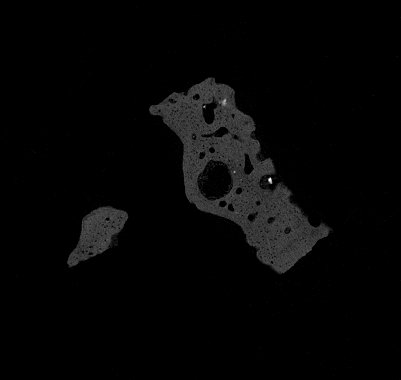

Supplement: S5 File — (ZIP) [file pone.0228610.s005.zip › 32_144/Br-2_IR_rec1674.jpg]

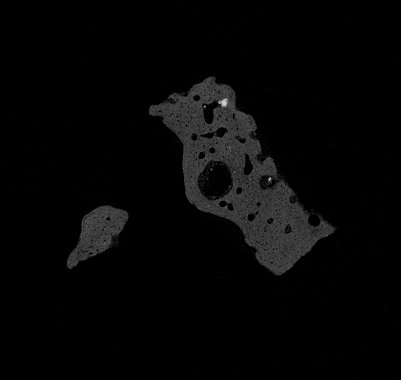

Supplement: S5 File — (ZIP) [file pone.0228610.s005.zip › 32_144/Br-2_IR_rec1678.jpg]

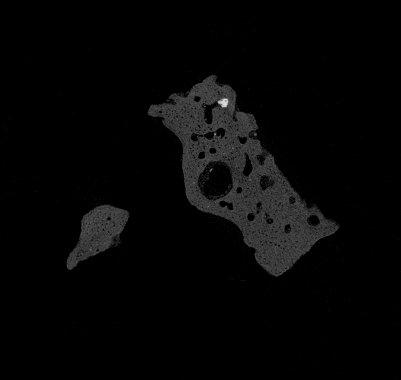

Supplement: S5 File — (ZIP) [file pone.0228610.s005.zip › 32_144/Br-2_IR_rec1682.jpg]

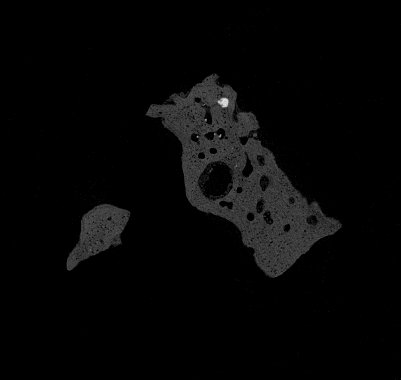

Supplement: S5 File — (ZIP) [file pone.0228610.s005.zip › 32_144/Br-2_IR_rec1686.jpg]

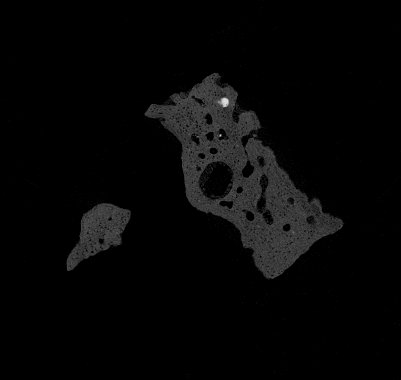

Supplement: S5 File — (ZIP) [file pone.0228610.s005.zip › 32_144/Br-2_IR_rec1690.jpg]

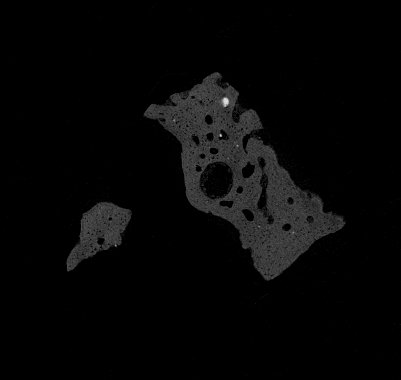

Supplement: S5 File — (ZIP) [file pone.0228610.s005.zip › 32_144/Br-2_IR_rec1694.jpg]

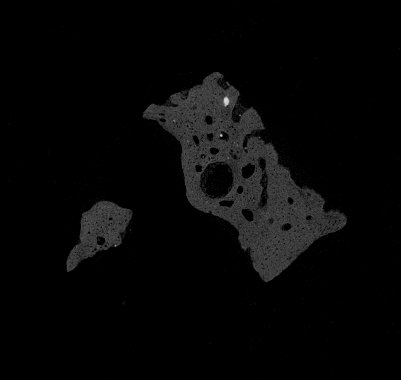

Supplement: S5 File — (ZIP) [file pone.0228610.s005.zip › 32_144/Br-2_IR_rec1698.jpg]

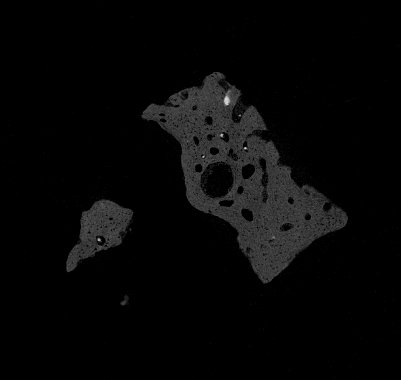

Supplement: S5 File — (ZIP) [file pone.0228610.s005.zip › 32_144/Br-2_IR_rec1702.jpg]

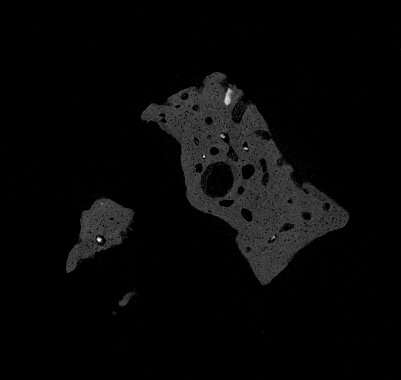

Supplement: S5 File — (ZIP) [file pone.0228610.s005.zip › 32_144/Br-2_IR_rec1706.jpg]

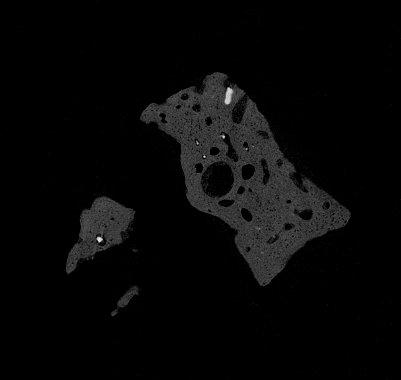

Supplement: S5 File — (ZIP) [file pone.0228610.s005.zip › 32_144/Br-2_IR_rec1710.jpg]

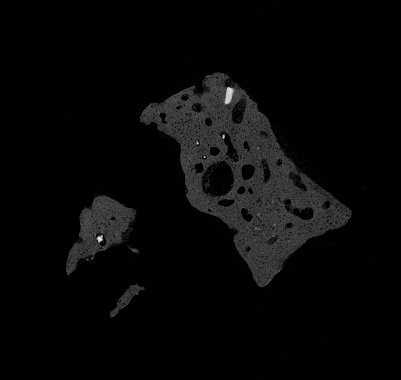

Supplement: S5 File — (ZIP) [file pone.0228610.s005.zip › 32_144/Br-2_IR_rec1714.jpg]

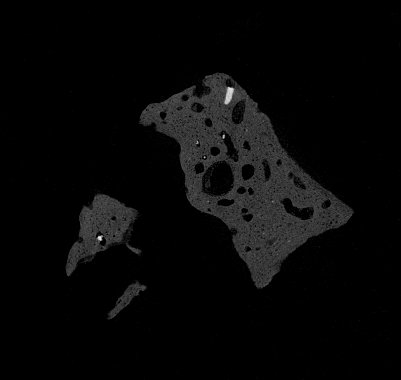

Supplement: S5 File — (ZIP) [file pone.0228610.s005.zip › 32_144/Br-2_IR_rec1718.jpg]

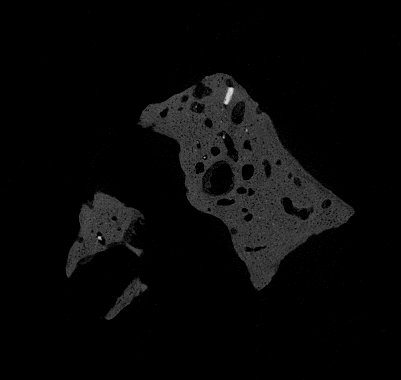

Supplement: S5 File — (ZIP) [file pone.0228610.s005.zip › 32_144/Br-2_IR_rec1722.jpg]

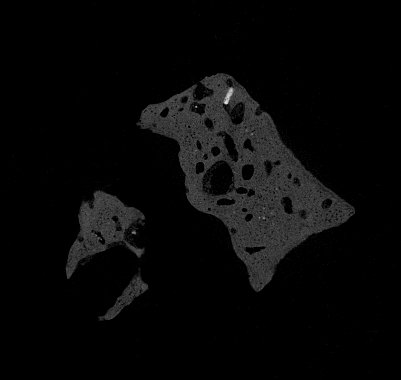

Supplement: S5 File — (ZIP) [file pone.0228610.s005.zip › 32_144/Br-2_IR_rec1726.jpg]

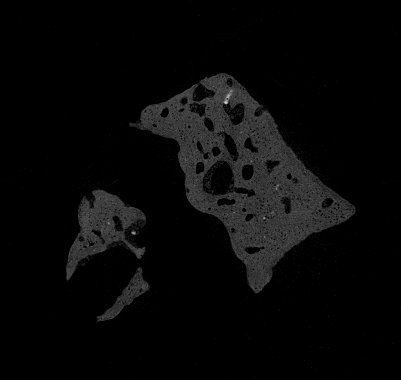

Supplement: S5 File — (ZIP) [file pone.0228610.s005.zip › 32_144/Br-2_IR_rec1730.jpg]

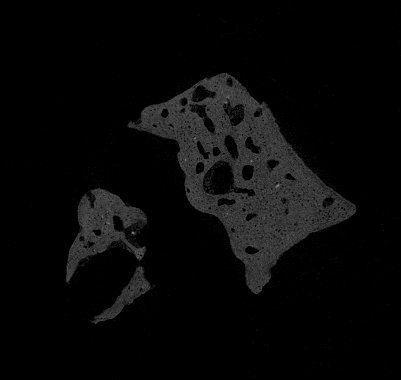

Supplement: S5 File — (ZIP) [file pone.0228610.s005.zip › 32_144/Br-2_IR_rec1734.jpg]

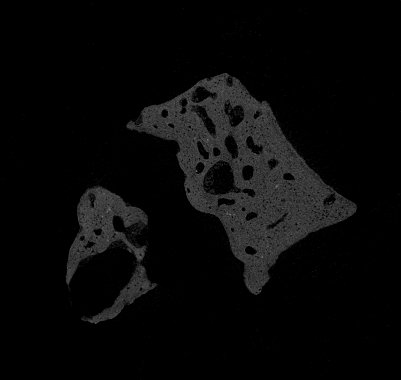

Supplement: S5 File — (ZIP) [file pone.0228610.s005.zip › 32_144/Br-2_IR_rec1738.jpg]

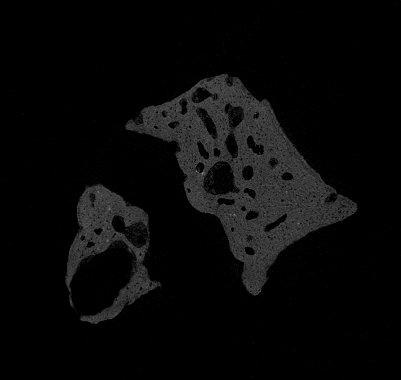

Supplement: S5 File — (ZIP) [file pone.0228610.s005.zip › 32_144/Br-2_IR_rec1742.jpg]

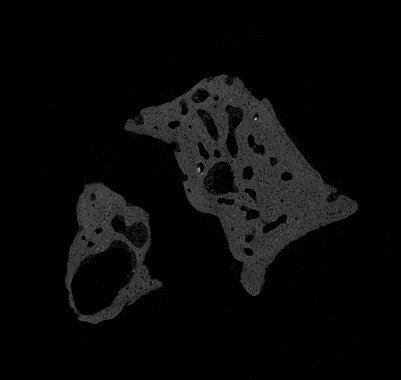

Supplement: S5 File — (ZIP) [file pone.0228610.s005.zip › 32_144/Br-2_IR_rec1746.jpg]

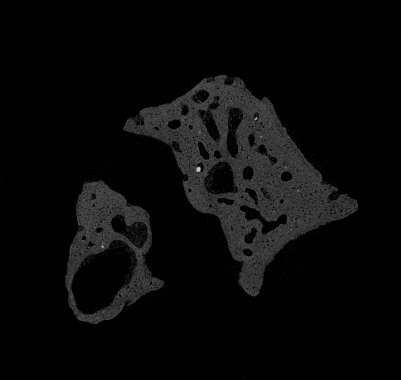

Supplement: S5 File — (ZIP) [file pone.0228610.s005.zip › 32_144/Br-2_IR_rec1750.jpg]

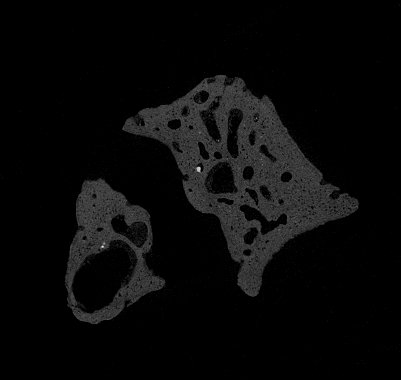

Supplement: S5 File — (ZIP) [file pone.0228610.s005.zip › 32_144/Br-2_IR_rec1754.jpg]

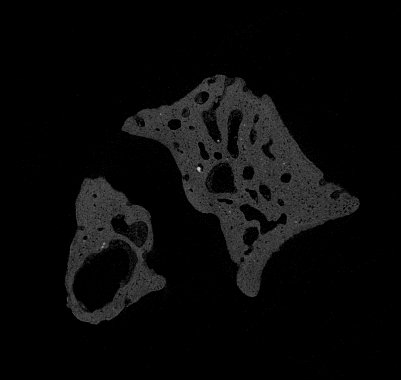

Supplement: S5 File — (ZIP) [file pone.0228610.s005.zip › 32_144/Br-2_IR_rec1758.jpg]

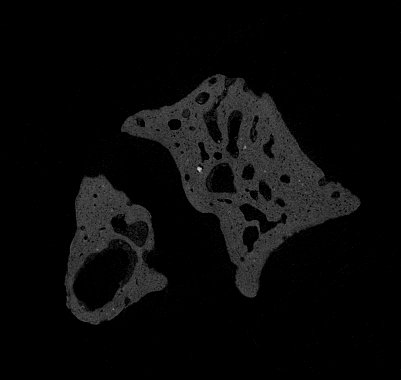

Supplement: S5 File — (ZIP) [file pone.0228610.s005.zip › 32_144/Br-2_IR_rec1762.jpg]

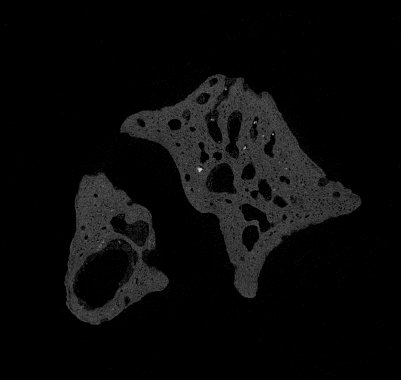

Supplement: S5 File — (ZIP) [file pone.0228610.s005.zip › 32_144/Br-2_IR_rec1766.jpg]

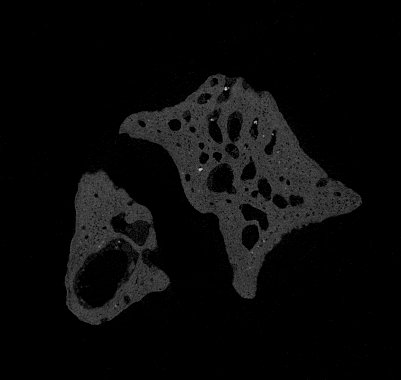

Supplement: S5 File — (ZIP) [file pone.0228610.s005.zip › 32_144/Br-2_IR_rec1770.jpg]

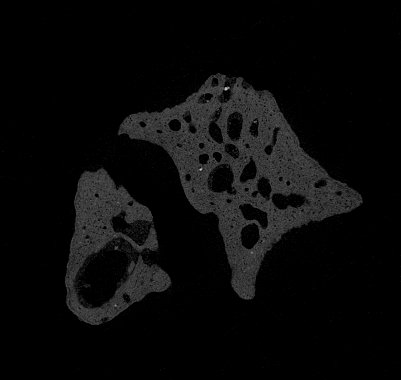

Supplement: S5 File — (ZIP) [file pone.0228610.s005.zip › 32_144/Br-2_IR_rec1774.jpg]

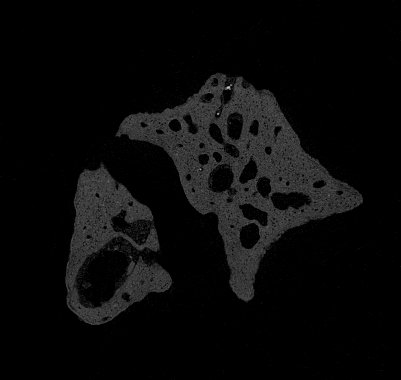

Supplement: S5 File — (ZIP) [file pone.0228610.s005.zip › 32_144/Br-2_IR_rec1778.jpg]

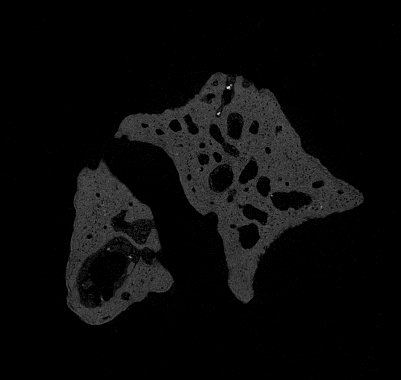

Supplement: S5 File — (ZIP) [file pone.0228610.s005.zip › 32_144/Br-2_IR_rec1782.jpg]

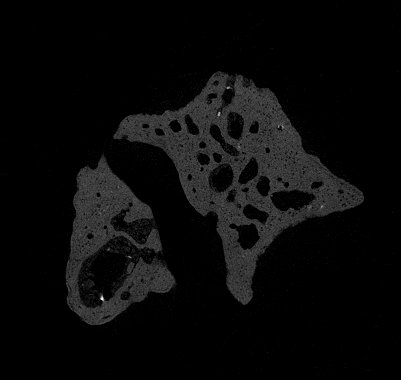

Supplement: S5 File — (ZIP) [file pone.0228610.s005.zip › 32_144/Br-2_IR_rec1786.jpg]

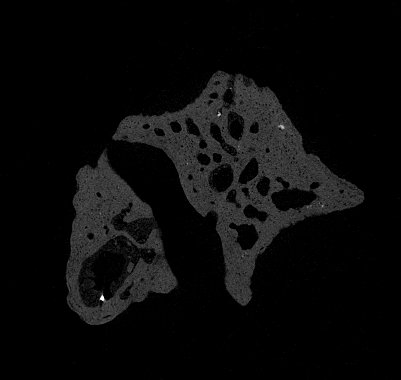

Supplement: S5 File — (ZIP) [file pone.0228610.s005.zip › 32_144/Br-2_IR_rec1790.jpg]

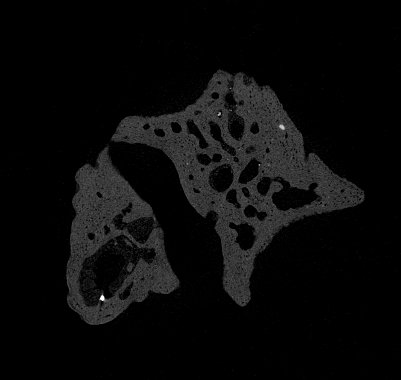

Supplement: S5 File — (ZIP) [file pone.0228610.s005.zip › 32_144/Br-2_IR_rec1794.jpg]

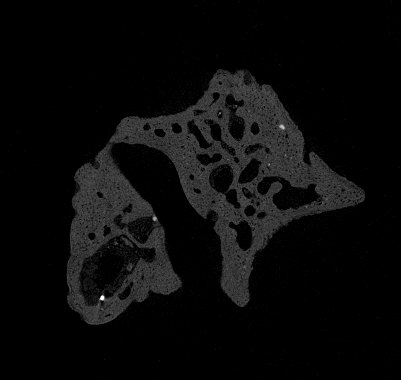

Supplement: S5 File — (ZIP) [file pone.0228610.s005.zip › 32_144/Br-2_IR_rec1798.jpg]

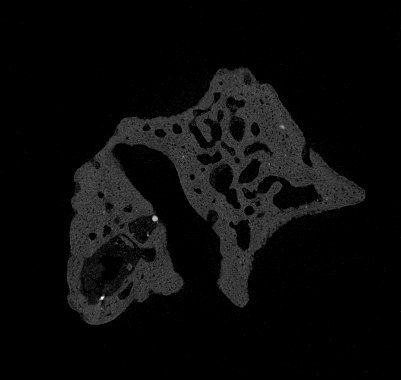

Supplement: S5 File — (ZIP) [file pone.0228610.s005.zip › 32_144/Br-2_IR_rec1802.jpg]

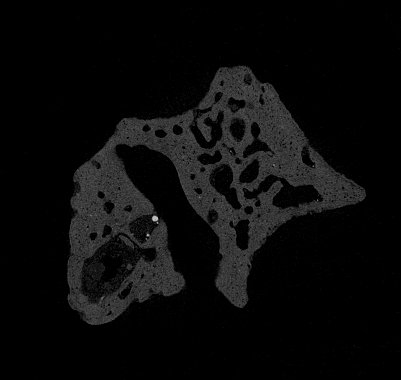

Supplement: S5 File — (ZIP) [file pone.0228610.s005.zip › 32_144/Br-2_IR_rec1806.jpg]

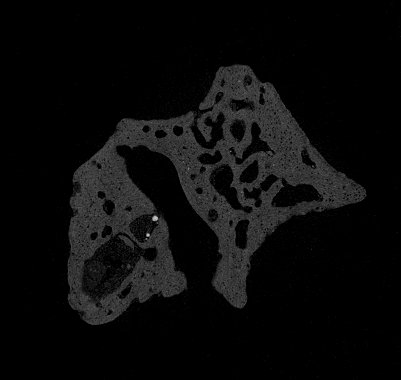

Supplement: S5 File — (ZIP) [file pone.0228610.s005.zip › 32_144/Br-2_IR_rec1810.jpg]

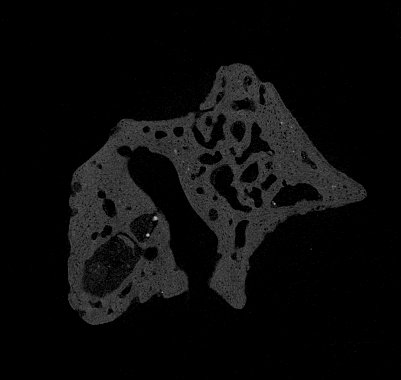

Supplement: S5 File — (ZIP) [file pone.0228610.s005.zip › 32_144/Br-2_IR_rec1814.jpg]
